# Phage satellites induced by virulent phages are mobilized by natural competence leading to phage resistance in a new host

**DOI:** 10.1038/s41467-026-72928-1

**Published:** 2026-05-12

**Authors:** Carlee Morency, Geneviève M. Rousseau, Zacharie Morneau, Sylvain Moineau

**Affiliations:** Département de biochimie, microbiologie, et bio-informatique, Faculté des sciences et de génie, Université Laval, Québec City, QC Canada

**Keywords:** Bacteriophages, Phage biology

## Abstract

A phage satellite (PS) typically resides within repeat regions (*attL* and *attR* sites) of a bacterial genome. Its genome ranges from 7 to 20-kb and includes genes encoding an integrase along with regulatory and DNA replication functions. However, it lacks genes associated with viral structural proteins. *Streptococcus thermophilus* (*S.t*.) is extensively used to produce yogurt and specialty cheeses. Intriguingly, the majority of *S.t*. strains harbor a PS while very few possess a complete prophage, suggesting that PSs may confer advantages to their hosts. In this study, we showed that PSs of *S.t*. can excise from the bacterial chromosome, at a very low rate, without any phage interaction. Furthermore, we found that they can also be induced by virulent phages. By leveraging CRISPR-Cas9, we selected *S.t*. cells devoid of any PS (delta-PS strain). Then, we mobilized a PS from one strain to a delta-PS strain, using only natural competence, bypassing the need for a helper phage. The resulting strain exhibited increased resistance to virulent phages. Through the isolation of phage mutants escaping the resistance phenotype, we pinpointed a specific phage protein responsible for the induction of a PS. Lastly, we demonstrated that a PS can be significantly induced by a virulent phage, which, in turn, greatly promotes its transfer and specific integration into new cells through natural competence. Our study introduces a novel natural approach to develop phage-resistant strains.

## Abstract (web summary)

Phage satellites are bacterial mobile genetic elements with integration and replication functions but lack genes for viral structural proteins. Here, Morency et al. show that a phage satellite in *Streptococcus thermophilus* enhances resistance to virulent phages and can be transferred to other bacterial cells via natural competence, bypassing the need for a helper phage.

## Introduction

Bacteriophages, or phages, are engaged in an ongoing arms race with their bacterial counterparts^1^. When they infect bacteria, virulent phages enter the lytic cycle during which they utilize the bacterial machinery to replicate viral genomes and produce proteins. The lytic cycle culminates with the lysis of the infected host, resulting in the release of new assembled virions in the local environment, ready to disseminate among the phage-sensitive bacterial population^2^. In contrast, temperate phages possess the ability to enter the lysogenic mode, during which they integrate their viral genome into the bacterial chromosome and remain dormant as a prophage. Subsequently, they can transition to the lytic cycle spontaneously or through induction triggered by an external factor.

Mobile genetic elements (MGEs) play a pivotal role in evolution by expanding the gene pool within a bacterial population^3,4^. Phage satellites (PSs) are a specific subset of MGEs that are integrated into the genomes of many bacterial species. Since PSs lack the genes that encode viral structural proteins, they hijack part of the assembly process of certain phages, which are referred to as helper phages. Some PSs redirect the helper phage packaging process to encapsidate their own nucleic acids within the newly formed virion capsid, instead of the helper phage genome. This partial takeover allows PSs to spread to other cells by using the virion as a delivery vehicle, while simultaneously limiting the replication and release of new helper phage infectious particles^5–9^.

Phage-inducible chromosomal islands (PICIs) are among the most extensively studied PSs^9^. They were first characterized in *Staphylococcus aureus* and are now referred to as *S. aureus* pathogenicity islands (SaPIs). Along with PICIs, SaPIs carry genes encoding an integrase, as well as those responsible for excision, replication, and packaging^10–12^. Accessory genes are also often found close to their integrase gene as well as between their *terS* and the 3′-end of their genome^9^. These accessory genes may code for various functions, including virulence, antibiotic resistance, and on occasion, phage resistance^6,13^. The SaPI excision from the bacterial genome and its subsequent replication are initiated by a temperate helper phage^14^. Much like other PICIs, SaPIs leverage the machinery of helper phages to disseminate throughout a bacterial population^13,15^. Upon entering a new host, SaPIs presumably integrate into the bacterial genome through a circular intermediate^12^. They also carry a short nucleotide region, known as the attachment site *attS*, that can recombine with the bacterial *attC* site. This SaPI integration into the bacterial genome results in the creation of two direct repeats, namely the *attL* (left) and *attR* (right) sites, positioned at each end of the integrated PS^12,16^. Thus far, studies showed that PICIs are mobilized between bacteria only through encapsidation by a helper phage^7,11,17^.

*Streptococcus thermophilus* (*S.t*.) is non-pathogenic Gram-positive lactic acid bacterium (LAB) used worldwide for the manufacturing of various fermented dairy products. However, the presence of virulent phages in milk poses an industrial risk, as they can infect and lyse these selected LAB strains, ultimately leading to low-quality fermented products^18^. To defend against virulent phages, all *S.t*. strains (100%) characterized to date rely on a chromosomally encoded type II-A CRISPR-Cas systems (referred to as CRISPR1). Some *S.t*. strains may even harbor two distinct type II-A CRISPR-Cas systems, denoted CRISPR1 and CRISPR3. These adaptive defense systems involve the initial acquisition of a 30-bp spacer from invading DNA, such as a phage genome, which is incorporated into a CRISPR array and converted into a short RNA molecule (crRNA). An endonuclease (Cas9), armed with the crRNA, can then recognize and cleave subsequent invading identical DNA sequences^19,20^. In response, virulent phages have developed strategies to counteract CRISPR-Cas systems. For example, some streptococcal phages produce anti-CRISPR (ACR) proteins that directly inactivate Cas9^21,22^. The discovery of ACR+ phages suggests that *S.t*. must adapt its defense systems to cope with evolving phages.

*S.t*. also possesses the ability to acquire free linear or circular DNA from its environment through natural competence^23^. In laboratory conditions, this process can be induced by adding a peptide (ComS) to *S.t*. competent cells^23,24^. Thus, exogeneous DNA can be introduced into *S.t*. strains in laboratory settings utilizing natural competence, but it is also a natural way for this species to acquire environmental DNA.

In this study, we revealed that more than two-thirds of *S.t*. strains harbor a PS with structural similarities to SaPIs. Furthermore, we demonstrated that virulent phages can trigger the induction of *S.t*. PSs and that upon cell lysis, their DNA can be transferred into a new strain through natural competence, offering a novel mean of mobilizing these genetic elements (Supp. Fig. 1). The exchange of PSs between *S.t*. strains was also found to enhance their overall resistance to virulent phages.

## Results and discussion

### PSs are prevalent among *S.t.* strains

**Fig. 1:**
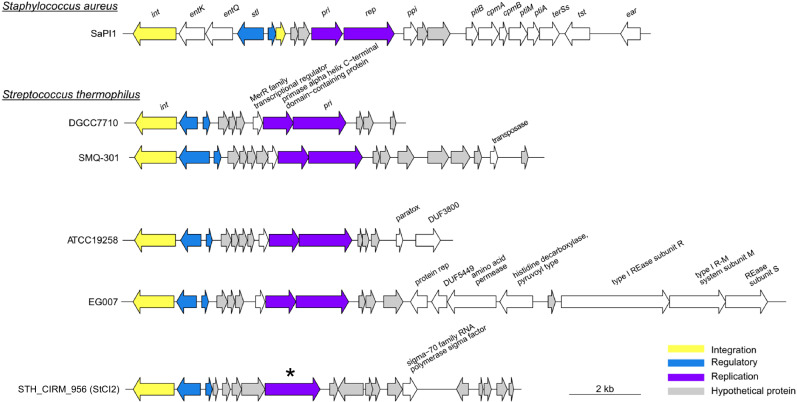
*Streptococcus thermophilus* phage satellites (StCI) display genomic diversity within a conserved organization. Genomic organization of *Staphylococcus aureus* SaPI1 and diversity of StCIs based on VirClust clusters. Annotations of StCIs are from GenBank (some were simplified) and SaPI1 annotations are from Fillol-Salom et al.^30^. Open reading frames (ORFs) are represented by arrows and the color represents their putative function. The ORF marked with an asterisk was analysed with HHphred^72,73^ and is likely a DNA primase. Genomic maps were produced in R software using genoPlotR package^55,74^.

While analyzing the genome of the model strain *S.t*. SMQ-301^25^, we noticed the presence of a putative PS. Of note, this strain does not contain a full prophage. Subsequently, we accessed 86 other complete *S.t*. chromosome sequences available on GenBank (Table S1). Through sequence alignments, we found that 69% (60/87) of them contained a putative PS (Supp. Fig. 2). These *S.t*. PSs exhibited characteristics previously reported for SaPIs and other types of PICIs^6,9^, including direct repeats at the ends on their genome, an integrase gene as well as regulatory and DNA replication proteins (Fig. 1).

Using the SatelliteFinder software^26,27^, we confirmed the presence/absence and also the type of PSs in the *S.t*. genomes (Table S1). Of note, two *S.t*. PSs (in strains STH_CIRM_1048 and STH_CIRM_1049) from our sequence alignments were not detected by SatelliteFinder. According to SatelliteFinder, most of the identified *S.t*. PSs are type C PICIs, whereas 8 *S.t*. PSs are type D PICIs (Table S1)^26,27^. Therefore, *S.t*. PSs will now be referred to as StCIs (*S. thermophilus* chromosomal islands).

StCI+ strains typically harbor only one StCI, except for strain STH_CIRM_956 (LR822020.1), which carried two distinct StCIs integrated at different chromosomal insertion sites (Table S1). Furthermore, at least 16 StCIs carried a probable phage resistance mechanism such as a restriction-modification system and/or an abortive infection system (Supp. Fig. 2). It is worth noting that anti-phage systems have been previously identified in PSs from other bacteria^7,28–30^.

StCI sequences displayed a slightly lower GC content (33–38%) compared to their host ($\sim$39%) (Table S1) as observed for other StCIs^5^. We delimited the StCIs by their direct repeats (16–18 bp), with the *attL* located upstream of the *int* gene at their 5′-end and the *attR* positioned at the 3′-end of the StCI. We detected five StCI integration sites: IS_a (associated with 45 StCIs), IS_b (5 StCIs), IS_c (7 StCIs), IS_d (3 StCIs), and IS_e (1 StCI) (Table S1). Some of the repeat sequences had nucleotide variations, indicating that further investigation is needed to confirm the mobilization of these StCIs (Table S1).

Homology searches indicated that the StCIs could be grouped into at least five clusters based on their accessory genes as well as the integrase gene (Supp. Fig. 2). Interestingly, StCIs belonging to cluster 3 possessed a distinct integrase in comparison to other StCIs. In fact, members of this StCI group exhibited close similarity (> 99% nucleotide identity, >85% coverage) to the PS (MK448403.1)^5^ found in the genome of *Streptococcus macedonicus* strain 33MO, a bacterium also associated with dairy products^31^ (Supp. Fig. 3). The StCIs from cluster 3 and the S. *macedonicus* PS even shared the same integration site in their respective genomes (IS_c; Table S1). The StCIs from cluster 3 also have similarity (> 97% nucleotide identity, >50% coverage) with a putative PS found in *Streptococcus dysgalactiae* strain DB60705-15 (CP033165.1), which is a human pathogen^32^ (Supp. Figs. 2 and 3). They also share the same integration site (IS_c; Table S1). These findings suggest that some StCIs could have been mobilized between streptococcal species akin to what was observed with certain SaPIs^9^.

### Phage satellites spontaneously excise from the *S. thermophilus* chromosome

**Fig. 2:**
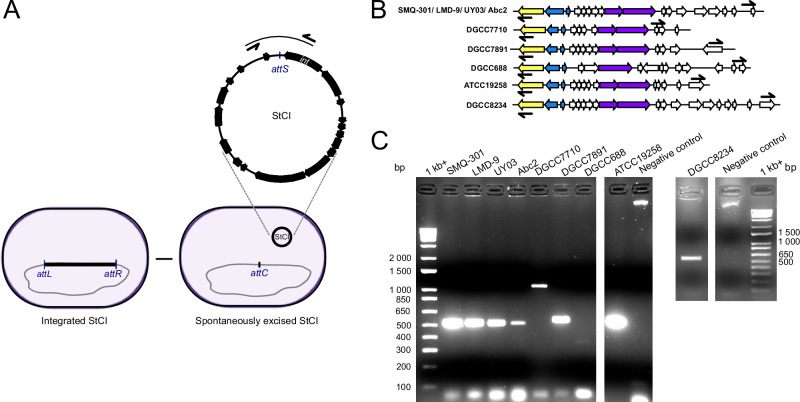
StCI can spontaneously excise from the bacterial chromosome. Circularized StCIs were detected by PCR in overnight cultures of nine *S.t*. strains, using primers specific to both StCI ends. **A** Integrated StCI can excise from the chromosome without any phage infection. Schematic representation of StCI spontaneous excision. **B** Genomic organization of StCIs in the tested *S.t*. strains. Open reading frames (ORFs) are represented by arrows and the color represents their putative function as in Fig. 1. **C** PCR products migrated on a 2% agarose gel. Nine *S.t*. strains tested and the expected size of PCR products: SMQ-301/506 bp, LMD-9/506 bp, UY03/506 bp, Abc2/506 bp, DGCC7710/1082 bp, DGCC7891/541 bp, DGCC688/357 bp, ATCC19258/566 bp, and DGCC8234/566 bp. The PCR reactions were conducted on bacterial overnight cultures and were also repeated on extracted genomic DNA (Supp. Fig. 4). StCI linear genomic maps were produced in R software (version 4.3.2) using genoPlotR package^55,74^.

To check for the activity of the StCIs, we performed a PCR assay using divergent primers specific to both ends of the StCIs found in nine *S.t*. strains: SMQ-301, UY03, Abc2, LMD-9, DGCC7710, DGCC7891, DGCC688, ATCC19258, and DGCC8234 (Fig. 2). Specifically, the primers were designed to detect circularized or concatemeric StCI DNA after their excision from the bacterial genome (Supp. Table S9). Strains were cultured overnight in liquid medium at 37 °C and the PCR performed directly on these cultures. Among those strains for which their genome is available in GenBank, there is no full prophage in strains SMQ-301, DGCC7710, and LMD-9, but there is one in ATCC 19258.

Our data clearly showed that all StCIs were excised from the genome of the 9 *S.t*. strains tested, as their excised forms were detected by PCR performed on overnight cultures (Fig. 2 and Supp. Fig. 4). These findings are consistent with the spontaneous excision observed in some SaPIs^16^, providing strong evidence of the active status of StCIs. The sequencing of the PCR products also confirmed the *attC* sequence for the three most common StCI integration sites: 5′-ATATTTCCCTCTAAAATC-3′ (IS_a), 5′-ATTCCATGAAAAAATAC-3′ (IS_b), and 5′-AATTCTACAACAAAAT-3′ (IS_c) (Table S1). Our results also revealed that StCIs integrated at the same *attC* site also possessed the same (or almost identical) *attS* site, indicating a specific pattern of StCIs integration into *S.t*. genome (Table S1). Interestingly, several StCIs located at the IS_b site, including the StCI of strain DGCC8234, have a single nucleotide variation (SNV) in their *attL* site compared to their *attR* site (Table S1). The sequencing of PCR products targeting the excised/integrated DGCC8234 StCI revealed that following the StCI excision, the *attL* site becomes the *attC* site and the *attR* site becomes the *attS* site. Because we could detect excised StCIs and pinpoint the *attC* site, the precise genomic positions of most StCIs^5,26^ could be identified (Table S1).

### Some *Streptococcus* sp. strains carry spacers that target phage satellites

As StCIs are readily excised from the chromosome of *S. thermophilus* strains and since this bacterial species heavily relies on CRISPR-Cas system to defend against MGEs, we searched in databases for CRISPR spacers targeting StCIs. We found that several *S.t*. strains, along with a few strains from other *Streptococcus* species, carry StCI-targeting spacers in their CRISPR loci (Table S2). All *S.t*. strains except one (KLDS 3.1003) that carry StCI-targeting spacers in their CRISPR1 or CRISPR3 array harbor no StCI targeted by the spacers in their array. In some cases, a StCI that is not targeted by the strain’s spacers is present. In the case of KLDS 3.1003, this *S.t*. strain possesses a spacer that targets its own StCI. However, we found three mutations in the PAM motif of the protospacer, indicating that the CRISPR-Cas system would not cleave this StCI DNA, explaining the co-occurrence of the spacer and the StCI in this strain^33^.

We also observed six strains from other streptococcal species (*S. salivarius*, *S. gallolyticus*, *S. macedonicus*, and *S. agalactiae*) that carried at least one spacer targeting PSs (Table S2). Rezaei Javan et al.^5^ previously detected the presence of PSs in *S. salivarius*, *S. gallolyticus*, and *S. agalactiae*, underscoring the significance of these MGEs in the *Streptococcus* genus.

By aligning each spacer with the NCBI BLASTn database, we identified three spacers that specifically target a StCI region, as these protospacers were not found in other genomic contexts. One of these StCI-specific spacers is carried by *S. gallolyticus* ICDDRB-NRC-S1, and targets a StCI transcriptional regulator (regulatory module) (Table S2). Another is carried by *S. thermophilus* TH985 and targets a StCI gene coding for a hypothetical protein. The last spacer was found in *S. thermophilus* FAM 13496 and also targets a gene coding for a hypothetical protein that is found in StCIs, but also in a *S. macedonicus* PS (accession number MK448403.1). Interestingly, we also identified one spacer carried by strains *S. salivarus* ATCC 25975 and NCTC7366 that targets a gene coding for a StCI integrase with two mismatches. However, it matches a protospacer in the integrase gene from a PS in *S. salivarus* with one SNV (with 100% coverage and 96.67% identity, accession number CP018186).

Taken altogether, it is tempting to speculate that CRISPR-Cas may serve as a defense system against PSs and that PS DNA can be mobilized between different streptococcal species.

### *S. thermophilus* strains with no StCI can be selected using CRISPR-Cas9

**Fig. 3:**
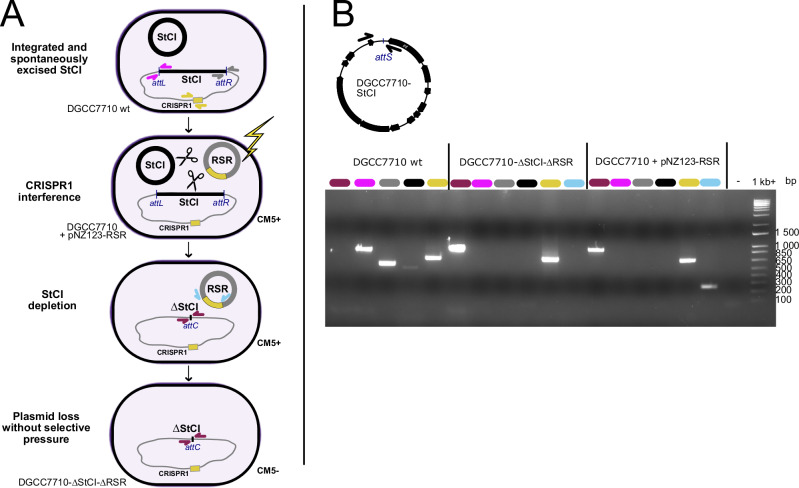
Endogenous CRISPR–Cas enables generation of StCI-Free *Streptococcus thermophilus* DGCC7710. **A** Schematic representation of the methodology to obtain StCI-free strains. **B** The presence/absence of StCIs was verified by PCR. The presence/absence of the plasmid containing the crRNA (RSR) was verified by PCR before and after serial passages in liquid growth medium without antibiotics (CM5-). PCR products were migrated on a 2% agarose gel. PCR primers used are color-coded in panels A and B (top of the gel). This experiment was repeated with other strains (Supp. Fig. 6). CM5 Chloramphenicol 5 µg/ml, RSR pNZ123 plasmid containing the crRNA, *int* StCI integrase, CRISPR1 Clustered regularly interspaced short palindromic repeats 1 locus in *S. thermophilus*.

As StCIs are spontaneously excised from the bacterial chromosome and certain *S.t*. strains harbor spacers that target these StCIs, we hypothesized that we could isolate StCI-free cells by harnessing their endogenous CRISPR-Cas activity. First, a mini CRISPR array (repeat-spacer-repeat unit, RSR) was cloned into the high-copy vector pNZ123, which contains a chloramphenicol resistance gene for selection. The spacer was designed to target the integrase gene (*int*) of the StCI found in *S.t*. strain SMQ-301. We then electroporated this plasmid into three *S.t*. strains: SMQ-301, DGCC7710, and UY03. For a fourth *S.t*. strain DGCC7891, a different spacer was used in the RSR plasmid because its endogenous CRISPR-Cas system uses a different PAM. The plasmid was also transformed into *S. thermophilus* DGCC7891 through natural competence^23^ since electroporation repeatedly failed. Following transformation, colonies were selected for the four strains on chloramphenicol-containing plates (5 µg/ml, CM5) and the presence or absence of StCIs in these colonies was confirmed by PCR. Colonies lacking StCI ($\Delta$StCI) were readily recovered from the four strains (Fig. 3 and Supp. Fig. 5). We then eliminated the targeting RSR plasmids by sequentially cultivating the bacteria in liquid medium without antibiotic selective pressure.

Then, we verified if the loss of the StCI changed the strains’ phage sensitivity profile when compared to the wild-type strains. The virulent phages 2972, D1126, and D4752 were amplified on *S.t*. strain DGCC7710-ΔStCI-ΔRSR, phages DT1 and 73 on SMQ-301-ΔStCI-ΔRSR and UY03-ΔStCI-ΔRSR, and phages D4446 and P738 on DGCC7891-ΔStCI-ΔRSR. Each phage was tested on their (ΔStCI) propagation host containing the pNZ123-RSR and on their corresponding wild-type host (StCl+) containing pNZ123. No major difference (< 1 log) in phage titers were detected (Table S3). We used the propagation host containing the pNZ123-RSR plasmid as a control to avoid any potential effect caused by the emergence of mutations during plasmid curing.

These findings demonstrate that CRISPR-Cas system can effectively facilitate the recovery of *S.t*. cells without StCIs.

### StCIs can be mobilized in other strains via natural competence

**Fig. 4:**
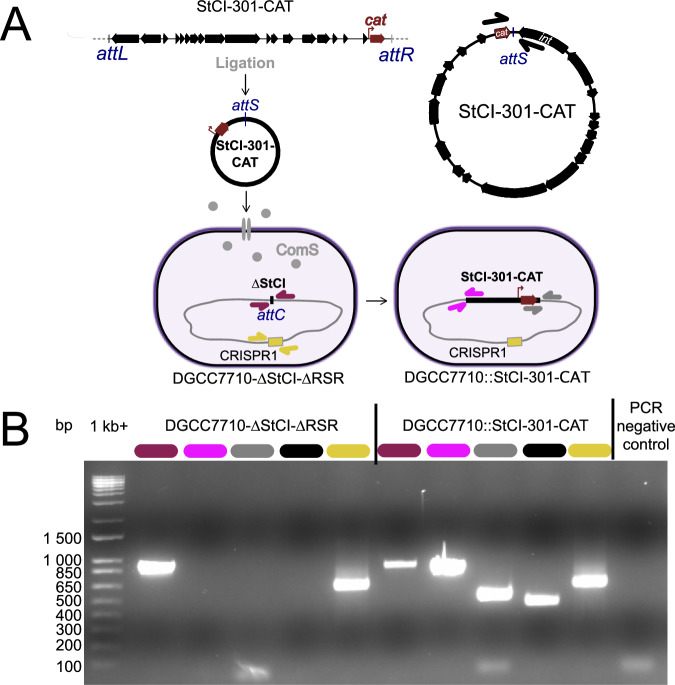
Mobilization of the engineered StCI-301-CAT element from *S. thermophilus* SMQ-301 into *S. thermophilus* DGCC7710-ΔStCI-ΔRSR via natural competence. The StCI from strain *S.t*. SMQ-301 and containing an engineered chloramphenicol resistance gene (StCI-301-CAT) was mobilized into the strain *S.t*. DGCC7710-∆StCI-∆RSR by natural competence using the ComS peptide. **A** The presence of the integrated StCI-301-CAT was confirmed by PCR using SP_circ_R/SMQ_F primers (*attL* end) and CAT-circ-F/SMQ_R (*attR* end). The empty bacterial attC was verified using SMQ_F/SMQ_R primers and the excised StCI with CAT-circ-F/ SP_circ_R primers. The strain ID was confirmed by the spacer content of its CRISPR1 locus. The no-DNA control from the same transformation was also verified by PCR. **B** PCR products migrated on a 2% agarose gel. PCR primers used are color-coded in panels A and B. The StCI-301-CAT integration was also confirmed in two other tested colonies. A similar experiment was reproduced in other strains using StCI-301-CAT-rev (Supp. Fig. 7). CAT Chloramphenicol acetyltransferase, *int* StCI integrase, CRISPR1 Clustered regularly interspaced short palindromic repeats 1 locus in *S. thermophilus*.

Since *S. thermophilus* is capable of natural competence and given that StCIs can be induced and potentially released via cell lysis, we hypothesized that *S.t*. cells might acquire a new StCI through natural transformation. Thus, we moved the StCI of *S.t*. SMQ-301 into various strains in which we had removed their StCl. To aid in detecting the mobilization, we first inserted a chloramphenicol resistance gene (*cat*) with a promoter into the accessory region of the StCI from strain *S.t*. SMQ-301. This gene and promoter were added in both orientations in the StCI and the two engineered StCIs were referred to as StCI-301-CAT and StCI-301-CAT-rev (Fig. 4 and Supp. Fig. 6). We then PCR-amplified the StCI-301-CAT and StCI-301-CAT-rev from its *attL* site to just before its *attR* site. Since an induced SaPI forms a circular structure^12^, we recreated this form with StCI-301-CAT(-rev) by ligating the PCR product covering the whole StCI length.

To create a DGCC7710 variant containing the StCI of SMQ-301, the ligated StCI-301-CAT mixture was transformed into DGCC7710-$\Delta$StCI-$\Delta$RSR using natural competence (Fig. 4). We also transformed StCI-301-CAT-rev into the same strain and into two other strains lacking their StCI (DGCC7891-$\Delta$StCI-$\Delta$RSR and UY03-$\Delta$StCI-$\Delta$RSR) (Supp. Fig. 6A). We also transformed the ligated StCI-301-CAT-rev into *S.t*. S4, a strain naturally devoid of a StCI (Supp. Fig. 6B and Table S1). Colonies were selected on CM5 plates and screened for the presence of StCI-301-CAT or StCI-301-CAT-rev by PCR.

The ligated StCI-301-CAT-rev was successfully transferred in all four *S.t*. strains (Supp. Fig. 6) and the StCI-301-CAT in DGCC7710-$\Delta$StCI-$\Delta$RSR (Fig. 4). Both StCIs were integrated in the same and expected genomic location (IS_a). In contrast, when the StCI-301-CAT-rev unligated linear PCR product was transformed into the $\Delta$StCI-$\Delta$RSR strains with a DNA amount equivalent to the one used with the ligated DNA mixture, no colonies containing the integrated StCI were detected, highlighting the need of ligating the PCR product for it to be successfully transformed.

We also PCR-amplified the StCI-301-CAT-rev just after its *attL* site to its *attR* site. Then, the linear and ligated PCR products (as described above) were transformed into DGCC7710-$\Delta$StCI-$\Delta$RSR and obtained StCI+ colonies only with the ligated PCR product.

These results align with the SaPI-dependant excision-circularization and integration model, where the circular form of SaPIs can integrate in the bacterial chromosome through the *attS* and *attC* sites^16^. We anticipated that the ligated StCI DNA would integrate into the $\Delta$StCI chromosome at the *attC* site. The linear StCI might have fail to circularize, which could have lead to poor replication and low integrase dosage, thus preventing integration.

Also, it is crucial to note that during natural competence in some Gram-positive bacteria, including in *S.t*., DNA enters the cell as a single-stranded DNA (ssDNA) and can integrate the host chromosome through homologous recombination^24^. Here, it is likely that the relatively short (18 bp) homologous sequence between the *attS* site on the linear StCI PCR product and the *attC* site in the recipient cells was insufficient for homologous recombination during natural transformation, as homologous sequences exceeding 500 bp are typically used for natural transformation experiments using LAB strains^34^.

Yet, it is possible to transform circular plasmids through natural competence, even if the incoming plasmid is converted into ssDNA, since its reconstitution does not rely on sequence homology^24^. A similar process likely occurred with the StCI ligation product. It is also possible that linear concatemeric StCI DNA from the ligation product entered the recipient cells and individual StCIs excised from the concatemer, recreating the circularized StCI, as it was previously shown with the SaPIbov1 island in *S. aureus*^12^. Interestingly, we tried to introduce the StCI-301-CAT-rev into a derivative of DGCC7710-$\Delta$StCI-$\Delta$RSR that carried a spacer targeting the *cat* gene, but no colonies were obtained. This supports the notion that CRISPR-Cas system can block invading StCIs.

Taken altogether, we successfully mobilized a StCI into various *S.t*. strains, at a specific integration site, through natural competence and without any helper phage.

### StCIs exhibit low-level of spontaneous excision without detectable replication, regardless of the bacterial growth phase

To determine whether StCIs can also be spontaneously induced in a new strain, and if so, to what extent, we quantified their excision and replication levels across different growth phases. Strains SMQ-301, DGCC7710, and DGCC7710::StCI-301-CAT were grown in liquid medium at 42 °C and samples were collected at OD_600_ 0.2, 0.4, 0.8, and after an overnight (ON) incubation. We extracted the genomic DNA and conducted a relative qPCR assay using several primers targeting the StCI primase (*pri*) gene, excised StCI, integrated StCI, and both StCI flanking genes to detect empty *attC* sites. The relative quantity of each target was normalized to the housekeeping host genes *gyrA* and *gyrB* and to the sample at OD_600_ 0.2 for each strain, using the $\triangle \triangle$Cq method^35^ (Supp. Fig. 7).

No correlation was found between the StCI excision/replication levels and the growth phase of the three strains. However, there were significantly more empty *attC* sites in SMQ-301 samples at OD_600_ 0.8 and ON than in the OD_600_ 0.2 sample (Supp. Fig. 7 and Table S4). There was also more excised StCI in the OD_600_ 0.8 sample compared to the OD_600_ 0.2. But the differences were less than twofold. There was no significant difference between the other SMQ-301 samples for all the targets.

To compare the StCI excision and replication levels at the strain level, we used the relative qPCR data from the previous experiment (Supp. Fig. 7), but only with the samples incubated overnight for each strain. Then, we applied the ∆Cq method^36^. Briefly, each target was normalized against the housekeeping genes *gyrA* and *gyrB* quantities in each strain (Supp. Fig. 8). This analysis reveals the relative quantity of the empty *attC* sites, excised StCI, the integrated StCI, and StCI primase in each strain, from ON cultures. Our results show that spontaneous excision is a rare event, since the relative quantity of the excised StCI and empty *attC* sites were significantly lower than the integrated StCI and the StCI primase in all tested strains (Supp. Fig. 8 and Table S4). The empty *attC* sites were also significantly lower than the excised StCI in all the strains. However, the empty *attC* site target had high cq values in the DGCC7710 strain ( > 30 cq), which makes the corresponding relative quantities less reliable. The StCI *pri* levels were not significantly different than the housekeeping genes *gyrA* and *gyrB* and the integrated StCI targets. Thus, StCIs can be spontaneously excised, even in a new host, at low excision levels with no detectable replication, and independent of the growth phase.

### StCIs can provide phage resistance

**Fig. 5:**
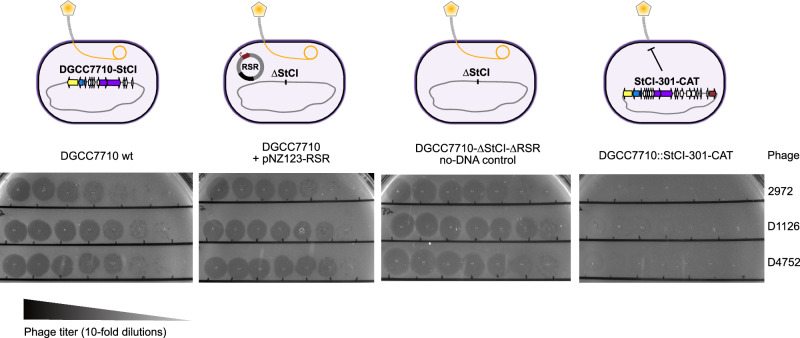
Phage-sensitivity profiles of *S. thermophilus* strains with StCI, targeting StCI, lacking StCI, or carrying the StCI-301-CAT element. The wild-type (wt) strain *S.t*. DGCC7710 with its native StCI, the *S.t*. DGCC7710 derivative containing a plasmid targeting its StCI (DGCC7710 + pNZ123-RSR), and DGCC7710 without its native StCI and without the StCI-targeting plasmid (DGCC7710-∆StCI-∆RSR no-DNA control) are phage-sensitive. The strain *S.t*. DGCC7710::StCI-301-CAT, which carries the StCI from strain *S.t*. SMQ-301 engineered to include a chloramphenicol resistance gene (*cat*) and introduced into DGCC7710-∆StCI-∆RSR via natural competence, was resistant to virulent phages. The same chloramphenicol resistance gene is found in StCI-301-CAT and in pNZ123-RSR. Phage plaque results are representative of at least three assays.

It was proposed previously that some PSs in *E. coli* and *S. aureus* encode antiviral systems^7,29^. To examine whether a StCI could confer phage resistance in its new host, we challenged *S.t*. strain DGCC7710::StCI-301-CAT with the virulent phages 2972, D1126, and D4752. These phages use a *pac*-type packaging system. Interestingly, introducing StCI-301-CAT into DGCC7710-$\Delta$StCI-$\Delta$RSR led to increased resistance against the three phages, with the strongest resistance being against phage 2972 (Fig. 5). We then investigated whether the orientation of the *cat* gene and its promoter in the StCI could influence the phage resistance phenotype. The orientation of the *cat* gene and its promoter had no impact on phage resistance (Supp. Fig. 9). To confirm that the phage resistance phenotype was due to StCI−301-CAT, it was removed from DGCC7710::StCI-301-CAT using CRISPR-Cas9, and the phage sensitivity was restored (Supp. Fig. 10).

Since the StCI−301 doesn’t provide a phage resistance phenotype in its native *S.t*. SMQ-301 host (as described in the section entitled *S. thermophilus strains with no StCI can be selected using CRISPR-Cas9*), it is likely that the some virulent phages have already evolved to bypass their host’s StCI-encoded phage defense system, or that there is an incompatibility between these phages and said defense system.

Given that StCI−301-CAT provided phage resistance when introduced into a new host, we sought to validate whether the native StCI-301, devoid of the antibiotic resistance gene, could also confer phage resistance in a new host. We considered using virulent phages as a selective marker, but due to the high activity of CRISPR-Cas systems in *S. thermophilus*, this would likely lead to the selection of phage-resistant colonies through spacer acquisition, hindering the process of screening for the presence of a new StCI. To address this, we introduced the plasmid pNZ123-ACRIIA5^21^ into *S.t*. DGCC7710-$\Delta$StCI-∆RSR. This plasmid carries a gene coding for the anti-CRISPR protein AcrIIA5. which effectively blocks both type II-A CRISPR-Cas systems in *S. thermophilus*^21,22^.

A ligated PCR product of the wild-type StCI-301 was then transformed into the strain DGCC7710-$\Delta$StCI-∆RSR + pNZ123-ACRIIA5 via natural competence. We also conducted a control experiment in which we added no DNA in the transformation assay. The recovery medium from both conditions was resuspended in 10 ml of CM5-containing medium (to maintain the plasmid pNZ123-ACRIIA5) and incubated overnight at 37 °C. Transformants were successfully selected when challenged with phage 2972 (Supp. Figs. 11 and 12). In the StCI-301 transformation, we obtained an average of 1.92 ± 1.28 × 10^8^ cfu/ml in the condition with no phage selective pressure and 599 ± 30 cfu/ml with the phage selective pressure. The StCI-301 integration was detected by PCR only in the colonies selected with phages (Supp. Fig. 12). Importantly, the introduction of the native StCI-301 provided phage resistance to *S.t*. DGCC7710, confirming that the chloramphenicol resistance cassette had no role in driving phage resistance in our prior experiments.

The findings above indicate that the transfer of an StCI from one strain to another by natural competence can increase the phage resistance of a given *S.t*. strain.

### StCI-301-CAT is induced by a phage-encoded protein

To gain insight into the new phage resistance phenotype, we investigated whether the infection by phages 2972, D1126, and D4752 could trigger the excision of StCI-301-CAT within its new host DGCC7710. Both strains DGCC7710 and DGCC7710::StCI-301-CAT were infected with each of these phages at a MOI of $\sim$5, and samples were collected at time intervals (15-, 20-, and 25-min post-infection). PCR reactions were performed with primers targeting the excised StCI and with primers targeting the CRISPR1 locus separately. An increase in the excised StCI-301-CAT was observed over time in the cultures infected with each of the three phages (Supp. Fig. 13). In contrast, we did not observe an increase in the excision of the native StCI in the phage-infected DGCC7710 cells. These results demonstrate that virulent phages can promote the excision of the StCI-301-CAT when it is integrated into a new host but cannot promote the excision of the native StCI present in the wild-type DGCC7710.

**Fig. 6:**
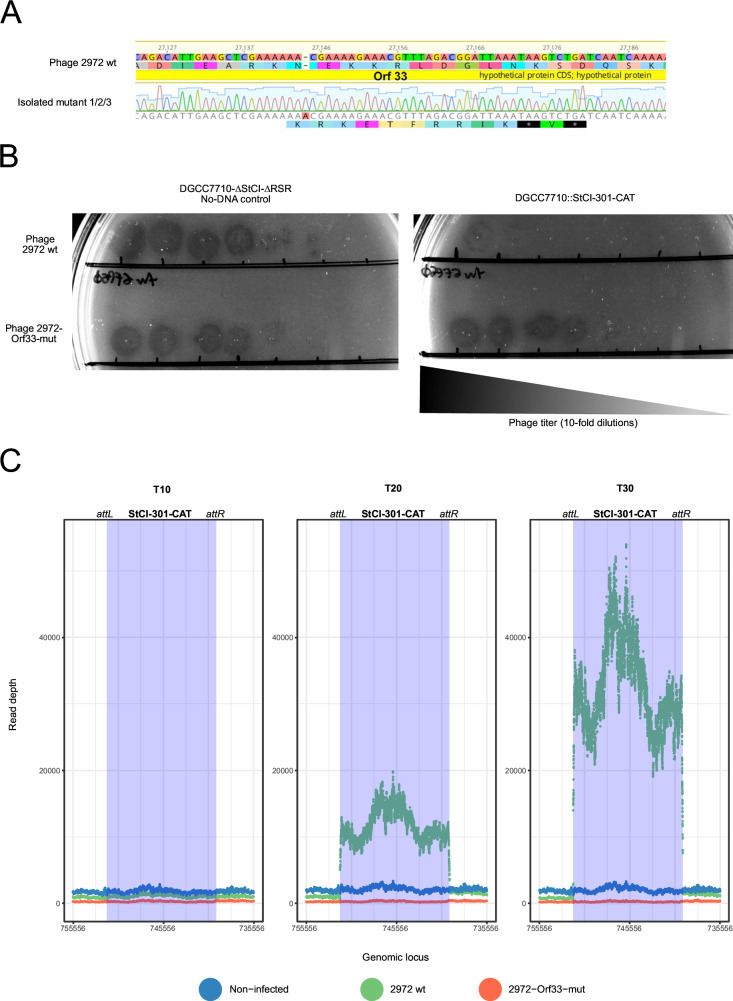
StCI-301-CAT is induced by a phage-encoded protein. **A** Alignment of a partial region of the *orf33* (GenBank accession number YP_238516.1) of the wild-type phage 2972 and of three phage mutants isolated on strain *S.t*. DGCC7710::StCI-301-CAT. Analysis conducted with Geneious software (version 11.1.5). **B** Phage resistance profile of *S. thermophilus* strains. The StCI of strain *S.t*. SMQ-301 was first engineered to contain a chloramphenicol resistance gene (*cat*) and was then mobilized into *S.t*. DGCC7710-∆StCI-∆RSR by natural competence. A control with no added DNA was carried on in parallel. The resulting *S.t* strain (DGCC7710::StCI-301-CAT) was more resistant to the wild-type phage 2972 but sensitive to a phage mutant that contained a mutation in its *orf33*. The colonies obtained from the no-DNA transformation control were sensitive to both phages. Plaque assays are representatives of three experiments. **C** Orf33 of phage 2972 is required for the induction of the chromosomally integrated StCI-301-CAT in *S. thermophilus* DGCC7710::StCI-301-CAT. This strain (also named SMQ-1512) was infected with either phage 2972 (green) or phage mutant 2972-Orf33-mut (red) at a MOI of 10. A non-infected bacterial control (blue) was carried out in parallel. Bacterial samples were collected at 10 (T10), 20 (T20), and 30 (T30) min post-infection. Genomic DNA was extracted and Illumina sequenced. A gradual increase of the read depth at the StCI-301-CAT genomic position was observed over time only when the strain was infected with the virulent wild-type phage 2972. Paired-end reads were mapped to the DGCC7710::StCI-301-CAT sequence (only a small region of the genome is shown), created using the GenBank NZ_CP025216 file and the StCI-301-CAT sequence. Results are a representative of two biological replicates. Chromosomal genomic positions are indicated under each graph. CAT Chloramphenicol acetyltransferase.

While StCI-301-CAT provided phage resistance to its new host, the protection was not absolute as phage plaques could still be observed at high phage concentrations (Fig. 5). We isolated three escape phage plaques from the infection of strain DGCC7710::StCI-301-CAT by phage 2972. Additionally, as a control, we purified three plaques from the infection of strain DGCC7710 by the wild-type phage 2972. A point mutation was identified in the genome of the three escape phages (Fig. 6A). Specifically, a nucleotide was inserted at the 179th bp of the phage’s *orf33*, resulting in a frameshift and a stop codon at the 70th amino acid of Orf33. The three isolated wild-type phage 2972 on the wild-type *S.t*. DGCC7710 did not carry a mutation in *orf33*.

The wild-type Orf33 of phage 2972 consists of 157 amino acids (18.1 kDa, pI 6.6, GenBank accession number YP_238516.1). The *orf33* is a non-essential gene^37^ and has homology with a putative Mu Gam-like end protection protein found in the streptococcal phage Sfi19 (GenBank NP_049950.1). Orf33 shares 100% amino acid identity with homologs in 22 *S.t*. phages in GenBank, as well as in phages D1126 and D4752.

Using plaque assays, we confirmed that the replication of escape phages with a truncated Orf33 was no longer impaired by StCI-301-CAT (Fig. 6B). We also measured the impact of the mutated Orf33 on phage plaque size. The replication of the phage escape mutant led to smaller plaques (0.148 ± 0.024 mm^2^) on strain DGCC7710 as compared to the wild-type phage 2972 (0.367 ± 0.009 mm^2^). Moreover, lysates of phage 2972 consistently had a higher titer (9.95 ± 1.01 × 10^9^ pfu/ml) on strain DGCC7710 compared to the phage mutant (2.27 ± 2.21 × 10^8^ pfu/ml) on the same strain. While the frameshift mutation in *orf33* still leads to functional phages, it impacted phage fitness.

Next, we aimed to verify whether the mutation in Orf33 of phage 2972 had an impact on the induction of StCI-301-CAT in strain DGCC7710. Strain DGCC7710::StCI-301-CAT was infected by phage 2972 or phage 2972-Orf33-mut at a MOI of 10. A non-infected control was carried out in parallel. Samples were collected 10-, 20-, and 30-min post-infection. Genomic DNA was extracted from all the samples and Illumina sequenced. A gradual increase of the read depth at the StCI-301-CAT genomic positions was observed over time when the strain was infected with phage 2972 (Fig. 6C and Supp. Fig. 14). However, the StCI-301-CAT read depth did not increase over time in the non-infected samples and in the samples infected with phage 2972-Orf33-mut. The excision and replication of StCI-301-CAT were also quantified by qPCR (Supp. Fig. 15 and Table S5). The relative qPCR results showed that only the wild-type phage 2972 triggered StCI-301-CAT excision and replication. Indeed, there was a 17503-fold increase of excised StCIs and 23-fold more StCIs (StCI primase gene was used as a proxy of the total StCIs to quantify its replication) in the sample infected by phage 2972 at 30 min post-infection compared to the non-infected sample (T10) (Supp. Fig. 15D). In the non-infected condition, the excised form of StCI was rare as most of the StCIs were integrated in the bacterial chromosome at levels comparable to the reference host genes (Supp. Fig. 8). Therefore, when StCI levels from the infected samples by phage 2972 (in which all StCIs were excised and replicating) were normalized to those from the non-infected control, the resulting fold change appeared even higher than that observed for the primase. This reflected both the increase in copy number due to StCI replication and the near absence of excised StCIs in the baseline condition. Indeed, the raw mean cq values of the excised StCI and the StCI primase were similar in DGCC7710::StCI-301-CAT cells infected by phage 2972 at 20 and 30 min post-infection timepoints (Table S5). These cq values were also lower than those of the reference host genes, indicating that the levels of excised and total StCIs were higher than chromosomal DNA, consistent with StCI replication. These results indicate that phage 2972 with its full size *orf33* is essential for StCI-301-CAT excision and replication.

Then, we tested whether overexpression of the full-length Orf33 could promote loss of StCI-301-CAT. Following Orf33-mediated excision, failure of the StCI to reintegrate before the genome replication and cell division would generate at least one daughter cell lacking the element. Repeated events could cure the population of StCI-301-CAT^6,16^. The *orf33* and *orf33-mut* alleles were separately cloned into the plasmid pNZ123-ERY, a version of pNZ123 containing an erythromycin resistance gene that drives constitutive expression. To our knowledge, no tightly regulated inducible-expression system is available in *S. thermophilus*. The resulting plasmids were independently electroporated into *S.t*. DGCC7710::StCI-301-CAT. Bacterial DNA was extracted, sequenced, and reads mapped to the DGCC7710::StCI-301-CAT sequence. A marked reduction in read coverage at the StCI-301-CAT locus was observed only with the strain carrying pNZ123-ERY-orf33-wt (Supp. Figs. 16 and 17) but not in the strains with the empty vector pNZ123-ERY or pNZ123-ERY-Orf33-mut, confirming that Orf33 is required for StCI-301-CAT excision.

**Fig. 7:**
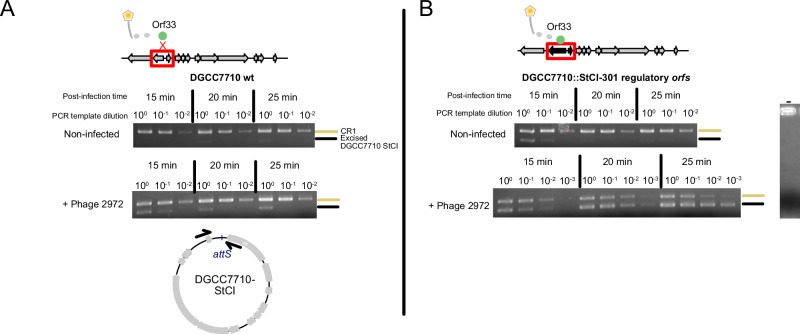
Wild-type phage 2972 can excise the StCI of *S. thermophilus* DGCC7710 only when the latter carries the regulatory *orfs* of the StCI from strain SMQ-301. The putative regulatory *orfs* (NCBI Reference Sequences WP_014608200.1 and WP_014608201.1) of the StCI from strain DGCC7710 were swapped with the ones from strain StCI-301 (NCBI Reference Sequences WP_011681043.1 and WP_041827685.1). The excision of the wild-type StCI of DGCC7710 (**A**) and of the engineered StCI of DGCC7710 containing regulatory *orfs* of StCI-301 (**B**) by the wild-type virulent phage 2972 was estimated by semi-quantitative PCR. Phage 2972 containing *orf33* (green circle) does not trigger the excision of the native DGCC7710-StCI. However, this phage can trigger the excision of the DGCC7710-StCI containing the regulatory region of the StCI-301. Strains *S.t*. DGCC7710 (wild-type, no-DNA control from the natural competence assay) and *S.t*. DGCC7710::StCI-301 regulatory *orfs* were infected at a MOI of ~5. Controls with non-infected *S.t*. strains were done in parallel. Samples from each bacterial culture were collected at 15, 20, and 25 min, post-infection. The experiment was conducted in biological duplicates. PCR reactions with primers targeting the excised StCI (DGCC7710_SP_circ_F5/SP_circ_R) and primers targeting the CR1 locus (Yc70/RDS7rev) to confirm strain ID were performed separately on tenfold serial dilutions of the samples. Phage 2972 was previously amplified twice on the wild-type strain DGCC7710.

We then hypothesized that Orf33 induces StCI-301 by interacting with its regulatory proteins, similar to the interaction between a phage-encoded protein and the SaPI-encoded Stl repressor^38^. To test this, we swapped the regulatory *orfs* of the DGCC7710 StCI (WP_014608200.1 and WP_014608201.1) with those from StCI-301 (WP_011681043.1 and WP_041827685.1). Semi-quantitative PCR showed that the wild-type phage 2972 (with intact *orf33*) could excise the modified DGCC7710 StCI carrying the StCI-301 regulatory genes, but not the native DGCC7710 StCI (Fig. 7).

Next, we assessed whether phage 2972 could induce not only excision, but also replication of the StCI in DGCC7710 carrying the StCI-301 regulatory *orfs*. The strain was infected with either phage 2972 or 2972-Orf33-mut at a MOI of 5 and samples were collected at 10, 20, and 30 min post-infection. Genomic DNA was extracted and analyzed by qPCR using primers targeting housekeeping genes (*gyrA* and *gyrB*)^39^, excised StCI, and the StCI primase gene. A time-dependent increase in excised StCI was observed only with phage 2972 infection (Supp. Fig. 18 and Tables S4 and S5), reaching a sixfold increase at 30 min post-infection compared to the non-infected sample (T0) (Supp. Fig. 18C). No increase in StCI excision was detected with phage 2972-Orf33-mut. Interestingly, the total StCI levels remained unchanged across all conditions, indicating that the chimeric DGCC7710-StCI did not replicate, even when excision increased. Even though there was a statistically higher StCI primase relative quantity in all the samples infected by phage 2972 and by phage 2972-Orf33-mut (T10) than in the non-infected control, it was inconsistent over time and less than twofold. Together, these results indicate that the StCI-301 regulatory *orfs* are required for excision, but are insufficient to support replication of this chimeric StCI. This may reflect incompatibility with DGCC7710-StCI replication machinery or disruption of replication elements during the genetic engineering process.

To verify if Orf33 alone is enough to excise the DGCC7710::StCI-301 regulatory *orfs*, we electroporated the pNZ123-ERY-Orf33-wt, pNZ123-ERY-Orf33-mut, and the empty plasmid into strains DGCC7710::StCI-301 regulatory *orfs* and DGCC7710. Then, we measured StCI excision by semi-quantitative PCR (Supp. Fig. 19). Our results demonstrated that Orf33 from phage 2972 is enough to excise the DGCC7710-StCI containing the StCI-301 regulatory *orfs*, but not the wild-type DGCC7710-StCI. As expected, the empty plasmid and pNZ123-ERY-Orf33-mut could not excise any StCI (Supp. Fig. 19). Thus, the StCI-301 regulatory *orfs* are essential for StCI excision by the phage-encoded Orf33. Overall, these results indicate that the Orf33 of phage 2972 can excise StCI-301, likely through an interaction with its regulatory *orfs*. Since the regulatory *orfs* of StCI-301 differ from those of the DGCC7710-StCI (Supp. Fig. 2), there may be an incompatibility between Orf33 from phage 2972 and the regulatory *orfs* of the DGCC7710-StCI that prevents excision.

**Fig. 8:**
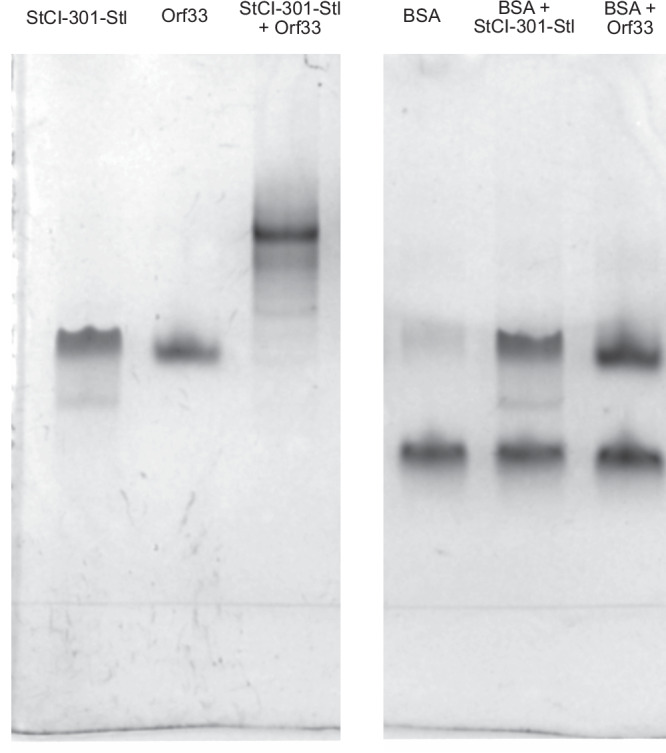
Orf33 of phage 2972 interacts with the Stl repressor (StCI-301-Stl) located in the regulatory region of StCI-301. Protein-protein native electrophoretic mobility shift assay (EMSA) with purified StCI-301-Stl, purified Orf33, and commercial bovine serum albumin (BSA). Proteins were loaded alone or in pairs, each at concentration of 3 µM, on 6% acrylamide gel. The gel was stained with Coomassie blue. Experiment was conducted in duplicate. BSA was considered as a monomer while StCI-301-Stl and Orf33 are dimers (see Supp. Fig. 20).

We then checked if Orf33 is directly interacting with its Stl repressor located in the regulatory *orfs* region^38^. We purified both proteins and conducted an electrophoretic mobility shift assay (EMSA). A distinct shift in migration was observed when the purified Orf33 and StCI-301-Stl were combined (Fig. 8), indicating a direct interaction between the two proteins. No interaction with bovine serum albumin was detected for either Orf33 or StCI-301-Stl. We also found, through size exclusion chromatography, that StCI-301-Stl and Orf33 both form dimers in solution (Supp. Fig. 20).

These results show that the Orf33 of phage 2972 binds to the Stl-like protein of StCI-301, likely triggering StCI-301 excision. We also showed that the phage 2972-Orf33-mut does not induce the StCI-301 (Fig. 6 and Supp. Fig. 15), and it can escape the StCI-301 phage-resistance mechanism (Fig. 6). Therefore, the phage-resistant mechanism activation seems to depend on the StCI induction.

### StCI mobility during phage infection

During virulent phage infection, the induction of a StCI increases its copy number within the cell (Fig. 6 and Supp. Fig. 15). These StCI can be released into the local environment after phage-driven cell lysis, which would favor StCI mobility to another cell through natural competence. To explore this possibility, we amplified phages 2972 and 2972-Orf33-mut on strain *S.t*. DGCC7710::StCI-301-CAT. A non-phage-infected control was carried out in parallel. The phage lysates and the supernatant of the non-infected control were filtered and used to transform strain *S.t*. UY03-$\triangle$StCI-$\triangle$RSR with and without the competence-inducing peptide ComS^23^. This recipient *S.t*. strain was used because phage 2972 cannot plaque and adsorb on *S.t*. UY03-$\triangle$StCI-$\triangle$RSR, which mitigates transduction of StCI−301-CAT and favors natural competence as the driver of StCI mobilization (Supp. Fig. 21). The titer of phage 2972-Orf33-mut was lower than 2972; thus, we normalized the amount of phage 2972-Orf33-mut to the amount of 2972. We also added a condition where we transformed 30 μl of the undiluted phage 2972-Orf33-mut lysate. Colonies were selected on LM17-CM5 plates. Even though phage 2972 cannot mobilize DNA into strain UY03-$\triangle$StCI-$\triangle$RSR through transduction, we added an extra control containing phages and bacteria, but with no ComS peptide.

**Fig. 9:**
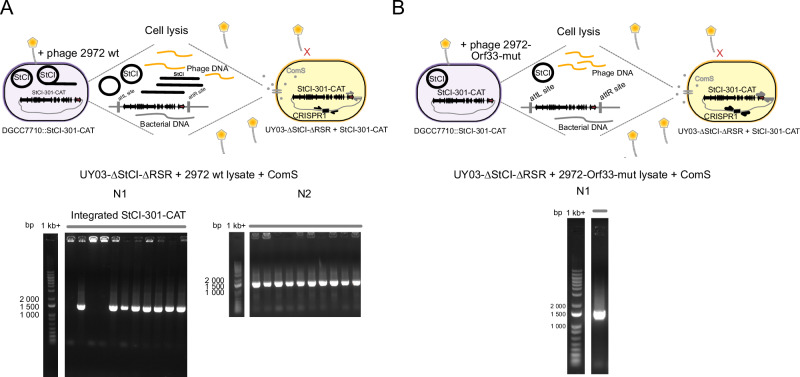
StCI-301-CAT can be mobilized from a phage lysate into a new host through natural competence. Wild-type phage 2972 (**A**) and phage mutant 2972-Orf33-mut (**B**) were amplified on *S.t*. DGCC7710::StCI-301-CAT. The lysates were filtered and transformed by natural competence into *S.t*. UY03-∆StCI-∆RSR with and without the natural competence-inducing peptide ComS^23^. Phage 2972 does not infect UY03-∆StCI-∆RSR (Supp. Fig. 21). For the phage-containing conditions, the recovery media were diluted 1/10 before colony selection on LM17 + CM5 plates. Separate no-DNA and no-bacteria controls were performed in parallel. An average of 23.3 ± 10.3 colonies (dilution 10^−1^) was obtained with the condition containing UY03-∆StCI-∆RSR + phage 2972 lysate + ComS. The presence of StCI-301-CAT was verified on 20 colonies by PCR across two biological replicates. Strain ID was confirmed by PCR amplification of the CRISPR1 locus (Source data file). We obtained 0.5 ± 0.7 colonies (dilution 10^−1^) in the condition containing UY03-∆StCI-∆RSR + phage 2972-Orf33-mut lysate + ComS. No StCI+ colony was obtained in the no-DNA, no-bacteria, and no ComS peptide conditions. PCR products were migrated on a 0.8% agarose gel. The PCR negative controls are available in the Source data file. N1 and N2 represent two biological assays of the StCI-301-CAT mobilization experiment. CAT Chloramphenicol acetyltransferase, CM5 Chloramphenicol 5 µg/ml, RSR pNZ123 plasmid containing the crRNA, CRISPR1 Clustered regularly interspaced short palindromic repeats 1 locus in *S. thermophilus*.

In the presence of ComS, we observed more chloramphenicol-resistant colonies (23.3 ± 10.3) of UY03-$\triangle$StCI-$\triangle$RSR when using wild-type phage 2972 lysate compared to when using the normalized phage 2972-Orf33-mut lysate (0.5 ± 0.7 colony). Ten colonies obtained with the phage 2972 lysate and the only colony obtained from the phage 2972-Orf33-mut lysate were screened by PCR and the PCR products were Sanger-sequenced to confirm the strain ID and to detect the integrated StCI-301-CAT (Fig. 9 and Supp. Fig. 22). StCI+ colonies were absent in the other tested conditions.

The PCR product from the colony obtained with phage 2972-Orf33-mut lysate was sequenced to cover the junction between the StCI 3′-end and its flanking gene. Sequence analysis revealed that this colony had a mutation in the StCI flanking region that differed from the wild-type UY03-$\triangle$StCI-$\triangle$RSR strain (Supp. Fig. 22). Interestingly, this mutated sequence was identical to the StCI flanking region found in DGCC7710::StCI-301-CAT. These results suggest that StCI-301-CAT integration in UY03-$\triangle$StCI-$\triangle$RSR genome likely occurred via homologous recombination with free genomic DNA of DGCC7710::StCI-301-CAT in the phage lysate rather than a StCI-specific integration. This mutated flanking region was not observed in the StCI+ colonies obtained with phage 2972 lysate, suggesting that all the integration events were StCI-specific.

Taken altogether, this experiment is a proof-of-principle that a StCI-inducing virulent phage enhances StCI mobility through natural competence and facilitates its specific integration in a new host.

We showed that StCI-301-CAT concentration increases following induction by the *pac*-type phage 2972, generating sufficient copies to be released into the environment via phage-mediated lysis and taken up by another host through natural competence. We also identified two putative *cos* sites in StCI-301 (5′-CCACGACAAGGTG-3′ and 5′-CCACAACAAGGTG-3′), each with one mismatch to the wild-type phage DT1 cos site^40^ (5′-CCACCACAAGGTG-3′). Thus, StCI-301 may also be mobilized by transduction, although this remains to be tested.

In conclusion, we showed that StCIs can spontaneously excise from the bacterial genome and be mobilized between strains through natural transformation. We also identified CRISPR spacers targeting PSs in *S. thermophilus* and other *Streptococcus* sp., suggesting PS mobility between streptococcal species. CRISPR-Cas systems appear to contribute to defense against these MGE, as demonstrated by our ability to select cells without StCIs using CRISPR-Cas9. StCIs and related MGEs likely play a significant role in shaping phage-bacteria interactions.

Although spontaneous StCI excision would be expected to reduce their prevalence in *S. thermophilus*, especially if excised elements fail to reintegrate before cell division^6,16^, over two-thirds of *S.t*. strains still carry at least one StCI. In comparison, only 13% of *S.t*. strains harbor a full prophage^41^. We further showed that StCI-free strain can readily reacquire these elements via natural competence, which may compensate for rare loss events. These finding strongly suggested that StCIs confers a benefit to *S.thermophilus*.

We also found that exchanging StCIs can enhance phage resistance, enabling the use of virulent phages to select StCI-containing cells. A phage protein (Orf33) was identified as essential for StCI induction through direct interaction with the StCI-encoded regulator Stl. Truncation of this protein abolishes both induction and phage resistance. Lastly, we demonstrated that virulent phages strongly induce StCI excision and transfer, promoting their dissemination and chromosomal integration through natural competence. While further work is needed to define the precise mechanism of StCI-mediated phage resistance, our findings highlight a potential strategy for developing natural phage-resistant strains for industrial applications.

## Methods

### Biological and molecular biology materials

Bacterial strains, phages, plasmids, and primers are listed in Tables S6–S9.

### *S.t.* phage satellite identification

Using the complete assembly-level filter of GenBank, 87 *S. thermophilus* genomes were extracted (March 2023). To identify the StCIs among them, we aligned StCI-301 with the extracted *S.t*. genomes using the Discontiguous Megablast algorithm in Geneious software (version 11.1.5)^42^ with the following parameters: scoring 2–3, gap cost 5 2, max *E*-value 10, word size 11, template type coding, low complexity filter, template length 18, and max target seqs 1,000,000. The hits were then manually verified in the corresponding genome. A StCI was defined as a region that contained a gene coding for an integrase, located between repeat regions (*attL* and *attR* sites), ranging in size from 7 to 20-kb, contained regulatory and replicating modules, and did not contain genes coding for viral structural proteins. *S. thermophilus* strains with no StCI hits (27 out of 87, 31%) were also analyzed using Prophage Hunter^43^. Using PECAAN (version 20221109), we manually annotated the StCIs from *S.t*. strains DGCC688, DGCC8234, DMST, EPS, and CS20^44^. We compared the amino acid identity between StCIs using the complete CDS annotation in GenBank files and the manual annotations (PECAAN). To group the StCIs based on their protein homology, we used their nucleotide sequences as inputs in the VirClust program^45^. The proteins were grouped into clusters using the following criteria: an *E*-value < 1 × 10^−5^, a bitscore > 50, coverage > 0, and sequence identity > 10%. Bootstrap analysis was performed, and a clustering distance threshold of 0.4 was applied.

We also used SatelliteFinder to confirm the presence/absence of StCI in the genome of *S. thermophilus* strains and to determine their classification^26,27^ (Table S1). The VirClust program was used to identify StCI proteins from the nucleotide sequences and to generate a protein fasta file containing the protein sequences of StCIs, which was used as an input in SatelliteFinder^26,27,45^. SatelliteFinder was ran on a Galaxy server surface (V0.9). We tried all the models of satellite family (cf-PICI, PICI, P4, and PLE)^26,27,46^.

### CRISPR spacers targeting StCIs

The complete sequences of the 61 StCIs (Table S1) were aligned against the *CRISPR Spacer Database and Exploration Tool* (http://crispr.genome.ulaval.ca)^47^ using BLASTn 2.9.0, Python 3.7.6, pandas 1.0.1, NumPy 1.18.1, and SQLite3 3.31.1 softwares^48–51^. A maximum of two mismatches were allowed. BLASTn searches using the web version were conducted on each spacer to verify their genomic contexts. Spacers were considered to specifically target PSs if they match, with 100% coverage and 100% identity, a region that is only located in the phage satellite.

### Spontaneous excision of StCIs detected by PCR

*S. thermophilus* strains were grown overnight at 37 °C in LM17 medium. PCR reactions with the Phusion High-Fidelity DNA Polymerase (NEB) were carried out on overnight cultures following the manufacturer’s recommendations. The following pairs of primers were used: SP_circ_F/SP_circ_R with strains SMQ-301, LMD-9, UY03, and Abc2; Abi-F/SP_circ_R with strain DGCC7891; SP_circ_R/DGCC7710_circ_F with strain DGCC7710; DGCC688_SP_circ_F/DGCC688_SP_circ_R with strain DGCC688; and DGCC8234_circ_F/DGCC8234_circ_R with *S.t*. DGCC8234 and ATCC19258.

### Phage amplification

Scrapings of virulent phage lysates stocked in LM17 with 15% glycerol at −80 °C were inoculated in 10 ml of LM17 containing their respective host and 10 mM of CaCl_2_. Cultures were incubated at 42 °C until lysis. Lysate was centrifuged to remove bacterial debris and filtered (0.45 μm). A second phage propagation was carried out at 42 °C by adding 50 μl of the first amplification to a 10 ml culture of the host that reached an O.D._600_ between 0.1 and 0.2. After cell lysis, the lysate was centrifuged, filtrated, and stored at 4 °C until use.

### DNA extraction from *S. thermophilus* cells

Ten milliliters of a *S. thermophilus* overnight culture were centrifuged and the pellet resuspended in 1 mL of 0.85% NaCl. This latter step was not conducted for samples taken at different timepoints for the qPCR experiments. The solution was centrifuged, and the pellet was resuspended in 400 μl of lysis buffer (20 mM Tris-HCl, 0.2 M NaCl, pH 6) containing 0.02 mg of purified endolysin from streptococcal phage 2972 or D5842. Endolysins were purified by His-tag affinity chromatography as described previously^52^. For the StCI-301 induction assays with plasmids, 1 mM of EDTA was added to the lysis buffer. Cell-containing samples were incubated for 1 h at 37 °C. Then, 50 μl of 10% SDS were added and further incubated for 30 min at 60 °C. Next, 20 μl of proteinase K (20 mg/mL) were added and re-incubated for 20 min at 60 °C. Two 500 μl phenol-chloroform (vol 1:1) treatments were carried out on the samples. Then, 100 μl of 7.5 M ammonium acetate and 1 ml of EtOH 95% were added to the aqueous phase and the sample was centrifuged. The nucleic acid pellet was washed three times with EtOH 70%, and the dried pellet was resuspended in EB buffer (10 mM Tris-Cl, pH 8.5) overnight. The following day, the sample was treated with 1 μl of RNase (10 mg/ml) for 15 min at 37 °C.

### Construction of StCI-301-CAT and StCI-301-CAT-rev

To add a selection marker (chloramphenicol resistance gene *cat* from pNZ123^53^) to the StCI of *S.t*. SMQ-301 (StCI-301), DNA fragments (StCI-CAT and StCI-CAT_rev) were synthetized (BioBasics). StCI-CAT also contained a promoter (5′-GTAGCTTTATTTTTGTTTTTATGATTACAAAGTGATACACTAATTTTATAAA-3′) added upstream of the *cat*. This synthesized fragment was flanked by two homologous sequences (~ 1 kb each) from the StCI-301 that directed the insertion site. Then, StCI-CAT was amplified with the internal primers SP_F and SP_R by PCR (Table S9). The PCR product was purified (Qiagen) and transformed into SMQ-301 by natural competence (see below). Colonies were selected on LM17-CM5 plates. The presence of the modified StCI into *S. thermophilus* SMQ-301 was confirmed by PCR using primers CM5-circ-F and SMQ-R. In the constructed StCI-CAT_rev, the *cat* and the promoter were in the reverse orientation, and the colony-screening was done with primers CM5-rev-circ-F and SMQ-R.

### Transformation of SMQ-301 with StCIs by natural competence

The complete StCIs were amplified by PCR (Bio-Rad CFX96 Real-Time System) using the following conditions: 25 μl reaction contained 1 μg of extracted genomic DNA, 1 μl of Phusion High-Fidelity DNA Polymerase, 1 μl of dNTPs, 0.25 μl of AttL_F and SP_ampli_R2 primers (50 mM), and 0.25 μl MgCl_2_ (25 mM). The PCR reaction started with 10 min at 95 °C, and then each cycle had a first step of 10 s at 98 °C, a hybridization step at 62 °C for 30 s, and a 7–8 min elongation time at 72 °C. The PCR was run for 35 cycles and with a final elongation step of 10 min at 72 °C. We also PCR-amplified the StCI-301-CAT-rev from just after its *attL* site to its *attR* site using SP_301_ampli_F and SP_301_ampli_R primers. The PCR amplified StCI from strain SMQ-301 was purified (Qiagen PCR purification kit) and phosphorylated (Anza PNK kit, Invitrogen). Phosphorylation reactions were inactivated for 5 min at 80 °C. Phosphorylated samples were ligated (T4 DNA ligase kit, Invitrogen) overnight at 16 °C. DNA templates were transformed into competent *S. thermophilus* cells that were prepared as described previously^23^ with the following modifications: 6 μl of 100 μM ComS peptide were added to 300 μl of competent cells and the mixture was incubated for 4 h at 37 °C before being spread on LM17-CM5 agar (1%) plates. Plates were incubated for 24–48 h at 42 °C. Transformation controls with no DNA were carried out in parallel. The transformants were confirmed by various PCR and Sanger sequencing. The details associated with the incubation time, reagents, amount of DNA, and primers used are available in Table S10.

### StCIs excision and replication quantification during different growth phases

*S. thermophilus* strains SMQ-301, UY03, DGCC7710, and DGCC7710::StCI-301-CAT were grown in liquid LM17 at 42 °C and 10 ml samples were collected at OD_600_ 0.2, 0.4, 0.8, and after a 24 h incubation. Samples were centrifuged and pellets were flash-frozen. Experiments were conducted in biological triplicate. Genomic DNA was extracted from the pellets and the DNA was eluted in cell culture-grade water. A relative qPCR experiment was conducted on the samples to quantify the excision, replication, and integration of StCIs. The primer binding efficiency was verified using several dilutions of a pool containing the same concentration of representative samples. Primers used targeted the housekeeping gyrase genes *gyrA* (GyrA_qPCR_F1/GyrA_qPCR_R1) and *gyrB* (GyrB_qPCR_F1/GyrB_qPCR_R1), StCI primase (*pri*) gene (primase_qPCR_F2/Primase_qPCR_R2), excised StCI (SP_excised_qPCR_F1/SP_excised_qPCR_R1), integrated StCI (delta_SP_qPCR_F1/SP_excised_qPCR_R2), and both StCI flanking genes to detect an empty *attC* site (delta_SP_qPCR_F1/delta_SP_qPCR_R1) (Table S9). qPCR measurements were done in technical triplicate. Each reaction contained 10 μl of Power SYBR mix, 0.5 μl of each primer (10 μM), 5 μl of DNA template, and 4 μl of cell culture-grade water. qPCR experiments were run with the following program: 15 s at 95 °C, 60 s at 60 °C × 40 cycles + Melting curve. The analysis was conducted on the Bio-Rad CFX Maestro software. Data points with a standard deviation > 0.250 in their technical replicates were removed from the analysis. Empty *attC* site amplification was low in the sample pool and unstable at low concentrations. Thus, a separate standard curve was carried out for the corresponding primer pair using genomic DNA from strain DGCC7710-$\triangle$StCI-$\triangle$RSR and measurements were conducted in technical duplicate. Each qPCR primer pair had an amplification efficiency between 90.9 and 95.1% and a *R*^2^ value between 0.998 and 1. We prepared qPCR reactions using ≈ 50 ng (dosed by spectrophotometry) of DNA from each sample. qPCR measurements were done in technical triplicate and using the same qPCR program as described above. Biological assays were run on separate plates, thus the variation between each assay included the technical and biological variability. The qPCR data calculations were conducted as previously described^35^, using the $\triangle \triangle$Cq method, but to calculate the relative quantity (RQ) for each target, we replace the “2” value from this formula: RQ = 2^delta Cq^, by the experimental primer amplification efficiency (E) value. All the data points used had a standard deviation <0.250. The relative quantity of each target was normalized to *gyrA* and *gyrB* and to the sample collected at an OD_600_ of 0.2. One sample (SMQ-301 OD_600_ of 0.8 from one assay) had a cq value slightly beyond the standard curve range (less than 0.5 cq) for one target (excised StCI (*R*^2^ = 0.999, *E* = 92.2%). The same efficiency was applied in the relative quantity calculations for this sample for the excised StCI target, as the deviation from the standard curve was minimal.

To compare the StCI excision/replication at the strain level, we used the qPCR data from the experiment described above, but only the samples incubated overnight from each strain and the relevant controls were included in the analysis. Since some targets (only for the DGCC7710 and DGCC7710::StCI-301-CAT samples) were run on separate plates, plate-to-plate variation were addressed by including inter-plate calibrators (one technical measurement for both housekeeping genes *gyrA* and *gyrB*) using the same genomic DNA across all plates. The mean cq value of both inter-plate calibrators on each plate was used to adjust cq values across two plates from the same biological assay. Housekeeping gene primer pairs (targeting gyrA and gyrB) were included for all the samples on each plate for normalization. The mean cq values of both inter-plate calibrators, in each plate, had a standard deviation (SD) < 0.25, except for one plate in one biological assay (SD = 0.31, DGCC7710 wt). After applying the inter-plate correction, the inter-plate variation was minimal (between 0.03 and 0.32 cq) and did not affect our biological conclusions.

Then, we applied the ∆Cq method as described previously^36^. The excised StCI, integrated StCI, empty *attC* site, and StCI *pri* cq values were normalized against the geometric mean of the *gyrA* and *gyrB* cq values ($\triangle$Cq method) using the RQ = E^−delta Cq^ equation^36^. For each target, we used the geometric mean of their amplification efficiency with the *gyrA* and *gyrB* primer amplification efficiencies, as the *E* value, since all the amplification efficiencies had less than 5% variation^54^. We used the geometric mean of the relative quantity in each assay as the normalized fold quantity. One-way ANOVA and Tukey tests were conducted on the log2-transformed normalized quantities when the ANOVA assumptions were met. When the data did not have a normal distribution and/or a homogeneous variance, a non-parametric Kruskal–Wallis test was conducted. The *p*-value threshold was set at 0.05. Statistical analyses were done in the R software v4.3.2^55^.

### Phage spot test assays and plaque size analysis

Phage spot test assays were done as detailed previously^21^ with the following modifications. Phage lysates were diluted tenfold in buffer (50 mM Tris-HCl, pH 7.5, 100 mM NaCl, 8 mM MgSO_4_). Next, 500 μl of an overnight host culture were mixed in molten soft agar (0.75%) (M17 Oxoid + 0.5% lactose or 0.5× M17 Nutribact + 0.5% lactose + 0.5% agarose + 0.5% glycine + 0.1% reconstituted skimmed milk) containing 10 mM CaCl_2_ at 55 °C and then poured on LM17 plates (Oxoid or 0.5× Nutribact) containing 1% agar and 10 mM CaCl_2_. Five μl or 10 μl of each phage dilution were dropped on the top layer. Plates were incubated overnight at 42 °C and plaques were recorded.

Phage plaque size analysis was done as detailed previously with the following modifications^52^. A sample of 500 μl of an overnight bacterial culture were mixed in molten soft agar (0.75%) (0.5× M17 Nutribact, 0.5% lactose, 0.5% agarose, 0.5% glycine, 10 mM CaCl_2_, 0.1% reconstituted skimmed milk) at 55 °C and then poured with 100 μl of phage dilution on LM17 (0.5× Nutribact) plates containing 1% agar and 10 mM CaCl_2_. These phage plaque assays were done in biological triplicate.

### Isolation of phage escape mutants

*S.t*. DGCC7710::StCI-301-CAT was infected in M17 broth with phage 2972 for 8 h and few phage plaques could be isolated and purified three times on the same strain. This phage isolation procedure was repeated with the phage-sensitive wild-type strain DGCC7710, but the incubation period was shorter. Phage DNA was extracted from the lysates as follows. One ml of phage lysate was mixed with RNase (10 mg/ml), DNase (1 mg/ml), and 10 mM MgSO_4,_ and incubated for 30 min at 37 °C. Then, 100 µl of pre-warmed SDS mix (0.25 M EDTA, 0.5 M Tris-HCl pH 9.0, 2.5% SDS) were added to the lysate and the mixture incubated for 30 min at 65 °C. After, 125 µl of cold KOAc 8 M were added and incubated for 30 min on ice. Samples were centrifuged for 10 min and the supernatant was treated with the same volume of a phenol-chloroform mixture (1:1). DNA was precipitated by adding a 0.7 volume of cold isopropanol and centrifuged for 10 min. The pellet was washed three times in EtOH 70%, dried, and resuspended overnight in 20 µl of EB buffer at 4 °C. Phage DNAs were sequenced (MiSeq, Illumina) as described in ref. ^56^.

### StCI-301 transformants were selected using phage 2972

Plasmid pNZ123 carrying the gene encoding for the anti-CRISPR protein AcrIIA5 (pNZ123-AcrIIA5) was purified from *Escherichia coli* DH5a^22^ using a Qiaprep Spin Miniprep kit and electroporated into *S.t*. DGCC7710-ΔStCI-ΔRSR^21^. Wild-type StCI-301 DNA was transformed by natural competence into *S.t*. DGCC7710-ΔStCI-ΔRSR containing pNZ123-AcrIIA5. A transformation control with no added DNA was also carried out. The recovered culture was transferred into 10 ml of chloramphenicol-containing (5 µg/ml) LM17 medium and incubated overnight at 37 °C. Then, 300 µl of each overnight culture was added to 3 ml of molten soft agar (0.75%) with 100 µl of phage 2972 lysate. Controls containing 300 µl of diluted (10^−5^) bacteria with 100 µl of LM17 in soft agar were carried out for each condition. Soft agar mixtures were spread on LM17-CM5 plates.

### Construction of pNZ123-ERY

The erythromycin resistance gene from plasmid pTRKH2^57^ was amplified using primers Ery_pTRKH2_F and Ery_pTRKH2_R. pNZ123 was amplified using primers pNZ_Ery_F and pNZ_Ery_R to exclude the *cat* gene. Both PCR products were purified (Qiagen PCR purification kit), assembled (Gibson method^58^), and electroporated into *L. lactis* MG1363^59^. Colonies were selected on GM17 plates containing 5 µg/ml of erythromycin and were screened by PCR using primers Ery_pTRKH2_F and pNZins_R. pNZ123-ERY. PCR products were extracted, purified, and confirmed by sequencing.

### Cloning of pNZ123-ERY-Orf33-wt and pNZ123-ERY-Orf33-mut

Primers were designed to clone the *orf33* of phage 2972 into pNZ123-ERY (Table S9). pNZ123-ERY was digested with XbaI and PCR-amplified using primers pNZ_XbaI_F and pNZ_XbaI_R2. PCR products were purified using a Qiagen PCR purification kit and the linear pNZ123-ERY was assembled^58^ with PCR products of *orf33-wt* or *orf33-mut*. Gibson assembly products were transformed into commercial *E. coli* DH5a competent cells following the manufacturer’s recommendations (NEB). Colonies were selected on BHI plates containing erythromycin (150 µg/ml) and screened by PCR using primers pNZins_F and pNZins_R. The two plasmids were extracted (Qiaprep Spin Miniprep) and electroporated, along with empty pNZ123-ERY, into *S.t*. DGCC7710::StCI-301-CAT. Colonies were selected on LM17 plates containing erythromycin (5 µg/ml) and confirmed by PCR using primers pNZins_F and pNZins_R and sequencing.

### Cloning of CRISPR spacers

A spacer targeting the gene coding for the integrase (*int*) of the StCI present in *S. thermophilus* SMQ-301 was cloned following a modified protocol^37^. Briefly, XbaI-R_Sint_R-for and XbaI-R_Sint_R-rev oligos were annealed to form the following template: XbaI restriction site - SMQ-301 CRISPR1 repeat—spacer targeting the StCI-301 *int*—SMQ-301 CRISPR1 repeat - XbaI restriction site. The annealed oligos and 1 µg of pNZ123 and pNZ123-ERY were digested with XbaI (NEB). The digested oligos were then phosphorylated with an Anza PNK kit. The digested pNZ123 was dephosphorylated using the Antarctic phosphatase kit (NEB). Oligos and linear pNZ123 were purified using a Qiaquick kit and ligated using a T4 DNA ligase. The ligation product was transformed in chemically competent *E. coli* DH5a cells. Colonies were selected on chloramphenicol-containing (20 µg/ml) LB plates and screened by PCR with primers pNZins_F and pNZins_R. PCR products were Sanger sequenced. Plasmids were extracted (Qiaprep Spin Miniprep) and electroporated into *S.t*. strains SMQ-301, DGCC7710, and UY03. Colonies were selected on LM17-CM5 plates. The absence of the StCI was confirmed by PCR using primers SMQ_F and SP_circ_R as well as SMQ_F and SMQ_R. The same crRNA was also cloned in the pNZ123-ERY as described above, but the colonies were selected on erythromycin-containing (150 µg/ml) plates. This plasmid and the empty vector were electroporated into *S.t* DGCC7710::StCI-301-CAT. The colonies were selected on LM17 plates containing erythromycin (5 µg/ml).

As the Cas9 of *S. thermophilus* DGCC7891 uses a different PAM motif (5′- NNACAAW-3′) than the one of SMQ-301 (5′-NNAGAA-3′), we selected a different spacer (5′-AGACAGCAAAAAAAAGCCACTGATTATCAG-3′) and the oligos XbaI-R_SNC_R_DGCC7891-F and XbaI-R_SNC_R_DGCC7891-R were annealed instead. The rest of the cloning process was the same as above, with the exception that the new spacer containing plasmid was transformed into *S.t*. DGCC7891 by natural competence.

### Plasmid loss assay

To remove the spacer-containing plasmid in *S. thermophilus* SMQ-301-pNZ123-RSR, DGCC7710-pNZ123-RSR, UY03-pNZ123-RSR, and DGCC7891-pNZ123-RSR-NC, each strain went through a series of overnight growth at 37 °C in liquid LM17 without antibiotic and at an inoculation rate of 1%^56^. After 5–10 passages, cultures were spread on LM17 plates, and colonies were streaked on LM17 plates with and without chloramphenicol (5 µg/ml). Colonies that did not grow on the CM5 plates were considered to have lost the plasmid, which was confirmed by PCR using primers pNZins_F and pNZins_R^56^.

### StCI-301-CAT induction using phages

Phages 2972 and 2972-Orf33-mut were amplified on *S. thermophilus* DGCC7710 as described previously, using 10 ml of filtered LM17 medium (0.5× M17 Nutribact). Then, we re-amplified the phages in 1-L of filtered LM17 and concentrated the phages through a CsCl gradient^60^. A two-hour DNase and RNase (1 μg/ml) treatment at 37 °C, with 10 mM of MgSO_4_ was conducted prior to the addition of 10% PEG 8000 and only one ultracentrifugation was carried out using a discontinuous CsCl gradient. Then, we infected a liquid culture of strain DGCC7710::StCI-301-CAT (OD_600_ of 0.5) in filtered LM17, containing 10 mM of CaCl_2_, with either phage 2972 or phage 2972-Orf33-mut at a MOI of 10. A non-infected control was carried out in parallel, and all cultures were incubated at 42 °C. Five ml samples were collected at 10-, 20-, and 30-min post-infection and flash-frozen. The thawed samples were centrifuged at 7197 × *g* during 5 min at 4 °C and bacterial DNAs were extracted from the pellets and sequenced (Illumina, SeqCenter, Pittsburgh, USA). This experiment was conducted in biological duplicate. Low-quality reads and Illumina adapters were removed using the FastQC program^61^ and Trimmomatic software package version 0.39^62^. We added the StCI-301-CAT sequence to the *S.t*. DGCC7710 sequence (NZ_CP025216) and mapped paired-end reads against this sequence using BWA aligner and SAMtools^63,64^. Read depth at each genomic position from the reference sequence was extracted using SAMtools^64^. The bioinformatic pipeline was done on Compute Canada’s server. Data analysis was done in the R environment^55^. The ggplot2, reshape, and tidyverse packages were used to create the graphs^65–67^.

A relative qPCR experiment was conducted on the sample to quantify the excision and replication of the StCI. The primer binding efficiency was verified using several dilutions of a pool containing the same amount of genomic DNA extracted from a culture of DGCC7710::StCI-301-CAT infected by phage 2972 (30 min post-infection with a MOI $\approx$ 10) and genomic DNA from a representative experimental sample of the DGCC7710::StCI-301 regulatory *orfs* infected by phage 2972 (30 min post-infection). We used primers targeting bacterial *gyrA* and *gyrB*^39^, the StCI *pri* gene, and the excised StCI (SP_excised_qPCR_F1/SP_excised_qPCR_R1) (Table S9). qPCR measurements were done in triplicate. Each reaction contained 10 µl of Power SYBR mix, 0.5 µl of each primer (10 µM), 5 ul of DNA template, and 4 µl of cell culture-grade water. Biological assays were conducted on separate plates and each plate contained all the targets. The relative quantity of each target was normalized to *gyrA* and *gyrB*, in each strain, using the ∆Cq method^36^ (Supp. Fig. 15B, C). We also conducted a second analysis where the targets were normalized against housekeeping genes and to the non-treated sample, in each biological assay^35^ (Supp. Fig. 15D). No inter-plate calibrators were conducted, thus the variation between each assay included the technical and biological variability. qPCR experiments were run with the following program: 15 s at 95 °C, 60 s at 60 °C × 40 cycles + Melting curve. The analysis was conducted on the Bio-Rad CFX Maestro software and data points that had a standard deviation > 0.250 in their technical triplicates were removed from the analysis. All the primer pairs had an amplification efficiency between 90 and 92.7% and a *R*^2^ value between 0.999 and 1. Then, we prepared qPCR reactions using $\approx \,$5 ng of DNA of each sample. Measurements were done in technical triplicate, and we used the same Bio-Rad program as described above. After removing some technical replicates, all the data points had a standard deviation <0.250. The qPCR data calculations were conducted as previously described^35^, using the $\triangle \triangle$Cq method, but to calculate the relative quantity (RQ), we replaced the “2” value from this formula: RQ = 2^deltaCq^, by the experimental primer efficiency value. The relative quantity of each target was normalized to *gyrA* and *gyrB* quantities and to the non-infected condition collected 10 min after the infection of the other samples (T10).

### StCI-301-CAT induction using plasmids

*S. thermophilus* strains DGCC7710::StCI-301-CAT + pNZ123-ERY, DGCC7710::StCI-301-CAT + pNZ123-Orf33-mut, and DGCC7710::StCI-301-CAT + pNZ123-Orf33-wt were grown to an OD_600_ of 0.5 with erythromycin (5 µg/ml), centrifuged at 4 °C, and pellets were flash-frozen. Bacterial DNAs were extracted and sequenced (Illumina, SeqCenter). The experiment was conducted in biological triplicate. We did the same bioinformatic analysis as described in the above section.

### Semi-quantitative detection of StCI induction by virulent phages

To determine whether the excision and replication of StCI-301-CAT occurred during virulent phage infection, we used an approach inspired by Köppen et al.^68^. Briefly, *S.t*. DGCC7710 and its engineered variant DGCC7710::StCI-301-CAT were grown until an OD_600_ of 0.2 and diluted in LM17 to reach a concentration of 4.4 × 10^6^ CFU/ml. Then, 10 mM of CaCl_2_ were added to the diluted culture. Two ml from the mixture were separated into two microtubes. In one microtube, DGCC7710::StCI-301-CAT was infected with virulent phages at a MOI of $\sim$5. Virulent phages 2972, D1126, and D4752 were amplified twice on DGCC7710 as described above. Phages were titered by spot test assay using 10 µl of tenfold phage dilutions with technical duplicate. With the other microtube, the same volume of LM17 was added as a non-infected control. Subsequently, 50 μl of each bacterial sample was collected at 15-, 20-, and 25-min post-infection. The time points were selected based on the latency period of phage 2972, which is estimated at 39 min^33^. Samples were kept in an 80% isopropanol bath at −80 °C and were subsequently heated for 15 min at 99 °C. PCR reactions with primers that target the excised StCI (CM5-circ_F/SP_circ_R) and the CRISPR1 locus (Yc70/ RDS7rev) were performed separately on tenfold dilutions of the samples. The same experiment was also performed using strain DGCC7710, but with primers Yc70/RDS7rev and DGCC7710_SP_circ_F5/ SP_circ_R. All semi-quantitative PCR experiments were done in biological duplicate.

### Swapping of the DGCC7710-StCI regulatory orfs with the StCI-301 ones

A DNA fragment (reg_SP_301) was synthetized (BioBasics), which contained the StCI-301 regulatory *orfs* flanked by two homologous sequences (~ 1 kb each) from the DGCC7710-StCI to direct the insertion site. The two homologous sequences excluded the DGCC7710-StCI regulatory *orfs*. Then, the StCI-301 regulatory *orfs* with the DGCC7710-StCI homologous sequences were amplified by PCR using the primers Ampli_g_block_reg_301_F and Ampli_g_block_reg_301_R. The amplified PCR product was purified (Qiagen PCR purification kit) and transformed by natural competence as described above, but the *S. thermophilus* DGCC7710 competent cells were grown in milk. A control with no added DNA was carried out in parallel. The recovery media were diluted and plated on LM17. Fifty colonies from the transformation assay were screened by PCR using primers Regulateur_PS-301_pet28a_F and Regulateur_PS-301_pet28a_R. The swap of the regulatory orfs was confirmed in the positive colonies using primers Regulateur_PS_DG_F and Regulateur_PS_DG_R. The StCI induction in the DGCC7710::StCI-301 regulatory *orfs* and the no-DNA control strains by phage 2972 was verified by semi-quantitative PCR as described above. The pNZ123-Orf33-wt, pNZ123-Orf33-mut, and the empty plasmid were electroporated in these strains to verify the StCI excision by semi-quantitative PCR, as described above.

### DGCC7710-StCI containing StCI-301 regulatory orfs induction using phages

We amplified phages 2972 and 2972-Orf33-mut on DGCC7710 and on DGCC7710::StCI-301-CAT, respectively, using filtered 0.5X LM17. Then, we re-amplified the phages in 1-liter volumes of filtered LM17 and concentrated the phages through a CsCl gradient, as described above. A liquid culture of *S.t*. DGCC7710::StCI-301 regulatory *orfs* (OD_600_ of 0.5) in LM17, containing 10 mM of CaCl_2_, was infected with phage 2972 or phage 2972-Orf33-mut at a MOI of ~5. A non-infected control was carried out in parallel, and all the cultures were incubated at 42 °C. One ml samples were collected 10-, 20-, and 30-min post-infection and were flash-frozen. A sample of the non-infected control was collected before the addition of phages (T0). Experiments were conducted in biological triplicate. Thawed samples were centrifuged at 17142 × *g* during 5 min at 4 °C and bacterial DNAs were extracted from the pellets. DNA pellets were eluted in cell culture grade water. qPCR experiments were conducted on some samples as described in the section above (StCI-301-CAT induction using phages). The relative quantity of each target was normalized to the bacterial genes *gyrA* and *gyrB*, in each strain, using the ∆Cq method^36^ (Supp. Fig. 18B). We also conducted a second analysis where the targets were normalized against *gyrA* and *gyrB* and to the non-infected condition collected just before the infection (T0)^35^ (Supp. Fig. 18C). One-way ANOVA and Tukey statistical tests were conducted on the log2-transformed normalized quantities from all the three assays. We set the *p*-value threshold at 0.05. Statistical analyses were done in the R software version 4.3.2^55^.

### StCI-301-CAT mobilization via natural competence using an inducing virulent phage

To verify if StCI induction could increase StCI mobility through natural competence following cell lysis by a virulent phage, we separately infected strain DGCC7710-StCI-301-CAT (OD_600_ of 0.2) with phage 2972 and phage 2972-Orf33-mut at a MOI of $\sim$5. Both cultures were incubated 2h15 at 42 °C. A non-infected control was carried out in parallel. Phages 2972 and 2972-Orf33-mut were amplified on DGCC7710 and on DGCC7710::StCI-301-CAT, respectively, prior to their purification on a CsCl gradient. The lysates and the non-infected control were centrifuged, and their supernatants filtered (0.2 μm). Then, 30 μl of each lysate were added to 300 μl of *S.t*. UY03-$\triangle$StCI-$\triangle$RSR with or without the natural competence-inducing peptide ComS^23^. Even if the titer of phage 2972-Orf33-mut was lower than 2972, we transformed the same amount of phage 2972-Orf33-mut as the amount of 2972. We also added a condition where we transformed 30 μl of the undiluted phage 2972-Orf33-mut lysate. One hundred μl of the recovery media were plated in technical duplicate on LM17-CM5 plates and incubated 48 h at 42 °C. No-added DNA and no-bacteria controls were carried-out in parallel. For the conditions containing phages, we plated the undiluted and the 10^−1^ dilution of the recovery media, since phage lysates contain endolysins and free phage DNA that could influence StCI+ colony number. To avoid the screening of false-positive colonies following the 48 h incubation at 42 °C, we streaked colonies on LM17-CM5 plates. Integration of the StCI-301-CAT in cells that grew on CM5 plates was verified by PCR using primers CM5-circ-F/Bras-R-R. Strain ID was confirmed by sequencing its CRISPR1 locus with primers Yc70/CR1-rev. The StCI− (false positives) colonies were removed from the total colony count. Natural competent *S.t*. UY03-$\triangle$StCI-$\triangle$RSR cells were grown in milk and prepared as described above. Assays were done in biological duplicate, including two independent amplifications for each phage and two independent non-infected DGCC7710::StCI-301-CAT supernatants.

To verify whether phage 2972 can infect *S. thermophilus* UY03-$\triangle$StCI-$\triangle$RSR, we performed a phage plaque assay as described above and phage adsorption assays using fluorescence microscopy^69^. For the latter, phage 2972 lysate (amplified on DGCC7710) was diluted to 10^9^ pfu/ml and stained with 0.1X SYBR Gold overnight at 4 °C. The stained lysate was treated with 1 µl of RNAse A (10 mg/ml) and 1 µl of DNAse (1 mg/ml) for 2 h at room temperature. *S.t*. strains UY03-$\triangle$StCI-$\triangle$RSR, *S.t*. DGCC7710 (positive control), and *L. lactis* MG1363 (negative control) were grown to an OD_600_ of 0.6. Then, 1 ml of each strain was centrifuged at 16,100 × *g* and resuspended twice in phage buffer with 10 mM CaCl_2_. Phage and bacteria were mixed to a MOI of 50 and incubated at room temperature for 1 min. The phage-bacteria mix was vortexed 15 s and 5 µl were put on an 1% agarose pad^70^ and covered with a coverslip. Imaging was done using a Revolve4 microscope (ECHO) in upright position with a 100X plan fluorite oil phase Ph3 objective (0.09 s, high gain) and with a FITC filter (2.5 s, low gain—EX: 470/40, EM: 525/50). Images were captured with a 5 MP CMOS Monochrome Camera and further treated with Fiji^71^. Adsorption assays were conducted in biological triplicate.

### Recombinant protein cloning, expression, and purification

Primers were designed to clone the gene coding for the Stl of StCI-301 into pET28a (Table S9). pET28a was digested with BamHI and PCR-amplified using primers pET28a_Fw/pET28a_rv. PCR products were purified (Qiagen), and the linear pET28a was assembled^58^ with the PCR product of StCI-301-Stl. Gibson assembly products were transformed into *E. coli* DH5a competent cells^42^. Colonies were selected on LB plates containing kanamycin (50 µg/ml) and screened by PCR using T7 and T7 terminator primers. The plasmid was extracted using a Qiaprep Spin Miniprep kit and transformed into *E. coli* BL21-CodonPlus (DE3)-RIPL (Agilent).

The *orf33* from phage 2972 was PCR-amplified with primers Orf33_pDEST14F and Orf33_pDEST14R (Table S9), and cloned into Gateway entry vector pDONR201 and then into Gateway expression vector pDEST14 following the manufacturer’s recommendations (Invitrogen). The LR reaction product containing the pDEST14-Orf33 was transformed in *E. coli* DH5a. Colonies were selected on LB plates containing ampicillin (100 µg/ml) and screened by PCR using primers pDEST14_F and pDEST14_R (Table S9). The plasmid was extracted (Qiaprep Spin Miniprep) and transformed into *E. coli* BL21 (DE3) pLysS.

Both proteins were expressed with uncleavable C-terminal 6xHis tags. For StCI-301-Stl, the *E. coli* culture was grown at 37 °C with shaking in Terrific Broth (Difco) supplemented with kanamycin (50 µg/ml) and chloramphenicol (34 µg/ml) until the OD_600_ reached 0.7. Protein expression was then induced with 1 mM isopropyl β-D-1-thiogalactopyranoside (IPTG) and the culture was incubated for 18 h at 18 °C with shaking. Cells were harvested by centrifugation at 5000 × *g* for 20 min and lysed using BugBuster protein extraction reagent (Millipore). Soluble and insoluble fractions were separated by centrifugation at 17,000 × *g* for 30 min. The insoluble pellet (containing StCI-301-Stl) was resuspended in buffer A (50 mM Tris, pH 8, 300 mM NaCl, 10 mM imidazole) supplemented with 8 M urea, and combined with 50% Ni-NTA agarose slurry (Qiagen), followed by agitation for 45 min at 7 °C. Suspension was transferred to a benchtop column and washed (10 column volume, CV) with buffer A + 8 M urea and with buffer A + 8 M urea supplemented with 50 mM imidazole. StCI-301-Stl was eluted with 6 CV of buffer A + 8 M urea supplemented with 250 mM imidazole. Elution fraction purity was confirmed by SDS-PAGE. Buffer exchange and on-column refolding of StCI-301-Stl was done by size exclusion chromatography (SEC) by injecting the purest elution fractions on a 16/600 Superdex 75 pg column (Cytiva) equilibrated in 20 mM Tris, pH 7.5, 150 mM NaCl, and 1% glycerol. The elution volume of StCI-301-Stl confirmed refolding of the repressor into a dimer (Supp. Fig. 23). Elution fractions from the SEC were pooled, concentrated on an Amicon 10 kDa filter (Millipore), flash frozen, and stored at −80 °C.

Orf33 from phage 2972 was expressed in the same conditions as StCI-301-Stl except for the antibiotics, which were replaced by 100 µg/mL ampicillin and 34 µg/mL chloramphenicol. Cells were harvested as for StCI-301-Stl. Pellet was resuspended in buffer A and lysed by sonication. The lysate was centrifuged at 17,000 × *g* for 30 min. Two protease inhibitor cocktail tabs (Roche) per liter of initial culture were added to the supernatant along with 90 U/mL Benzonase nuclease (Millipore) and 10 mM MgCl_2_. Suspension was incubated 18 h at 7 °C with agitation and loaded onto a Histrap HP 5 ml column (Cytiva). Column was washed with 10 CV of buffer A, 5 CV of 20 mM Tris pH 8 with 2 M NaCl (to remove nucleic acids), 5 CV of buffer A, and 10 CV of buffer A with 60 mM imidazole. Protein was eluted with buffer A and 255 mM imidazole. Purity was assessed by SDS-PAGE, purest fractions were pooled and concentrated using an Amicon 10 kDa filter before buffer exchange using SEC on a 16/600 superdex 75 pg column with buffer containing 20 mM Tris pH 8, 300 mM NaCl, and 1% glycerol. Elution fractions were pooled, concentrated on an Amicon 10 kDa filter, flash frozen, and stored at −80 °C.

### Protein-protein electrophoretic mobility shift assay (EMSA)

Three µM of (dimeric) purified StCI-301-Stl was combined with equimolar (dimeric) purified Orf33 from phage 2972 or (monomeric) BSA (Sigma-Aldrich) in 40 mM Tris pH 8, 0.1% Triton X-100, and 20 mM KCl. Binding reactions were left 20 min at 20 °C. Reactions were loaded on a 6% acrylamide gel, which was pre-electrophoresed for 20 min and migrated 90 min at 110 V on ice in pre-chilled 0.5X Tris Borate EDTA buffer. Gel was stained with Coomassie Blue.

### Reporting summary

Further information on research design is available in the Nature Portfolio Reporting Summary linked to this article.

## Supplementary information

Supplementary Information

Peer Review file

Reporting Summary

## Source data

Source Data

## Data Availability

GenBank accession numbers of previously published bacterial and phages genomes are mentioned in the text and are publicly available in the NCBI database. Sequencing datasets are available under the BioProject ID PRJNA1260446. Supplementary data generated in this study are provided in the Supplementary Information. The qPCR data and the uncropped gels of the figures are available in the Source Data file. Source data are provided with this paper.
