# Supplementary material for: Phage satellites induced by virulent phages are mobilized by natural competence leading to phage resistance in a new host
Source: Nat Commun. 2026 May 12;17:6528. doi: 10.1038/s41467-026-72928-1 (PMC13376191; doi:10.1038/s41467-026-72928-1)
Supplement: Supplementary file 1 — Supplementary Information [file 41467_2026_72928_MOESM1_ESM.pdf]

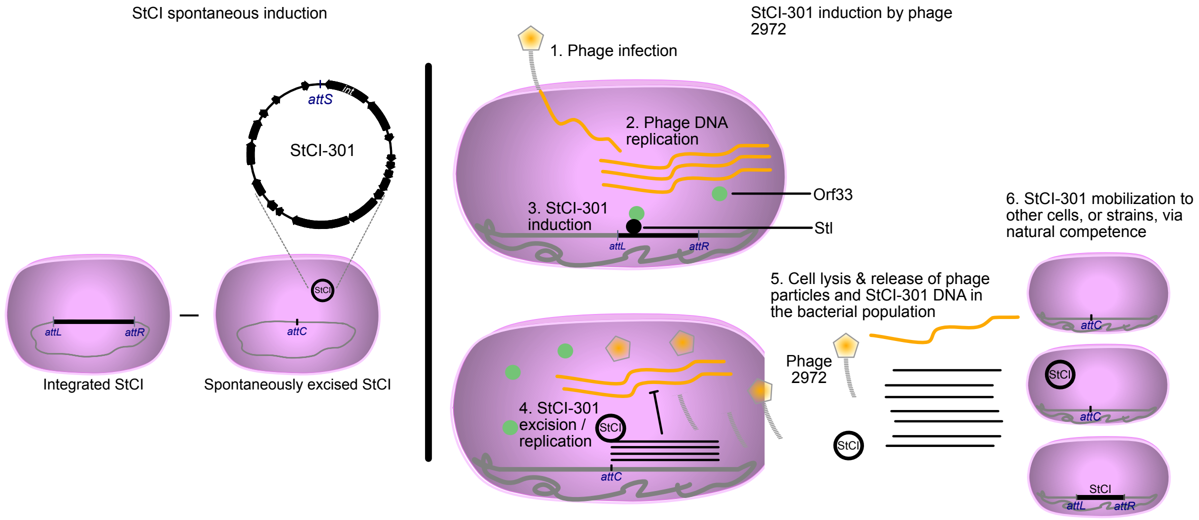

Supp. Figure 1: Schematic representation of StCI spontaneous excision, which occurs at a low frequency without phage infection. The StCI-301 element can be induced by the virulent phage 2972. Specifically, the Orf33 of phage 2972 binds to the Stl repressor of StCI-301, thereby triggering StCI induction. Once induced, StCI excises from the bacterial chromosome and replicates its DNA. StCI-301 then interferes with the lytic cycle of phage 2972 through an unknown mechanism. After bacterial cell lysis, both phage particles and StCI DNA are released into the environment, where StCI can be further mobilized into another bacterial host via natural competence.

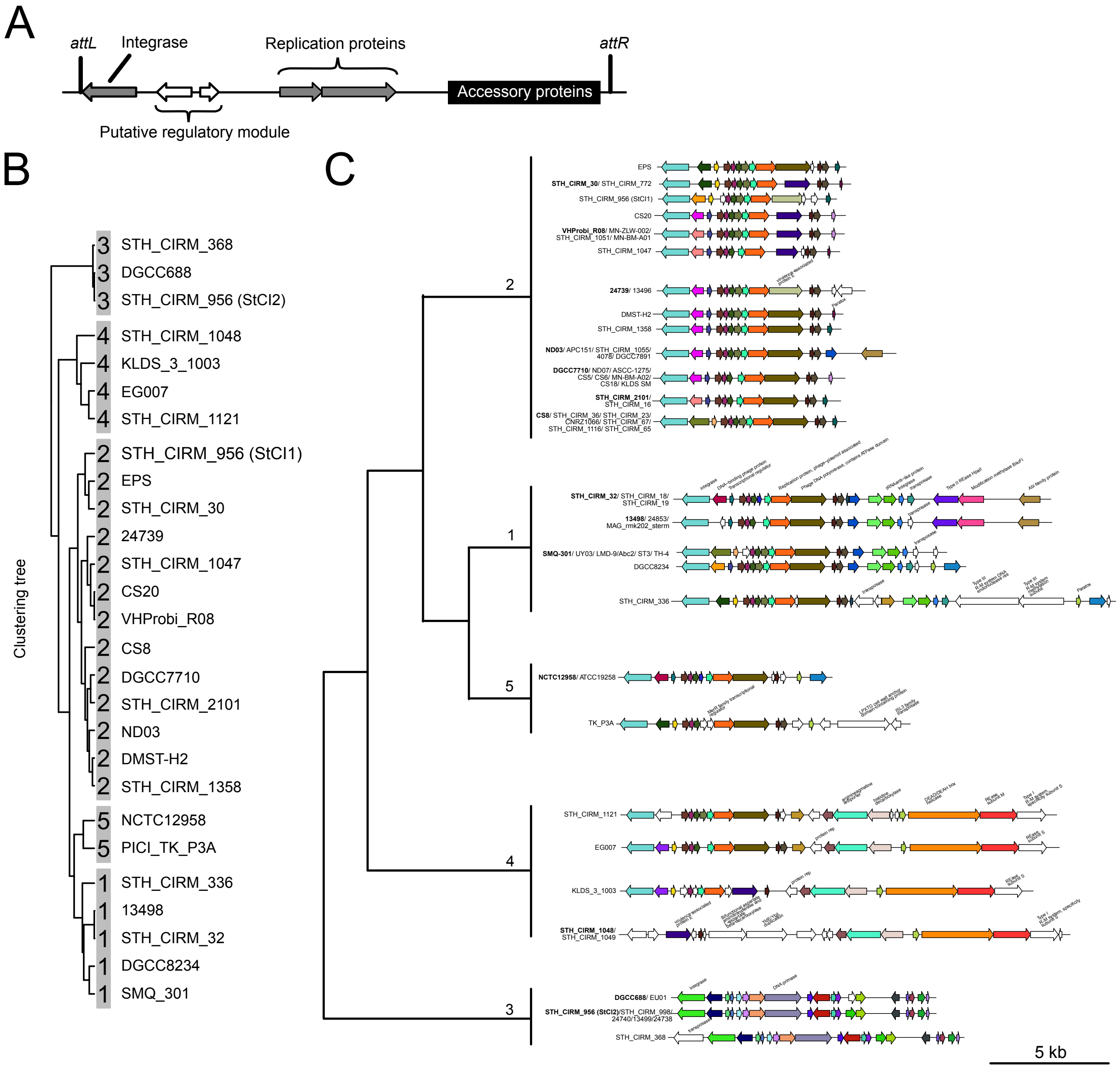

Supp. Figure 2: Genome alignment of StCIs. Panel A: Schematic representation of StCI structure. Panel B: StCIs were clustered based on their protein homology using VirClust[1]. Panel C: Simplified representation of the VirClust tree from panel B, including the StCI genome arrangements. The numbers 1 to 5 indicate the StCI cluster based on VirClust analysis. The *S.t.* strains are labeled to the left of each corresponding StCI genome. The representative host for each StCI grouping is indicated in bold. Open reading frames are represented by arrows and their annotations were extracted from GenBank files. Arrows with the same color indicate at least 80% of amino acid identity. R-M: Restriction-modification system, REase: Restriction endonuclease. Regulatory module or hypothetical proteins are not shown. StCI genomic maps were produced in R software (version 4.3.2) using *genoPlotR* package[2,3].

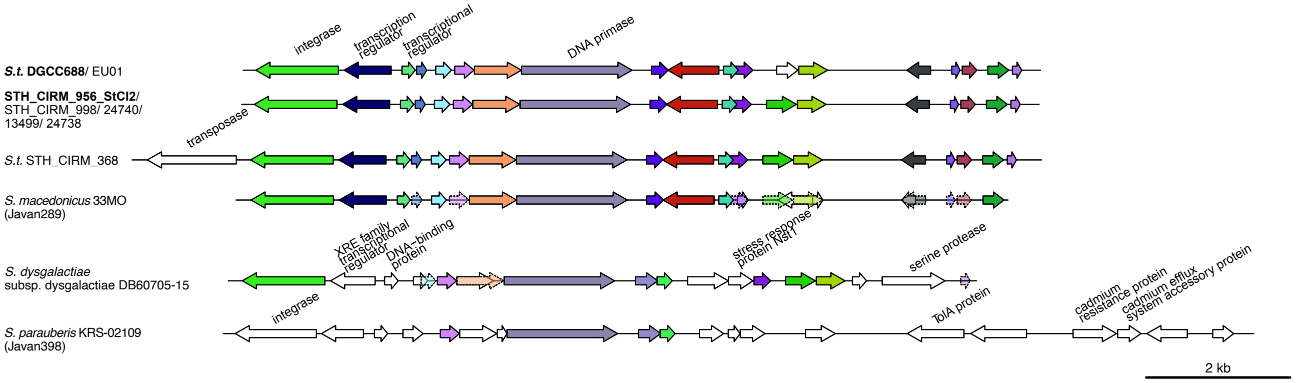

Supp. Figure 3: Genome alignment of StCIs from the cluster 3 (Supp. Figure 1) with probable satellite phages from strains *S. macedonicus* 33MO, *S. dysgalactiae* subsp. *dysgalactiae* DB60705-15, and *S. parauberis* KRS-02109. Open reading frames are represented by arrows. Arrows with the same color indicate at least 80% amino acid identity. Bacterial strain names are indicated to the left of each corresponding satellite phage genome. Annotations from GenBank files were kept. Seemingly missing *orfs* were manually added and are represented as arrows with dotted lines. Annotations from hypothetical proteins are not shown. Phage satellite genomic maps were produced in R software (version 4.3.2) using *genoPlotR* package[2,3].

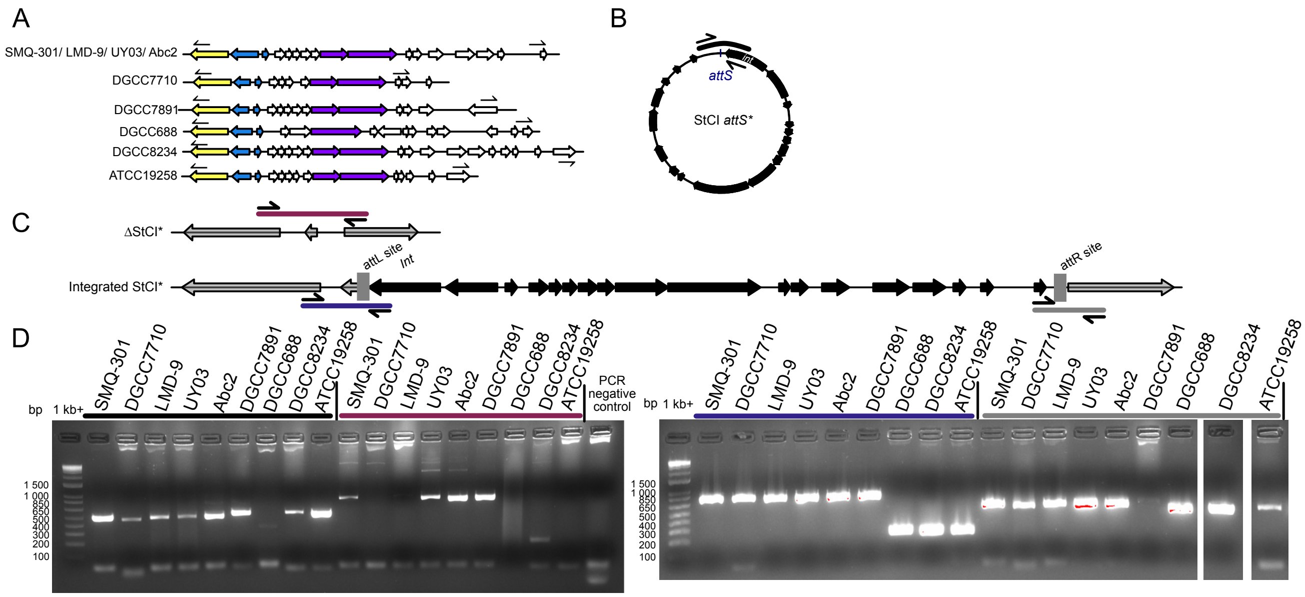

Supp. Figure 4: Detection of integrated and excised StCIs in nine *S.t.* strains. StCI can spontaneously excise from the bacterial chromosome without phage infection. Panel A: Genomic organization of StCIs in the nine *S.t.* strains. The excised StCIs were detected by PCR using primers (one side arrows) specific to StCI ends. Open reading frames are represented by arrows and their putative function are color-coded (yellow/integration, blue/regulatory, and purple/replication). Panel B: Schematic representation of StCI excision and circularization. Panel C: Schematic representation of primer locations in *S.t.* strains with and without StCI. *Primer sequence and location vary between strains (Supp. Table 9). PCR products are colored-coded thick lines. Panel D: PCR products migrated on a 2% agarose gel and PCR products were Sanger-sequenced. Name of the *S.t.* strain on top of each lane. Sequencing of PCR products revealed the *attS* site, leading to the exact StCI genomic positions. Primers used to amplify the specific StCI in each strain are in Supplementary Table S9. StCI linear genomic maps were produced in R software (version 4.3.2) using *genoPlotR* package[2,3].

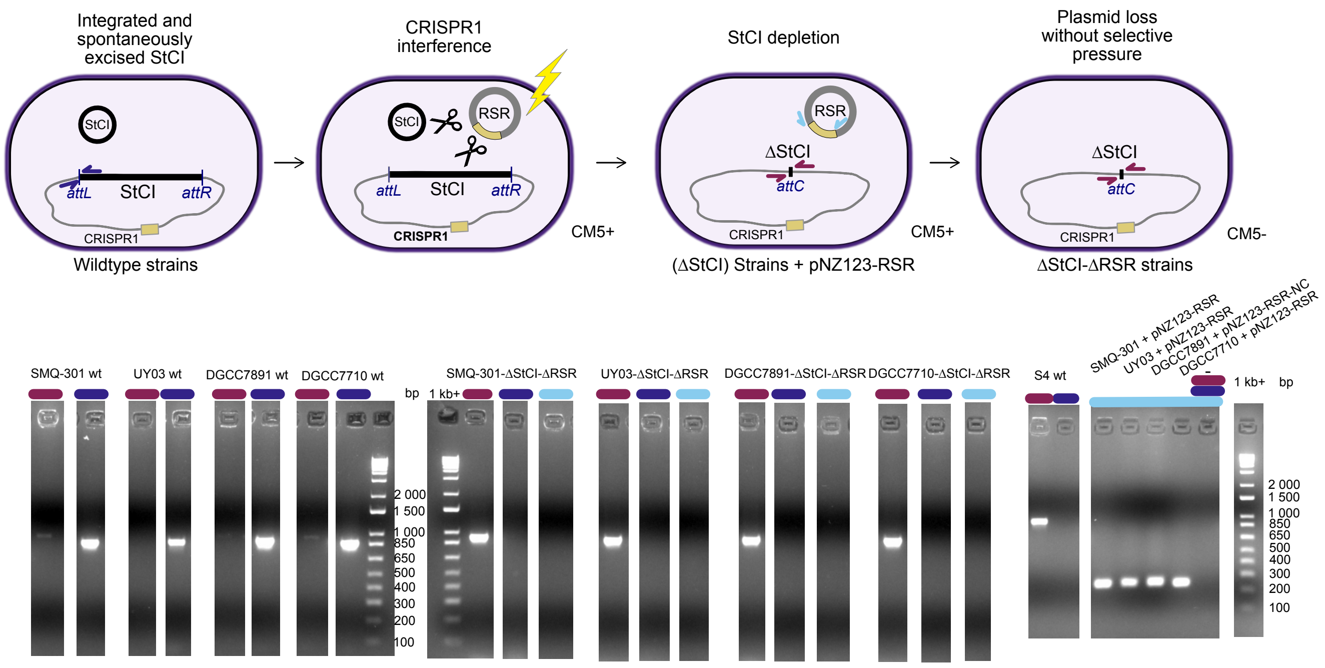

Supp. Figure 5: Four *S.t.* isolates without StCIs were selected using CRISPR-Cas9. The top panel presents the general methodology to generate a strain without a StCI. In the bottom panel, the presence/absence of StCIs was verified by PCR in ΔStCI strains, wild-type strains, and in S4, a wild-type *S.t.* strain naturally lacking a StCI. The presence/absence of the plasmid containing a crRNA was determined by PCR in the ΔStCI strains before and after serial growth in a medium without antibiotics. PCR products were migrated on a 2% agarose gel. Specific primers are color-coded in the figure, and their sequences are shown in Supplementary Table S9.

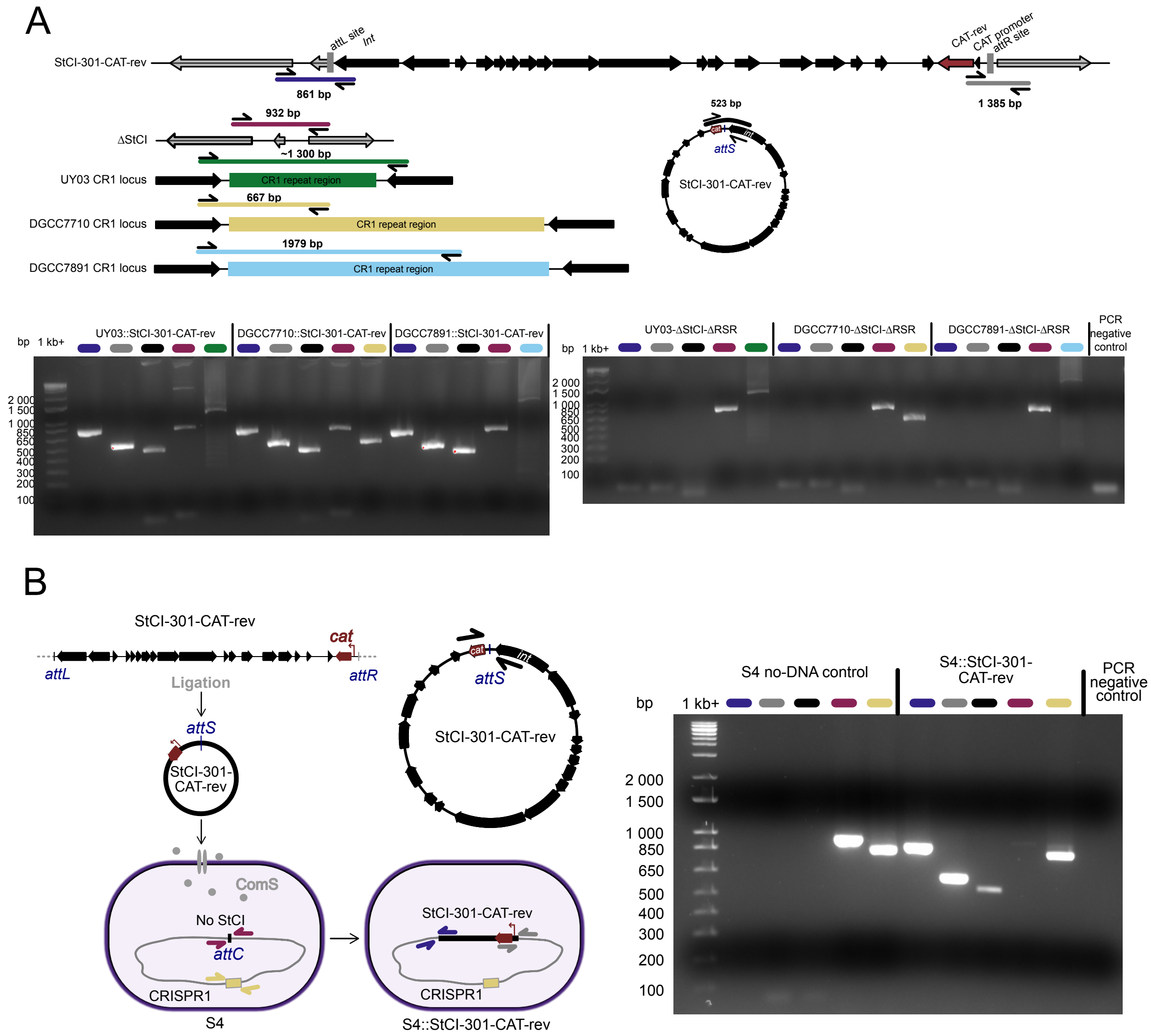

Supp. Figure 6: The StCI of *S.t.* SMQ-301, engineered to contain a chloramphenicol resistance gene in the reverse orientation (StCI-301-CAT-rev) of the flanking genes, was mobilized into three *S.t.* ΔStCI strains (Panel A) and into *S.t.* S4 (Panel B) using natural competence. The presence/absence of the new integrated StCI was confirmed by PCR. The strain ID was confirmed by the spacer content of its CRISPR1 locus. PCR products migrated on a 2% agarose gel. Predicted PCR products and specific primers used are color-coded. The StCI linear genomic map was produced in R software (version 4.3.2) using *genoPlotR* package[2,3].

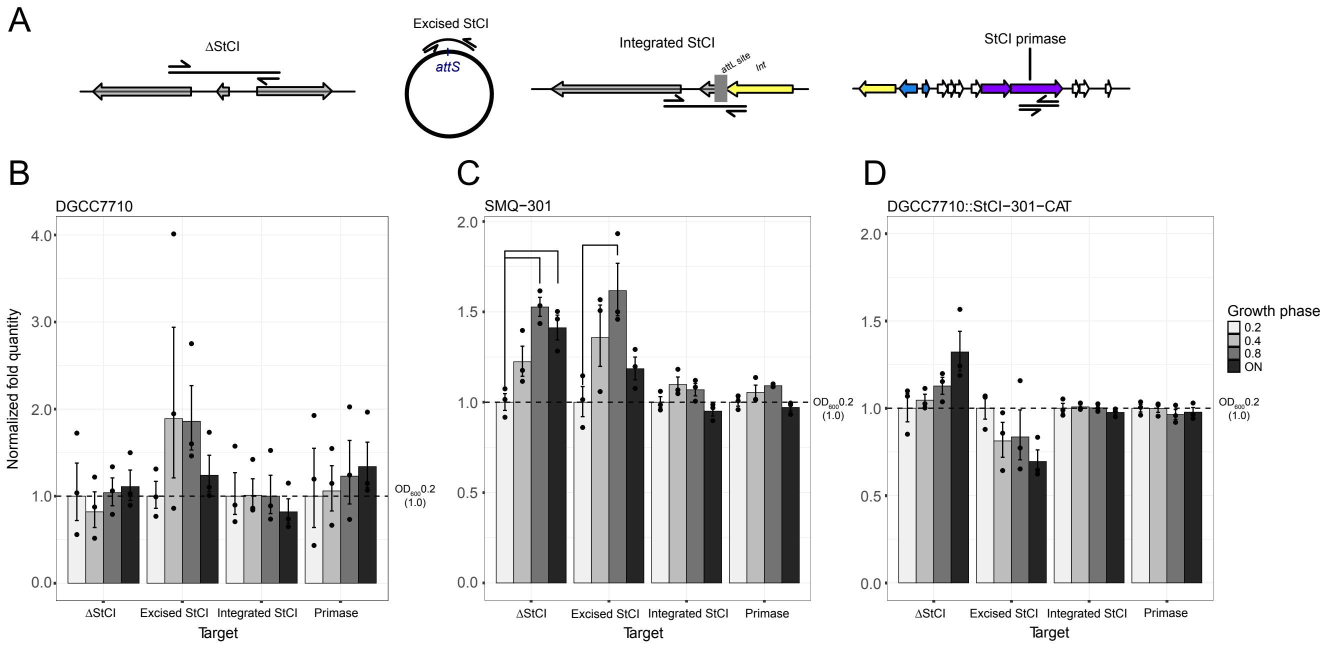

Supp. Figure 7: Relative qPCR results to quantify StCI excision and replication at various growth phases of *S.t.* strains DGCC7710, SMQ-301, and DGCC7710::StCI-301-CAT. Strains were grown in LM17 medium at 42°C and samples were collected at OD_{600} of 0.2, 0.4, 0.8, and after an overnight incubation (ON). The relative qPCR assays were performed on extracted genomic DNA, using primers targeting the StCI *pri* gene (coding for a primase), the excised StCI, the integrated StCI, as well as both StCI flanking genes to detect an empty *attC* site (ΔStCI). The relative quantity of each target was normalized to the housekeeping genes *gyrA* and *gyrB* and to the sample at OD_{600} 0.2 for each strain using the ΔΔCq method[4]. Fold changes are plotted on a linear scale, statistical significance brackets indicate a significant difference (p-value ≤ 0.05) between the corresponding samples from the same target. Error bars represent the mean ± standard error of the mean and were calculated on log_2-transformed data[4]. Statistical tests were conducted on log_2-transformed data[4]. Panel A: Schematic representation of the amplicon position for the ΔStCI, excised StCI, integrated StCI, and StCI primase targets. Panels B, C and D: qPCR data for strains DGCC7710, SMQ-301, and DGCC7710::StCI-301-CAT, respectively. Black dots represent the normalized fold quantity for each biological assay (n = 3). The significant p-values (< 0.05) are available in Supplementary Table S4.

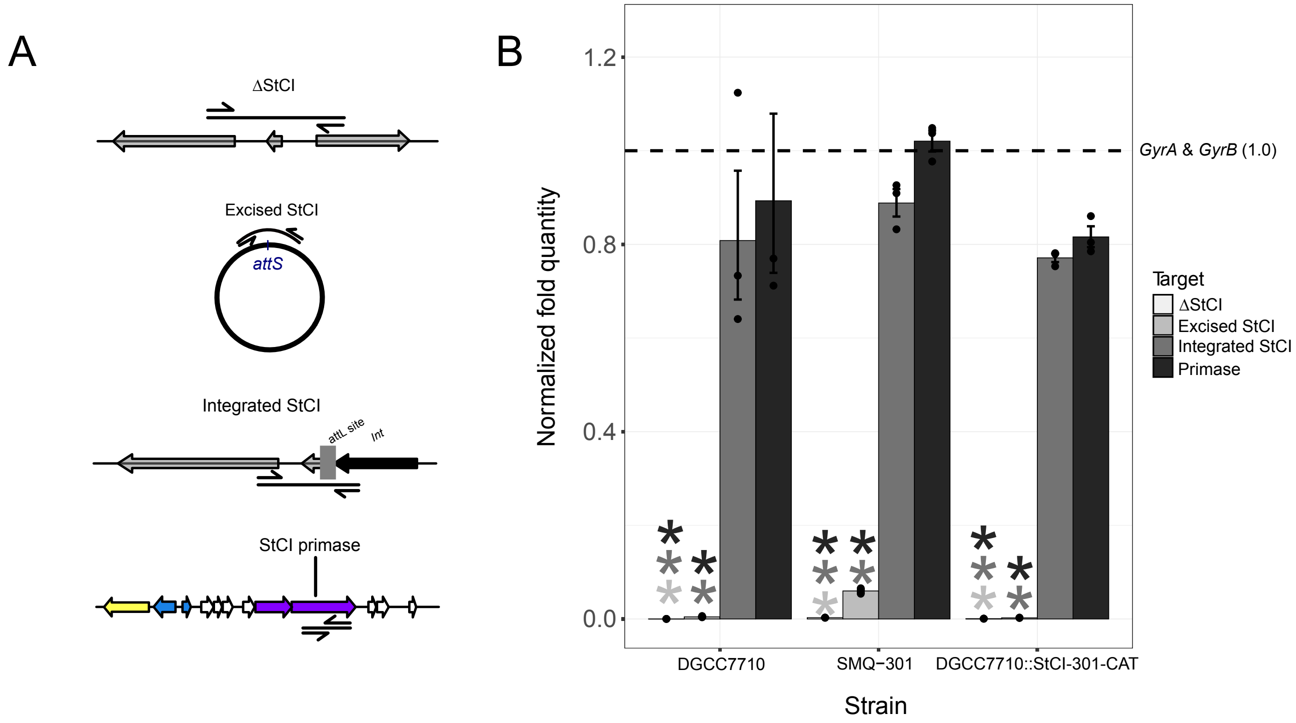

Supp. Figure 8: Relative qPCR results to quantify the StCI excision and replication levels in overnight cultures for *S.t.* strains DGCC7710, SMQ-301, and DGCC7710::StCI-301-CAT strains. Panel A: Schematic representation of the amplicon position for the ΔStCI, excised StCI, integrated StCI, and StCI primase targets. Panel B: Strains were grown overnight in LM17 at 42°C. Relative qPCR assays were conducted on extracted genomic DNA, using primers targeting the StCI *pri* gene, the excised StCI, the integrated StCI, and both StCI flanking genes to detect an empty *attC* site (ΔStCI). The relative quantity of each target was normalized to the housekeeping genes *gyrA* and *gyrB* in each strain using the ΔCq method[5]. An asterisk above a condition indicates that there is a significant difference (p-value ≤ 0.05) between this condition and the one sharing the asterisk shade/color (from the same strain). Each condition with an asterisk has a lower normalized fold quantity value than the color/shade matching condition. Fold change is plotted on a linear scale. Error bars represent the mean ± standard error of the mean and were calculated on $\log_2$-transformed data[4]. Statistical tests were conducted on $\log_2$-transformed data[4]. Black dots represent the normalized fold quantity for each biological assay (n = 3). The significant p-values (< 0.05) are available in Supplementary Table S4.

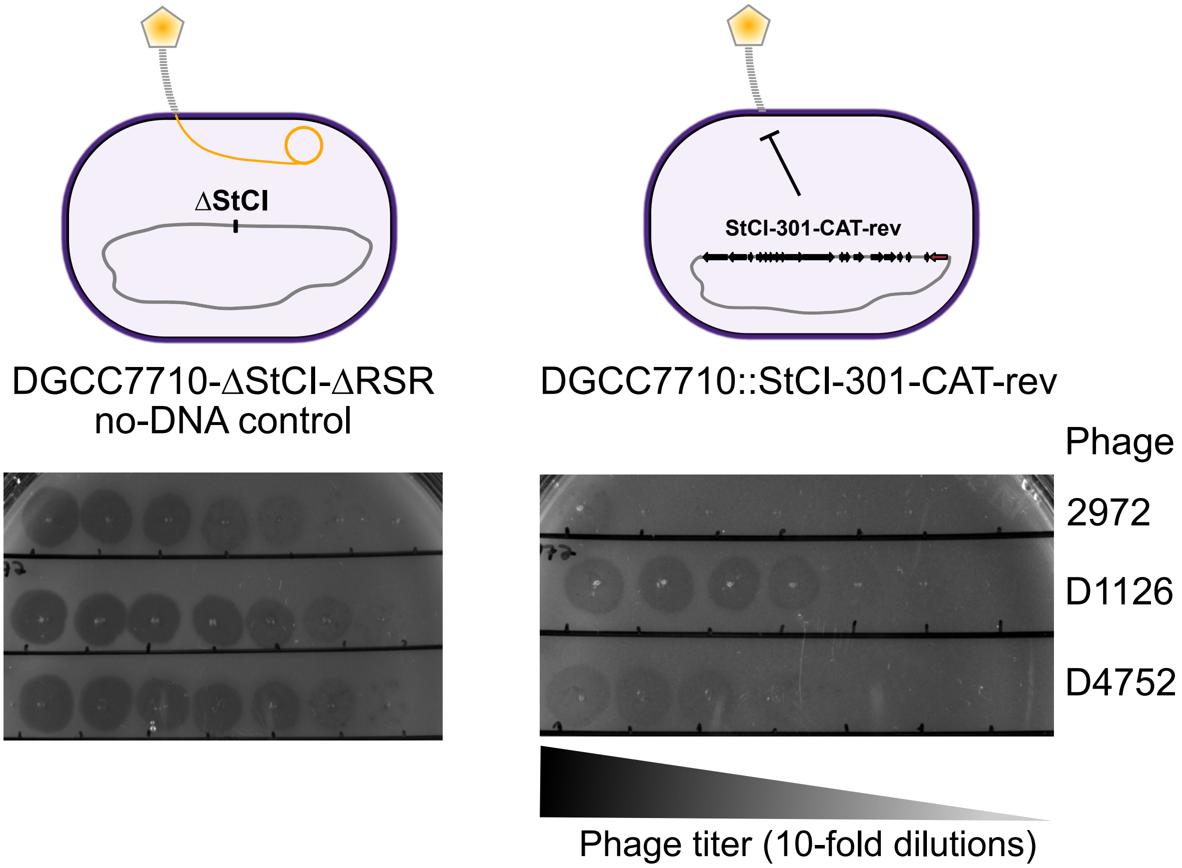

Supp. Figure 9: Phage resistance profile of *S. thermophilus* strains. The engineered StCI from *S.t.* SMQ-301, containing a chlroramphenicol resistance gene in the reverse orientation (StCI-301-CAT-rev), was mobilized into strain *S.t.* DGCC7710-ΔStCI-ΔRSR by natural competence. A no-DNA control was used in parallel. Strain DGCC7710-ΔStCI-ΔRSR (no-DNA control) was sensitive to virulent phages while strain DGCC7710 containing the StCI-301-CAT-rev was resistant to some virulent phages. Plaque assays are representatives of three experiments.

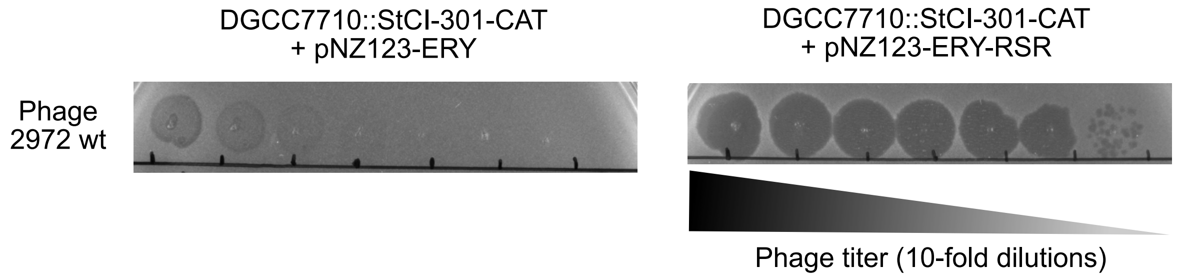

Supp. Figure 10: Phage resistance profile of *S. thermophilus* strains. The StCI of strain *S.t.* SMQ-301, engineered to contain a chlroramphenicol resistance gene (StCI-301-CAT), was mobilized to strain *S.t.* DGCC7710-ΔStCI-ΔRSR by natural competence. A crRNA targeting the StCI-301 integrase (RSR) was cloned into pNZ123-ERY. This plasmid and the empty plasmid were transformed separately into *S.t.* DGCC7710 containing the StCI-301-CAT (DGCC7710::StCI-301-CAT). The RSR plasmid was used to delete the StCI-301-CAT previously mobilized into DGCC7710-ΔStCI-ΔRSR and to verify its phage resistance profile. Strain DGCC7710::StCI-301-CAT containing the pNZ123-ERY was more resistant to phage 2972 while the strain containing the RSR plasmid reverted to a phage sensitive profile. Plaque assays are representatives of three experiments.

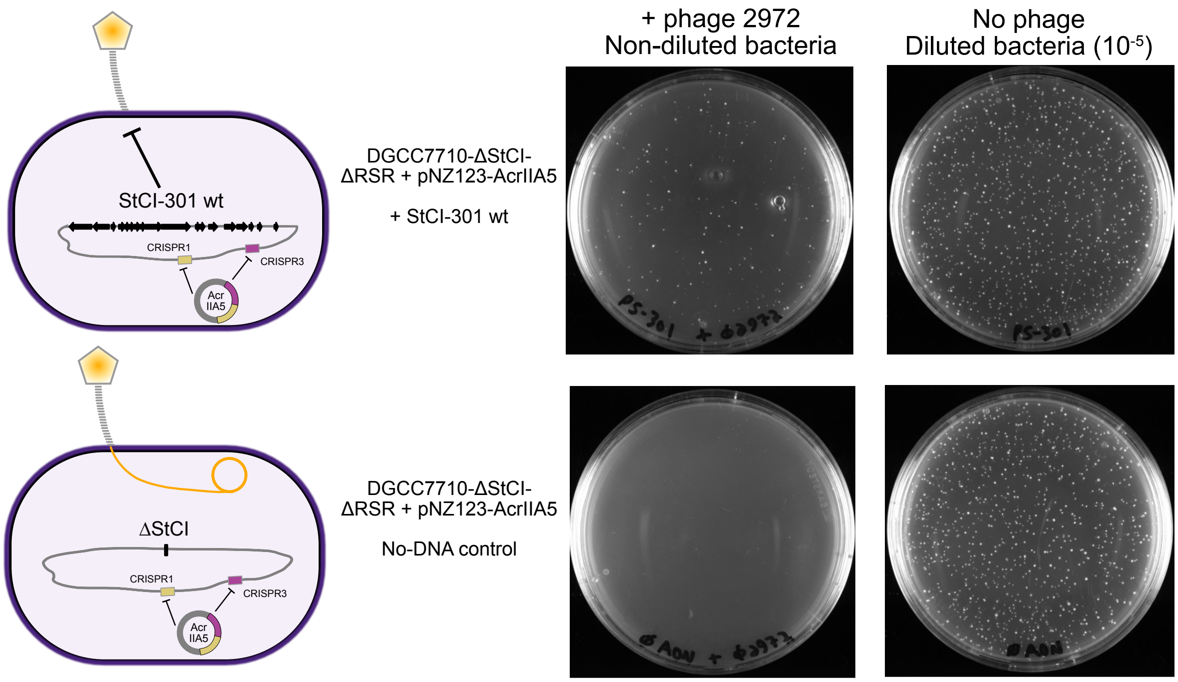

Supp. Figure 11: The StCI of S.t. SMQ-301 (StCI-301) was mobilized by natural competence into the ΔStCI-ΔRSR derivative of S.t. DGCC7710 containing pNZ123-AcrIIA5. Cells containing StCI-301 were selected with phage 2972. Controls with no StCI DNA (no-DNA control) with and without phages were also performed. The transformed cultures were added to 10 ml of LM17 and grown overnight at 37°C. Cultures were then infected with the virulent phage 2972 in 3 ml of top LM17 agar (+10 mM $CaCl_2$) and poured on LM17 agar (+10 mM $CaCl_2$). In the StCI-301 transformation, an average of $1.92 \pm 1.28 \times 10^8$ cfu/ml was obtained with no phage selective pressure and 599 ± 30 cfu/ml with phages. Assays were performed twice.

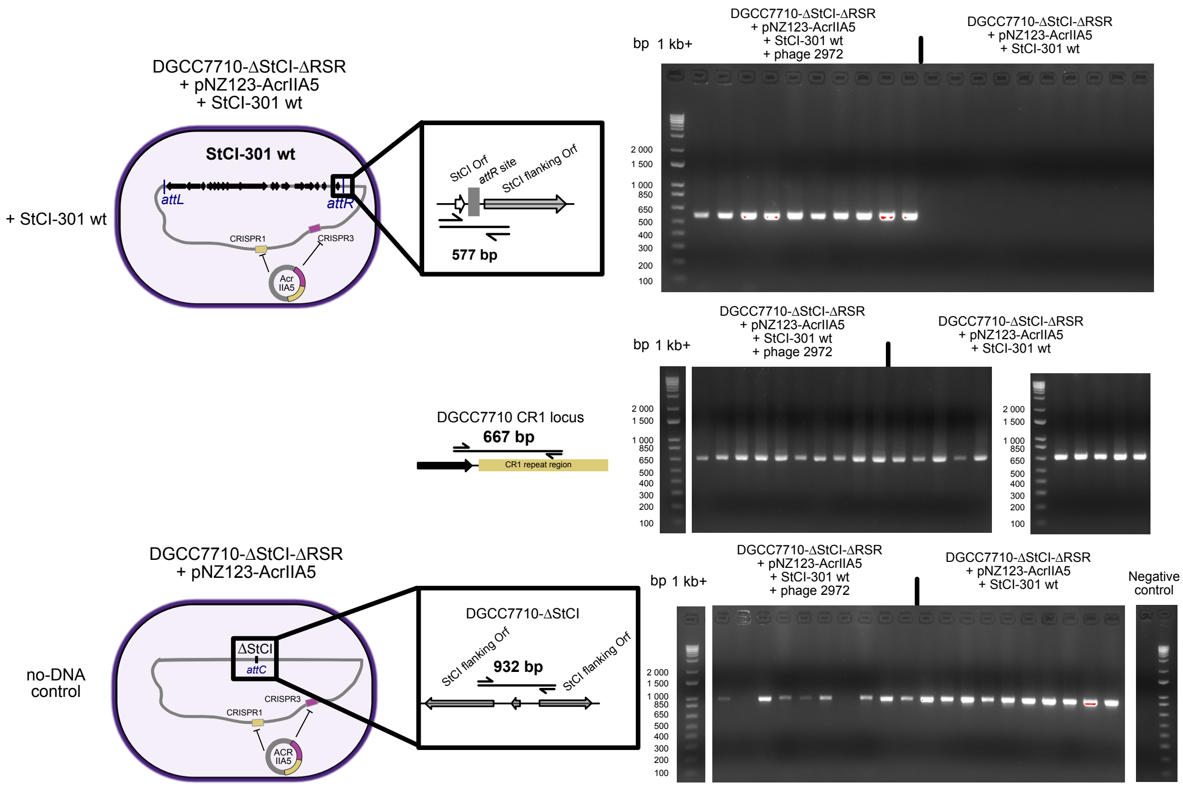

Supp. Figure 12: The StCI of *S.t.* SMQ-301 was mobilized into the ΔStCI-ΔRSR derivative of *S.t.* DGCC7710 containing the plasmid pNZ123-AcrIIA5. The presence/absence of the new integrated StCI was confirmed by PCR in 10 colonies obtained with phage selection and in 10 colonies obtained from the control without phages. The strain ID was confirmed by the spacer content of its CRISPR1 locus. PCR products were migrated on a 2% agarose gel.

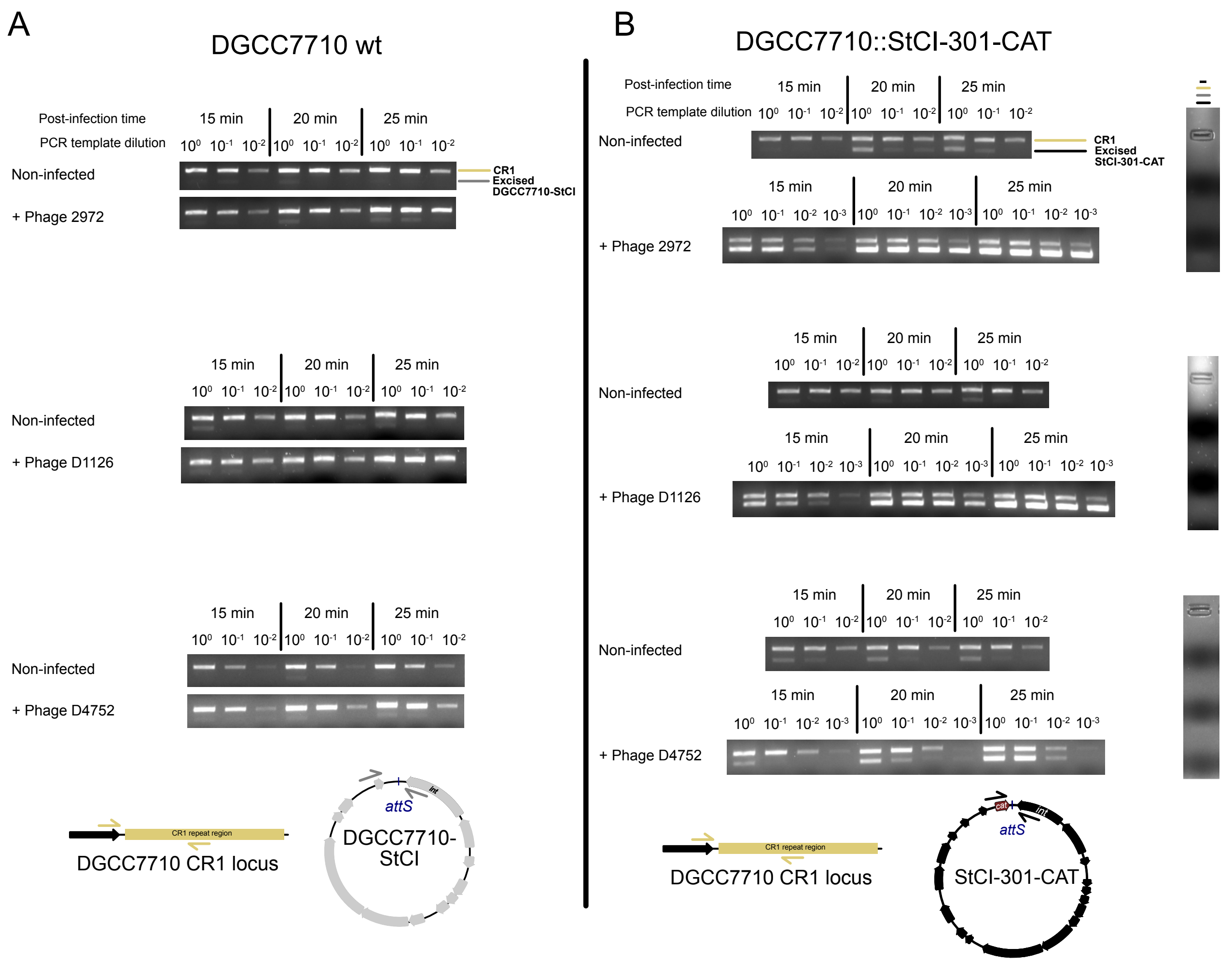

Supp. Figure 13: Virulent phages 2972, D1126, and D4752 do not excise the wild-type DGCC7710-StCI, but they excise the StCI-301-CAT. Excision of the DGCC7710-StCI (Panel A) and StCI-301-CAT (Panel B) by phages 2972, D1126, and D4752 were verified by semi-quantitative PCR. Strains DGCC7710 (wild-type) and DGCC7710::StCI-301-CAT were infected by these phages at a MOI of ~5. Non-infected *S.t.* strains were tested in parallel as controls. Then, bacterial samples were collected at 15, 20, and 25 minutes, post-infection. PCR reactions with primers targeting the excised StCI (CM5-circ_F/SP_circ_R or DGCC7710_SP_circ_F5/SP_circ_R) and the CRISPR1 (CR1) locus (Yc70/RDS7rev) to confirm strain ID were performed separately on 10-fold dilutions of the samples. Phages were amplified twice on *S.t.* DGCC7710. Assays were performed twice.

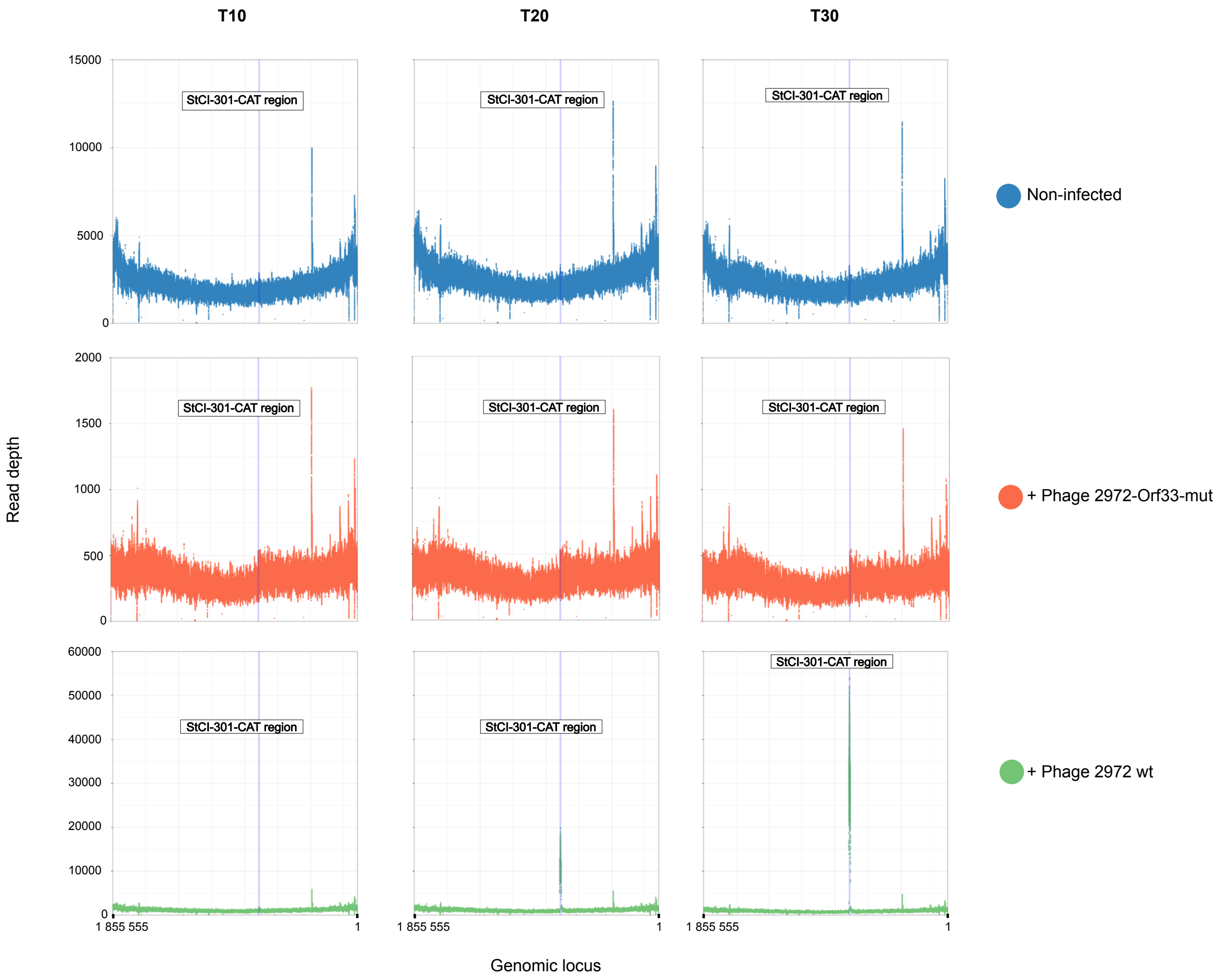

Supp. Figure 14: Orf33 of phage 2972 is essential for the induction of StCI-301-CAT in strain DGCC7710::StCI-301-CAT. This strain was infected by phage 2972 wt and phage 2972-Orf33-mut at a MOI of 10. A non-infected control was carried out in parallel. Samples were collected at 10, 20, and 30 min post-infection. Genomic DNA was extracted and Illumina-sequenced. Paired-end reads were mapped against strain DGCC7710::StCI-301-CAT sequence (whole genome is shown), created using the GenBank NZ_CP025216 file and the StCI-301-CAT sequence. We observed a few differences between the NZ_CP025216 sequence and from our DGCC7710 sequence. There is a gradual increase of the read depth at the StCI-301-CAT genomic positions over time only when the strain is infected by the wild-type phage 2972. The StCI-301-CAT genomic region is indicated by a blue vertical line.

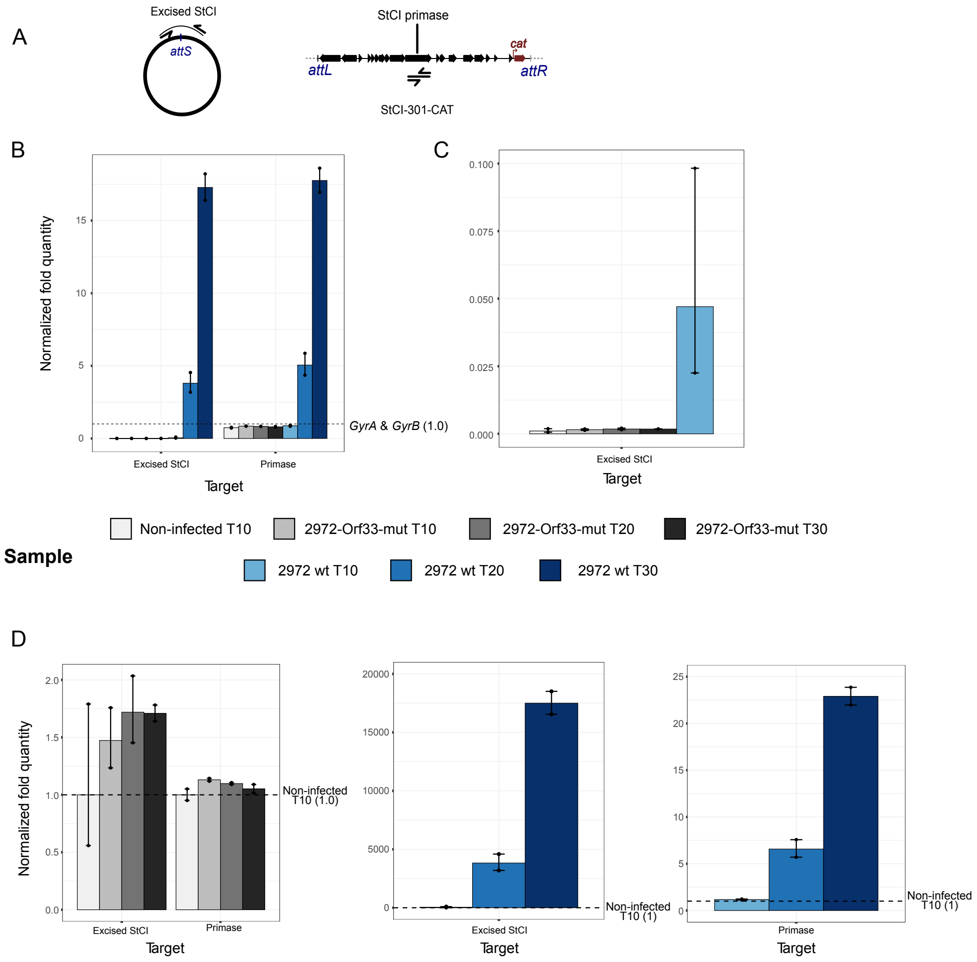

Supp. Figure 15: Relative qPCR results to quantify StCI excision and replication in *S.t.* DGCC7710::StCI-301-CAT (SMQ-1512) when infected with the wild-type virulent phage 2972 and phage 2972-Orf33-mut over time (T10, T20, and T30). Panel A: Schematic representation of the amplicon position for the excised StCI and StCI primase targets. Panel B: There is no increase over time in the relative quantity of excised StCI and in the StCI primase in bacterial samples infected with phage 2972-Orf33-mut. In contrast, when strain DGCC7710::StCI-301-CAT is infected by the wild-type phage 2972, there is an increase over time of the excised StCI and the StCI primase, indicating that StCI-301-CAT is replicating. The relative qPCR assays were conducted on extracted genomic DNA, using primers targeting the StCI *pri* gene (primase), the excised StCI, and housekeeping genes *gyrA* and *gyrB*. The relative quantity of each target was only normalized to the housekeeping genes *gyrA* and *gyrB*, in each strain, using the ΔCq method[5]. Fold changes are plotted on a linear scale, error bars represent the mean ± standard error of the mean and were calculated on $\log_2$-transformed data[4]. Statistical tests were conducted on $\log_2$-transformed data[4]. Black dots represent the normalized fold quantity for each biological assay (n = 2). Panel C: Zoom on Panel B for the non-infected samples, the samples infected with phage 2972-Orf33-mut and the wild-type phage 2972 at T10 (excised StCI target). Panel D: The relative quantity of each target in Panel B was normalized to the housekeeping genes *gyrA* and *gyrB* and to the non-infected sample (T10) using the ΔΔCq method[4].

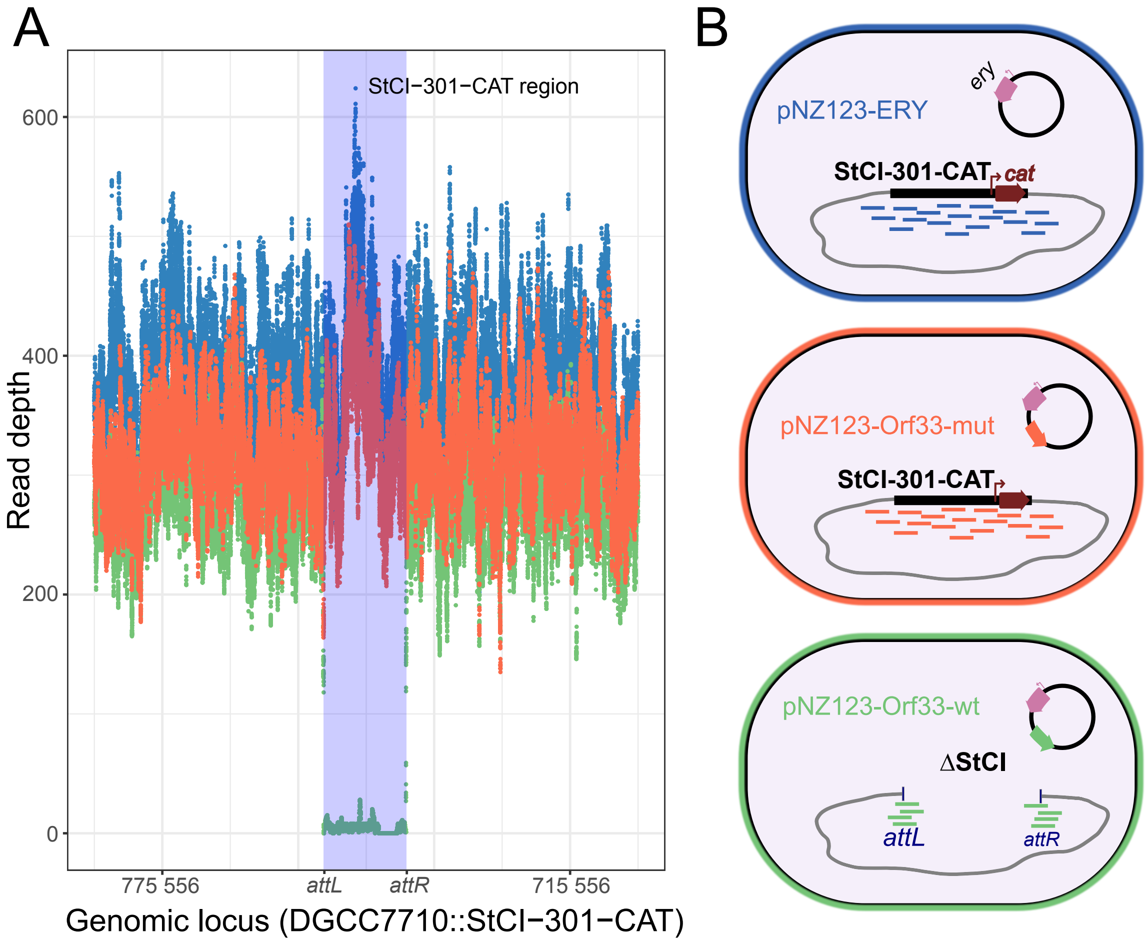

Figure 16: The Orf33 of phage 2972 promotes the StCI-301-CAT excision. Panel A: The genes coding for the wild-type and mutated Orf33 of phage 2972 were cloned in the plasmid pNZ123-ERY (pNZ123-Orf33-wt and pNZ123-Orf33-mut). The two constructed plasmids and the empty plasmid were transformed separately into *S.t.* DGCC7710 containing the engineered StCI of *S.t.* SMQ-301 (DGCC7710::StCI-301-CAT). Bacterial strains were grown to an OD_{600nm} of 0.5 with erythromycin. The bacterial DNA from the three strains were extracted and sequenced (Illumina). Paired-end reads were mapped against the DGCC7710::StCI-301-CAT sequence. There was a significant drop in the number of reads with only the strain containing pNZ123-Orf33-wt. The StCI-301-CAT genomic region is indicated with a purple rectangle (region from the *attL* site to the nucleotide before the *attR* site). Only a small section of the whole genome is shown (see Supp. Figure 17 for the complete genome read depth). Assays were performed three times. Panel B: Schematic representation of the experiment results.

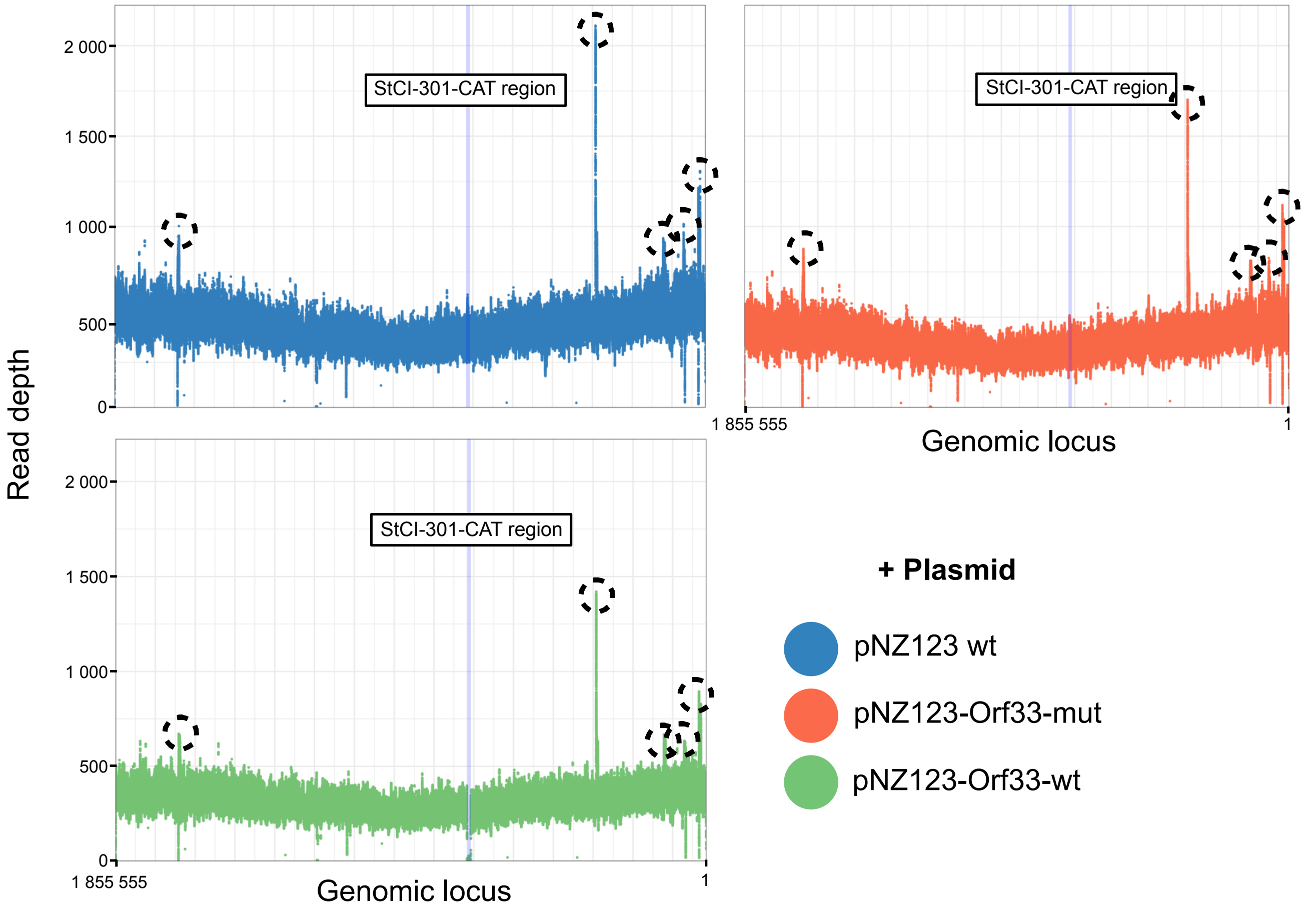

Supp. Figure 17: Extended view of Panel A of Supp. Figure 16. The Orf33 of phage 2972 is essential for the excision of StCI-301-CAT. The genes coding for the wild-type and mutated Orf33 of phage 2972 were cloned in the plasmid pNZ123-ERY (pNZ123-Orf33-wt and pNZ123-Orf33-mut). The two plasmid constructs and the empty plasmid were introduced separately in strain *S.t.* DGCC7710 containing the engineered StCI of *S.t.* SMQ-301 (DGCC7710::StCI-301-CAT). Bacterial strains were grown to an OD_{600} of 0.5 with erythromycin. Bacterial DNA from the three strains were extracted and sequenced (Illumina). Paired-end reads were mapped against the DGCC7710::StCI-301-CAT sequence (whole genome is shown), created using the GenBank NZ_CP025216 file and the StCI-301-CAT sequence. We observed a few differences between the NZ_CP025216 sequence and our DGCC7710 sequence. The StCI-301-CAT genomic region is indicated by a vertical blue line. Circled peaks are rRNA genomic regions. Assays were performed three times.

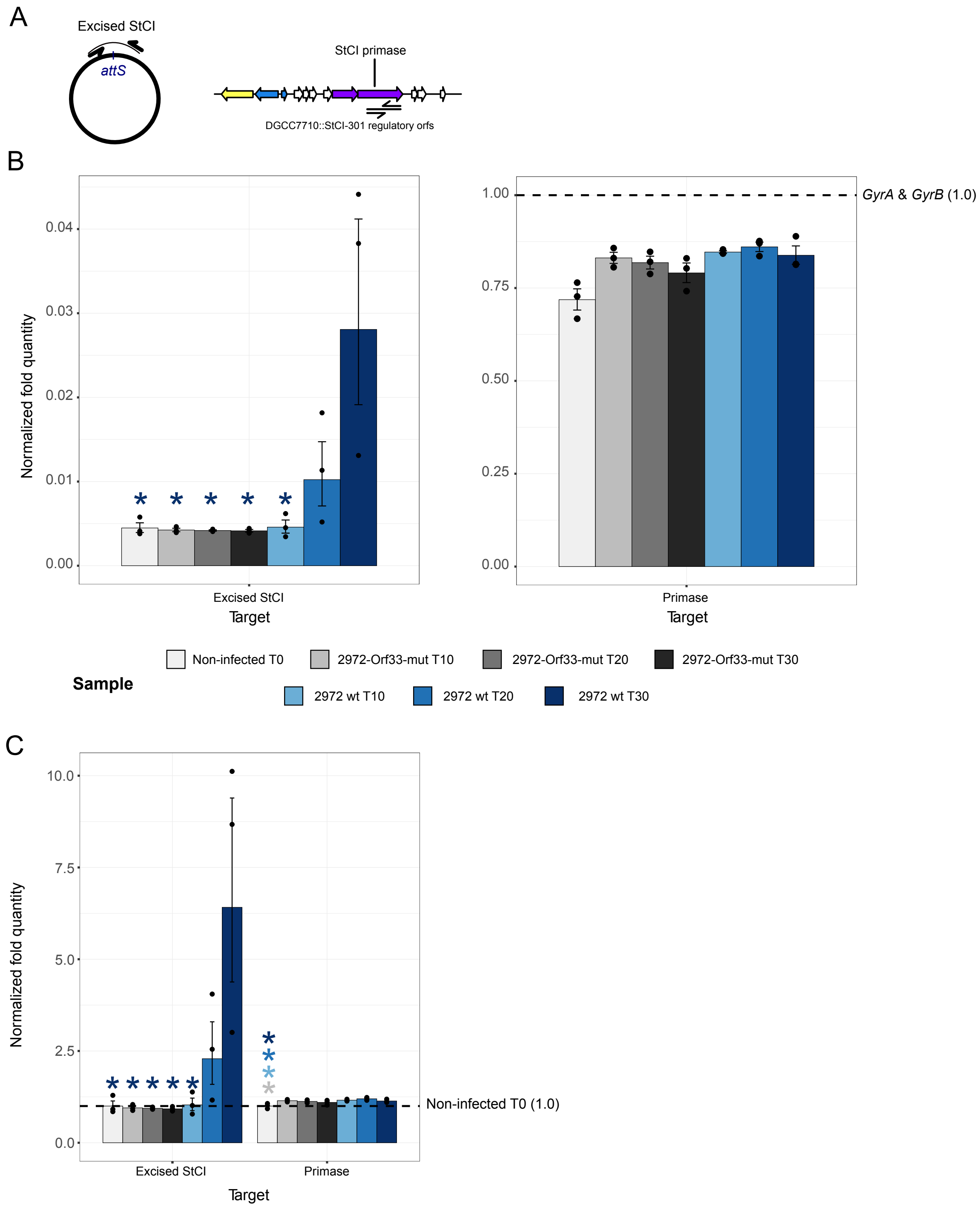

Supp. Figure 18: Relative qPCR results to quantify the StCI excision and replication in strain *S.t.* DGCC7710 containing the StCI-301 regulatory *orfs* (SMQ-1528) when infected with the wild-type phage 2972 and phage mutant 2972-Orf33-mut over time (T10, T20, and T30). Panel A: Schematic representation of the amplicon position for the excised StCI and StCI primase targets. Panel B: The relative qPCR experiment was conducted on extracted genomic DNA, using primers targeting the StCI *pri* gene (primase), the excised StCI, and the housekeeping genes *gyrA* and *gyrB*. The relative quantity of each target was only normalized to the housekeeping genes *gyrA* and *gyrB*, in each strain, using the ΔCq method[5]. Fold changes are plotted on a linear scale. Error bars represent the mean ± standard error of the mean and were calculated on log_2-transformed data as previously described[4].

qPCR measurements were done in technical triplicate. An asterisk above a condition indicates that there is a significant difference (p-value ≤ 0.05) between this condition and the one sharing the asterisk shade/color (from the same target). Each condition with an asterisk has a lower normalized fold quantity value than the color/shade matching condition. Statistical tests were conducted on $\log_2$-transformed data[4]. Black dots represent the normalized fold quantity for each biological assay (n = 3). The significant p-values (< 0.05) are available in Supplementary Table S4. Panel C: The relative quantity of each target from Panel B was normalized to the housekeeping genes *gyrA* and *gyrB* and to the non-infected sample (T0) using the $\Delta\Delta$Cq method[4]. The significant p-values (< 0.05) are available in Supplementary Table S4.

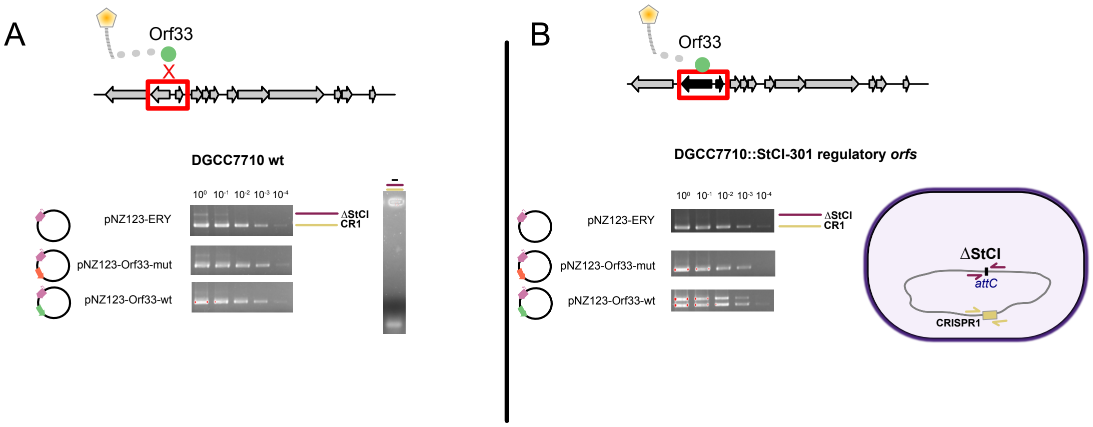

Supp. Figure 19: The Orf33 from phage 2972 (green circle) excises the DGCC7710-StCI only when it contains the StCI-301 regulatory *orfs*. The Orf33 does not trigger the native DGCC7710-StCI excision (Panel A). On the other hand, Orf33 is involved in the excision of the DGCC7710-StCI containing the regulatory region of the StCI-301 (Panel B). The genes coding for the wild-type Orf33 and mutated Orf33 of phage 2972 were cloned in the plasmid pNZ123-ERY (pNZ123-Orf33-wt and pNZ123-Orf33-mut). The two constructed plasmids and the empty plasmid were transformed separately into *S.t.* DGCC7710 (Panel A) and into DGCC7710::StCI-301 regulatory *orfs* (Panel B). The StCI excision in *S.t.* DGCC7710 and in *S.t.* DGCC7710::StCI-301 regulatory *orfs* by the constructed plasmids was estimated by semi-quantitative PCR. Bacterial strains were grown to an O.D.$_{600nm}$ of 0.5 with erythromycin. PCR reactions with primers (SMQ-F/SMQ-R) targeting the StCI-free integration site IS_a and with primers (Yc70/RDS7rev) targeting the CR1 locus were performed separately on 10-fold serial dilutions of each sample. Assays were performed two times. Genomic maps were produced in R software (version 4.3.2) using *genoPlotR* package[2,3].

A

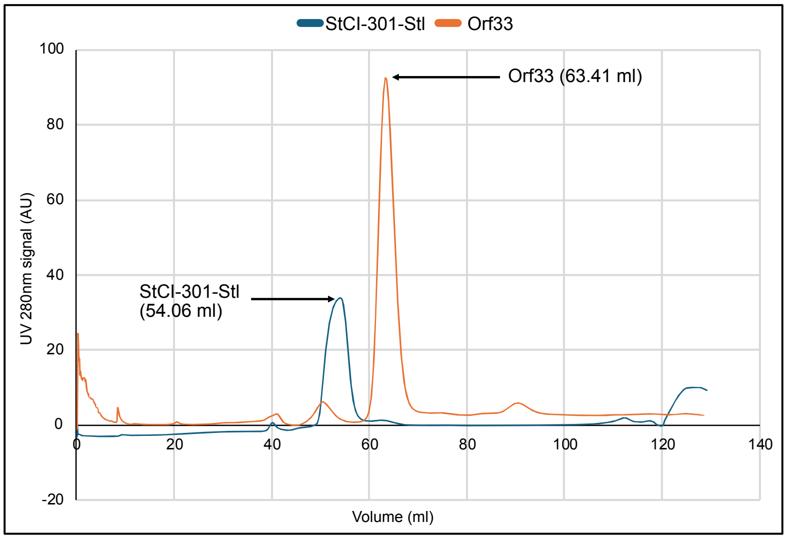

B

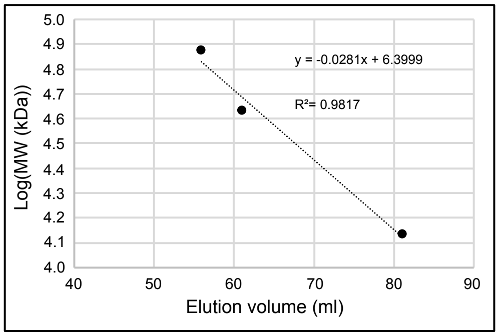

C

	Theoretical MW (kDa)	Calculated MW (kDa)
StCl-301-Stl	34	76
Orf33	19	42

Supp. Figure 20: Size exclusion chromatography (SEC) analysis of StCI-301-Stl and phage-encoded Orf33. Panel A: Chromatogram of Orf33 and StCI-301-Stl elution profiles. Panel B: SEC elution standard curve made with conalbumin (75 kDa), ovalbumin (43 kDa), and ribonuclase A (13.7 kDa) standards. Panel C: Calculated molecular weights (MW) of StCI-301-Stl and Orf33 using the standard curve presented in Panel B. Calculated MWs indicate folding of Orf33 and StCI-301-Stl as dimers. Theoretical molecular weights include 6xHis-tags fused to both proteins.

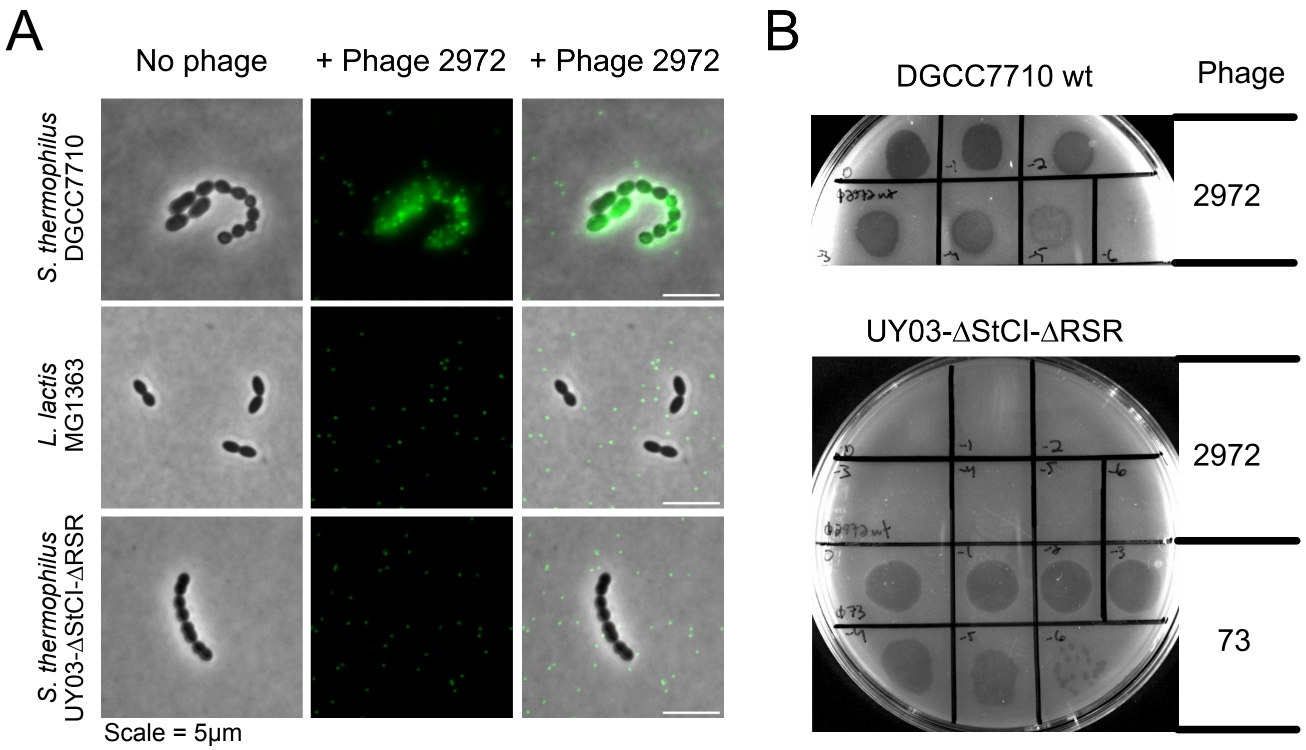

Supp. Figure 21: Phage adsorption assay using fluorescence microscopy. Panel A: The wild-type phage 2972 adsorbs to its host *S.t.* DGCC7710 but it does not adsorb to *S.t.* UY03-ΔStCI-ΔRSR and *Lactococcus cremoris* MG1363. The first column represents the bacterial strains with no phage added. Phage 2972 lysate was stained with SYBR Gold and treated with RNAse and DNAse. The three strains were grown to an OD_{600} of 0.6. Then, 1 ml of each strain was centrifuged and resuspended twice in phage buffer with 10 mM $CaCl_2$. Phage and bacteria were mixed at an MOI of ~50 and incubated at room temperature for 1 minute. The phage-bacteria mix was vortexed 15 sec and 5 µl of the phage-bacteria mix was put on an 1% agarose pad[7], covered with a coverslip. Images were taken with a 100x plan fluorite oil phase Ph3 objective (0.09s, high gain) in the first and third column, and with FITC (2.5 s, low gain - EX: 470/40, EM: 525/50), in the second column. Images were further treated with Fiji[8]. Adsorption assays were conducted in biological triplicate. Panel B: Phage resistance profile of *S. thermophilus* strains. The UY03-ΔStCI-ΔRSR strain was resistant to phage 2972 but sensitive to phage 73. Plaque assays are representatives of two experiments.

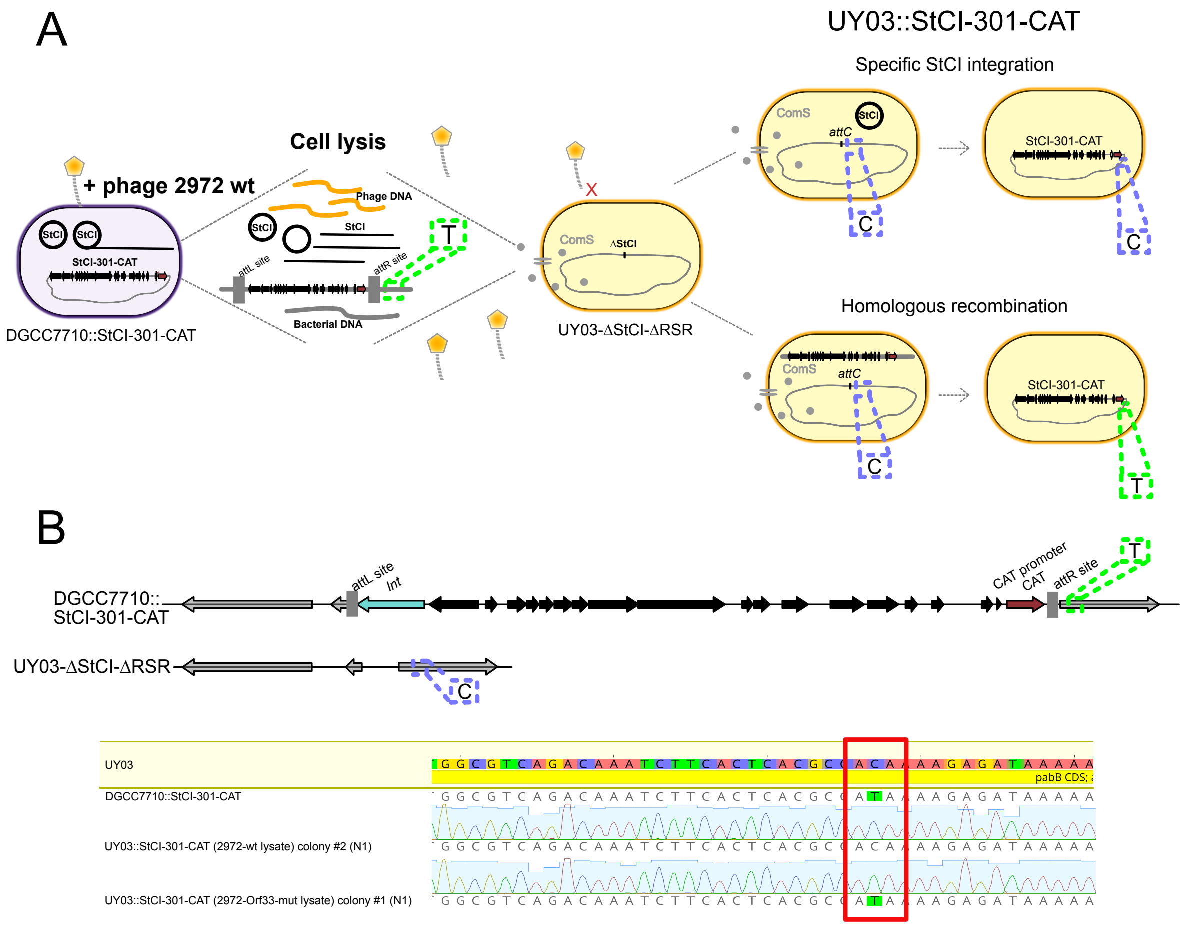

Supp. Figure 22: Details of the StCI-301-CAT mobilization to *S.t.* strain UY03-ΔStCI-ΔRSR. Panel A: Phage 2972 and phage 2972-Orf33-mut were amplified on *S.t.* strain DGCC7710-StCI-301-CAT. The lysates were filtered and used in a natural competence assay with *S.t.* UY03-ΔStCI-ΔRSR as well as with and without the natural competence inducing peptide ComS[9]. The PCR products from the colonies obtained in the StCI mobilization experiment using phage lysates, by natural competence, were Sanger-sequenced (Figure 9). Panel B: Sequencing revealed 100% identity with the reference sequence containing the StCI-301-CAT into UY03-ΔStCI-ΔRSR for all the tested StCI+ colonies (N=2) with the phage 2972 lysate condition (only one colony is shown). We repeated the same method with the single StCI+ UY03 colony obtained using the phage 2972-Orf33-mut lysate, and we detected a SNP (T) in the StCI-301-CAT flanking region that matches the DGCC7710::StCI-301-CAT DNA sequence (T). The SNP is a T in the strain DGCC7710::StCI-301-CAT sequence instead of a C in the recipient strain UY03-ΔStCI-ΔRSR sequence. Nucleotide alignments were conducted with Geneious software (version 11.1.5). Genomic maps were produced in R software (version 4.3.2) using *genoPlotR* package[2,3].

Table S1: *Streptococcus thermophilus* strains used in this study with their StCI features.

GenBank Accession Number	*Streptococcus thermophilus* Strain	StCI	PICI Type[a]	StCI Length (bp)	StCI Genomic Positions	Putative *attL-attR* sites	IS [b]	StCI %GC	Host Strain %GC	VirClust Cluster [e]	Javan *et al.*, 2019 [c]
CP000023.1	LMG18311	No	ND						39.3		
CP000419.1	LMD-9	Yes	C	11 388	756 545 - 745 158	ATATTTCCCTCTAAAATC	IS_a	36.1	39.1	1	Javan607
CP002340.1	ND03	Yes	C	9 969	745 783 - 735 815	ATATTTCCCTCTAAAATC	IS_a	35.8	39.0	2	Javan611
CP003499.1	MN-ZLW-002	Yes	C	7 853	736 398 - 728 546	ATATTTCCCTCTAAAATC	IS_a	37.1	39.1	2	Javan610
CP006819.1	ASCC1275	Yes	C	7 795	934 143 - 926 349	ATATTTCCCTCTAAAATC	IS_a	37.5	39.1	2	Javan601
CP010999.1	MN-BM-A02	Yes	C	7 795	744 100 - 736 306	ATATTTCCCTCTAAAATC	IS_a	37.5	39.0	2	Javan609
CP012588.1	MN-BM-A01	Yes	C	7 852	550 066 - 542 215	ATATTTCCCTCTAAAATC	IS_a	37.1	39.1	2	Javan608
CP013939.1	S9	No	ND						39.0		
CP016026.1	KLDS SM	Yes	C	7 795	61 108 - 53 314	ATATTTCCCTCTAAAATC	IS_a	37.5	39.1	2	Javan605
CP016394.1	ND07	Yes	C	7 795	68 489 - 76 283	ATATTTCCCTCTAAAATC	IS_a	37.5	39.0	2	Javan612
CP016439.1	CS8	Yes	C	8 157	179 106 - 187 262	ATATTTCCCTCTAAAATC	IS_a	37.3	39.0	2	Javan603
CP016877.1	KLDS 3.1003	Yes	C	~17 643	1484190 - 1501832	NA	IS_e	~37.9	38.9	4	
CP017064.1	ST3	Yes	C	11 389	763 446 - 752 058	ATATTTCCCTCTAAAATC	IS_a	36.1	39.0	1	
CP019935.1	APC151	Yes	C	9 968	1795464 - 1785497	ATATTTCCCTCTAAAATC	IS_a	35.8	39.1	2	
CP022547.1	B59671	No	ND						39.1		
CP025399.1	GABA	No	ND						39.1		
CP025400.1	EPS	Yes	C	8 210	1028153 - 1036362	ATATTTCCCTCTAAAATC	IS_a	37.5	39.0	2	
CP028896.1	CS5	Yes	C	7 795	753 401 - 745 607	ATATTTCCCTCTAAAATC	IS_a	37.5	39.1	2	
CP030250.1	CS20	Yes	C	7 818	789 809 – 781 992	ATATTTCCCTCTAAAATC	IS_a	37.2	38.9	2	
CP030927.1	CS9	No	ND						38.9		
CP030928.1	CS18	Yes	C	7 795	752 409 - 744 615	ATATTTCCCTCTAAAATC	IS_a	37.5	39.1	2	
CP031545.1	ST109	No	ND						39.2		
CP031881.1	ST106	No	ND						39.3		
CP035306.1	IDCC2201	No	ND						39.2		
CP038020.1	ATCC19258	Yes	C	9 217	416 422 - 425 638	ATTTCATGAAAAAATAC	IS_b	36.6	39.0	5	
CP045596.1	TK-P3A	Yes	C	12 410	890 094 - 902 503	ATTCCATGAAAAAATAC / ATTTCATGAAAAAATAC	IS_b	37.2	39.1	5	
CP046134.1	MAG_mrk202_sterm	Yes	C	15 855	1089056 - 1104910	ATATTTCCCTCTAAAATC	IS_a	34.1	39.0	1	
CP047191.1	EU01	Yes	D	10 943	584 931 - 595 870	AATTCTACAACAAAAT	IS_c	33.7	38.9	3	
CP049053.1	ST64987	No	ND						39.0		
CP050870.1	CS6	Yes	C	7 795	751 595 - 743 801	ATATTTCCCTCTAAAATC	IS_a	37.5	39.1	2	
CP061019.1	24853	Yes	C	15 855	762 320 - 746 466	ATATTTCCCTCTAAAATC	IS_a	34.1	39.0	1	
CP061020.1	24740	Yes	D	10 942	1846863 -1835922	AATTCTACAACAAAAT	IS_c	33.7	38.9	3	

CP061021.1	24739	Yes	C	9 144	775 850 - 766 707	ATATTTCCCTCTAAAATC	IS_a	36.6	39.1	2
CP061022.1	24738	Yes	D	10 942	1852786 - 1841845	AATTCTACAACAAAAT	IS_c	33.7	39.0	3
CP061023.1	13499	Yes	D	10 942	1852540 - 1841599	AATTCTACAACAAAAT	IS_c	33.7	39.0	3
CP061024.1	13498	Yes	C	15 855	777 875 - 762 021	ATATTTCCCTCTAAAATC	IS_a	34.1	39.0	1
CP061025.1	13496	Yes	C	9 144	777 942 - 768 799	ATATTTCCCTCTAAAATC	IS_a	36.6	39.1	2
CP063275.1	DMST-H2	Yes	C	7 902	795 892 - 787 991	ATATTTCCCTCTAAAATC	IS_a	37.1	39.0	2
CP065477.1	4078	Yes	C	9 969	751 455 - 741 487	ATATTTCCCTCTAAAATC	IS_a	35.8	39.1	2
CP065483.1	CNRZ1151	No	ND						39.1	
CP065495.1	CNRZ385	No	ND						39.1	
CP069275.1	EG007	Yes	C	18 658	54 594 - 73 251	ATTCCATGAAAAAATAC / ATTTCATGAAAAAATAC	IS_b	38.4	39.1	4
CP089060.1	CH8	No	ND						39.3	
CP094945.1	VHProbi R08	Yes	C	7 625	736 257 - 728 633	ATATTTCCCTCTAAAATC	IS_a	37.2	39.1	2
CP101646.1	UCCSt95	No	ND						39.2	
CP102538.1	TH-4	Yes	C	11 388	1250226 -1261613	ATATTTCCCTCTAAAATC	IS_a	36.1	39.1	1
CP102797.1	ST057-1	No	ND						39.1	
CP113237.1	TCI633	No	ND						39.0	
CP116772.1	TSGB 4141	No	ND						39.1	
FR875178.1	JIM8232	No	ND						38.9	
LR822006.1	STH_CIRM_16	Yes	C	7 754	746 980 - 739 227	ATATTTCCCTTCTAAAATC / ATATTTCCCTCTAAAATC	IS_a	37.4	39.0	2
LR822008.1	STH_CIRM_18	Yes	C	15 854	776 025 - 760 172	ATATTTCCCTCTAAAATC	IS_a	34.1	39.1	1
LR822009.1	STH_CIRM_19	Yes	C	15 861	754 560 - 738 700	ATATTTCCCTCTAAAATC	IS_a	34.1	39.0	1
LR822010.1	STH_CIRM_29	No	ND						39.1	
LR822011.1	STH_CIRM_23	Yes	C	8 159	1007593 - 1015751	ATATTTCCCTCTAAAATC	IS_a	37.3	39.0	2
LR822012.1	STH_CIRM_30	Yes	C	8 192	728 910 - 720 719	ATATTTCCCTCTAAAATC	IS_a	36.4	39.1	2
LR822013.1	STH_CIRM_32	Yes	C	15 854	863 961 - 879 814	ATATTTCCCTCTAAAATC	IS_a	34.1	39.1	1
LR822014.1	STH_CIRM_36	Yes	C	8 157	732 907 - 724 751	ATATTTCCCTCTAAAATC	IS_a	37.3	39.1	2
LR822015.1	STH_CIRM_65	Yes	C	8 157	737 429 - 729 273	ATATTTCCCTCTAAAATC	IS_a	37.3	39.1	2
LR822017.1	STH_CIRM_336	Yes	C	18 729	52 329 - 71 057	ATTTCATGAAAAAATAC	IS_b	36.0	39.1	1
LR822019.1	STH_CIRM_772	Yes	C	8 193	723 538 - 715 346	ATATTTCCCTCTAAAATC	IS_a	36.4	39.2	2
LR822020.1	STH_CIRM_956 (StCI1)	Yes	C	7 778	1145494 - 1153271	ATATTTCCCTCTAAAATC	IS_a	37.1	38.7	2
LR822020.1	STH_CIRM_956 (StCI2)	Yes	D	10 943	1966248 - 1955306	AATTCTACAACAAAAT	IS_c	33.7	38.7	3
LR822023.1	STH_CIRM_368	Yes	D	12 362	1993695 - 1981334	AATTCTACAACAAAAT	IS_c	34.3	38.8	3
LR822025.1	STH_CIRM_961	No	ND						38.9	

LR822026.1	STH_CIRM_967	No	ND						38.9		
LR822027.1	STH_CIRM_998	Yes	D	10 943	1945003 - 1934061	AATTCTACAACAAAAT	IS_c	33.7	38.9	3	
LR822029.1	STH_CIRM_1035	No	ND						39.1		
LR822030.1	STH_CIRM_1046	No	ND						39.0		
LR822031.1	STH_CIRM_1047	Yes	C	7 724	725 807 - 718 084	ATATTTCCCTCTAAAATC	IS_a	36.3	39.0	2	
LR822032.1	STH_CIRM_1050	No	ND						39.0		
LR822033.1	STH_CIRM_1048	Yes	ND	~19 124	364 401 - 383 524	NA	IS_d	~37.4	39.0	4	
LR822034.1	STH_CIRM_1049	Yes	ND	~19 124	364 401 - 383 524	NA	IS_d	~37.4	39.0	4	
LR822035.1	STH_CIRM_1051	Yes	C	7 853	743 929 - 736 077	ATATTTCCCTCTAAAATC	IS_a	37.1	39.1	2	
LR822036.1	STH_CIRM_1055	Yes	C	9 969	751 454 - 741 486	ATATTTCCCTCTAAAATC	IS_a	35.8	39.1	2	
LR822037.1	STH_CIRM_1121	Yes	C	~18 616	366 946 - 385 561	NA	IS_d	~38.4	39.2	4	
LR822039.1	STH_CIRM_1116	Yes	C	8 157	744 407 - 736 251	ATATTTCCCTCTAAAATC	IS_a	37.3	39.1	2	
LR822040.1	STH_CIRM_1125	No	ND						39.2		
LR822041.1	STH_CIRM_1122	No	ND						39.0		
LR822042.1	STH_CIRM_1358	Yes	C	7 868	731 980 - 724 113	ATATTTCCCTCTAAAATC	IS_a	37.5	39.1	2	
LR822043.1	STH_CIRM_2101	Yes	C	7 754	738 454 - 730 701	ATATTTCCCTCTAAAATC	IS_a	37.4	39.1	2	
LR824002.1	STH_CIRM_67	Yes	C	8 157	1289499 - 1297655	ATATTTCCCTCTAAAATC	IS_a	37.3	39.1	2	
LS483339.1	NCTC12958	Yes	C	9 217	53 505 - 62 721	ATTTCATGAAAAAAATAC	IS_b	36.6	39.0	5	
LS974444.1	N4L	No	ND						39.1		
LT604076.1	ACA-DC 2	No	ND						39.2		
NC_006449.1	CNRZ1066	Yes	C	8 157	736 535 - 728 379	ATATTTCCCTCTAAAATC	IS_a	37.3	39.1	2	Javan602
NZCP011217.1	SMQ-301	Yes	C	11 388	762 273 - 750 886	ATATTTCCCTCTAAAATC	IS_a	36.1	39.1	1	Javan613
NZCP025216.1	DGCC7710	Yes	C	7 795	747 469 - 739 675	ATATTTCCCTCTAAAATC	IS_a	37.5	39.0	2	Javan604
	DGCC688	Yes	D	10 943		AATTCTACAACAAAAT	IS_c	33.7	NA	3	
	DGCC7891	Yes	C	9 969		ATATTTCCCTCTAAAATC	IS_a	35.8	NA	2	
	Abc2	Yes	C			ATATTTCCCTCTAAAATC	IS_a	NA	NA		
	UY03	Yes	C	11 388		ATATTTCCCTCTAAAATC	IS_a	36.1	NA		
	DGCC8234	Yes	C	12 484		ATTCCATGAAAAAAATAC / ATTTCATGAAAAAAATAC	IS_b	36.1	NA	1	
	S4	No[d]	ND						NA		

[a] StCI identified with SatelliteFinder[10,11]. [b] StCI integration site.
[c] StCI identified by Javan *et al.,* 2019[12]. [d] No StCI detected in the insertion site IS_a. [e]Analysis conducted with VirClust[13].

Table S2: *Streptococcus* sp. that have spacers in their CRISPR arrays that target *S. thermophilus* StCIs.

**Strains containing the spacer**					**Targeted StCI**			
**Spacer Sequence (30 pb, 5 ‘-3’)**	**Bacterial Strain**	**GenBank ID**	**StCI [a,b]**	**CRISPR ID, position in the array [c]**	**StCI**	**Mismatch**	**Nucleotides flanking (3’) the spacer**	**StCI targeted region**
ACAAGAAAAATTATTGCCTACGGAACTGTA	*S. thermophilus* TH982	CM003136.1	No[b]	CR3, Spacer 8	KLDS_3_1003	0	TGAAACA	Gene coding for the restriction enzyme of a type I R-M system
					STH_CIRM_1048	0	TGAAACA	
					STH_CIRM_1049	0	TGAAACA	
					STH_CIRM_1121	0	TGAAACA	
					EG007	0	TGAAACA	
ACGGTCTGTATCGAAAAAGACAACTTGGCT	*S. thermophilus* N4L	LS974444.1	No	CR3, Spacer 24	KLDS_3_1003	2	ACGGTGG	Protein Rep
	S. thermophilus MN_BM_A01	CP012588.1	Yes	CR3, Spacer 20	EG007	2	ACGGTGG	
	S. thermophilus MN-ZLW-002	CP003499.1	Yes	CR3, Spacer 20				
TTGATTTTAAAGCTAACGGATCAACACGCT	*S. salivarius* ATCC 25975	CP015283.1			STH_CIRM_956	2	ATAAAGA	Integrase[d]
	S. salivarius NCTC7366	LS483366.1						
CAAAGTCCTCTTCGTATTGATCATAGCTTC	*S. thermophilus* KLDS_3_1003	CP016877.1	Yes	CR1, Spacer 10	KLDS_3_1003	0	AAGAATT	Gene coding for the restriction enzyme of a type I R-M system
					STH_CIRM_1048	0	GGAGCGC	
					STH_CIRM_1049	0	GGAGCGC	
					STH_CIRM_1121	0	GGAGCGC	
					EG007	0	GGAGCGC	
GACAACGAACGAGAGAGTTTATTAGAAGTG	*S. gallolyticus* ICDDRB-NRC-S1	CP013688.1			EPS	0	ATAAAAA	XRE family transcriptional regulator[e]
					TK_P3A	0	ATAAAAA	
					STH_CIRM_30	0	ATAAAAA	
					STH_CIRM_336	0	ATAAAAA	
					STH_CIRM_772	0	ATAAAAA	
TCCATAGAGCGTCTTAAACAAAGAATAGTC	*S. thermophilus* CS8	CP016439.1	Yes	CR1, Spacer 30	13496	0	CGATATA	Non-coding region
	S. thermophilus CNRZ1066	CP000024.1	Yes	CR1, Spacer 30	24739	0	CGATATA	
					DMST	0	CGATAAT	
					LMD-9	0	CGATATA	
					TH-4	0	CGATATA	
					SMQ_301	0	CGATATA	
					ST3	0	CGATATA	
					STH_CIRM_1047	0	CGATATA	
					STH_CIRM_1048	2	CGATAGA	
					STH_CIRM_1049	2	CGATAGA	
					STH_CIRM_1121	2	CGATAAA	
					STH_CIRM_30	0	CGATATA	
					STH_CIRM_772	0	CGATAAT	
					STH_CIRM_956	2	CGATAAT	
GGGAAGTTTGGCGCAAAATCGTTCCGTTTT	*S. macedonicus* 27MV	PEBM01000038.1			CNRZ1066	2	TTTAGAA	Hypothetical protein
					CS8	2	TTTAGAA	
					EPS	2	TTTAGAA	
					STH_CIRM_1358	2	TTTAGAA	
					STH_CIRM_16	2	TTTAGAA	

					STH_CIRM_2101	2	TTTAGAA	
					STH_CIRM_23	2	TTTAGAA	
					STH_CIRM_36	2	TTTAGAA	
					STH_CIRM_65	2	TTTAGAA	
					STH_CIRM_67	2	TTTAGAA	
					STH_CIRM_956SP1	2	TTTAGAA	
					STH-CIRM_1116	2	TTTAGAA	
ATAATCCTTAATCATTTGTTCAAAAGAAAC	*S. thermophilus* TH985	CM003139.1	No[b]	CR3, Spacer 9	13498	0	TGGGGTT	Hypothetical protein[e]
					24853	0	TGGGGTT	
					LMD-9	0	TGGGGTT	
					MAG_rmk202	0	TGGGGTT	
					SMQ_301	0	TGGGGTT	
					ST3	0	TGGGGTT	
					STH_CIRM_18	0	TGGGGTT	
					STH_CIRM_19	0	TGGGGTT	
					STH_CIRM_32	0	TGGGGTT	
					STH_CIRM_336	0	TGGGGTT	
					TH-4	0	TGGGGTT	
CATATCACCCTCATATTCATGTTTTGATGA	*S. thermophilus* SMQ-301	CP011217.1	Yes	CR3, Spacer 1	STH_CIRM_1121	0	TGGTGAG	Hypothetical protein
TCTGACGATAAAGAGAATATCTCAAAGGGT	*S. thermophilus* ST3	CP017064.1	Yes	CR1, Spacer 2	KLDS_3_1003	2	ACAGAAA	Protein Rep
					STH_CIRM_1048	2	ACAGAAA	
					STH_CIRM_1049	2	ACAGAAA	
					EG007	2	ACAGAAA	
GACCTAGGTTACTTGTTGTCAATTAGACCA	*S. agalactiae* 3966RFQB	QLZC01000003.1			KLDS_3_1003	2	TGGAATA	Non-coding region
					STH_CIRM_1048	2	TGGAATA	
					STH_CIRM_1049	2	TGGAATA	
					STH_CIRM_1121	2	TGGAATA	
					EG007	2	TGGAATA	
TTACGCATAATGGTGAATTCATTTTAGTAG	*S. macedonicus* 27MV	PEBM01000038.1			KLDS_3_1003	0	CAGAAGA	Gene coding for the S subunit of a type I R-M system
TTTTGTACAAGGCAAAGGAGTTACTCCAAA	*S. thermophilus* TH982	CM003136.1	No[b]	CR3, Spacer 2	STH_CIRM_1048	0	AGAAGAA	Gene coding for the S subunit of a type I R-M system
					STH_CIRM_1049	0	AGAAGAA	
TTCGCATAAGACTTCTTCAAACCAAAACAT	*S. thermophilus* FAM 13496	VBTK01000005.1	No[b]	CR3, Spacer 4	13499	0	TGGTGTA	Hypothetical protein[e]
					24738	0	TGGTGTA	
					24740	0	TGGTGTA	
					EU01	0	TGGTGTA	
					STH_CIRM_368	0	TGGTGTA	
					STH_CIRM_956SP2	0	TGGTGTA	
					STH_CIRM_998	0	TGGTGTA	

[a] Presence of phage satellite (StCI) in *S. thermophilus* strains. [b] Incomplete genome. [c] *S. thermophilus* strains may carry up to four CRISPR loci. CR1, CRISPR locus 1 associated to a type II-A CRISPR-Cas system. CR3, CRISPR locus 3 associated to a second Type II-A CRISPR-Cas system. [d] The best protospacer matching the spacer is located in *S. salivarus* phage satellite integrase with one mutation (with 100% coverage and 96.67% identity, based on BLAST analysis, accession number CP018186). [e] The protospacer matching the spacer is only found in StCI region (with 100% coverage and 100% identity, based on a BLAST analysis).

Supp. Table 3: Efficiency of plating (EOP) of *S. thermophilus* phages on their (ΔStCI) propagation host containing the pNZ123-RSR and on their corresponding wild-type host (StCI+) containing pNZ123.

			Assay 1		Assay 2			
Strain	Phage	Phage volume	pfu/ml	EOP	pfu/ml	EOP	Mean EOP	±SD
DGCC7710 + pNZ123	2972	10 µl	2.20E+08	1.14	2.00E+08	2.00	1.57	0.61
DGCC7710-ΔStCI + pNZ123-RSR	2972	10 µl	2.50E+08		4.00E+08			
DGCC7710 + pNZ123	D4752	10 µl	2.00E+09	1.50	2.30E+09	0.70	1.10	0.57
DGCC7710-ΔStCI + pNZ123-RSR	D4752	10 µl	3.00E+09		1.60E+09			
UY03 + pNZ123	73	10 µl	4.20E+05	0.29	4.00E+06	1.00	0.64	0.25
UY03-ΔStCI + pNZ123-RSR	73	10 µl	1.20E+05		4.00E+06			
UY03 + pNZ123	DT1	10 µl	2.00E+06	1.50	8.00E+05	4.88	3.19	2.39
UY03-ΔStCI + pNZ123-RSR	DT1	10 µl	3.00E+06		3.90E+06			
SMQ-301 + pNZ123	73	10 µl	1.10E+09	0.18	1.20E+09	0.67	0.42	0.17
SMQ-301-ΔStCI + pNZ123-RSR	73	10 µl	2.00E+08		8.00E+08			
SMQ-301 + pNZ123	DT1	10 µl	3.00E+08	2.00	2.50E+08	1.44	1.72	0.40
SMQ-301-ΔStCI + pNZ123-RSR	DT1	10 µl	6.00E+08		3.60E+08			
DGCC7891 + pNZ123	P738	5 µl	1.00E+08	2.80	3.60E+08	1.72	2.26	0.38
DGCC7891-ΔStCI + pNZ123-RSR	P738	5 µl	2.80E+08		6.20E+08			
DGCC7891 + pNZ123	D4446	5 µl	6.00E+07	1.33	8.00E+07	3.00	2.17	1.18
DGCC7891-ΔStCI + pNZ123-RSR	D4446	5 µl	8.00E+07		2.40E+08			

EOP values were calculated using this formula: Phage titer (ΔStCI host + pNZ123-RSR) / Phage titer (wild-type host + pNZ123).
Mean EOP is the average of the EOP values obtained from two assays ± standard deviation (SD).

Supp. Table 4: Significant p-values (< 0.05) from the Tukey multiple pairwise-comparison test.

Supp. Figure number	Strain	Target	Comparison 1	Comparison 2	p-value
Supp. Figure 7	SMQ-301	ΔStCI	0.8	0.2	2E-03
			ON	0.2	6E-03
		Excised StCI	0.8	0.2	2E-02
Supp. Figure 8	DGCC7710		Excised StCI	ΔStCI	8E-07
			Integrated StCI	ΔStCI	2E-09
			Primase	ΔStCI	2E-09
			Integrated StCI	Excised StCI	7E-08
			Primase	Excised StCI	6E-08
	SMQ-301		Excised StCI	ΔStCI	1E-10
			Integrated StCI	ΔStCI	5E-14
			Primase	ΔStCI	5E-14
			Integrated StCI	Excised StCI	7E-10
			Primase	Excised StCI	4E-10
	DGCC7710::StCI-301-CAT		Excised StCI	ΔStCI	2E-06
	(SMQ-1512)		Integrated StCI	ΔStCI	6E-14
			Primase	ΔStCI	6E-14
			Integrated StCI	Excised StCI	3E-12
Supp. Figure 18 Panel B	SMQ-1528	Excised StCI	2972 wt T30	2972-Orf33-mut T10	4E-04
			2972 wt T30	2972-Orf33-mut T20	4E-04
			2972 wt T30	2972-Orf33-mut T30	3E-04
			2972 wt T30	Non-infected T0	5E-04
			2972 wt T30	2972 wt T10	6E-04
Supp. Figure 18 Panel C	SMQ-1528	Excised StCI	2972 wt T30	2972-Orf33-mut T10	3E-04
			2972 wt T30	2972-Orf33-mut T20	3E-04
			2972 wt T30	2972-Orf33-mut T30	3E-04
			2972 wt T30	Non-infected T0	4E-04
			2972 wt T30	2972 wt T10	5E-04
		Primase	Non-infected T0	2972-Orf33-mut T10	3E-02
			2972 wt T10	Non-infected T0	2E-02
			2972 wt T20	Non-infected T0	3E-03
			2972 wt T30	Non-infected T0	4E-02

A one-way Anova analysis was initially conducted on the different samples/targets.

Table S5: Mean cq values obtained in the qPCR experiments using phages (Figure 8 and Supp. Figure 17). The *gyrA* and *gyrB* targets are located on the corresponding reference genes, the *pri* target is located in the StCI *primase* gene, and the excised StCI target is located on the excised StCI including its *attS* site.

		**Biological assay #1**				**Biological assay #2**				**Biological assay #3**			
**Strain**	**Condition**	***gyrA***	***gyrB***	***pri***	**Excised StCI**	***gyrA***	***gyrB***	***pri***	**Excised StCI**	***gyrA***	***gyrB***	***pri***	**Excised StCI**
SMQ-1528	Non-infected_T0	20.23	20.48	20.84	28.90	21.29	21.36	21.74	29.74	20.30	20.46	21.00	28.28
SMQ-1528	2972-Mutant_T10	19.44	19.43	19.68	27.67	19.83	19.96	20.18	28.29	19.88	19.92	20.23	28.39
SMQ-1528	2972-Mutant_T20	19.85	19.72	20.04	28.12	19.76	19.71	20.10	28.14	19.17	19.18	19.48	27.62
SMQ-1528	2972-Mutant_T30	20.97	20.87	21.21	29.33	20.92	20.80	21.20	29.17	20.20	20.13	20.63	28.68
SMQ-1528	2972-Wildtype_T10	16.96	17.23	17.36	25.78	20.57	20.81	20.93	28.48	19.18	19.40	19.55	27.57
SMQ-1528	2972-Wildtype_T20	19.98	20.19	20.30	28.15	20.09	20.29	20.47	27.06	22.07	22.24	22.36	28.30
SMQ-1528	2972-Wildtype_T30	16.13	16.45	16.47	22.93	19.83	20.06	20.26	24.95	17.64	17.87	18.07	22.54
SMQ-1512	Non-infected_T10	17.40	17.61	18.03	28.91	20.57	20.64	21.01	30.21				
SMQ-1512	2972-Mutant_T10	17.23	17.41	17.58	27.52	17.46	17.59	17.76	27.18				
SMQ-1512	2972-Mutant_T20	16.47	16.61	16.83	26.50	16.62	16.74	16.95	26.12				
SMQ-1512	2972-Mutant_T30	16.51	16.66	16.99	26.36	16.43	16.51	16.76	26.12				
SMQ-1512	2972-Wildtype_T10	18.36	18.78	18.85	22.13	18.10	18.35	18.36	24.04				
SMQ-1512	2972-Wildtype_T20	15.86	16.20	13.30	13.71	17.20	17.42	15.04	15.53				
SMQ-1512	2972-Wildtype_T30	16.29	16.56	11.91	11.98	17.42	17.68	13.18	13.26				

The values represent the average cq of technical replicates.

Table S6: Description and features of the bacterial strains, phages, plasmids, and primers used in this study.

**Strain name**[a]	**Reference number**	**Source / Accession Number**	**Description**[a]
E. coli			
BL21 CodonPlus (DE3)-RIPL		Agilent	F^{-} *ompT hsdS*(r_B^{-} m_B^{-}) dcm^{+} Tet^{r} gal λ(DE3) *endA* Hte [*argU proL* Cam^{r}] [*argU ileY leuW* Strep/Spec^{r}]
BL21-CodonPlus (DE3)-RIPL + pET-28a-StCI-301-Stl	SMQ-1715	This study	BL21-CodonPlus (DE3)-RIPL containing pET-28a-StCI-301-Stl
BL21 (DE3) pLysS		Stratagene	F^{-} *ompT hsdSB* (r_B^{-} m_B^{-}) *dcm^{+} gal* (DE3) pLysS (Cam^{r}) Chemically competent cells for protein expression
BL21 (DE3) pLysS + pDEST14-Orf33	SMQ-1713	This study	BL21 (DE3) pLysS containing pDEST14-Orf33
DH5a		New England Biolabs	NEB 5-alpha chemically competent cells
DH5a + pDEST14-Orf33	SMQ-1712	This study	DH5a with pDEST14-Orf33
DH5a + pDONR-Orf33	SMQ-1711	This study	DH5a with pDONR-Orf33
DH5a + pET-28a-StCI-301-Stl	SMQ-1714	This study	DH5a with pET-28a-StCI-301-Stl
DH5a + pNZ123-RSR-NC	SMQ-1507	This study	DH5a with pNZ123-RSR-NC
DH5a + pNZ123-ERY-RSR	SMQ-1530	This study	DH5a with pNZ123-ERY-RSR
DH5a + pNZ123-ERY-Orf33-wt	SMQ-1697	This study	DH5a with pNZ123-ERY-Orf33-wt (from phage 2972)
DH5a + pNZ123-ERY-Orf33-mut	SMQ-1698	This study	DH5a with pNZ123-ERY-Orf33-mut (from phage 2972-Orf33-mut)
DH5a + pNZ123-AcrIIA5	SMQ-1346	14	DH5a with pNZ123-AcrIIA5
DH5a + pNZ123-RSR	SMQ-1506	This study	DH5a with pNZ123-RSR
Lactococcus lactis			
MG1363	SMQ-236	AM406671.1	Strain used for sub-cloning
MG1363 + pNZ123-ERY	SMQ-1508	This study	MG1365 with pNZ123-ERY plasmid

Streptococcus thermophilus			
Abc2	HER1479	15	Wild-type strain
ATCC 19258	SMQ-515	CP038020.1	Wild-type strain
DGCC688	SMQ-1561	This study	Wild-type strain
DGCC8234	SMQ-1560	16	Wild-type strain
LMD-9	HER1484	CP000419	Wild-type strain
DGCC7710	SMQ-692	NZ_CP025216	Wild-type strain, host of phage 2972
DGCC7710-ΔStCI-ΔRSR	SMQ-1509	This study	DGCC7710 without StCI and pNZ123-RSR
DGCC7710 + pNZ123	SMQ-1339	17	SMQ-692 with pNZ123
DGCC7710 + pNZ123-RSR	SMQ-1511	This study	DGCC7710 with pNZ123-RSR
DGCC7710::StCI-301-CAT	SMQ-1512	This study	DGCC7710 with StCI-CAT of SMQ-301 (StCI-301-CAT)
DGCC7710-ΔStCI-ΔRSR no-DNA-control	SMQ-1685	This study	SMQ-1509 no-DNA control in the same natural transformation experiment than SMQ-1512
DGCC7710::StCI-301-CAT-rev	SMQ-1513	This study	DGCC7710 with StCI-CAT-rev of SMQ-301 (StCI-301-CAT-rev)
DGCC7710-ΔStCI-ΔRSR no-DNA control	SMQ-1686	This study	SMQ-1509 no-DNA control in the same natural transformation experiment than SMQ-1513
DGCC7710-ΔStCI-ΔRSR + pNZ123-AcrIIA5	SMQ-1919	This study	SMQ-1509 + pNZ123-AcrIIA5
DGCC7710::StCI-301-CAT + pNZ123-ERY	SMQ-1514	This study	DGCC7710 with StCI-301-CAT + pNZ123-ERY
DGCC7710::StCI-301-CAT + pNZ123-ERY-RSR	SMQ-1531	This study	DGCC7710 with StCI-301-CAT + pNZ123-ERY-RSR
DGCC7710::StCI-301-CAT + pNZ123-ERY-Orf33-mut	SMQ-1515	This study	DGCC7710 with StCI-301-CAT + pNZ123-ERY-Orf33-mut
DGCC7710::StCI-301-CAT + pNZ123-ERY-Orf33-wt	SMQ-1516	This study	DGCC7710 with the StCI-301-CAT + pNZ123-ERY-Orf33-wt

DGCC7710::StCI-301 regulatory *orfs*	SMQ-1528	This study	DGCC7710 with the StCI-301 regulatory *orfs* in its native StCI (swap)
DGCC7710::StCI-301 regulatory *orfs* + pNZ123-ERY	SMQ-1691	This study	SMQ-1528 containing pNZ123-ERY
DGCC7710::StCI-301 regulatory *orfs* + pNZ123-ERY-Orf33-wt	SMQ-1692	This study	SMQ-1528 with pNZ123-ERY-Orf33-wt
DGCC7710::StCI-301 regulatory *orfs* + pNZ123-ERY-Orf33-mut	SMQ-1693	This study	SMQ-1528 with pNZ123-ERY-Orf33-mut
DGCC7710 no-DNA control	SMQ-1529	This study	DGCC7710 no-DNA control in the same natural transformation experiment than SMQ-1528
DGCC7710 no-DNA control + pNZ123-ERY	SMQ-1694	This study	SMQ-1529 with pNZ123-ERY
DGCC7710 no-DNA control + pNZ123-ERY-Orf33-wt	SMQ-1695	This study	SMQ-1529 with pNZ123-ERY-Orf33-wt
DGCC7710 no-DNA control + pNZ123-ERY-Orf33-mut	SMQ-1696	This study	SMQ-1529 with pNZ123-ERY-Orf33-mut
DGCC7891	SMQ-1358	18	Wild-type strain
DGCC7891 + pNZ123-RSR-NC	SMQ-1517	This study	DGCC7891 with pNZ123-RSR-NC
DGCC7891-ΔStCI-ΔRSR	SMQ-1518	This study	DGCC7891 without its StCI and the pNZ123-RSR-NC
DGCC7891::StCI-301-CAT-rev	SMQ-1519	This study	DGCC7891 with StCI-CAT-rev of SMQ-301
DGCC7891-ΔStCI-ΔRSR no-DNA control	SMQ-1687	This study	SMQ-1518 no-DNA control in the same natural transformation experiment than SMQ-1519
SMQ-301	HER1368	NZ_CP011217	Wild-type strain
SMQ-301 + pNZ123-RSR	SMQ-1523	This study	SMQ-301 with pNZ123-RSR
SMQ-301::StCI-CAT	SMQ-1521	This study	SMQ-301 with *cat* gene added in its StCI
SMQ-301::StCI-CAT-rev	SMQ-1522	This study	SMQ-301 with *cat* gene in reverse orientation added in its StCI

SMQ-301-ΔStCI-ΔRSR	SMQ-1520	This study	SMQ-301 without its StCI and the pNZ123-RSR
S4	SMQ-1562	19	Wild-type strain
S4::StCI-301-CAT-rev	SMQ-1524	This study	S4 with the StCI-CAT-rev of SMQ-301
S4 no-DNA control	SMQ-1688	This study	SMQ-1562 no-DNA control in the same natural transformation experiment than SMQ-1524
UY03	SMQ-1208	20	Wild-type strain
UY03 + pNZ123	SMQ-1689	This study	SMQ-1208 with pNZ123
UY03 + pNZ123-RSR	SMQ-1526	This study	UY03 with pNZ123-RSR
UY03::StCI-301-CAT-rev	SMQ-1527	This study	UY03 containing the StCI-CAT-rev of SMQ-301
UY03-ΔStCI-ΔRSR	SMQ-1525	This study	UY03 without its StCI and the pNZ123-RSR
UY03-ΔStCI-ΔRSR no-DNA control	SMQ-1690	This study	SMQ-1525 no-DNA control in the same natural transformation experiment than SMQ-1527

Table S7: Description and features of phages used in this study.

Phages	Description	Source / Accession number
2972	*Brüssowvirus*, virulent phage infecting DGCC7710	21 / NC_007019.1
2972-Orf33-mut	Phage 2972 with a frameshift mutation in *orf33*	This study
73	*Moineauvirus*, virulent phage infecting *S.t.* SMQ-301 and UY03	20
D1126	*Brüssowvirus*, virulent phage infecting *S.t.* DGCC7710	14
D4446	Virulent phage infecting *S.t.* DGCC7891	19
D4752	*Brüssowvirus*, virulent phage infecting *S.t.* DGCC7710	22
DT1	*Moineauvirus*, virulent phage infecting *S.t.* SMQ-301 and UY03	23
P738	Virulent phage infecting *S.t.* DGCC7891	19

Table S8: Description and features of plasmids used in this study.

Plasmids	Description	Source
pDEST14	Native expression vector, ampicillin resistance gene	Invitrogen
pDEST14-Orf33	pDEST14 + Orf33 from phage 2972	This study
pDONR201	Native entry vector, kanamycin resistance gene	Invitrogen
pDONR-Orf33	pDONR201 + Orf33 from phage 2972	This study
pET-28a	Native vector, kanamycin resistance gene	Novagen
pET-28a-StCI-301-Stl	pET28a + StCI-301-Stl	This study
pTRKH2	Native vector, erythromycin resistance gene	24
pNZ123 wt	Native vector, chloramphenicol resistance gene, negative control	25
pNZ123-AcrIIA5	pNZ123 + AcrIIA5 from phage D1126 (MZ090947.1) inserted in XbaI site (pNZAcr-1126)	14
pNZ123-RSR-int	pNZ123 + crRNA targeting the StCI integrase in SMQ-301, DGCC7710 and UY03	This study
pNZ123-RSR-NC	pNZ123 + crRNA targeting a non-coding region in StCI of DGCC7891	This study
pNZ123-ERY	pNZ123 in which the *cat* gene was swapped for the *ery* gene from pTRKH2	This study
pNZ123-ERY-RSR	pNZ123-ERY + crRNA targeting the StCI integrase in SMQ-301, DGCC7710, and UY03	This study
pNZ123-ERY-Orf33-wt	pNZ123-ERY containing the *orf33* (YP_238516.1) of phage 2972	This study
pNZ123-ERY-Orf33-mut	pNZ123-ERY containing a mutated (frameshift) *orf33* of phage 2972	This study

Table S9: Description and features of primers used in this study.

Primer Name	Sequence (5'-3')	Function	Source
2972_orf33_cloning_F	ATTACAGCTCCAGATCCAGTACTGAA TTCTCAACAATTAGGGAGGGTAGG	pNZ123-ERY-Orf33-wt and pNZ123-ERY-Orf33-mut	This study
2972_orf33_cloning_R	GAAAATATGCACTCGAGAAGCTTGAG CTCTCGTTGCTTTGGTAATTTTCATC	pNZ123-ERY-Orf33-wt and pNZ123-ERY-Orf33-mut	This study
Ampli_g_block_reg_301_F	CATGGTTACGGTTTGCTTATC	Amplification of StCI-301 regulatory *orfs* with DGCC7710-StCI homologous sequences	This study
Ampli_g_block_reg_301_F	CAAACACACTTTCTAACTTCTTG	Amplification of StCI-301 regulatory *orfs* with DGCC7710-StCI homologous sequences	This study
AttL-F	ATATTTCCCTCTAAAATCCTTTAATTTT	Whole StCI amplification	This study
Abi-F	CCTTATTAGTTAATTTTTTCCTTCG	Detection of the excised DGCC7891-StCI	This study
Arg_F	CTGCAATGAGTTCTTTAGTATTC	Detection of the excised/integrated StCIs in DGCC8234/ATCC19258	This study
ArgR_R	CAGCTTGACGATCTCTCTTC	Detection of the excised/integrated StCIs in DGCC8234/ATCC19258	This study
Bras-R-R	AGGAAAAAGGGCTTGGAAGATG	Detection of integrated StCIs	This study
CAT-circ-F	GAATTGTCAGATAGGCCTAATG	Detection of the excised/integrated StCI-301-CAT	
CAT-circ-rev-F	CCAATTGTCTAAATCAATTTTATTAA AG	Detection of the excised/integrated StCI-301-CAT-rev	This study
CR1-DGCC7891-R	GAGACACAGGAGTAGGAAAG	CR1 locus screening (DGCC7891)	This study
CR1-rev	TAAACAGAGCCTCCCTATCC	CR1 locus screening	26
Delta_SP_qPCR_F1	CGCTTGGAAGTCACTTTTTTCAAT	Amplification of empty *attC* site / qPCR exp.	This study
Delta_SP_qPCR_R1	ACCAAAAATGAACTTTTCAGATAGTTTT	Amplification of empty *attC* site / qPCR exp.	This study
DGCC688_SP_circ_F	GATTTATTATTTGGTAAAAAACAATAGG	Detection of the excised DGCC688 StCI	This study
DGCC688_SP_circ_R	GGCAGTTAATTCACTATAAACTC	Detection of the excised DGCC688 StCI	This study
DGCC7710_circ_F	CTATTCTAAGGGTTGTAGTGAAG	Detection of the excised DGCC7710 StCI	This study
DGCC7710_SP_circ_F5	CACGCGAAATATTGAGCTTAG	Detection of the excised DGCC7710 StCI (semi-quantitative PCR)	This study
DGCC8234_circ_F	CTATATCAATCCACTTCCCTTAG	Detection of the excised DGCC8234 StCI	This study
DGCC8234_circ_R	GCAAGCACTAAAATCTATCTAAAAG	Detection of the excised DGCC8234 StCI	This study
Ery_pTRKH2_F	TGAGAGGAGGCATATCAAATGAACTT TAATTAAGCTAAAAATTTGTAATTAA GAAGGAG	Amplification of *ery*^{a} gene from pTRKH2 / pNZ123-ERY	This study
Ery_pTRKH2_R	TGTAAAAAGTACAGTCGGCATTATCTCAT ATTATTTCCTCCCGTTAAATAATAG	Amplification of *ery*^{a} gene from pTRKH2 / pNZ123-ERy	This study
GyrA_qPCR_F1	AGCCATGCGTAAACTTTCTG	Amplification of the *S.t. gyrA* / qPCR exp.	This study
GyrA_qPCR_R1	GTCCACCTGGGGTAGTCTCT	Amplification of the *S.t. gyrA* / qPCR exp.	This study

GyrB_qPCR_F1	GACATAGCCAGCTTCCAAGA	Amplification of the *S.t. gyrB* / qPCR exp.	This study
GyrB_qPCR_R1	GCTCGCTATCACAAGTTGGT	Amplification of the *S.t. gyrB* / qPCR exp.	This study
HP_DGCC688_R	CAGACTTTGAAGGTGGACTG	Detection of the excised/integrated DGCC688 StCI	This study
Orf33_pDEST14F	GGGGACAAGTTTGTACAAAAAAGCAGGCTGAAGGAGATAGAACCATGGCAACTTTATATGAGTTAACAGGT	Amplification of *orf33* from phage 2972 for cloning in pDEST14	This study
Orf33_pDEST14R	GGGGACCACTTTGTACAAGAAAGCTGGGTTTATTAATGGTGATGGTGATGGTGTCTTATGTTAAGGTTTCTCCTTTCTTCA	Amplification of *orf33* from phage 2972 for cloning in pDEST14	This study
pDEST14_F	CAACGGTTTCCCTCTAGATC	pDEST14 insert screening	This study
pDEST14_R	GTTAGCAGCCGGATCATC	pDEST14 insert screening	This study
pET28a_Fw	CACCACCACCACCACCACTGA	Amplification of linear pET28a	27
pET28a_rv	CATGGTATATCTCCTTCTTA	Amplification of linear pET28a	27
PICI-ampli_R2	CCCCTACTATTTCATAAGTTTAATTAGAGTG	Whole StCI amplification	This study
pNZ_ERY_F	AGTTATCTATTATTTAACGGGAGGAAATAATATGAGATAATGCCGACTGTAC	Amplification linear pNZ123-ERY	This study
pNZ_ERY_R	ACTCCTTCTTAATTACAAATTTTTAGCTTAATTAAAGTTCATTTGATATGCCTCC	Amplification linear pNZ123-ERY	This study
pNZins_F	AATGTCACTAACCTGCCCCG	pNZ123 insert screening	28
pNZins_R	CATTGAACATGCTGAAGAGC	pNZ123 insert screening	28
pNZ_XbaI_F	AGAGCTCAAGCTTCTCGAG	Amplification of linear pNZ123	28
pNZ_XbaI_R	AGAATTCAGTACTGGATCTGGAGC	Amplification of linear pNZ123	28
Primase_qPCR_F2	CGCGGACGAGTTCAATGTTT	Amplification of the StCI primase / qPCR exp.	This study
Primase_qPCR_R2	AGCACTTTCTAGCGGTCTGC	Amplification of the StCI primase / qPCR exp.	This study
RDS7rev	GGATCCGGATCCGTTGAGGCCTTGTTC	CR1 locus screening (DGCC7710)	26
Regulateur_PS-301_pet28a_F	AATTTTGTTTAACTTTAAGAAGGAGATATACCATGAGTGTAATAGGGAACTTTTTAAAAGAA	Detection of StCI-301 regulatory *orfs*	This study
Regulateur_PS-301_pet28a_R	TTAGCAGCCGGATCTCAGTGGTGGTGGTGGTGGTGTTTACTATATCTTATTCCGTTTAACGTA	Detection of StCI-301 regulatory *orfs*	This study
Regulateur_PS_DG_F	ATTACAGCTCCAGATCCAGTACTGAATTCTCAGGCAAGCCATATTATATCG	Amplification of the DGCC7710 StCI regulatory *orfs* region	This study
Regulateur_PS_DG_R	GAAAATATGCACTCGAGAAGCTTGAGCTCTGTAAGTTTGGTGACTGCGAA	Amplification of the DGCC7710 StCI regulatory *orfs* region	This study
RpsD_F	GTCCAGCATTCGTATCATTTG	Detection of the excised/integrated DGCC688 StCI	This study
SMQ_F	GTAGTAAATTTTAATTCTCGGAATTC	Detection of the excised/integrated StCIs	This study

SMQ_R	CGTGAGTGAAGATTTGTCTGAC	Detection of the excised/integrated StCIs	This study
SP_301_ampli_F	CTTTAATTTTAAAAAATGTTTGTAAAAACCCCG	Whole StCI amplification (excluding *attL* site)	This study
SP_301_ampli_R	GATTTTAGAGGGAAATATCCCCTACTATTTC	Whole StCI amplification (including *attR* site)	This study
SP_circ_F	GACTACGTTTTAGACGGTAAATG	Detection of the excised/integrated SMQ-301 StCI	This study
SP_circ_R	GTGGCTCTAAAATCCATATAAAAG	Detection of the excised/integrated StCIs	This study
SP_F	CCCTTATCGCCTTCTATAATG	Amplification of the StCI-301-CAT/ StCI-301-CAT-rev	This study
SP_R	GGTCAGTTTCAGTTGTCAAGC	Amplification of the StCI-301-CAT/ StCI-301-CAT-rev	This study
SP_excised_qPCR_F1	GCTCTCAAAAATCGCTTTGTCATCA	Amplification of the excised StCI / qPCR exp.	This study
SP_excised_qPCR_R1	AAGAAAAAGCCCATAACAACGGG	Amplification of the excised StCI / qPCR exp.	This study
SP_excised_qPCR_R2	AAAGCCCATAACAACGGGGT	Amplification of integrated StCI / qPCR exp.	This study
XbaI-R_Sint_R-for	TCTAGATCTAGAGTTTTTGTACTCTCAAGATTTAAGTAACTGTACAACATTTAGAGTTGTCTAAACTATCAAGGTAGGGTTTTTG	pNZ123-RSR-int construction	This study
XbaI-R_Sint_R-rev	TCTAGATCTAGAGTTGTACAGTTACTTAAATCTTGAGAGTACAAAAACCCTACCTTGATAGTTTAGACAACTCTAAATGTTG	pNZ123-RSR-int construction	This study
XbaI-R_SNC_R_DGCC7891-F	TCTAGATCTAGAGTTTTTGTACTCTCAAGATTTAAGTAACTGTACAACAGACAGCAAAAAAAAGCCACTGATTATCAGGTTTTTG	pNZ123-RSR-NC construction	This study
XbaI-R_SNC_R_DGCC7891-R	TCTAGATCTAGAGTTGTACAGTTACTTAAATCTTGAGAGTACAAAAACCTGATAATCAGTGGCTTTTTTTTGCTGTCTGTTG	pNZ123-RSR-NC construction	This study
Regulateur_PS_301_pet28a_F	AATTTTGTTTAACTTTAAGAAGGAGATATACCATGAGTGTAATAGGGAACTTTTTAAAAGAA	Cloning of the pET-28a-StCI-301-Stl	This study
Regulateur_PS_301_pet28a_R	TTAGCAGCCGGATCTCAGTGGTGGTGGTGGTGGTGTTTACTATATCTTATTCCGTTTAACGTA	Cloning of the pET-28a-StCI-301-Stl	This study
T7 Promoter Primer	TAATACGACTCACTATAGGG	pET-28a insert screening	Novagen
T7 Terminator Primer	GCTAGTTATTGCTCAGCGG	pET-28a insert screening	Novagen
Yc70	TGCTGAGACAACCTAGTCTCTC	CR1 locus screening	26

[a] CAT, chloramphenicol acetyltransferase; Ery, erythromycin.

Table S10: Details of reagents and DNA used in natural competence assays to mobilize StCIs to *S. thermophilus* strains.

	Phosphorylation					Ligation			Transformation		PCR verification
**StCI**	**PCR Product (µg/µl)**	**PNK (µl)**	**Buffer (µl)**	**Final Volume (µl)**	**Incubation time, room temp. (min)**	**T4 (µl)**	**Buffer (µl)**	**Final volume (µl)**	**Stra**	**DNA (µg ligation / µg linear PCR product)**	**Primers**
StCI-301-CAT-rev	4 / 18.27	2	4	24.3	120	2	8	34.3	UY03-ΔStCI-ΔRSR	4 / 4	Yc70/CR1-rev; SMQ-F/SP-circ-R; CM5-circ-rev-F/ SMQ-R
	4 / 18.27	2	4	24.3	120	2	8	34.3	DGCC7891-ΔStCI-ΔRSR	4 / 4	Yc70/ CR1-DGCC7891-R; SMQ-F/SP-circ-R; CM5-circ-rev-F/ SMQ-R
	4 / 18.27	2	4	24.3	120	2	8	34.3	DGCC7710-ΔStCI-ΔRSR	4 / 4	Yc70/RDS7rev; SMQ-F/SP-circ-R; CM5-circ-rev-F/ SMQ-R
	2 / 17.06	2	4	40	45	2	8	50	S4	2 / 2	Yc70/CR1-rev; SMQ-F/SP-circ-R; CM5-circ-rev-F/SMQ-R; SMQ-F/SMQ-R
StCI-301-CAT	12 / 40	6	12	58	45	6	24	88	DGCC7710-ΔStCI-ΔRSR	12 µg ligation	Yc70/RDS7rev; CM5-circ-F/SMQ-R; SMQ-F/R; CM5-circ-F/SP-circ-R
StCI-301 wt	12 / 24.9	6	12	42.9	45	6	24	72.9	DGCC7710-ΔStCI-ΔRSR + pNZ123-AcrIIA5	12 µg ligation	Yc70/RDS7rev; SP-circ-F/SMQ-R; SMQ-F/SMQ-R

References

1. Moraru, C. VirClust - A tool for hierarchical clustering, core protein detection and annotation of (prokaryotic) viruses. *Viruses*. **15**, 1007 (2023).
2. R Core Team, R: A language and environment for statistical computing. R Foundation for Statistical Computing, Vienna, Austria. Available at: https://www.R-project.org/ (2020).
3. Guy, L., Roat Kultima, J., & Andersson, S.G.E. genoPlotR: comparative gene and genome visualization in R. *Bioinformatics* **26**, 2334-2335 (2010).
4. Taylor, S. C., Nadeau, K., Abbasi, M., Lachance, C., Nguyen, M., & Fenrich, J. The ultimate qPCR experiment: Producing publication quality, reproducible data the first time. *Trends Biotechnol*. **37**, 761-774 (2019).
5. Livak, K. J. & Schmittgen, T. D. Analysis of relative gene expression data using real-time quantitative PCR and the 2(-Delta Delta C(T)) method. *Methods* **25**, 402–408 (2001).
6. Geneious 11.1.5, Available at: https://www.geneious.com
7. Skinner, S. O., Sepulveda, L. A., Xu, H., & Golding, I. Measuring mRNA copy number in individual *Escherichia coli* cells using single-molecule fluorescent in situ hybridization. *Nat. Protoc.* **8**, 1100-1113 (2013).
8. Schindelin, J. et al. Fiji: an open-source platform for biological-image analysis. *Nat. Methods* **9**, 676–682 (2012).
9. Fontaine, L. et al. A novel pheromone quorum-sensing system controls the development of natural competence in *Streptococcus thermophilus* and *Streptococcus salivarius*. *J. Bacteriol*. **192**, 1444-1454 (2010).
10. De Sousa, J. et al. Identification and characterization of thousands of bacteriophage satellites across bacteria. *Nucleic Acids Res*. **51**, 2759-2777 (2023).
11. Néron, B. et al. MacSyFinder v2: Improved modelling and search engine to identify molecular systems in genomes. *Peer J*. **3**, e28 (2023).
12. Javan, R. R. et al. Prophages and satellite prophages are widespread in *Streptococcus* and may play a role in pneumococcal pathogenesis. *Nat. Commun*. **10**, 4852 (2019).
13. Moraru, C. VirClust - A tool for hierarchical clustering, core protein detection and annotation of (prokaryotic) viruses. *Viruses*. **15**, 1007 (2023).
14. Hynes, A. P. et al. Widespread anti-CRISPR proteins in virulent bacteriophages inhibit a range of Cas9 proteins. *Nat. Commun*. **9**, 2919 (2018).
15. Suárez, V. B. et al. Thermophilic lactic acid bacteria phages isolated from Argentinian dairy industries. *J. Food Prot.* **65**, 1597-1604 (2002).
16. Barrangou, R. et al. CRISPR provides acquired resistance against viruses in prokaryotes. *Science* **315**, 1709-1712 (2007).
17. Hynes et al. Programming native CRISPR arrays for the generation of targeted immunity. *mBio*. **7**, e00202-16 (2016).
18. Dandoy, D. et al. The fast milk acidifying phenotype of *Streptococcus thermophilus* can be acquired by natural transformation of the genomic island encoding the cell-envelope proteinase PrtS. *Microb. Cell. Fact*. **10**, S21 (2011).
19. Philippe, C. et al. Novel genus of phages infecting *Streptococcus thermophilus*: Genomic and morphological characterization. *Appl. Environ. Microbiol.* **86**, e00227-20 (2020).
20. Achigar, R. et al. Phage-host interactions in *Streptococcus thermophilus*: Genome analysis of phages isolated in Uruguay and ectopic spacer acquisition in CRISPR array. *Sci. Rep.* **7**, 43438 (2017).
21. Lévesque, C. et al. Genomic organization and molecular analysis of virulent bacteriophage 2972 infecting an exopolysaccharide-producing *Streptococcus thermophilus* strain. Appl. Environ. Microbiol. **71**, 4057-4068 (2005).
22. Labrie, S. J. et al., A mutation in the methionine aminopeptidase gene provides phage resistance in *Streptococcus thermophilus*. *Sci. Rep.* **9**, 13816 (2019).
23. Tremblay, D. M. & Moineau, S. Complete genomic sequence of the lytic bacteriophage DT1 of *Streptococcus thermophilus*. *Virology* **255**, 63-76 (1999).
24. O'Sullivan, D. J. & Klaenhammer, T. R. High- and low-copy-number *Lactococcus* shuttle cloning vectors with features for clone screening. *Gene* **137**, 227-231 (1993).
25. De Vos, W. M. & Simons, G. F. M. “Gene cloning and expression systems in Lactococci.” In M. J. Gasson & W. M. De Vos (Eds.), *Genetics and Biotechnology of Lactic Acid Bacteria* (pp. 52-105), Springer (1994).
26. Horvath, P. et al. Diversity, activity, and evolution of CRISPR loci in *Streptococcus thermophilus*. J. Bacteriol. **190**, 1401–1412 (2008).
27. Oechslin et al. Fermentation practices select for thermostable endolysins in phages. *Mol. Biol. Evol*. **41**, msae055 (2024).
28. Lemay, M.-L., et al., S. Targeted genome editing of virulent phages using CRISPR-Cas9. *Bio-Protocol*. **8**, e2674 (2018).
