# Supplementary material for: Phage satellites induced by virulent phages are mobilized by natural competence leading to phage resistance in a new host
Source: Nat Commun. 2026 May 12;17:6528. doi: 10.1038/s41467-026-72928-1 (PMC13376191; doi:10.1038/s41467-026-72928-1)
Supplement: Supplementary file 2 — Peer Review file [file 41467_2026_72928_MOESM2_ESM.pdf]

nature portfolio

Peer Review File

Phage satellites induced by virulent phages are mobilized by natural competence leading to phage resistance in a new host

Corresponding Author: Professor Sylvain Moineau

This file contains all reviewer reports in order by version, followed by all author rebuttals in order by version.

Attachments originally included by the reviewers as part of their assessment can be found at the end of this file.

Version 0:

Reviewer comments:

Reviewer #1

(Remarks to the Author)
The manuscript “Satellite prophages induced by virulent phages are mobilized by natural competence leading to phage resistance in a new host” by Merency et al. describes the discovery of mobilizable phage satellite islands in Streptococcus thermophilus. The manuscript is in most parts well-written and shows some promising results. The authors have done a substantial amount of work, with overall reasonable controls, and they could potentially be up to a significant discovery. My main concern is that some of the authors results seem to contradict each other and are inconsistent with their claims.

Major concerns:
The authors use expression of orf33 from a plasmid in a strain background containing an inducible prophage. Figure 6 shows a read coverage map across the island’s integration site. While the island is retained within the chromosome in the plasmid control as well as with the orf33-mutant expressing plasmid, no coverage is observed when orf33 wt is expressed. This is very odd when comparing to other phage satellites after induction under similar conditions or via a phage. Rather than loss of coverage, one would expect this to increase substantially as the island excises and replicates as indicated by their semi-quantitative PCRs. Instead, complete loss suggests that presence of orf33 is detrimental to the island and that these experimental conditions select for loss of it rather than induction. This would fit together with the limited stability of the island in its integration site and suggest that the additional selective pressure through orf33 expression will select against it. I also noted that the authors chose to measure restoration of the attC site to quantify/identify island induction through their orf33 experiments (Figure S. 17). I am surprised by this as in all other experiments the authors use primers that would identify circular island DNA after excision.
Orf33 shares homology to Mu-Gam protein. This protein binds to linear DNA ends and can protect these fragments from degradation. Furthermore, Gam, together with DNA ligase can constitute a nonhomologous end-joining repair pathway. The authors state that this protein is non-essential in their phage but do not provide data as to the impact of the frameshift mutation on the phage’s biology. Are there any differences in virulence, PFUs obtained after infection with a set amount of the phage mutant?
Given these results, I do not think that the authors have shown that these islands are induced by virulent prophages via orf33. It appears that orf33 has a different role.
No evidence provided that circular DNA is taken up. The authors do not provide a characterisation of the orf33 mutant’s impact on phage virulence and phage titres. By contrast, they show that even in the absence of orf33 you obtain transformants. How can you be certain that this is not caused by impacts on phage packaging or the ability of the phage to inject DNA into its host. The role of Gam could be to protect linear phage DNA when entering a new host.
Satellite prophages or PICI are normally packaged into phage particles when induced and can be transferred via induction. Have the authors tested this? Their methodology relies on filtering lysates, which would not remove any phage particles. To exclude that any form of transduction plays a role in their observations, this should be confirmed. Line 285-286 and Figure S11 indicate that there might either be transduction via the phage for the initial propagation or transfer of free StPI DNA from the lysate. It should be confirmed if this is that case.
Figure 1: The gene functions should be colour-coded in the figure. At least to the level depicted above to make identification easier. There are clear differences in architecture of the regulatory motif.

Figure 2: Missing the control for the integrated StPI. What is the ratio between integrated and excised StPIs? This is to make sure they are integrated normally and not just primarily excised. Overnight incubation seems arbitrary and why would they induce after an overnight incubation specifically? Is there any data to support this and what is the level of integrated/excised StPIs in other growth phases? Figures 3 shows differences in the level of excision among the different StPIs.

Figure 3: First lane indicates that SpPIs are low level integrated as the band is very weak, potentially suggesting that the primary lifestyle of these elements is not in their integrated form?

Figure 4: Why is the DGCC7719-DSP +SP not showing the lane with the red primer pair? It should be at least empty if all cells show integration but could show some band if not all have the SP in the chromosome. Given the previous results, I would also like to see the amount of circular StSP in this strain.

Figure 5: How similar or different is the StPI of DGCC7710 to the island providing resistance? Can a resistance determinant be identified? A comparison could be added. Are there any genes indicative of packaging interference or redirection present in the StPI? Are other natural isolates also resistant to this phage (CRISPR-spacers, etc.)? Have the authors tested whether there was loss of phage protection in the different StPI deletion strains?

Line 290-292: This statement is contradictory and if true the rationale for inducer mutant selection does not work. The authors would need to later proof that this was the case using an inducer mutant but cannot conclude this from their data.

Supplementary figure S7: The last panel is unclear. What is shown here? Is this the empty locus of attC? If so, how can you explain that there are bands in most of the selected colonies, while you also have bands in the top panel indicating integration?

Supplementary figures S8-12: missing a control for baseline levels of circularised island before infection. It would be better to quantify the differences before and after infection using qPCR. Please also include a graphical representation of primer binding sites and product sizes in the figures.
Figure S16: what are the other high coverage regions in the graphs?

Figure S17: this is measuring restoration of the attC site not induction or replication of the island.

Supplementary figure S18: I would like to see numbers of how much the orf33 mutant showed a reduced StPI competence transfer and on the phage titres from these lysates. How is orff33 impacting phage titres?

Figure S19: The flanking SNPs that are identical to the genome of the donor could suggest generalised transduction?

Minor comments:
Use of species abbreviation not consistent and alternating between S. thermophilus and S.t or St.
Table S1: Column header “Putative AttL/AttR sites” should be “Putative attL/attR sites”
Line 47: “to hijack” should be “hijacking”.
Line 70: “lysed” should be “lyse”
Line 93: missing reference to strain table of strains used for StPI identification.
Line 104-105: Given the lower GC content, what is the likely origin of these phage satellites?
Line 114: “Javan289-like” a word is missing here and the term Javan-like has not been defined.
Line 145-147: Something is missing in this sentence. There is justification but no statement that is being justified. Are satellite prophages being targeted or phages? Which one is know? If it is known that SPs are targeted by CRISPR, then where is the novelty?

Line 155: replace “few” with an accurate number and/or percentage.

Line 190: “orientation” should be “orientations”.

Line 250: “inti” should be “into”
Line 353-354: The phages cannot infect? On what basis? They cannot attach? They cannot inject DNA? Please clarify. If they can inject attach and inject DNA, there is no reason why they couldn't potentially inject StPIs as well.

Line 389-390: might also suggest that these elements rely on virulent phages for their propagation.

Reviewer #2

(Remarks to the Author)
The manuscript “Satellite prophages induced by virulent phages are mobilized by natural competence leading to phage resistance in a new host” NCOMMS-23-63184-T by Morency et al., describes a series of phage satellite systems found in Streptococcus thermophilus and other related species, which can interfere with lytic phages and mobilise via transformation into natural competent strains. From GenBank, they performed a bioinformatic analysis using sequence alignment from a S. thermophilus strain containing a phage satellite (StCISMQ-301) to find more phage satellites in S. thermophilus and close

related species. They identified similar characteristics to Staphylococcus aureus PICIs, (also known as SaPIs), containing integrases, replicases, and packaging genes direct with attachment sites at both ends of the element. Distinctively, they found several anti-phage systems within the accessory genes, which suggests that these elements can indeed have an impact in the evolution and ecology for the bacterium and its phages. The authors propose that these elements can be induced by lytic phages, while also act as a defense island that can allow the development of phage-resistant bacteria for fermentation technology.

The authors conducted experiments to understand the induction, and analysed the satellite's excision by examined the attC site on the chromosome with PCR. They employ a CRISPR interference plasmid to cure strains from the satellite, allowing them to generate a sensitive strain to phages and transfer other satellites. With these strains, they mobilised the StCISMQ-301 satellite via transformation and examined phage interference using different lytic phages, which allowed them to identify phages variants that had escaped the resistance by mutating a phage-encoded protein (named ORF33).

Overall, I find the authors manuscript exciting as the field of phage satellites keeps expanding and we keep observing different lifestyles that they opt to hijack and adapt to their helper phages. Their hypotheses are supported to some extend by evidence provided and I think it is an important system to characterise and a key finding to improve fermentation technologies.

Major concerns and comments:

The phage satellites described and used in their analyses were previously annotated as a phage satellite by Javan et al., and identified as PICIs by De Sousa et al., importantly these were classified as Type C, which depend on packaging proteins from their helper phage to mobilise. I would like to encourage the authors to follow the nomenclature already establish in the field, e.g. for E. coli is EcCIn, where first letters are referencing the specie, "CI" for chromosomal island, and "n" would be the specific strain containing the PICI.

The authors focus on the excision of the satellite. While excision is indeed important and part of the induction and packaging mechanisms of these elements, their findings lack evidence for the rest of the typical life cycle of an induced phage satellite. It is possible that they do not capture the full breadth of the interaction and excision is happening due to SOS-response after phage infection (as seen with P4 satellites and some PICIs).
Typically, induction will trigger the element to excise and undergoes extensive replication, to then use its packaging genes to hijack and interfere with the helper and other resident prophages. Evaluating replication is crucial and a standard for the field of satellites. Please see publications by Seed and Penadés where they've used either southern blots and/or qPCR to quantify and show replication patters in cell and encapsidation in phage particles.

Furthermore, if these elements can be induced by virulent phages, then these would probably transfer in their capsids into new host cells. Which would likely interfere with the phage production and result in less viable phage particles. Was there any inspection of the phage lysate? Was the satellite DNA packaged into the capsids?
Demonstrating the transfer rates of the phage satellite is important to validate these aspects, and to showcase that these can transfer into closely related species and potentially exploit the HGT driven by their new host phages.

Additionally, they mentioned that these elements were able to excise after an overnight incubation (Line 21-22), which highlight the unstable integration or independent excision via QS signals. I would test these aspects using the marked StCISMQ-301 with the chloramphenicol resistance prove if this is still the case under growth selection of chloramphenicol on cultures with infection and without infection.

The authors were able to identify mutations on ORF33 in their phages that enabled them to escape from the interference properties of the satellite, and they propose that this is the inducer of the satellite and therefore the defense system. I would argue that ORF33 is a gene that triggers or is targeted by the defence system in the satellite, as typically defence systems on PICIs and P4-satellites will carry their interference role against non-inducing phages without the induction of the satellites. (See Rousset et al., Cell Host Microbe 2022 and Fillol-Salom et al., Cell 2022). If ORF33 is indeed the inducer of the StCI, I would require seeing the protein-protein interaction with the satellite repressor (stl) to confirm the mechanism.

Minor comment

Transfer of PICIs and other satellites elements via transformation has been proven by using the concept of rebooting. I would argue that many PICIs, including those in Streptococcus can be acquired by transformation.

Since most PICIs hijack temperate phages. Where there any prophages tested in the interference and/or induction? If resident prophages are in the same strains, we would suspect those to be able to induce the satellite when infecting or being induce with mitomycin C.

Things are difficult to follow on Fig 1. Are the elements cluster by phage defence system?
Please provide a detail list with genes and putative roles in the figures where the genome organization is showcase. Also provide what the colour codes indicate and please take a careful look at the colour schemes used in different figures. Please consider using colour-blind friendly pallets in figure preparation (https://www.nature.com/articles/d41586-021-02696-z).

I believe there is a story to be told and there is genuine novelty on this satellite/defence system. However, to validate these hypothesis more details and experiments need attention.

Reviewer #3

(Remarks to the Author)
The authors describe a type of phage satellite that, although it is unclear if (and how) it differs from already known satellites, seems to move between bacteria through natural transformation. I find the idea intriguing, and something that, if shown, could provide an important contribution for our knowledge on the mobility of phage satellites. I honestly think there is something interesting at play here, but I find the study to be somewhat incomplete regarding the characterization of this mobile element, and also not very convincing regarding the natural relevance of the mechanism proposed here. My assessment is based on 5 main key points that I expand below, and for each I attempted to suggest additional analysis or clarifications that I hope can contribute to addressing some of the issues of the study.

1) There are several pieces of information missing in the characterization of this satellite. I understand that the manuscript might not be focused in an extensive computational analysis, but some details are nevertheless important in order to evaluate the biology of this potential new element. The paper begins by stating that "a genome analysis was conducted and a seemingly functional satellite was detected". However, there are no details on this process, particularly regarding how the satellite was detected, and what suggests that it is functional (and whether this assumption is based on computational or experimental evidence).

This follows with a very shallow in silico characterization of this satellite. It is not at all clear on whether this satellite is similar to known PICI, or if they are likely to form a novel family of satellites. Table S1 seems to indicate that these are different enough from known PICI to not be detected as such, but what are the differences or similarities? It is unclear whether it involves their gene repertoire, because this is also vague in the text. The authors refer to "regulatory module" and "replication proteins", but what are these proteins? Are they homologs of known genes in other satellites? E.g., alpA/stl, primases or TerS, in PICIs? Along the text, the authors refer what is known in PICIs/SaPIs to support some of their observations, hence it is important to know more about the similarities and differences between these two (types of?) elements.

The study is also not clear on the diversity of the group of putative satellites mentioned here. Fig 1 does not help to understand the homology between them, beyond a color-based functional (?) homology. There seems to be some diversity, at least in their length and (I assume) their accessory genes. Maybe a few less examples (in the main text), with ANI or other homology measure between the genomes of the elements would be beneficial. The authors mention that satellites were grouped based on their accessory genes as well as "other genes" (lines 112-113), but how was this grouping performed, and based on what exactly? (ie, what do "other genes such as the integrase" mean?) More details on how the satellites were grouped would be important. And also, what are the actual groups? Table S1 has several satellites, are they all represented by the groups in Fig1, or are there more groups? Maybe a column in this table with the corresponding group would be beneficial (if the methods for how the groups were created are made clearer).

The methodological information on the annotation of these satellites is also lacking. Methods refer the use of PECAAN to annotate satellites from 5 strains only (what about the others?). It is also unclear how were the defense systems in these satellites detected. Were they manually and individually inspected? Was an homology search performed with known systems? Did the authors use an automated software such as DefenseFinder? Information on what do these systems resemble (some genes in Fig 1 are annotated as restriction-modification enzymes or ABI genes), but a more systematic classification would be appreciated (and useful).

2) My second main issue concerns the construction of satellite-free strains. I understand that having a CRISPR-i that targets the integrase of the satellite would prevent it from re-integrating the genome once it spontaneously excises, but this system wouldn't prevent the satellite to remain in the cell as extrachromosomal, correct? The loss of the satellite could happen spontaneously, if it doesn't replicate much, as it would get "diluted" in the population during cell division. But I could find no data on the replication of satellite after excision, so it is difficult to evaluate the effect of dilution. Figure 3 shows PCRs for the presence of the plasmid (with the spacer that targets the integrase), and the genomic region corresponding to an excised satellite. But the authors should also perform a PCR for the circularized satellite (as for the previous experiment, in Figure 2, and using the appropriate controls) to confirm that the satellite is actually absent from the cell. Even if it seems that the satellite is not integrated in the genome, being sure that it is completely absent from the cell, even as extrachromosomal, is key for the following analyses.

3) A third main issue is with the actual proof that these satellites can move between cells by transformation, particularly in natural settings. Since these satellites excise and circularize spontaneously (according to Fig2), it is not clear to me why the authors used an artificially circular form of the satellite from PCR products. A more convincing evidence of a natural process of transfer could come from lysing the cells and allowing the substrate to serve as a source of transformable DNA into the recipient cell. Or to co-culture the donor and recipient strain, which following the natural death of donor cells could lead to the transfer of the satellite to the recipient cells. An additional control for any of these two experiments could involve inserting the CM5 cassette in another, non mobile region of the genome of a satellite-free strain, in order to isolate and quantify the effect of the transformation of the satellite.

The authors mention the impossibility of both transforming and integrating a linear PCR product, based on the length of

homology of the attachment sites. However, it is not evident for me why the length of the att sites should change depending on whether the satellite is circular or linear, and whether it is ligated or not. Wouldn't the integration be mediated by the Integrase in the satellite (and not the just the att sites)? Moreover, wouldn't this also mean that the spontaneous excision of the satellite prevents its re-integration in the original genome (and hence why use CRISPR-i in the first place to generate satellite-free mutants)? I admit that I might be missing some key rationale here, that can perhaps be better explained by the authors. Nevertheless, the important point here for me is: what would be the relevance of this type of transfer in nature, if it always requires an artificial ligation?

Another key, related, point is that there is no sense of the efficiency of the transfer of satellite by transformation. The absence of replicate experiments also does not allow to understand how reliable this mechanism of transfer is. Measuring the surviving colonies across replicates would allow one to estimate the frequency at which the transfer event happens (and thus its relevance in a natural setting). Even if the authors use an artificial ligated product, a few numbers would be of importance. Namely, how many colonies were resistant to chlorampenicol? And were all of them resistant because of the transformation and integration of the satellite, or just the transformation of the resistance gene? The gels show only one example (for each strain), but there is no sense on whether this was selected from a few surviving colonies or from a whole large surviving population.

4) A fourth point concerns the ability of satellites to defend against phage infection. First, the authors could develop a bit more on the mechanism that provides anti-phage resistance, beyond a suggestion that it might be related to induction of the satellite (lines 394-95). Two things could be done here, a computational prediction of the putative defense system (using, e.g., DefenseFinder) and/or a more extensive experimental analysis of the effect of each gene (using, e.g., CRISPR-i for the different satellite-encoded genes). A particular reason why I stress this point is because the SP-301 satellite used in these experiments doesn’t seem to have many accessory genes associated with defense systems, as the satellites SMQ-310-like seem to only have a very small ORF (in white)(based on Fig1, which as I already mentioned is not great to find homologies between elements).

Moreover, and as for transformation, there is little information about the variability of the infection, beyond a single plaque assay.

The role of the CAM resistance gene is also unclear. Is there an obvious expectation on why would the orientation of the CAM gene influence phage resistance? And could the phage resistance come from the CM resistant gene itself (as some chlorampenicol resistance mechanisms involve changes in the permeability of the cells, it could possible)? A possible control for the effect of the CAM gene is to build a DGCC7719-DeltaSP::CM5 to test its susceptibility to phage.

Related to the previous point, a more direct test of the influence of the satellite in phage resistance would be to generate a SMQ-310::deltaSP-310 (i.e., the original strain without the satellite, using CRISPR-i as before - and assuming it works well) and directly compare the phage resistance ability between the WT strains and the satellite-free strain. This would also avoid the need for a resistance marker gene.

Finally, the effect of a truncated orf33 in phage escape mutants could also need some more details. Particularly, more replicates would be beneficial to quantify the variation between assays (gels and plaques seem to correspond to one culture/sample). It is also unclear why the sequencing was used here instead of semi-quantitative PCRs as before (or vice versa, why wasn't sequencing used before to test for induction). It makes it complicated to compare across experiments.

5) My final main issue concerns the absence of proper quantification for the phage-mediated satellite induction and transfer. For instance, a qPCR-based quantification would be more informative than PCR gels (even if semi-quantitative). It would also benefit from using more replicates, since the gels only show one culture. Without this, it is difficult to properly compare the effect of satellite induction across phages (and in the absence of phages, where the satellite is also supposed to induce). For instance, the D4752 phage seems to induce less, but it is not clear. Also, regarding the excision of the satellite in strain DGCC7710wt (Figs S8-S11), it is unclear if the primers are specific to SP-310, or to native satellites of this strain. The native satellite does seems to excise when strain is exposed to some phages (Figs S10-S11). Moreover, before (lines 133-134) it was mentioned that the satellites excised in all strains tested, including strain DGCC7710 (line 129). Here (Figs S8-S11) no excision seems to be detected in absence of phage (but maybe because the primers are not appropriate? As I mentioned before, it is not clear).

For the phage-mediated transfer itself, my comments are similar. There is little data beyond a PCR and a plating assay, and no information about replicates (it seems to come from a single culture?). How many times was the infection/transfer attempted, and how many times did it succeed? Are there differences in the numbers of colonies with an integrated satellite when using lysates of phage with and lysates of phage without the orf33 mutation? Can the authors provide a measure of the efficiency of transfer of the satellite via the phage, and assess how does this efficiency relates to the transfer of the ligated product of the satellite (is it more/less)?

I have some other points that, although less important, could benefit from some details/clarifications/discussion:

- There is a discrepancy in describing the completeness of genomes in the different parts of the text. In the main text (line 92 and 94), and in the methods (line 407) the 86 (or 87, text and methods differ in the number) genomes are referred to as complete. However, in lines 151-152 it is mentioned that "… some of these S. t. genomes were incomplete". Is this a different

dataset? If not, please clarify whether the genomes are complete or not.

- I am intrigued by the presence of CRISPR spacers that target the satellites. It is not immediately clear to me why this would happen. If satellites act as anti-phage systems, why would they be actively targeted by the bacteria? The authors themselves mention that "… the presence of SP likely confer[sic] a benefit to the host cell" (lines 389-390). It would benefit the manuscript to hypothesize or discuss a bit this point. An additional piece of data that could help understand why this is the case would be to identify which genes are actually targeted by the CRISPR spacers (maybe there is an overlap with phage-encoded genes)?

- Related to the previous point: is the spacer targeting the CMP gene (lines 221-225) already found in the original genomes of the strains? Or was it artificially added to the genome in order to make a proof of principle that the CRISPR system is able to block the infection by the satellite?

- The induction of the satellite. The authors found that it could be spontaneously induced, or induced by virulent phages. Beyond line 389 (which refers to S. thermophilus as a whole group), I couldn't find any data or statement saying that there are no prophages on these strains (that could also serve as their helpers).

- Lines 281-286. I was a bit lost with this rationale. Why would the double amplification on a satellite-free strain mitigate the signal of the induced satellite?

- In FigS7, it is not clear what I am looking at in the lower panel. The primers are for the genomic region without SP-301? If so, it seems that in 8 out of 10 colonies that were infected (and survived) phage infection the SP-301 was absent/lost?

- Lines 146-147: Sentence has as strange formulation (starts with "… since this bacterial species…" but has no conclusion). Data used to support this only comes later in the paragraph. I suggest a re-write of this paragraph to avoid confusion.

- Lines 205-207: Why do the transformation results align with what is shown in SaPIs? I agree that the spontaneous excision could be compatible, but as far as I am aware, all the studied SaPIs require a phage to be transferred.

- Line 230: "It was proposed that specific SPs may encode antiviral systems". Please specify in which ones this was shown (P4, PICI…), as throughout the text "SP" refers to the satellites studied here, and it might confuse the readers.

- Some references are strange, e.g.,
- Line 97, reference 25 is used to support the similarity between PICI and and the satellite in this work, but ref 25 discusses that PICIs encode immune systems (maybe ref 7 or 10 would be more appropriate here?)
- Line 258, why is ProphageHunter an appropriate reference here?
- Line 274, why is a reference to the description of a temperate phage in Francisella relevant here?

- Some supplementary material seems to be inappropriately referenced. For instance, line 100 references Table 1 for the existence of two satellites in one genome. Since Table 1 has the read depth of plasmids used in an experiment from somewhere else in the manuscript (which should arguably be supplementary), I suspect the authors mean Table S1 here? Also, in line 130, should be Figure S2, I think. And line 421 cites Table 1, but it should be Table S1 as well.

- Some other imprecisions:
- Line 47: "hijack a portion of the viral assemblies of certain temperate phages". Not necessarily the case, as PLEs and PICMIs hijack virulent phages.
- Lines 49-50: "… markedly diminishes the replication and the release of new temperate phages". Again not necessarily so. cfPICI and PICMI were observed to have a very low (if any) cost for the hijacked phage.
- Sometimes S.t. is used, other times S. thermophilus (e.g., line 127, 184, 194, etc)
- CRISPR1 and CRISPR3 (line 150) correspond to the CR1 and CR3 mentioned previously (line 74)?

Finally, the manuscript could also greatly benefit from an overall revision of the text itself. There are several typos that can confuse the reader (al least in some cases it confused this one). Some examples:
• Line 21: "can excised"
• Line 70: "can infect and lysed"
• Line 74: "adaptative" (not really a typo in the strictest sense, but "adaptive" is a more common term)
• Line 75: "30-bp (spacer)". Not really sure what was meant to be written here. Sequence? Spacer without the parenthesis?
• Line 84: "… structurally similarities…"
• Lines 84-85: "… phages can triggered…"
• Line 114: ".. Belonging to the Javan289-like possessed…" -> Javan289-like group?
• Line 127: "To confirm if the activity of SP in S. thermophilus strains, we performed…"
• Line 167: "design" -> designed? to design?
• Line 187: "… through this transformation process." -> through A transformation process (or simply "through transformation")
• Line 244: "… as describe earlier,…"
• Line 250: "… introduced inti a new host…"
• Line 390: "… the presence likely confer…"
• Line 392: "… enabled us to use of virulent phages…"

Version 1:

Reviewer comments:

Reviewer #1

(Remarks to the Author)
I would like to thank the authors for providing some additional clarification and experimental data. However, I still believe that the current evidence does not (yet) fully support their conclusions and are in parts contradictory. Unfortunately, the authors have either misunderstood or chosen to ignore completely or partially many of the issues raised in the last version. Their responses could have been improved by citing the specific changes that had been introduced in response to received comments.
While I still believe that the authors potentially have identified a new type of virulent phage phage-inducible chromosomal island induction, there is some key evidence missing to confirm their conclusions.
Additionally, the organisation of the manuscript complicates its assessment and might benefit from some restructuring. For example, all the transformation experiments using ligated PCRs and phage lysates would make more sense to follow each other to focus on mobilisation rather than separating them. Focusing on clearly showing induction, including the relevant mechanisms first and then providing some evidence for the biological impact of this induction would make a more logical story provided the right evidence for each is presented.

Major comments:
General comment:
A major problem of the study is that the authors only assess induction semi-quantitatively based on OD/CFU estimates and do not relate quantity of SP relative induction levels to a chromosomal gene. While this data suggests that more attS and attC sites are formed, it is difficult to gauge how complete this is. All evidence is PCR-based and at no point do we see evidence of induction of the whole SP. I would have expected to see evidence of the spontaneous induction of SP in Figure 7, for example through increased read coverage across the SP-301 region compared to the surrounding chromosome. This is not evident from the current representation. There is also no attempt to confirm that the full length of the SP is excised and replicating. This would have been valuable data to support the ability of virulent phages to induce SPs completely and is applicable to many of the experiments that were performed using PCR.
Nomenclature of SP: The authors are trying to establish that virulent phages can induce these chromosomal islands, which they call satellite phages. It would be better to use a nomenclature that has been established in the field of phage-inducible chromosomal islands as indicated by reviewer 2. “The phage satellites described and used in their analyses were previously annotated as a phage satellite by Javan et al., and identified as PICIs by De Sousa et al., importantly these were classified as Type C, which depend on packaging proteins from their helper phage to mobilise. I would like to encourage the authors to follow the nomenclature already establish in the field, e.g. for E. coli is EcCIn, where first letters are referencing the specie, “CI” for chromosomal island, and “n” would be the specific strain containing the PICI.” Introducing a new independent nomenclature would complicate future cross referencing and complicate further analysis.
“Satellite phages can be mobilized in other strains via natural competence” and “SP mobility during phage infection”
The authors use PCR amplified and ligated SP in the experiments to assess mobilisation of the SP through natural competence (Figures 4, S5, S6). While they succeed in showing this, its relevance is not clear at this stage. In the end, this just reflects the uptake of any circular DNA into a naturally competent host. It becomes clearer once they perform their phage infection and lysate transformation experiments.
How are these SPs released? Is phage infection required? How much is released in a normal supernatant by spontaneous induction? Is this enough to transform via natural competence?
I would suggest combining this section (or have consecutive sections) with “SP mobility during phage infection”. It might make be more straightforward to show that lysates contain transformable DNA and then provide the in vitro ligated PCR product data to confirm the type of DNA (i.e. circular DNA is required). It would fit at the end of the results to give biological relevance. However, one important aspect that needs to be addressed here is the impact of the orf33 mutation on phage fitness. How can the authors be sure that the reduced number of transformants with this mutant is not due to fitness impacts? What were the phage titres after amplification for both? The materials and methods section states that 50 ul of lysate were used in the experiment. Given the lower phage titre and fitness impact of the orf33 mutant, could it be assumed that less DNA is released in the cells infected with mutant lysates compared to those infected with the wt strain? There is no control to monitor release of bacterial DNA as a reference for the general efficiency of DNA release. How can the authors be sure that they are providing the same quantity of bacterial lysate for both phages?

“SP-301-CAT is induced by a phage-285 encoded protein”
The authors show that infection with virulent phages can result in an increase in attS sites, indicative of excised and circularised SP. This is promising but requires further verification and supporting data (see below).
Another important consideration is how the ofr33 frameshift impacts the inducing phage. If the phage is severely impacted in terms of replication, phage titres and/or plaque sizes, loss of induction might no be due to the loss of orf33 but caused by lower overall phage reproduction and consequently lower levels of other phage proteins. From the data provided, the orf33 phage mutant is severely impaired in its replication when infecting a strain containing a non-induced SP (DGCC7710) with an almost 2-log reduction in phage titres compared to the wt phage. This alone could explain an inability of the phage to induce the SP and consequently for the uninduced SP not to provide protection against phage infection.
As clarified in their response, the authors use a constitutive expression vector to drive expression of orf33. This results in the complete loss of SP sequence coverage (Fig. 7), which the authors attribute to rapid loss of the SP after induction. However, my previous contention remains unaddressed. While it is possible that orf33 induces the island, this experiment provides no conclusive evidence for this but rather only allows to conclude that expression of orf33 wt is detrimental to the presence of

the SP. Is this why, whenever the authors are using this plasmid, they never show reconstitution of the attS site and focus on attC only?
A better and necessary experiment to address this might have been to either infect the SP containing strain with the wt or orf33 mutant phage and monitor excision and replication over a time course following infection (including a sample before adding the phage) via Illumina sequencing. This would have shown that orf33 was important for induction of the SP. To further corroborate this or as an alternative experiment, a tightly controlled, inducible-expression plasmid would have better allowed to monitor induction following orf33 expression. Again, a time course of induction would have been appropriate and could either be monitored by Illumina sequencing or Southern blotting.
The experiment showing that the regulatory region of SP-301 participated in the induction process is elegant. It clearly showed that a phage protein was targeting this region. However, infection with the orf33 phage mutant was not performed and thereby a crucial piece of evidence is missing. Again, only restoration of the attC site is monitored when expressing orf33 from a constitutive promoter not allowing any conclusion of whether it is responsible or only detrimental to the SP. Could the authors use a reporter plasmid to monitor the induction of replication genes to confirm the role of ORF33? This would also require the inducible expression or the protein to monitor induction.

“SP mobility during phage infection”
Satellite prophages or PICI are normally packaged into phage particles when induced and can be transferred via transduction. Have the authors evaluated this? Their methodology relies on filtering lysates, which would not remove any phage particles. To exclude that any form of transduction plays a role in their observations, this should be confirmed.
The authors state that “The virulent phage 2972 does not adsorb to the receiving strain UY03 thereby eliminating the transduction possibility.” However, no evidence for this statement is provided.
Additionally, I would like to see confirmation of whether SP-301 DNA is packaged into phage capsids or not.

Tables:
Please ensure consistent nomenclature of all strains used in the study and verify that all strains that are indicated in the manuscript are indeed present in the relevant table. Currently, several key strains are either missing or labelled inconsistently between the text and the table.

Figures:
General comments on figures and supplementary figures:
- The use of colours and line thickness as well as figure sizes is not very accessible. Colour coding for the different PCR products throughout the manuscript is extremely hard to see and the chosen colours are not colour-blind friendly. Only using large zoom on the pdf could colours be guessed at. Please rework this in future submissions according to the guidelines specified by reviewer 2 (https://www.nature.com/articles/d41586-021-02696-z). Please not that this comment refers to all and not only a single figure.
- Figures should have a descriptive title before launching into experimental details.
- Please consider the use of panel labels (a,b,c, etc.) this would greatly help with identifying the relevant sections between figures and text.
- Please check labelling of all figures. There are multiple instances where there are labelling errors in both main and supplementary figures.
Figure 1 is much improved, and I am happy with the changes together with Figure S1.
Figure 2 is still missing the relevant controls for integrated SP compared to the spontaneously induced SP. Figure S16 does not include any of the required information and relates to a different strain set. I appreciate that the authors have evaluated spontaneous induction of one of their SPs in other growth phases. This data should be included with the main manuscript and not only as a response to the reviewers. Why was this done for only one of their SPs? How was data normalised? Is the data they are showing due to bacterial growth rather than real changes in excision?
Figure 4: SP-301 appears to be far less stable in this strain background. There is also a considerable proportion of attC still present suggesting that the island is not fully integrated. Does the cat gene cause this? Figure S6 suggests that there are differences in stability between different strain backgrounds receiving the SP-301.
Figure 8: Please introduce panels to better label the different elements of the figure.
While there is evidence that phage infection induces the chimeric SP when using phage 2972, a complementary experiment using the orf33 phage mutant would support the role of the orff33 product in inducing SP-301 regulatory element containing SPs.
Expression from a constitutive promoter is not a suitable system to show that a gene product is responsible for the induction of an SP. Here, only restoration of the attC site is monitored but no evidence for the presence/absence or quantity of SP is provided.
Top panels: What are the green circles meant to represent?
Bottom left panel: should read “SP-DGCC7710 wt” not “DGCC7710 wt no-DNA control”
Figure 9: What are the green circles meant to represent?

Supplementary figures:
Figure S5: attC controls only performed for delta SP recipient but not for the strains after transformation. No control for stability of the integrated island (attS). This was done for Figure 4 and should have been also performed for S5.
Figures S10: There is some error in the labelling of this figure. From the experimental description in the results section, both samples were treated with phage and the difference is the presence or absence of SP-301 DNA. Currently, the figure states that this was added to all samples.

Minor comments:
Line 146-148:

“The sequencing of the resulting PCR products also confirmed the attC sequence for the three most common S.t. SP integration sites: ATATTTCCCTCTAAAATC (IS_a), ATTTCATGAAAAAATAC (IS_b), and AATTCTACAACAAAAT (IS_c) (Table S1).”
This statement is missing context, i.e. “As attS sites mirror attC sites, the sequencing of the resulting PCR products…”
Line 151:
“Because we could induce S.t. SPs and pinpointed the attC site,…” This sentence is redundant as already covered in the previous sentence.

Lines 169-170:
“Therefore, it is tempting to speculate that CRISPR-Cas may also serve as a defense system against SPs.” Yes, but also this could be considered evidence that these SP can move between streptococcal species pointing towards their mobility.

Lines 190-192:
“By conducting spot test assays, we verified if the loss of the SP in DGCC7710-ΔSP::pNZ123-RSR, UY03-ΔSP::pNZ123-RSR, SMQ-301-ΔSP::pNZ123-RSR and DGCC7891-ΔSP::pNZ123-RSR strains could increase their phage sensitivity when compared to the wild-type strains containing pNZ123.” There is something wrong with the nomenclature of the strain here. Do they really contain an integrated plasmid? Where are these strains in the strain table? Why did the authors assess the phage titres in strains with the plasmid? This is in contradiction to the previous paragraph where the plasmid was eliminated. Please correct/clarify both in the text as well as in the table.
Lines 207-208:
the authors could highlight that by amplifying from attL to just before attR, this would result in the complete circularised SP after ligation.
Lines 219-221:
“In contrast, when the SP-301-CAT-rev linear PCR product was transformed into the ΔSP strains with an equivalent amount of ligated DNA from SP-301-221 CAT-rev, no colonies containing integrated SP were detected (data not shown).” There is a reference missing: an equivalent amount of DNA relative to what?

Lines 273-283:
The authors use an elegant approach to select for SPs providing phage resistance. By challenging a transformation assay either with or without the addition of circular SP-301, the effectively exploited the anti-phage properties of this island to select for successful transformation of the markerless SP. There are some issues with the annotations in the cited figure S10 (see figure section).
I would also have liked to see how these newly transformed colonies performed on a spot assay and whether they would show the same levels of phage protections as the marked chloramphenicol-resistant SP.

Line 308: “Surprisingly” I am not sure why this is surprising, please remove.
Additionally, while overall colony numbers obtained are stated in the figure legend and text, there is no reference as to how many colonies/PCR products were Sanger sequenced to confirm attR and its flanking regions.

Lines 404, 410 &416: How many colonies/PCR products were sequenced for each?

Reviewer #2

(Remarks to the Author)
The authors have addressed to some extent some of my previous comments. I have added my comments to the authors in the same tables they've provided, highlighting my response in blue.

Reviewer #3

(Remarks to the Author)
The manuscript by Morency and colleagues underwent several modifications, including adding analyses, controls and visual schemes, but also by the re-writing of large portions of the text. These addressed many of the concerns I had raised, and I am happy to say that I consider the logic and the quality of the manuscript to have much improved. I find now no major issues, but I still have a few important points that I would like the authors to address. Most of these can be solved by data that is already produced, or by adding text that further explains/discusses these ideas.

- The targeting of CRISPR-Cas to SPs. I will not argue against the existence of spacers that target the SPs, but I'm not sure I agree that knowing the DNA targeted by specific spacer is irrelevant. Some proteins of SPs have commonalities with those found in virulent and/or temperate phages (Integrases, TerS, etc). Hence, a CRISPR spacer that was acquired from a phage might target a SP as a collateral effect. On the other hand, if they target SP specific genes (like stl or the equivalent regulatory protein in the StSPs), then this would be supporting evidence that bacteria are acquired spacers directly from the SP. The reason I insist on this is also because it would be nice to have some discussion on why would bacteria actively try to prevent infection by SPs that can provide an advantage (by protecting against some phage). Since the authors already have identified the spacers, the targeted DNA (which genes, from which SPs and from which strains) should be easy to pinpoint and present, and I think it would add a nice eco-evolutionary component to the paper.

- On the point of mobilization by transformation, I am now more convinced that is the case. I apologise that I missed the data

on mobilization under more natural conditions on Figures S17 and S18 in the previous version, but it was indeed not very clear what was being tested. Nevertheless, I am still a bit puzzled on the particular experiment that uses the artificially ligated SP. I understand that this is meant to provide a laboratory-specific assay to test the concept of the mobilization by transformation. As I was reading the new version, I initially assumed that this would be because the efficiency of transfer by natural transformation using just cell lysates would be too low to detect. But the SPs do circularize by themselves, and if I understand correctly the data in the last section of results (Figure S18), transfer under natural conditions (without phage induction) occurs on average in 12.5% of the cases, which is very reasonable. Thus these conditions should be enough to generate enough SP DNA and detect the transfer, especially if there is the CAT gene to select on. So the need to add a level of artificiality using the ligated form is still not clear to me. Perhaps the authors can just add a brief rationale at the beginning of this part (lines 207-208) that could help the readers follow the reasoning for their methodological approach? Even if it has a technical reason. I would also like to read some justification on why two orientations for the CAT gene were used, as well as some speculation on why it gives different results, given that only (?) the CAT-rev version worked in all four strains. Maybe some of these points could be more strongly addressed by including the quantifications of the efficiency of transfer here (and not just in the last experiment). If colonies were selected, there must have been colonies to count. Are the number of CAT resistant (and thus SP containing) colonies obtained with the ligated version that much higher? And are there differences between the CAT and CAT-rev efficiencies?

- Considering the phage resistance provided by the SP, I think it is quite convincing now. What is also interesting to me is that SP-301 provides no protection against the phages that infect its own host SMQ-301 strain (73 and DT1, lines 194-195), but it does provide resistance against phages that infect other strains. This to me suggests that some of these SPs might not only travel frequently between hosts, but might also undergo selection across multiple hosts as well (and the phages they encounter). If the authors agree, maybe some discussion could be added on this point?

- Finally, regarding the spontaneous excision of SPs. The authors suggest, in the conclusion, that SP can be lost naturally within a population ("While the spontaneous SP excision suggests a scarcity of these MGE within the bacterial population, especially if the SP fails to reintegrate the chromosome before cell division"). However, it would be beneficial to provide a point of speculation on whether the re-acquisition by transformation could counter-act this loss. Note that it would be extra nice to have some numbers too for this process of loss (how frequent it is) and this would add an important information for this process. But I understand it might fall out of the scope of the paper.

A few minor issues and edit suggestions:
- Line 98: "… seemingly functional SP…" -> Not wanting to be annoying, but I still think there is no obvious evidence (at this point in the text) that it is "functional". I would just say "a putative SP".

- The SMQ-310 and DGCC7710 strains have no prophages, as stated by the authors. Maybe I missed it, but what about the other strains from Figure 2? Can you state from early on (e.g., lines 138-139) whether this is the case or not?

- Line 161: "… or they have a different SP than the ones targeted by the spacers". Meaning a different group, or just different SPs in general?

- Line 196-197: Add "… and that these SPs provide no protection against infection by the tested virulent phages" Otherwise, the previous paragraph seems out of place. What is the rationale for testing phage-resistance here? Why no conclusion? I think this is supposed to provide a complementary point to the experiment on phage resistance later on, but here it seems a bit lost.

- Line 207: Figure 4, instead of Figure 2?

- Lines 311-312: "The orf33 is a non essential gene and it matched a putative…" -> Has homology with?

- Lines 429-430: "We found that swapping SPs enhance the overall phage resistance of a S.t. strain. " -> I would tamper this a bit and just say that "swapping SPs CAN enhance the overall phage resistance". Given that the phage-SP interaction might be specific, some pairs swapped might not give any resistance at all (and only one proper swap was tested in the manuscript).

- Figure S18 is out of order in the supplementary data file (I thought it was missing at first!)

Version 2:

Reviewer comments:

Reviewer #1

(Remarks to the Author)
Morency et al. “Satellite phages induced by virulent phages are mobilized by natural competence leading to phage resistance in a new host”
The revised manuscript represents a significant improvement. The authors provide compelling new evidence that virulent phages can induce Streptococcus thermophilus chromosomal islands (StCIs) via an interaction between the phage-encoded

Orf33 and the island-encoded repressor. I am convinced that this interaction underlies island derepression, and the data in Figure 6, cassette swaps and EMSA, strongly support a conserved mechanism similar to that seen in other PICIs.
While I have no major concerns with the central conclusion, several issues remain with the presentation and clarity of the manuscript. Some previous reviewer comments are not addressed in the text or rebuttal, and aspects of the experimental design and interpretation remain unclear. I detail these below.

Terminology and framing

The manuscript repeatedly refers to StCIs as “satellite phages.” This term implies they are defective prophages, which may not accurately reflect their evolutionary origin. I recommend replacing this with “phage satellites,” which avoids presuming origin while still describing their dependency on helper phages.

Mechanistic and interpretive gaps

The authors show convincingly that virulent phages induce StCIs, but the manuscript does not address whether StCIs are packaged into phage capsids. While negative data may not be available, this omission should be acknowledged. The authors could include a short discussion of plausible reasons for lack of packaging. For example, absence of a recognised pac site or TerS functon(no terS gene is annotated), and whether the StCIs might be cos-type PICIs instead. Could other virulent phages with compatible packaging machinery enable StCI mobilisation? How likely is transfer via released StCI DNA post-lysis compared to classical transduction mechanisms?

Issues requiring clarification or revision

• Lines 204–210: The rebuttal notes that strains were used before plasmid loss due to the emergence of mutations during plasmid curing. This should be stated clearly in the manuscript, along with whether the mutations affected phage resistance and, if so, their locations.
• Orf33 mutant titer: The rebuttal claims the orf33 mutant has higher titre than wild-type, but the manuscript states the opposite: wild-type phage yields 9.95×10^9 PFU/ml, compared to 2.27×10^8 PFU/ml for the mutant. Please clarify which is correct and ensure consistency between text and data.
• Lines 268 ff. (“StCI excision and replication”): This section is difficult to follow due to insufficient description of the qPCR setup. It is unclear where primers bind, what exactly is being amplified, or what is meant by "excised StCI" in this context. Why does primase copy number not correlate with excised StCI copy number? A schematic showing amplicon positions (as in other figures) would help, along with expanded legends and clearer explanation of the biological meaning of these measurements.
• Figures S8 and S9: The differences between these two datasets are not explained clearly in either the figure legends or the text. Clarify what each figure represents and what distinguishes them. Please clarify whether fold changes are plotted on a linear or log scale, and if statistics were performed on log-transformed values, state so in the legend.
• Lines 294–296: These two sentences contradict each other. Please revise for clarity.
• Lines 403–421: As above, a schematic showing the positions of primers and expected amplicons would greatly improve this section’s clarity.

Figure-specific feedback

• Figure 1: The “phage resistance protein” annotation appears speculative and is not supported elsewhere. Based on genomic context, a primase seems more plausible. Please revise or remove accordingly.
• Figure 3: While colour coding has improved, the colours may still be difficult to distinguish when reduced to journal page size. Consider using distinct patterns or line types to enhance clarity.
• Figure 5: There appears to be mislabelling of some strains in the figure or legend. Please check that all labels match and are correctly described.
• Figure 6: Excellent and compelling.
• Figure 8: As noted above, explain why primase copy number does not correlate with excised StCI despite being located on the island.
• Figures S18 and S19: The depiction in S18A is confusing; it appears to show the result rather than the experimental setup, which is misleading as panel A. Consider switching panels, so the experimental design comes first and clearly label outcomes. If S19 is an extended view of S18, please state this explicitly or combine them into one figure.

Minor revisions

• Line 59: “gram-positive” → “Gram-positive”
• Line 98: “was not” → “were not”
• Line 342: “an increased” → “an increase”
• SP vs StCI: Consistently use “StCI” throughout, including figures and legends (e.g. lines 161, 166, 168, 169, 172–185).
• Line 212–214: The statement about phages evolving to bypass restriction should be supported with data or omitted.
• Lines 239–242: Consider moving this into the earlier experiment description (lines 223–224) for better narrative flow.
• Lines 252–255: The inability to circularise the linear StCI might explain poor replication and low integrase dosage, preventing integration. This possibility should be discussed.
• Lines 385 ff.: The choice of a non-inducible expression system should be justified. While such systems may not yet exist for S. thermophilus, inducible tools (e.g. anhydrotetracycline-regulated) have been developed in this species (see PMID: 37191512).

Suggestions for future work (not required for acceptance)

Lines 357–369 note that orf33 is a MuGam-like end-protection protein. If Orf33 affects phage DNA replication rather than just StCI induction, this could impact plaque formation. Future work could test whether orf33 complementation in both donor and recipient restores phage replication and StCI induction. This would help clarify whether Orf33 is required primarily for island derepression, phage replication, or both. This is a suggestion only and not essential for the current manuscript.

Conclusion

This revised manuscript contains valuable insights into StCI biology and the role of phage-encoded inducers. While the

main conclusions are well supported, the manuscript would benefit from improvements in clarity, consistency, and figure annotation to ensure accessibility to a broader readership. I look forward to seeing a revised version addressing these concerns.

Reviewer #2

(Remarks to the Author)
I appreciate the authors' efforts in addressing the previous comments and providing additional experimental data. The change in nomenclature to StCI (Streptococcus thermophilus Chromosomal Island) is a welcome improvement for consistency within the field.

However, despite the new data, I remain largely unconvinced regarding the complete lifecycle of the StCI, particularly its replication. While the Illumina experiment (Figure 6 and Supp. Figure 17) and qPCR data (Figure 8) indicate increased excision after phage infection, the evidence for robust replication of the entire element is still not as clear as would be expected for a successful transfer mechanism.

Specifically, I note that the replicase expression in Figure 8 (assuming this refers to the qPCR of the replicase gene) does not appear to follow the same pattern as the excision of the element. This discrepancy raises questions about the efficiency of the replication cycle, and perhaps this is why the authors cannot transduce the element (maybe). For an element to be successfully transferred, extensive replication is crucial to generate sufficient copies for packaging and transfer. The current data, while showing some induction and excision, does not fully demonstrate the prolific replication necessary to support a complete satellite lifecycle.

The evidence for efficient replication of the entire StCI, comparable to what is seen in PICIs, PICMIs, P4s and PLEs, is still lacking. I would like to encourage the authors to further discuss this apparent disconnect between excision and replication, and perhaps explore why the replicase gene's expression pattern differs from the excision profile. This would significantly strengthen the manuscript's conclusions regarding the StCI's lifecycle and its classification.

This aspect is central to classifying these elements as novel phage satellites or understanding their complete mechanism of transfer. And while the transformation experiments seem to prove their transfer by this mechanism, the authors do not discuss the great implications that this mean of transfer would have on the field. Having a phage-induced element without transferring via viral particles seems like an extremely odd strategy after demonstrating that the StCI can interfere with the phage.

Reviewer #3

(Remarks to the Author)
The authors have now satisfactorily addressed all the points I had raised in the two previous rounds of revisions. I commend the authors for the additional analyses that make the study and its results much more robust.

Version 3:

Reviewer comments:

Reviewer #2

(Remarks to the Author)
The manuscript presents a substantial revision with further experimental analysis of Streptococcus thermophilus chromosomal islands (StCIs), addressing their excision behaviour, inducibility by lytic phages, transfer via natural competence, and associated phage resistance phenotypes.

At the same time, I believe several conclusions still require further clarification or experimental support to ensure they are fully aligned with the data presented.

While spontaneous excision of the StCIs is clearly demonstrated, the data suggests that such events occur at low frequency and without detectable replication under non-phage conditions. In non-infected cells, the StCI primase copy number remains comparable to single-copy chromosomal genes, suggesting that StCI activity is limited to excision rather than amplification. Clarifying this distinction more explicitly throughout the manuscript would help avoid overinterpretation of spontaneous StCI activity like other phage satellites and PICIs have.

StCI replication is inferred primarily from increased primase copy number during phage infection. Although this is a reasonable proxy for copy number, replication itself is not directly measured. In addition, the large fold changes observed—particularly for excised StCI forms are strongly influenced by normalisation to very low baseline levels, which complicates biological interpretation. Presenting these results with additional emphasis on absolute copy number estimates or raw qPCR metrics could help readers better contextualise the extent of replication (if any). In line with this, the graphical abstract should be modify as the replication state has not been validated and this figure could be representing a typical mechanism from phage satellites which the StCIs might not use.

The mobilisation of StCIs via natural competence is convincingly demonstrated under controlled laboratory conditions using circularised DNA and competence induction. However, the authors do not address how frequently or efficiently such transfer might occur outside of these experimental settings. Framing this explicitly as proof-of-principle mobilisation under defined conditions would help align the conclusions with the scope of the experiments.

Finally, although acquisition of StCIs correlates with increased resistance to several virulent phages, the underlying mechanism of resistance remains unresolved. The data do not distinguish whether resistance arises from StCI replication burden, direct interference with phage development, or indirect effects associated with island induction. Importantly, excision alone is unlikely to be the cause for phage resistance, as excision without dedicated interference functions would not be expected to substantially impair phage propagation. In well-characterised phage satellites with dedicated interference genes reduces phage progeny typically range from 100 to 1000-fold, reflecting active inhibition rather than excision per se. The resistance reported here may be involve an additional, as yet uncharacterised mechanism linked to StCI induction or to StCI interactions beyond excision alone (perhaps metabolic). Further clarification of this point, either experimentally or within a discussion of alternative mechanisms, would strengthen the interpretation of the resistance phenotype.

Overall, the findings support a model in which StCIs are conditionally inducible genetic elements whose excision are strongly dependent on phage encoded proteins. Further refinement of the distinction between excision and replication, clearer framing of mobilisation under experimental versus natural conditions, and additional insight into the basis of phage resistance would enhance the alignment between the data and the manuscript's conclusions.

Open Access This Peer Review File is licensed under a Creative Commons Attribution 4.0 International License, which permits use, sharing, adaptation, distribution and reproduction in any medium or format, as long as you give appropriate credit to the original author(s) and the source, provide a link to the Creative Commons license, and indicate if changes were made.
In cases where reviewers are anonymous, credit should be given to 'Anonymous Referee' and the source.
The images or other third party material in this Peer Review File are included in the article's Creative Commons license, unless indicated otherwise in a credit line to the material. If material is not included in the article's Creative Commons license and your intended use is not permitted by statutory regulation or exceeds the permitted use, you will need to obtain permission directly from the copyright holder.
To view a copy of this license, visit https://creativecommons.org/licenses/by/4.0/

**Comments from Reviewer #1**	**Our answers**
The authors use expression of orf33 from a plasmid in a strain background containing an inducible prophage. Figure 6 shows a read coverage map across the island's integration site. While the island is retained within the chromosome in the plasmid control as well as with the orf33-mutant expressing plasmid, no coverage is observed when orf33 wt is expressed. This is very odd when comparing to other phage satellites after induction under similar conditions or via a phage. Rather than loss of coverage, one would expect this to increase substantially as the island excises and replicates as indicated by their semi-quantitative PCRs. Instead, complete loss suggests that presence of orf33 is detrimental to the island and that these experimental conditions select for loss of it rather than induction. This would fit together with the limited stability of the island in its integration site and suggest that the additional selective pressure through orf33 expression will select against it. I also noted that the authors chose to measure restoration of the attC site to quantify/identify island induction through their orf33 experiments (Figure S. 17). I am surprised by this as in all other experiments the authors use primers that would identify circular island DNA after excision.	First, we apologize for a confusion which was likely due to the title of our manuscript, which we have now changed. Here, we are studying a satellite phage (SP), not a prophage. The text was clarified.<br><br>Regarding Orf33, we used a constitutive overexpression of Orf33. Therefore, the SP is rapidly lost after cell division, which increased the number of cells with an empty *attC* site.<br><br>We didn't follow induction overtime with the plasmid as the constitutive overexpression led to a rapid excision of the SP. The primers binding on the excised SP were used to observe the SP replication overtime after phage infection (but not with the plasmid).<br><br>Figures were modified for clarity.
Orf33 shares homology to Mu-Gam protein. This protein binds to linear DNA ends and can protect these fragments from degradation. Furthermore, Gam, together with DNA ligase can constitute a nonhomologous end-joining repair pathway. The authors state that this protein is non-essential in their phage but do not provide data as to the impact of the frameshift mutation on the phage's biology. Are there any differences in virulence, PFUs obtained after infection with a set amount of the phage mutant?	Orf33 of the virulent phage 2972 induces the SP found in the *S. thermophilus* strain SMQ-301 but not the SP found in the *S. thermophilus* strain DGCC7710. This indicates that the SP induction by Orf33 is SP-specific.<br><br>If the role of Orf33 was only to protect DNA from degradation, it should protect the DNA of both SPs (from DGCC7710 and SMQ-301), and we would not have observed different phage induction patterns between these two SPs (Figures 6 and S11)<br><br>Orf33 is not essential, since we can isolate functional phages that have a truncated Orf33 (Orf33 mut). We also added a reference.<br><br>As requested, we have performed experiments to compare phage plaque sizes and titers with the wild-type phage and the orf33 mutant phage. The text was modified accordingly.

Given these results, I do not think that the authors have shown that these islands are induced by virulent prophages via orf33. It appears that orf33 has a different role.	See above. Again, we are sorry for the confusion. There is no prophage in the strain. It is induced by a virulent phage. Text was clarified. We have also performed extra experiments see new Figure 8.
No evidence provided that circular DNA is taken up. The authors do not provide a characterisation of the orf33 mutant's impact on phage virulence and phage titres. By contrast, they show that even in the absence of orf33 you obtain transformants. How can you be certain that this is not caused by impacts on phage packaging or the ability of the phage to inject DNA into its host. The role of Gam could be to protect linear phage DNA when entering a new host.	We apologize for the confusion here because in the natural competence assay (Figure 4), we did not use phages. We used only PCR products that were either linear (per usual) or circularized (by us). Transformants were obtained only with circular DNA.<br>In the natural competence assay when we used phages to induce the SP-301-CAT (Figures S17 & S18), indeed, the role of Gam-like protein may be to protect linear phage DNA when entering a new host but we have shown that the orf33 of the virulent phage 2972 induces the SP (Figure 8). Therefore, in the absence of orf33, there is no SP induction (except for spontaneous induction), which could explain the very few (4) transformants obtained. In contrast, we obtained way more transformants with the wild-type 2972 that induces the SP-301-CAT. The text was clarified.
Satellite prophages or PICI are normally packaged into phage particles when induced and can be transferred via induction. Have the authors tested this? Their methodology relies on filtering lysates, which would not remove any phage particles. To exclude that any form of transduction plays a role in their observations, this should be confirmed. Line 285-286 and Figure S11 indicate that there might either be transduction via the phage for the initial propagation or transfer of free StPI DNA from the lysate. It should be confirmed if this is that case.	The virulent phage 2972 does not adsorb to the receiving strain UY03 thereby eliminating the transduction possibility. We have also done a control containing only the phages and bacteria with no ComS peptide and we did not have any colony in this condition. The text was clarified.
Figure 1: The gene functions should be colour-coded in the figure. tit least to the level depicted above to make identification easier. There are clear differences in architecture of the regulatory motif.	Figure 1 was modified accordingly. More details on gene homology between the SPs of *S. thermophilus* are available in Supp. Figure 1.
Figure 2: Missing the control for the integrated StPI. What is the ratio between integrated and excised StPIs? This is to make sure they are integrated normally and not just primarily excised. Overnight incubation seems arbitrary and why would they induce after an overnight incubation specifically? Is there any data to support this and what is the level of integrated/excised StPIs in other growth phases?	The information about the relative ratio between integrated and excised SP was added in new Supp. Figure 16. There is significantly more integrated SP than excised.<br>Indeed, the selection of overnight incubation without phages was arbitrary but it clearly showed that

	the SP was spontaneously excised. As requested, we have tested if there was SP spontaneous induction in other growth phases and the answer is yes. See Figure A below.
Figures 3 shows differences in the level of excision among the different StPIs. Figure 3: First lane indicates that SpPIs are low level integrated as the band is very weak, potentially suggesting that the primary lifestyle of these elements is not in their integrated form?	It is in fact the opposite. First lane indicates low level of occupied AttC sites. Figure 3 was modified for clarity.
Figure 4: Why is the DGCC7719-DSP +SP not showing the lane with the red primer pair? It should be at least empty if all cells show integration but could show some band if not all have the SP in the chromosome. Given the previous results, I would also like to see the amount of circular StSP in this strain.	As requested, we have modified Figure 3 and 4 with more PCR conditions.
Figure 5: How similar or different is the StPI of DGCC7710 to the island providing resistance? Can a resistance determinant be identified? A comparison could be added. Are there any genes indicative of packaging interference or redirection present in the StPI? Are other natural isolates also resistant to this phage (CRISPR-spacers, etc.)?	Homology between SPs found in the *S. thermophilus* strains DGCC7710 and SMQ-301 is now available in Supp. Figure 1. We ran the recent versions of DefenseFinder & Padloc and could not identify a known defense or interference mechanism.
Figure 5: Have the authors tested whether there was loss of phage protection in the different StPI deletion strains?	Yes, it was tested, and no loss of phage protection was observed. We added this information into the text.
Line 290-292: This statement is contradictory and if true the rationale for inducer mutant selection does not work. The authors would need to later proof that this was the case using an inducer mutant but cannot conclude this from their data.	Agree. This was a mistake. The text was changed accordingly. The resistance phenotype is SP-specific and not only related to SP induction or copy number.
Supplementary figure S7: The last panel is unclear. What is shown here? Is this the empty locus of attC? If so, how can you explain that there are bands in most of the selected colonies, while you also have bands in the top panel indicating integration?	Yes, it is the empty locus of attC. The SP has spontaneously excised in some cells as shown in Figure 2 and Figure A in this document.
Supplementary figures S8-12: missing a control for baseline levels of circularised island before infection. It would be better to quantify the differences before and after infection using qPCR. Please also include a graphical representation of primer binding sites and product sizes in the figures.	The goal here was to show the qualitative increase of induction over time. Time 15 was our baseline. Figure 6 and Supp. Figure 11 were modified as requested for more clarity.
Figure S16: what are the other high coverage regions in the graphs?	The high coverage regions are rRNA regions, we have modified (now) Supp. Figure 14.
Figure S17: this is measuring restoration of the attC site not induction or replication of the island.	Title of the Figure was changed to clarify as indeed it shows the restoration of the *attC* site.
Supplementary figure S18: I would like to see numbers of how much the orf33 mutant showed a reduced StPI competence transfer and on the phage titres from these lysates. How is orff33 impacting phage titres?	As suggested, colony numbers were added in the legend, and we have done more experiments to see the impact of *orf33* on the phage fitness (added in the main text).

Figure S19: The flanking SNPs that are identical to the genome of the donor could suggest generalised transduction?	No, because the phage 2972 does not adsorb to the strain UY03. The text was clarified.
Use of species abbreviation not consistent and alternating between S. thermophilus and S.t or St.	Noted. We kept *S.t.*
Table S1: Column header "Putative attL/attR sites" should be "Putative attL/attR sites"	Modified accordingly
Line 47: "to hijack" should be "hijacking".	Modified accordingly
Line 70: "lysed" should be "lyse"	Modified accordingly
Line 93: missing reference to strain table of strains used for StPI identification.	Table S1 was added in the text.
Line 104-105: Given the lower GC content, what is the likely origin of these phage satellites?	We do not know the origine of the SP in *S. thermophilus*. They are quite different than phages typically infecting *S. thermophilus* strains. The average GC content of SP is approx. 38%, which is similar to *S. thermophilus* genome. Javan *et al.* 2019 have also noticed a lower GC content in their SP when compared to the whole bacterial genome.
Line 114: "Javan289-like" a word is missing here and the term Javan-like has not been defined.	SP group was added. We have now redefined the SP groups based on a VirClust analysis.
Line 145-147: Something is missing in this sentence. There is justification but no statement that is being justified. Are satellite prophages being targeted or phages? Which one is know? If it is known that SPs are targeted by CRISPR, then where is the novelty?	Indeed, we are sorry for this mistake. Sentence was corrected and it is indeed novel as presented here.
Line 155: replace "few" with an accurate number and/or percentage.	Modified accordingly
Line 190: "orientation" should be "orientations".	Modified accordingly
Line 250: "inti" should be "into"	Modified accordingly
Line 353-354: The phages cannot infect? On what basis? They cannot attach? They cannot inject DNA? Please clarify. If they can inject attach and inject DNA, there is no reason why they couldn't potentially inject StPIs as well.	They cannot adsorb and we observed no plaque in a phage plaque assay. We have also done a control containing only the phages and bacteria (no ComS) and we obtained no transductant. We have clarified the text.
Line 389-390: might also suggest that these elements rely on virulent phages for their propagation.	It could be, but we showed in this study that SPs can spontaneously excised from the chromosome (with or without a phage infection) and SP DNA can be mobilized to other cells by natural competence.

**Comments from Reviewer #2**	**Our answers**
The phage satellites described and used in their analyses were previously annotated as a phage satellite by Javan et al., and identified as PICIs by De Sousa et al., importantly these were classified as Type C, which depend on packaging proteins from their helper phage to mobilise. I would like to encourage the authors to follow the nomenclature already establish in the field, e.g. for E. coli is EcCIn, where first letters are referencing the specie, "CI" for chromosomal island, and "n" would be the specific strain containing the PICI.	We added the term StCI in the introduction. We conducted an extra analysis using SatelliteFinder by De Sousa et al. 2023 to have a better classification of these elements (Table S1).
The authors focus on the excision of the satellite. While excision is indeed important and part of the induction and packaging mechanisms of these elements, their findings lack evidence for the rest of the typical life cycle of an induced phage satellite. It is possible that they do not capture the full breadth of the interaction and excision is happening due to SOS-response after phage infection (as seen with P4 satellites and some PICIs). Typically, induction will trigger the element to excise and undergoes extensive replication, to then use its packaging genes to hijack and interfere with the helper and other resident prophages.	A friendly reminder that the excision of the SP described in this study can occur spontaneously induced without helper phages and there is no complete prophage in the strain (SMQ-301). We have clarified the text to avoid confusion.
Evaluating replication is crucial and a standard for the field of satellites. Please see publications by Seed and Penadés where they've used either southern blots and/or qPCR to quantify and show replication patterns in cell and encapsidation in phage particles.	We have now shown the replication of the satellite phage in the semi-quantitative PCR, we have added a new figure (Figure 8).
Furthermore, if these elements can be induced by virulent phages, then these would probably transfer in their capsids into new host cells. Which would likely interfere with the phage production and result in less viable phage particles. Was there any inspection of the phage lysate? Was the satellite DNA packaged into the capsids?<br><br>Demonstrating the transfer rates of the phage satellite is important to validate these aspects, and to showcase that these can transfer into closely related species and potentially exploit the HGT driven by their new host phages.	When we amplified the virulent phage 2972 on *S. thermophilus* DGCC7710::SP-301-CAT (DGCC7710 derivative containing the SP of strain SMQ-301), all the isolated phages had a mutation in orf33 and they cannot induce the SP-301-CAT. We also did not observe a clear lysis during phage amplification. These results suggest that the virulent phages that can lyse the cells have quickly evolved or/and were strongly selected to prevent SP induction.<br><br>We conducted a transduction assay using phage 2972 lysate amplified on *S. thermophilus* DGCC7710::SP-301-CAT. We added 1.8 x 10^6 pfu to 5.1 x 10^7 cfu of the strain DGCC7710-ΔSP (containing a marker to differ from cells in the lysate) and we got only one transductant. This very low

	frequency (0.00006%) may be generalized transduction.<br><br>We had a control with the same condition using a phage 2972 lysate amplified on *S. thermophilus* DGCC7710:: SP-DGCC7891 (with an erythromycin resistance gene). **There was no phage resistance**, so it is unlikely that this phage packaged specifically the SP of DGCC7891 and we had a higher phage titer. We used 1.4 x 10^8 pfu and we got 86 colonies, which represents a transduction frequency of 0.00006%, which is exactly the same as in the 2972 lysate amplified on DGCC7710::SP-301-CAT with more than 4 logs of phage resistance. Therefore, we do not think that the phage resistance phenotype is related to the encapsidation.
Additionally, they mentioned that these elements were able to excise after an overnight incubation (Line 21-22), which highlight the unstable integration or independent excision via QS signals.<br><br>I would test these aspects using the marked StCISMQ-301 with the chloramphenicol resistance prove if this is still the case under growth selection of chloramphenicol on cultures with infection and without infection.	In fact, these SP are rather stable as they are found in most wild-type *S. thermophilus* strains for which the genome has been sequenced to date, presumably because they offer a fitness advantage (for example phage resistance). And here we show that they may be transferred from one strain to another by natural competence.<br><br>We have done an extra experiment that shows that the excision of the SP of strain *S. thermophilus* DGCC7710 is dependant on its regulatory proteins. Indeed, the SP of DGCC7710 is <u>not</u> induced by the virulent phage 2972. But it is induced by phage 2972 when we swapped its regulatory proteins with the ones from SP found in strain *S. thermophilus* SMQ-301. There was no chloramphenicol in this experiment (Figure 8).<br><br>We also added an extra figure showing that in a culture of a wild-type *S. thermophilus,* the level of integrated SP is much higher that the spontaneous excised SP (Figure S15).

The authors were able to identify mutations on ORF33 in their phages that enabled them to escape from the interference properties of the satellite, and they propose that this is the inducer of the satellite and therefore the defense system. I would argue that ORF33 is a gene that triggers or is targeted by the defence system in the satellite, as typically defence systems on PICIs and P4-satellites will carry their interference role against non-inducing phages without the induction of the satellites. (See Rousset et al., Cell Host Microbe 2022 and Fillol-Salom et al., Cell 2022). If ORF33 is indeed the inducer of the StCI, I would require seeing the protein-protein interaction with the satellite repressor (stl) to confirm the mechanism.	The focus of this paper is not on the induction mechanism, but we conducted an extra experiment showing that the phage-encoded Orf33 alone is inducing SPs by interacting with their regulatory orfs. (Figure 8) These results were added to the main text.
Transfer of PICIs and other satellites elements via transformation has been proven by using the concept of rebooting. I would argue that many PICIs, including those in *Streptococcus* can be acquired by transformation.	And yet, nobody as shown it by natural transformation until this manuscript.
Since most PICIs hijack temperate phages. Where there any prophages tested in the interference and/or induction? If resident prophages are in the same strains, we would suspect those to be able to induce the satellite when infecting or being induce with mitomycin C.	There is no prophage in the *S. thermophilus* strains used in this study. This is now mentioned clearly in the manuscript.
Things are difficult to follow on Fig 1. are the elements cluster by phage defence system? Please provide a detail list with genes and putative roles in the figures where the genome organization is showcase. Also provide what the colour codes indicate and please take a careful look at the colour schemes used in different figures. Please consider using colour-blind friendly pallets in figure preparation (https://www.nature.com/articles/d41586-021-02696-z).	We do agree. We modified Figure 1 accordingly and we made a simpler version. The homology details between each satellite phage are also available in Supp. Figure 1.<br><br>Colors have been changed accordingly.

**Comments from Reviewer #3**	**Our answers**
1) There are several pieces of information missing in the characterization of this satellite. I understand that the manuscript might not be focused in an extensive computational analysis, but some details are nevertheless important in order to evaluate the biology of this potential new element. The paper begins by stating that "a genome analysis was conducted and a seemingly functional satellite was detected". However, there are no details on this process, particularly regarding how the satellite was detected, and what suggests that it is functional (and whether this assumption is based on computational or experimental evidence).	We have simplified Figure 1. We also conducted a new analysis to group the SPs based on their protein homology using VirClust (Figure S1). We first identified the SPs with a sequence homology analysis, we double-checked the identified SPs using SatelliteFinder. In the lab, we have identified, by PCR, the attL and attR sites from the identified insertion sites with primers on the excised SPs, so we could define the exact length of the SPs. We clarified the text and added the extra analysis in the Materials/Methods section.
This follows with a very shallow in silico characterization of this satellite. It is not at all clear on whether this satellite is similar to known PICI, or if they are likely to form a novel family of satellites.	We apologize for this omission. Souza et al. previously classified some of the SPs from *S. thermophilus* into types C and D. Figure 1 and TableS1 were modified.
Table S1 seems to indicate that these are different enough from known PICI to not be detected as such, but what are the differences or similarities?	We used SatelliteFinder to confirm the SP in Table S1 and they are indeed PICIs (types C and D).
It is unclear whether it involves their gene repertoire, because this is also vague in the text.	The text was clarified.
The authors refer to "regulatory module" and "replication proteins", but what are these proteins? Are they homologs of known genes in other satellites? E.g., alpha/stl, primases or TerS, in PICIs?	Figure 1 was simplified and modified according to the comments of the other reviewers. The text was also clarified.
along the text, the authors refer what is known in PICIs/SaPIs to support some of their observations, hence it is important to know more about the similarities and differences between these two (types of?) elements.	We agree. The text was modified at various places to account for this comment.
The study is also not clear on the diversity of the group of putative satellites mentioned here. Fig 1 does not help to understand the homology between them, beyond a color-based functional (?) homology. There seems to be some diversity, at least in their length and (I assume) their accessory genes. Maybe a few less examples (in the main text), with ANI or other homology measure between the genomes of the elements would be beneficial.	We do agree and we have then conducted a VirClust analysis to group the SPs based on their protein homology (see Supp. Figure 1). In Figure 1, we now kept only one representative of each cluster.
The authors mention that satellites were grouped based on their accessory genes as well as "other genes" (lines 112-113), but how was this grouping performed, and based on what exactly? (ie, what do "other genes such as the integrase" mean?) More details on how the satellites were grouped would be important. And also, what are the actual groups? Table S1 has several satellites, are they all represented by the groups in Fig1, or are there more groups? Maybe a column in this table with the corresponding group would be beneficial (if the methods for how the groups were created are made clearer).	We modified the figures, table and text accordingly.

The methodological information on the annotation of these satellites is also lacking. Methods refer the use of PECAAN to annotate satellites from 5 strains only (what about the others?).	GenBank annotations were used for most of the SPs, but 5 strains were not annotated, so we used PECAAN to make annotations. We adjusted the figure titles accordingly.
It is also unclear how were the defense systems in these satellites detected. Were they manually and individually inspected? Was an homology search performed with known systems? Did the authors use software such as DefenseFinder? Information on what do these systems resemble (some genes in Fig 1 are annotated as restriction-modification enzymes or ABI genes), but a more systematic classification would be appreciated.	We used Padloc and DefenseFinder to verify if there was a known defense mechanism in the SPs of SMQ-301 SP, but it is not the case.<br><br>We are no longer using phage resistance is our SP grouping.
2) My second main issue concerns the construction of satellite-free strains. I understand that having a CRISPR-i that targets the integrase of the satellite would prevent it from re-integrating the genome once it spontaneously excises, but this system wouldn't prevent the satellite to remain in the cell as extrachromosomal, correct? The loss of the satellite could happen spontaneously, if it doesn't replicate much, as it would get "diluted" in the population during cell division. But I could find no data on the replication of satellite after excision, so it is difficult to evaluate the effect of dilution. Figure 3 shows PCRs for the presence of the plasmid (with the spacer that targets the integrase), and the genomic region corresponding to an excised satellite. But the authors should also perform a PCR for the circularized satellite (as for the previous experiment, in Figure 2, and using the appropriate controls) to confirm that the satellite is actually absent from the cell. Even if it seems that the satellite is not integrated in the genome, being sure that it is completely absent from the cell, even as extrachromosomal, is key for the following analyses.	We did not used CRISPRi (dead Cas9).<br><br>We used the endogenous type II-A (Cas9) CRISPR-Cas of *S. thermophilus*. Thus, the SP DNA was cut and thus, it wouldn't stay very long in the cell. We added more PCR conditions and modified Figure 3 accordingly. It confirms that no extrachromosomal satellite phages are in the DGCC7710-delta-SP strain.
3) A third main issue is with the actual proof that these satellites can move between cells by transformation, particularly in natural settings. Since these satellites excise and circularize spontaneously (according to Fig2), it is not clear to me why the authors used an artificially circular form of the satellite from PCR products. A more convincing evidence of a natural process of transfer could come from lysing the cells and allowing the substrate to serve as a source of transformable DNA into the recipient cell. Or to co-culture the donor and recipient strain, which following the natural death of donor cells could lead to the transfer of the satellite to the recipient cells. An additional control for any of these two experiments could involve inserting the CM5 cassette in another, non mobile region of the genome of a satellite-free strain, in order to isolate and quantify the effect of the transformation of the satellite.	We certainly agree but a natural process of transfer was <u>already</u> shown (see Figures S17 and S18). It came from lysing the cells and allowing the substrate to serve as a source of transformable DNA into the recipient cell. As a control, we used a phage mutant that did not induce the SP. Text was clarified.

The authors mention the impossibility of both transforming and integrating a linear PCR product, based on the length of homology of the attachment sites. However, it is not evident for me why the length of the att sites should change depending on whether the satellite is circular or linear, and whether it is ligated or not. Wouldn't the integration be mediated by the Integrase in the satellite (and not the just the att sites)? Moreover, wouldn't this also mean that the spontaneous excision of the satellite prevents its re-integration in the original genome (and hence why use CRISPR-i in the first place to generate satellite-free mutants)? I admit that I might be missing some key rationale here, that can perhaps be better explained by the authors. Nevertheless, the important point here for me is: what would be the relevance of this type of transfer in nature, if it always requires an artificial ligation?	It seems that we were not clear enough here. We are sorry. In fact, the absence of transformation is not related to the length homology, we simply didn't get transformant with the linear PCR product as compared to the circularized PCR product. Integration of the ligated SP is not related to homologous recombination. In natural settings, the SP will be excised and circularized and release in the environment. Here, we first generated a PCR product to have enough DNA for the natural transformation protocol in the lab. The text was clarified.
Another key, related, point is that there is no sense of the efficiency of the transfer of satellite by transformation. The absence of replicate experiments also does not allow to understand how reliable this mechanism of transfer is. Measuring the surviving colonies across replicates would allow one to estimate the frequency at which the transfer event happens (and thus its relevance in a natural setting). Even if the authors use an artificial ligated product, a few numbers would be of importance. Namely, how many colonies were resistant to chlorampenicol? And were all of them resistant because of the transformation and integration of the satellite, or just the transformation of the resistance gene? The gels show only one example (for each strain), but there is no sense on whether this was selected from a few surviving colonies or from a whole large surviving population.	We agree that numbers would be better. But here, we simply wanted to show that it would be feasible to use a natural transformation lab protocol (with added transformation peptide) to transfer the satellite prophage.<br><br>Now, as requested, we have added the colony numbers in our last experiments (Figure S17), which is mimicking natural settings and also using the virulent phages as the selection for the transformants (Figure S9).
4) A fourth point concerns the ability of satellites to defend against phage infection. First, the authors could develop a bit more on the mechanism that provides anti-phage resistance, beyond a suggestion that it might be related to induction of the satellite (lines 394-95). Two things could be done here, a computational prediction of the putative defense system (using, e.g., DefenseFinder) and/or a more extensive experimental analysis of the effect of each gene (using, e.g., CRISPR-i for the different satellite-encoded genes). A particular reason why I stress this point is because the SP-301 satellite used in these experiments doesn't seem to have many accessory genes associated with defense systems, as the satellites SMQ-310-like seem to only have a very small ORF (in white)(based on Fig1, which as I already mentioned is not great to find homologies between elements).	We used Padloc and DefenseFinder to check for known defense system in the SP of SMQ-301, to no avail. We do not know the anti-phage mechanism. But we do know that it can be used to select SP-containing transformant. Considering the recent publications in the field of phage defense, it would require a significant amount of work to solve this, which we believe is way beyond the scope of the message we want to convey here: natural transformation can be used to move SP, leading to improve phage resistance. Of note, we have added extra data in which we removed the SP in strain DGCC7710::SP-301-CAT and the phage sensitivity was restored (Figure S8), confirming the role of the SP in phage resistance.

Moreover, and as for transformation, there is little information about the variability of the infection, beyond a single plaque assay.	Plaque assays were done multiple times, with many bacteria and phages. It is the standard assay to determine phage resistance. The number of assays (n) was added to the legend of the Figure and the phage reductions were added to the text.
The role of the CAM resistance gene is also unclear. Is there an obvious expectation on why would the orientation of the CAM gene influence phage resistance? And could the phage resistance come from the CM resistant gene itself (as some chlorampenicol resistance mechanisms involve changes in the permeability of the cells, it could possible)? A possible control for the effect of the CAM gene is to build a DGCC7719-DeltaSP::CM5 to test its susceptibility to phage.	The CAM gene has no impact of the phage resistance phenotype.<br>The requested control had been done (and now added). We apologize that it was not initially in the Figure. It should also be noted that in Figure S9 and S10, transformants (containing a new SP) were selected without CAM and still provided phage resistance.
Related to the previous point, a more direct test of the influence of the satellite in phage resistance would be to generate a SMQ-310::deltaSP-310 (i.e., the original strain without the satellite, using CRISPR-i as before - and assuming it works well) and directly compare the phage resistance ability between the WT strains and the satellite-free strain. This would also avoid the need for a resistance marker gene.	We could not do the proposed experiment as the virulent phages are already adapted to the wild-type defense mechanism.<br>However, we did swap the satellite prophage from one strain to another, and the phage sensitivity profile of the strain with a new SP changed, became phage resistant. In addition, now, we have removed the new SP from that strain, and it became phage sensitive again, thereby confirming the role of the SP in the phage resistance (Figure S8).
Finally, the effect of a truncated orf33 in phage escape mutants could also need some more details. Particularly, more replicates would be beneficial to quantify the variation between assays (gels and plaques seem to correspond to one culture/sample). It is also unclear why the sequencing was used here instead of semi-quantitative PCRs as before (or vice versa, why wasn't sequencing used before to test for induction). It makes it complicated to compare across experiments.	We have done extra experiments to add more details on the truncated Orf33, such as the plaque size and phage titer and the text was modified accordingly.<br>We added the number of replicates in the relevant figure legends. We used the sequencing to have quantitative data on the SP induction and to confirm the role of orf33 in induction (Table 1, Figures 7 & S16). For qualitative data (yes/no induction), we used the semi-quantitative PCR for all the other tested conditions, which is a simpler method to know if there is a SP induction. We modified the figures for more clarity in the semi-quantitative PCR experiments.

5) My final main issue concerns the absence of proper quantification for the phage-mediated satellite induction and transfer. For instance, a qPCR-based quantification would be more informative than PCR gels (even if semi-quantitative). It would also benefit from using more replicates, since the gels only show one culture. Without this, it is difficult to properly compare the effect of satellite induction across phages (and in the absence of phages, where the satellite is also supposed to induce). For instance, the D4752 phage seems to induce less, but it is not clear. Also, regarding the excision of the satellite in strain DGCC7710wt (Figs S8-S11), it is unclear if the primers are specific to SP-310, or to native satellites of this strain. The native satellite does seems to excise when strain is exposed to some phages (Figs S10-S11). Moreover, before (lines 133-134) it was mentioned that the satellites excised in all strains tested, including strain DGCC7710 (line 129). Here (Figs S8-S11) no excision seems to be detected in absence of phage (but maybe because the primers are not appropriate? As I mentioned before, it is not clear).	Numbers were added for the frequency of transformation.<br><br>Semi-quantitative PCR were performed when a yes/no answer was needed and not for quantification purposes.<br><br>Biological replicates (at minimum, n=2) were done. The gels did show multiple points over time.<br><br>Figures were reworked for clarification purposes. For example, excision was quantified using Illumina sequencing when Orf33 is overexpressed (Figures 7 and S15).<br><br>As for the primers, cartoons were added to localize primers on the SP regions.
For the phage-mediated transfer itself, my comments are similar. There is little data beyond a PCR and a plating assay, and no information about replicates (it seems to come from a single culture?). How many times was the infection/transfer attempted, and how many times did it succeed? Are there differences in the numbers of colonies with an integrated satellite when using lysates of phage with and lysates of phage without the orf33 mutation? Can the authors provide a measure of the efficiency of transfer of the satellite via the phage, and assess how does this efficiency relates to the transfer of the ligated product of the satellite (is it more/less)?	More information was added. Per the scientific method, experiments were performed multiple times. Numbers were added.<br><br>Colony numbers are now provided in conditions mimicking natural settings Figure S17 and the transformants selection with phage 2972 (Figure S9).
- There is a discrepancy in describing the completeness of genomes in the different parts of the text. In the main text (line 92 and 94), and in the methods (line 407) the 86 (or 87, text and methods differ in the number) genomes are referred to as complete. However, in lines 151-152 it is mentioned that "... some of these S. t. genomes were incomplete". Is this a different dataset? If not, please clarify whether the genomes are complete or not.	The text was clarified in all these sections. Complete genomes were used for SP ID.<br><br>However, the spacer database was made using complete and incomplete genomes. See Table S2 for this discrepancy.
- I am intrigued by the presence of CRISPR spacers that target the satellites. It is not immediately clear to me why this would happen. If satellites act as anti-phage systems, why would they be actively targeted by the bacteria? The authors themselves mention that "... the presence of SP likely confers a benefit to the host cell" (lines 389-390). It would benefit the manuscript to hypothesize or discuss a bit this point. An additional piece of data that could help understand why this is the case would be to identify which genes are actually targeted by the CRISPR spacers (maybe there is an overlap with phage-encoded genes)?	CRISPR-Cas can acquire spacers from invading DNA such as SP. Considering that CRISPR-Cas systems are very active in *S. thermophilus*, it is no longer surprising to see spacers targeting SP as we clearly show here that *S. thermophilus* SP can be moved from one strain to another. It should be reminded that cutting any SP region will lead to selection of a SP-free cell as we did in *S.t.* DGCC 7891 (Figure

	S4). Thus, the targeted gene has limited relevance here. Also, there is no homology between *S.t.* phages and SP (Rezaei Javan et al. 2019). The presence of SP may also have a fitness cost.
- Related to the previous point: is the spacer targeting the CMP gene (lines 221-225) already found in the original genomes of the strains? Or was it artificially added to the genome in order to make a proof of principle that the CRISPR system is able to block the infection by the satellite?	There no spacer targeting the CAM gene in the original strain. Indeed, it was artificially added to the genome to make a proof of concept that the CRISPR-Cas can block the transfer of SP.
- The induction of the satellite. The authors found that it could be spontaneously induced, or induced by virulent phages. Beyond line 389 (which refers to S. thermophilus as a whole group), I couldn't find any data or statement saying that there are no prophages on these strains (that could also serve as their helpers).	There is no prophage in the strains SMQ-301 and DGCC7710. This was added in the manuscript.
- Lines 281-286. I was a bit lost with this rationale. Why would the double amplification on a satellite- free strain mitigate the signal of the induced satellite?	We understand the issue. Virulent phage D5691 induces the SP of strain DGCC7710, leading to a background of induced SP. Thus, we have amplified phage D5691 on the SP-free strain to remove the background in the lysate. However, to improve the overall clarity in the paper, we decided to remove phage D5691.
- In FigS7, it is not clear what I am looking at in the lower panel. The primers are for the genomic region without SP-301? If so, it seems that in 8 out of 10 colonies that were infected (and survived) phage infection the SP-301 was absent/lost?	This is a dynamic state. SP can be excised from the genome or lost from cells within the population. The various levels of PCR products illustrate the dynamics. The legend of Figure S10 was modified for clarity. We also provided Figure A below, that shows the SP dynamics (excised and integrated) in SMQ-301 during different growth phases.
Lines 146-147: Sentence has as strange formulation (starts with "... since this bacterial species..." but has no conclusion). Data used to support this only comes later in the paragraph. I suggest a re-write of this paragraph to avoid confusion.	Yes, sorry. Modified per comment from another reviewer.
Lines 205-207: Why do the transformation results align with what is shown in SaPIs? I agree that the spontaneous excision could be compatible, but as far as I am aware, all the studied SaPIs require a phage to be transferred.	The text was modified to improve clarity.
Line 230: "It was proposed that specific SPs may encode antiviral systems". Please specify in which ones this was shown (P4, PICI...), as throughout the text "SP" refers to the satellites studied here, and it might confuse the readers.	Sentence was clarified and a reference is provided for the reader to explore further.

Some references are strange, e.g., Line 97, reference 25 is used to support the similarity between PICI and and the satellite in this work, but ref 25 discusses that PICIs encode immune systems (maybe ref 7 or 10 would be more appropriate here?)	We modified the references accordingly.
Line 258, why is ProphageHunter an appropriate reference here?	Reference was removed.
Line 274, why is a reference to the description of a temperate phage in Francisella relevant here?	Reference was removed here but kept in M+M section. We modified the protocol from this paper to conduct the semi-quantitative PCR.
Some supplementary material seems to be inappropriately referenced. For instance, line 100 references Table 1 for the existence of two satellites in one genome. Since Table 1 has the read depth of plasmids used in an experiment from somewhere else in the manuscript (which should arguably be supplementary), I suspect the authors mean Table S1 here? Also, in line 130, should be Figure S2, I think. And line 421 cites Table 1, but it should be Table S1 as well.	Agree. Modified accordingly.
Line 47: "hijack a portion of the viral assemblies of certain temperate phages". Not necessarily the case, as PLEs and PICMIs hijack virulent phages.	Agree. "temperate" was removed.
"... markedly diminishes the replication and the release of new temperate phages". Again not necessarily so. cfPICI and PICMI were observed to have a very low (if any) cost for the hijacked phage.	"temperate" was removed.
Sometimes S.t. is used, other times S. thermophilus (e.g., line 127, 184, 194, etc)	We kept *S. thermophilus*
CRISPR1 and CRISPR3 (line 150) correspond to the CR1 and CR3 mentioned previously (line 74)?	We kept CRISPR1 and CRISPR3.
• Line 21: "can excised"	Modified accordingly
• Line 70: "can infect and lysed"	Modified accordingly
• Line 74: "adaptative" (not really a typo in the strictest sense, but "adaptive" is a more common term)	Modified accordingly
• Line 75: "30-bp (spacer)". Not really sure what was meant to be written here. Sequence? Spacer without the parenthesis?	Spacer without the parenthesis. Modified accordingly
• Line 84: "... structurally similarities..."	Modified accordingly
• Lines 84-85: "... phages can triggered..."	Modified accordingly
• Line 114: ".. Belonging to the Javan289-like possessed..." -> Javan289-like group?	Modified accordingly
• Line 127: "To confirm if the activity of SP in S. thermophilus strains, we performed..."	Modified accordingly
• Line 167: "design" -> designed? to design?	Modified accordingly: designed
• Line 187: "... through this transformation process." -> throughAtransformation process (or simply "through transformation")	Modified accordingly: through transformation
• Line 244: "... as describe earlier,..."	Modified accordingly
• Line 250: "... introduced inti a new host..."	Modified accordingly
• Line 390: "... the presence likely confer..."	Modified accordingly
• Line 392: "... enabled us to use of virulent phages..."	Modified accordingly

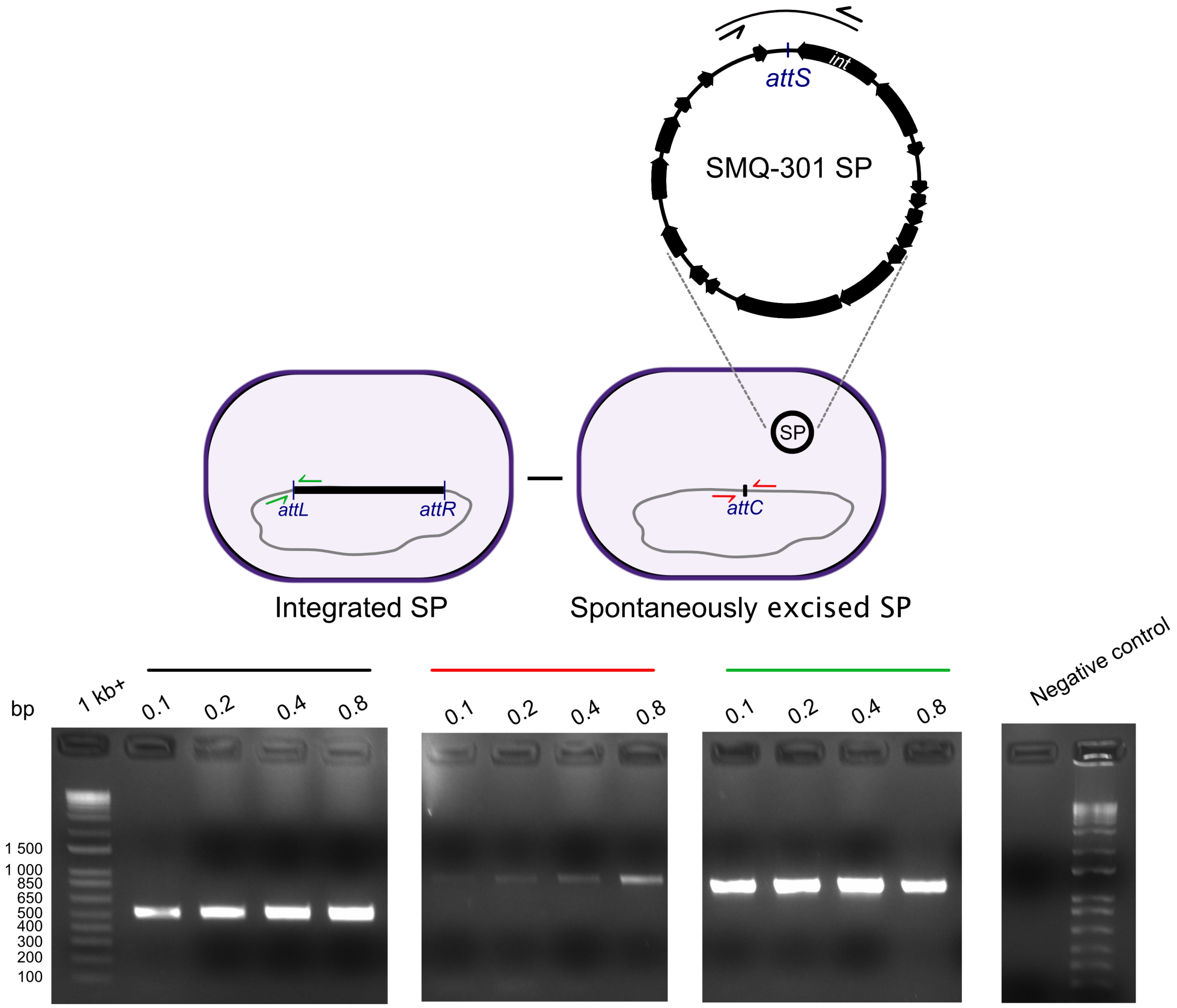

Figure A: Detection of the excised and integrated SPs in the strain *S. thermophilus* SMQ-301 in different growth phases (O.D.$_{600nm}$ of 0.1, 0.2, 0.4, and 0.8). The circularized SPs were detected by PCR using primers that are specific to both SP ends. The unoccupied attC site was detected using primers that are binding to flankings genes in the bacterial genome, and the integrated SP was detected using primers binding on one SP end and its corresponding flanking gene in the bacterial genome. Integrated SP can excise from the chromosome without any phage infection in different growth phases.

Reviewer #1 comments	Our answer	Lines
A major problem of the study is that the authors only assess induction semi-quantitatively based on OD/CFU estimates and do not relate quantity of SP relative induction levels to a chromosomal gene. While this data suggests that more attS and attC sites are formed, it is difficult to gauge how complete this is. All evidence is PCR-based and at no point do we see evidence of induction of the whole SP. I would have expected to see evidence of the spontaneous induction of SP in Figure 7, for example through increased read coverage across the SP-301 region compared to the surrounding chromosome. This is not evident from the current representation.	We have now conducted an Illumina sequencing experiment with different timepoints during phage infection to monitor the induction of the whole StCI over time (Figure 6). Of note, the spontaneous excision is rare as we observed in our new qPCR experiment (Supp. Figure 9). We also conducted qPCR experiments to quantify the StCI induction (Supp. Figure 17).	268 – 296<br><br>370 – 384
There is also no attempt to confirm that the full length of the SP is excised and replicating. This would have been valuable data to support the ability of virulent phages to induce SPs completely and is applicable to many of the experiments that were performed using PCR.	In the above Illumina experiment (Figure 6), we have now observed a significant increase of read coverage in the whole StCI area over time when induced by the wild-type phage, indicating excision and replication.	370 – 378
Nomenclature of SP: The authors are trying to establish that virulent phages can induce these chromosomal islands, which they call satellite phages. It would be better to use a nomenclature that has been established in the field of phage-inducible chromosomal islands as indicated by reviewer 2. “The phage satellites described and used in their analyses were previously annotated as a phage satellite by Javan et al., and idenFfied as PICIs by De Sousa et al., importantly these were classified as Type C, which depend on packaging proteins from their helper phage to mobilise. I would like to encourage the authors to follow the nomenclature already establish in the field, e.g. for E. coli is EcCIn, where first lemers are referencing the specie, “CI” for chromosomal island, and “n” would be the specific strain containing the PICI.” Introducing a new independent nomenclature would complicate future cross referencing and complicate further analysis.<br>“Satellite phages can be mobilized in other strains via natural competence” and “SP mobility during phage infection”	We are now using the suggested nomenclature. We used StCI (*Streptococcus thermophilus* Chromosomal Island).	From line 101
The authors use PCR amplified and ligated SP in the experiments to assess mobilisation of the SP through natural competence (Figures 4, S5, S6). While they succeed in showing this, its relevance is not clear at this stage. In the end, this just reflects the uptake of any circular DNA into a naturally competent host. It becomes clearer once they perform their phage infection and lysate transformation experiments.	The purpose of the experiment was to show that the entire StCI DNA integrated in its specific integration site (*attC*) in another strain. This experiment was also necessary to quantify the phage resistance phenotype in Figure 5 (and Supp. Fig. 10). In addition, transferring new DNA into industrial strains requires careful consideration as the use of genetically engineered organisms (GMOs) has generally a negative perception to consumers. The European regulatory authorities for example do not classify strains	

	as GMOs when genes are mobilized via transduction, conjugation, or *transformation*, provided the genetic material originates from the same species as that of the recipient strain. Thus, transferring a StCI from one strain to another through natural competence to enhance phage resistance would not be classified as a GMO. Its relevance is of significance.	
How are these SPs released? Is phage infection required? How much is released in a normal supernatant by spontaneous induction? Is this enough to transform via natural competence? I would suggest combining this section (or have consecutive sections) with "SP mobility during phage infection". It might make be more straightforward to show that lysates contain transformable DNA and then provide the in vitro ligated PCR product data to confirm the type of DNA (i.e. circular DNA is required). It would fit at the end of the results to give biological relevance.	We have redesigned and redone this experiment to address these questions. The number of StCIs released by spontaneous induction in the supernatant of the non-infected control (no phage) is not enough for the StCI transfer in our experimental settings. Also, when using the phage 2972-orf33-mutant lysate, we obtained no colony that had a StCI-specific integration. We only obtained colonies that had a StCI-specific integration when using a wild-type phage lysate. Therefore, a StCI-inducing phage infection is required in our conditions to have transformable DNA that can be mobilized in another strain through natural competence (not through transduction). The DNA in the ligation product from the *in vitro* experiment could be circular and/or concatemeric, we clarified this section.	258 - 261<br><br>442 - 476
However, one important aspect that needs to be addressed here is the impact of the orf33 mutation on phage fitness. How can the authors be sure that the reduced number of transformants with this mutant is not due to fitness impacts? What were the phage titres after amplification for both? The materials and methods section states that 50 ul of lysate were used in the experiment. Given the lower phage titre and fitness impact of the orf33 mutant, could it be assumed that less DNA is released in the cells infected with mutant lysates compared to those infected with the wt strain? There is no control to monitor release of bacterial DNA as a reference for the general efficiency of DNA release. How can the authors be sure that they are providing the same quantity of bacterial lysate for both phages?	We redesigned and performed another experiment to address these issues. We infected strain DGCC7710::StCI-301-CAT with the wild-type phage 2972 or the phage mutant 2972-Orf33-mut in the same conditions (same bacterial culture, same OD_{600}, and same MOI) and we measured the final phage titers. The phage 2972-Orf33-mut had a higher titer than the wild-type phage. Thus, we transformed 30 µl of each lysate (and the non-infected supernatant as a control) and we added a condition where we transformed with the same number of phage 2972-Orf33-mut and wild-type phage 2972 in the 30 µl of lysate. We also added a control to monitor the release of bacterial DNA and no colony was obtained. When using the phage 2972-Orf33-mutant lysates, we obtained no colony that had a StCI-specific integration. We only obtained colonies that had a StCI-specific integration when using the wild-type phage 2972 lysate.	442 – 476<br><br>891 - 931
"SP-301-CAT is induced by a phage-285 encoded protein"<br>The authors show that infection with virulent phages can result in an increase in attS sites, indicative of excised and circularised SP. This is	While conducting the original semi-quantitative experiments, the phage titer of phage 2972 mutant was too low.<br><br>We have now been able to purify and concentrate the phage Orf33-mutant (as well	370 – 378

promising but requires further verification and supporting data (see below).<br>Another important consideration is how the ofr33 frameshif impacts the inducing phage. If the phage is severely impacted in terms of replication, phage titres and/or plaque sizes, loss of induction might no be due to the loss of orf33 but caused by lower overall phage reproduction and consequently lower levels of other phage proteins. From the data provided, the orf33 phage mutant is severely impaired in its replication when infecting a strain containing a non-induced SP (DGCC7710) with an almost 2-log reduction in phage titres compared to the wt phage. This alone could explain an inability of the phage to induce the SP and consequently for the uninduced SP not to provide protection against phage infection. As clarified in their response, the authors use a constitutive expression vector to drive expression of orf33. This results in the complete loss of SP sequence coverage (Fig. 7), which the authors attribute to rapid loss of the SP after induction. However, my previous contention remains unaddressed. While it is possible that orf33 induces the island, this experiment provides no conclusive evidence for this but rather only allows to conclude that expression of orf33 wt is detrimental to the presence of the SP. Is this why, whenever the authors are using this plasmid, they never show reconstitution of the attS site and focus on attC only? A better and necessary experiment to address this might have been to either infect the SP containing strain with the wt or orf33 mutant phage and monitor excision and replication over a time course following infection (including a sample before adding the phage) via Illumina sequencing. This would have shown that orf33 was important for induction of the SP.	as the wild-type phage) and conducted the requested Illumina sequencing (Figure 6). By doing so, we have showed that Orf33 is indeed essential for the StCI induction.	
To further corroborate this or as an alternative experiment, a tightly controlled, inducible-expression plasmid would have better allowed to monitor induction following orf33 expression. Again, a time course of induction would have been appropriate and could either be monitored by Illumina sequencing or Southern blotting.	To our knowledge, there is no tightly inducible-expression plasmid available in *S. thermophilus*. However, our results from the Illumina experiment clearly show that Orf33 is essential to the StCI induction (Figure 6). We have also conducted a new experiment that show the interaction between Orf33 protein and the Stl protein of StCI-301 (Figure 9).	370 – 378<br><br>432 - 440
The experiment showing that the regulatory region of SP-301 participated in the induction process is elegant. It clearly showed that a phage protein was targeting this region. However, infection with the orf33 phage mutant was not performed and thereby a crucial piece of evidence is missing. Again, only restoration of the attC site is monitored when expressing orf33 from a constitutive promoter not allowing any conclusion of whether it is responsible or only detrimental to the SP. Could the	We have now shown that the Orf33 is binding to the StCI-301 Stl regulatory protein and our qPCR and Illumina experiments show that Orf33 is essential in the StCI induction (Figure 6 and Supp. Figure 17). These results corroborate with the loss of the StCI-301 when Orf33 is constitutively expressed and inducing this StCI (Supp. Figure 18).	432 – 440<br><br>370 – 395

authors use a reporter plasmid to monitor the induction of replication genes to confirm the role of ORF33? This would also require the inducible expression or the protein to monitor induction.		
"SP mobility during phage infection"<br>Satellite prophages or PICI are normally packaged into phage particles when induced and can be transferred via transduction. Have the authors evaluated this? Their methodology relies on filtering lysates, which would not remove any phage particles. To exclude that any form of transduction plays a role in their observations, this should be confirmed.	To create a generalized transduction control, we first added a chloramphenicol resistance gene (CAT) into the StCI of *S. thermophilus* strain DGCC7710. We amplified phage 2972 on this strain and on the strain DGCC7710::StCI-301-CAT (a strain engineered to contain the StCI from strain SMQ-301). Then, we conducted a transduction experiment with these lysates to infect DGCC7710-ΔStCI-ΔRSR and UY03-ΔStCI-ΔRSR, two strains engineered to lack a a StCI. We obtained no colony in the UY03-ΔStCI-ΔRSR conditions. We only obtained one transductant in DGCC7710-ΔStCI-ΔRSR using the lysate of phage 2972 amplified on DGCC7710::StCI-CAT (our "positive generalized transduction control"). Thus, we do not have an efficient positive control to conduct a transduction experiment, but still no colony was observed in the recipient UY03-ΔStCI-ΔRSR strain. However, in the transformation experiment using phage lysates, we have done controls containing phages and bacteria without adding the ComS peptide (which is essential for the DNA entry in the cell by natural competence). We obtained no colony in the no-ComS conditions; thus transduction is not involved in the StCI transfer in our experiment. We also showed that phage 2972 does not adsorb to the strain UY03-ΔStCI-ΔRSR (Supp. Figure 22).	442 – 476<br><br>891 - 931
The authors state that "The virulent phage 2972 does not adsorb to the receiving strain UY03 thereby eliminating the transduction possibility." However, no evidence for this statement is provided.	We have added the Supp. Figure 22.	
I would like to see confirmation of whether SP-301 DNA is packaged into phage capsids or not.	We conducted a transduction experiment using a virulent phage 2972 lysate amplified on *S. thermophilus* DGCC7710::StCI-301-CAT. We added 1.8 x 10^6 pfu to 5.1 x 10^7 cfu of the strain DGCC7710-ΔStCI-ΔRSR (containing a marker to differ from cells in the lysate) and we got only one transductant. This very low frequency (0.00006%) may be generalized transduction.<br><br>We had a control using a phage 2972 lysate amplified on strain DGCC7710::DGCC7891-StCI (with an erythromycin resistance gene). There was no phage resistance, so it is unlikely that this	

	phage packaged specifically the StCI of DGCC7891 and we had a higher phage titer. We used 1.4 x 10^8 pfu and we got 86 colonies, which represents a transduction frequency of 0.00006%, which is the same as in the 2972 lysate amplified on DGCC7710::StCI-301-CAT with more than 4 logs of phage resistance. Therefore, we do not think that the phage resistance phenotype is related to the encapsidation and the very low transduction frequency is probably generalized transduction, since phage 2972 is a *pac*-type phage.	
Tables:<br>Please ensure consistent nomenclature of all strains used in the study and verify that all strains that are indicated in the manuscript are indeed present in the relevant table. Currently, several key strains are either missing or labelled inconsistently between the text and the table.	Modified accordingly. We used StCI (*Streptococcus thermophilus* Chromosomal Island).	From line 101
Figures:<br>General comments on figures and supplementary figures:<br>The use of colours and line thickness as well as figure sizes is not very accessible. Colour coding for the different PCR products throughout the manuscript is extremely hard to see and the chosen colours are not colour-blind friendly. Only using large zoom on the pdf could colours be guessed at. Please rework this in future submissions according to the guidelines specified by reviewer 2 (https://www.nature.com/articles/d41586-021-02696-z).	Modified accordingly.	
-Figures should have a descriptive title before launching into experimental details.<br>- Please consider the use of panel labels (a,b,c, etc.) this would greatly help with identifying the relevant sections between figures and text.<br>- Please check labelling of all figures. There are multiple instances where there are labelling errors in both main and supplementary figures.	Modified accordingly.	
Figure 1 is much improved, and I am happy with the changes together with Figure S1.<br><br>Figure 2 is still missing the relevant controls for integrated SP compared to the spontaneously induced SP.<br><br>Figure S16 does not include any of the required information and relates to a different strain set.<br><br>I appreciate that the authors have evaluated spontaneous induction of one of their SPs in other growth phases. This data should be included with the main manuscript and not only as a response to	The purpose of Figure 2 was to show that StCIs can excised from the chromosome and the sequencing of the excised PCR product allowed us to identify the *attS*, *attL,* and *attR* sites. We cannot compare the excised vs integrated StCI only based on the intensity of PCR products, since the binding efficiency may differ between primer pairs. However, we added the requested conditions in Supp Figure 4 and we conducted qPCR experiments to compare the integrated vs excised StCIs, between different growth phases, in the three main strains mentioned in	

the reviewers. Why was this done for only one of their SPs? How was data normalised? Is the data they are showing due to bacterial growth rather than real changes in excision?	this paper: SMQ-301, DGCC7710, and SMQ-1512. (Supp. Figures 8 and 9).	
Figure 4: SP-301 appears to be far less stable in this strain background. There is also a considerable proportion of attC still present suggesting that the island is not fully integrated. Does the cat gene cause this?<br><br>Figure S6 suggests that there are differences in stability between different strain backgrounds receiving the SP-301.	As mentioned in previous answer, we can't compare the excised vs integrated StCI only based on the intensity of PCR products. It seems that StCI-301 is less stable in the SMQ-301 strain than the StCI-301-CAT in the DGCC7710 strain background (Supp. Figure 9). Interestingly, the DGCC7710-StCI seems more stable than the StCI-301 in their respective host. However, the StCIs and the bacterial background are quite different and cannot be directly compared. In DGCC7710, there is not a clear difference in the stability between the DGCC7710-StCI and the StCI-301-CAT. These StCIs are different and cannot be directly compared. The StCI stability could be more related to the strain context rather than the CAT gene (Supp. Figure 9).	
Figure 8: Please introduce panels to better label the different elements of the figure.	Modified accordingly.	
While there is evidence that phage infection induces the chimeric SP when using phage 2972, a complementary experiment using the orf33 phage mutant would support the role of the orff33 product in inducing SP-301 regulatory element containing SPs. Expression from a constitutive promoter is not a suitable system to show that a gene product is responsible for the induction of an SP. Here, only restoration of the attC site is monitored but no evidence for the presence/absence or quantity of SP is provided.<br>Top panels: What are the green circles meant to represent? Bottom left panel: should read "SP-DGCC7710 wt" not "DGCC7710 wt no-DNA control"	We have now conducted qPCR experiments with the chimeric StCI in infected conditions (wild-type phage 2972 and phage mutant 2972-Orf33-mut), and non-infected conditions. Our results clearly show that Orf33 is essential in the StCI excision (Figure 8). We have also clarified the panels.	403 - 413<br><br>874 - 889
Figure 9: What are the green circles meant to represent?	It is the Orf33 protein. The Figure 7 and Supp. Figure 20 were modified to improve clarity.	
Figure S5: attC controls only performed for delta SP recipient but not for the strains after transformation. No control for stability of the integrated island (attS). This was done for Figure 4 and should have been also performed for S5.	We have now done the requested controls (Supp. Figure 6).	
Figures S10: There is some error in the labelling of this figure. From the experimental description in the results section, both samples were treated with phage and the difference is the presence or absence of SP-301 DNA. Currently, the figure states that this was added to all samples.	Modified accordingly.	
Minor comments:<br>Line 146-148: "*The sequencing of the resulting PCR products also confirmed the attC sequence for*	Modified accordingly.	142 - 148

the three most common S.t. SP integration sites: ATATTTCCCTCTAAAATC (IS_a), ATTTCATGAAAAAATAC (IS_b), and AATTCTACAACAAAAT (IS_c) (Table S1).” This statement is missing context, i.e. “*As attS sites mirror attC sites, the sequencing of the resulting PCR products...”*		
Line 151: “*Because we could induce S.t. SPs and pinpointed the attC site,...”* This sentence is redundant as already covered in the previous sentence.	Modified accordingly.	146 - 154
Lines 169-170: “*Therefore, it is tempting to speculate that CRISPR-Cas may also serve as a defense system against SPs*.” Yes, but also this could be considered evidence that these SP can move between streptococcal species pointing towards their mobility.	We have conducted an additional analysis requested by reviewer 3, and we showed that some spacers are specific to StCIs (Table S2). Thus, there is evidence that StCIs have moved between streptococcal species. We have modified our conclusions accordingly.	172 - 185
Lines 190-192: “*By conducting spot test assays, we verified if the loss of the SP in DGCC7710-ΔSP::pNZ123-RSR, UY03- ΔSP::pNZ123-RSR, SMQ-301-ΔSP::pNZ123-RSR and DGCC7891-ΔSP::pNZ123-RSR strains could increase their phage sensitivity when compared to the wild-type strains containing pNZ123*.” There is something wrong with the nomenclature of the strain here. Do they really contain an integrated plasmid? Where are these strains in the strain table? Why did the authors assess the phage titres in strains with the plasmid? This is in contradiction to the previous paragraph where the plasmid was eliminated. Please correct/clarify both in the text as well as in the table.	We have modified the strain nomenclature to avoid confusion. They do not contain an integrated plasmid, they contain the RSR-plasmid. However, in this experiment, we used the strains before their plasmid loss, because the several passages without antibiotic pressure caused the accumulation of mutations in the whole genome. This section was clarified.	204 - 210
Lines 207-208: the authors could highlight that by amplifying from attL to just before attR, this would result in the complete circularised SP after ligation.	Indeed, we modified accordingly, but we also specified that the ligation reaction could also produce concatemeric StCIs.	223 – 226<br><br>258 - 261
Lines 219-221: *“In contrast, when the SP-301-CAT-rev linear PCR product was transformed into the ΔSP strains with an equivalent amount of ligated DNA from SP-301-221 CAT-rev, no colonies containing integrated SP were detected (data not shown).”* There is a reference missing: an equivalent amount of DNA relative to what?	The amount of linear PCR product was the same as the DNA used in the ligation reaction. We clarified the sentence.	236 - 239
Lines 273-283: The authors use an elegant approach to select for SPs providing phage resistance. By challenging a transformation assay either with or without the addition of circular SP-301, the effectively exploited the anti-phage properties of this island to select for successful transformation of the markerless SP. There are some issues with the annotations in the cited figure S10 (see figure section).<br>I would also have liked to see how these newly transformed colonies performed on a spot assay and whether they would show the same levels of phage	We have clarified the annotation. We have tested, in a spot test assay, three purified transformants selected using phage 2972 and they all have a phage-resistance phenotype of approx. 5 logs compared to a colony that was not selected with phages. However, based only on this experiment, we cannot be certain that the StCI is solely responsible for the observed phage-resistant phenotype, since the phage interacts with other cell components. Thus, we cannot really compare this phage-resistance phenotype with the	

protections as the marked chloramphenicol-resistant SP.	chloramphenicol-resistant StCI strain that was not in contact with any phages. This experiment only shows that phage 2972 selects StCI+ cells and we addressed the StCI phage-resistance effect in other experiments (Figure 5 and Supp. Figure 11).	
Line 308: "*Surprisingly*" I am not sure why this is surprising, please remove. Additionally, while overall colony numbers obtained are stated in the figure legend and text, there is no reference as to how many colonies/PCR products were Sanger sequenced to confirm attR and its flanking regions.<br><br>Lines 404, 410 &416: How many colonies/PCR products were sequenced for each?	All the PCR products in the Figure were Sanger sequenced. Modified accordingly (Supp. Figure 23).	

Reviewer #2

**Reviewer's comment**	**Our answer**	**Lines**
Thank you for performing this analysis. As confirmed by Sousa et al. these elements can be classified as PICIs. I would like to encourage again to follow the nomenclature and use it throughout all the manuscript and not just as a brief mention.	We have changed the nomenclature in the manuscript accordingly.	From line 101
However, these PCR products were performed on the att sites and replication is still require for a succesful transfer. Therefore, either southern blots or qPCR probing a gene in the PICI will be necessary. For qPCR a chromosome gene can be use as reference for normalising data.	We have now conducted an Illumina experiment to follow the entire StCI replication over time when induced by phages (Figure 6 and Supp. Figure 17). As suggested, we have also conducted a qPCR experiment to monitor the replication of the DGCC7710-StCI containing the StCI-301 regulatory *orfs* when induced by phages (Figure 8).	370 – 384<br><br>403 - 413<br><br>874 - 889
Indeed, SP can be spontaneously induced as observed with the PICIs found in S. pyogenes and strains with PICIs can lack helper prophages as seen with some S. aureus and S. pyogenes isolates. I apologise if my response was suggesting that these elements can only be induced by the resident prophages. My point here is that to demonstrate that these elements are phage-inducible, or not, extensive replication is necessary to be proven. This mechanism grants the element with many copies of their DNA for a succesfull packaging/transfer and as seen with PICIs and other Satellite. Please avoid using responses as "friendly reminder". We all do our best on granting a fair review.	We have now proven the induction and replication of the StCI-301 by phage 2972 (Figure 6 and Supp. Figure 17).	370 – 384
Thank you. Based on this analysis we can see that the lytic phages can induce the PICI to some extend. And this shows increased excision. However, these PCR products were performed on the att sites and replication is still require for a succesful transfer. Therefore, either southern blots or qPCR probing a gene in the PICI will be necessary. For qPCR a chromosome gene can be use as reference for normalising data.	Thank you for your comment, we have now conducted the requested qPCR experiment and the StCI-301 is induced (excision + replication) by phage 2972 (Supp. Figure 17).	370 – 384
Thank you for performing these. Indeed it seems that the PICIs are not able to be transfer by these phages. My main point here was to show that these PICIs are transfer by the phage as seen with other satellites, like cfPICIs and PICMIs, who don't necessarily interfere with their helper, but they are able to encapsidate and transfer to other host using the viral capsids. It would be interesting to see if phage capsids can transfer these elements.	We did not observe StCI-CAT mobilization through transduction in our transformation experiment using phage lysates. To create a generalized transduction control, we first added a chloramphenicol resistance gene (CAT) into the StCI of strain DGCC7710. We amplified phage 2972 on this strain and on strain DGCC7710::StCI-301-CAT. Then, we conducted a transduction experiment with these lysates to infect DGCC7710-ΔStCI-ΔRSR and UY03-ΔStCI-ΔRSR. We obtained no colony in the UY03-ΔStCI-ΔRSR conditions. We only obtained one transductant in DGCC7710-ΔStCI-ΔRSR using the lysate	442 – 476<br><br>891 - 931

	of phage 2972 amplified on DGCC7710::StCI-CAT (our "positive generalized transduction control"). Thus, we do not have a very efficient positive control to conduct a transduction experiment, but no colony was observed in the recipient UY03-ΔStCI-ΔRSR strain. However, in the transformation experiment using phage lysates, we have done controls containing the phages and bacteria without adding the ComS peptide (which is essential for the DNA entry in the cell by natural competence). We obtained no colony in the no-ComS conditions; thus transduction is not involved in the StCI transfer in our experiment. We also showed that phage 2972 does not adsorb to the strain UY03-ΔStCI-ΔRSR (Supp. Figure 22).	
Regarding the transformation and induction by the 2972-orf33-mut, it seems that the phage titer is 100-fold reduced. This is likely affecting the induction and transformation assays as this is likely to impact the amount of cells being destroyed and then PICI DNA released for transformation. I find this part a bit problematic, and as also mentioned by the other reviewers, it makes the transfer less reliable by this mechanism.	We understand the issue that the lysate contents might differ. Thus, we have redesigned the experiment to consider the difference in the phage titers and we amplified the wild-type phage 2972 and phage mutant 2972-Orf33-mut. Titers were normalized.<br>Because there is a phage-resistance mechanism in the DGCC7710::StCI-301-CAT, the wild-type phage 2972 had a lower titer than phage 2972-Orf33-mut.	442 – 476<br><br>891 - 931
I find this argument a bit weak, since a big part of the manuscript focuses on the mechanism of induction-excision-replication-transfer, which is ultimately the proof to showcase a new member phage-inducible chromosomal island or to classify these elements as a novel phage satellite. As I mentioned above, it seems phages can indeed induce excision, but we are still lacking the confirmation of the whole satellite lifecycle.	We have now shown that the Orf33 is binding to the StCI-301 regulatory protein, through a protein-protein interaction (Figure 9).	432 - 440
This was shown in an application approach for engineering satellites by Ibarra-Chavez et al. BioDesgin Research (2020).	Ibarra-Chavez et al. have electroporated plasmids and did not use only the PICI as the DNA template or natural transformation. Of note, their work was done in *E. coli* and *S. aureus*, but not in *S. thermophilus.*	
As mentioned before, there is little prove of phage-mediated transfer or transformation as the differences on phage titer could have a great implications on the amount of DNA available for transforming. An MOI of 1:1 typically results in 63% of cells being infected, compared to MOI 1:10 resulting in 10% infected cells.	We have now redesigned the experiment to address these issues. Both phages were amplified on the DGCC7710::StCI-301-CAT in the same conditions using a MOI of 5.	891 - 931
Thank you. Please consider revising the manuscript once more as there are still figures that are labelled incorrectly.	Modified accordingly.	

Reviewer #3

Reviewer's comment	Our answer	Lines
The targeting of CRISPR-Cas to SPs. I will not argue against the existence of spacers that target the SPs, but I'm not sure I agree that knowing the DNA targeted by specific spacer is irrelevant. Some proteins of SPs have commonalities with those found in virulent and/or temperate phages (Integrases, TerS, etc). Hence, a CRISPR spacer that was acquired from a phage might target a SP as a collateral effect. On the other hand, if they target SP specific genes (like stl or the equivalent regulatory protein in the StSPs), then this would be supporting evidence that bacteria are acquired spacers directly from the SP. The reason I insist on this is also because it would be nice to have some discussion on why would bacteria actively try to prevent infection by SPs that can provide an advantage (by protecting against some phage). Since the authors already have identified the spacers, the targeted DNA (which genes, from which SPs and from which strains) should be easy to pinpoint and present, and I think it would add a nice eco-evolutionary component to the paper.	Thank you for the clarification.<br><br>We have now specified the region in the StCI targeted by the spacer and conducted BLAST analysis to verify if the spacers are found in other genomic contexts (see Table S2).<br><br>By aligning each spacer with the NCBI BLASTn database, we identified three spacers that target specifically a SP region, as they are not found in other genomic contexts.<br><br>"Taken altogether, our results suggest that some spacers are specifically acquired from satellite phage DNA that can be mobilized between different *Streptoccocal* species."	172 – 185<br><br>538 - 541
- On the point of mobilization by transformation, I am now more convinced that is the case. I apologise that I missed the data on mobilization under more natural conditions on Figures S17 and S18 in the previous version, but it was indeed not very clear what was being tested. Nevertheless, I am still a bit puzzled on the particular experiment that uses the artificially ligated SP. I understand that this is meant to provide a laboratory-specific assay to test the concept of the mobilization by transformation. As I was reading the new version, I initially assumed that this would be because the efficiency of transfer by natural transformation using just cell lysates would be too low to detect. But the SPs do circularize by themselves, and if I understand correctly the data in the last section of results (Figure S18), transfer under natural conditions (without phage induction) occurs on average in 12.5% of the cases, which is very reasonable. Thus these conditions should be enough to generate enough SP DNA and detect the transfer, especially if there is the CAT gene to select on. So the need to add a level of artificiality using the ligated form is still not clear to me. Perhaps the authors can just add a brief rationale at the beginning of this part (lines 207-208) that could help the readers follow the reasoning for their methodological approach?	We wanted to have an experiment, in which only the StCI DNA is mobilized by natural competence to show that the entire StCI DNA integrates in its specific integration site (*attC*) in another strain. This experiment was also necessary to quantify the phage resistance phenotype in Figure 5 and Supp Figure 10.<br><br>We have now transformed the supernatant from a DGCC7710::StCI-301-CAT culture into UY03-ΔStCI-ΔRSR and we obtained no colony, thus it might not be easy to transfer StCI this way in laboratory conditions.<br><br>Indeed, based on experiment from our previous version of this manuscript, the DNA from an overnight culture of the strain containing the StCI-CAT could have been a successful DNA template to mobilize the StCI-CAT to other strains. However, during natural competence, all the DNA with homologous regions can recombine with the recipient strain DNA. Thus, the genomic DNA from the overnight culture could recombine with the DNA of the recipient strain, making it highly variable and unsuitable to properly verify the phage resistance phenotype.	219 - 242<br><br>442 – 476<br><br>891 - 931
Even if it has a technical reason. I would also like to read some justification on why two orientations for	We started working with the CAT-rev to mobilize the StCI in four strains, but we	

the CAT gene were used, as well as some speculation on why it gives different results, given that only (?) the CAT-rev version worked in all four strains. Maybe some of these points could be more strongly addressed by including the quantifications of the efficiency of transfer here (and not just in the last experiment). If colonies were selected, there must have been colonies to count. Are the number of CAT resistant (and thus SP containing) colonies obtained with the ligated version that much higher? And are there differences between the CAT and CAT-rev efficiencies?	switched to the CAT, since the gene is in the same orientation as the other StCI accessory genes. Thus, we transformed the StCI-CAT in strain DGCC7710 for the other experiments, we did not try in other strains, since we already showed the specific SP mobilization with the StCI-CAT-rev. Since our ligation experiments were not done within the same conditions for the StCI-CAT and StCI-CAT-rev, we can't compare their colony numbers and there could be a lot of variations in the amount of the different DNA conformations between the ligation reactions. The use of the two orientations of the CAT genes did not affect the phage resistance phenotype (Figure 5 and Supp. Figure 10).<br><br>We clarified this section in the manuscript and added the colony numbers related to the mobilization of the excised StCI from the (new) last experiment, which is mimicking natural settings and comparing the StCI transfer from inducing, non-inducing, and spontaneous induction conditions.	
Considering the phage resistance provided by the SP, I think it is quite convincing now. What is also interesting to me is that SP-301 provides no protection against the phages that infect its own host SMQ-301 strain (73 and DT1, lines 194-195), but it does provide resistance against phages that infect other strains. This to me suggests that some of these SPs might not only travel frequently between hosts, but might also undergo selection across multiple hosts as well (and the phages they encounter). If the authors agree, maybe some discussion could be added on this point?	We agree. Discussion was added.<br><br>"These findings demonstrate that CRISPR-Cas9 can effectively facilitate the recovery of *S.t.* cells without any StCI, and it also suggests that the phages might have evolved to bypass their host StCI phage resistance mechanism, since the presence/absence of these specific StCIs did not change the phage sensitivity profile of their host."	211 - 214
Finally, regarding the spontaneous excision of SPs. The authors suggest, in the conclusion, that SP can be lost naturally within a population ("*While the spontaneous SP excision suggests a scarcity of these MGE within the bacterial population, especially if the SP fails to reintegrate the chromosome before cell division*"). However, it would be beneficial to provide a point of speculation on whether the re-acquisition by transformation could counter-act this loss. Note that it would be extra nice to have some numbers too for this process of loss (how frequent it is) and this would add an important information for this process. But I understand it might fall out of the scope of the paper.	It is a good point, and we added the re-acquisition hypothesis in our conclusion. We added qPCR experiment that quantify the relative amount of empty *attC* site vs excised StCI vs integrated StCI in three different strains (Supp. Figure 9), but we cannot know if the element is lost in one cell, since the excised form can still be present even if there is an empty *attC* site. However, our qPCR results showed that empty *attC* sites (= no integrated StCI) are extremely rare in the three strains tested (Supp. Figure 9).	284 – 296<br><br>491 - 492
- Line 98: "... seemingly functional SP..." -> Not wanting to be annoying, but I still think there is no obvious evidence (at this point in the text) that it is "functional". I would just say "a putative SP".	Agree. Modified accordingly.	89

The SMQ-310 and DGCC7710 strains have no prophages, as stated by the authors. Maybe I missed it, but what about the other strains from Figure 2? Can you state from early on (e.g., lines 138-139) whether this is the case or not?	We have now specified if prophage are detected in the genomes available on GenBank.	136 - 137
Line 161: "... or they have a different SP than the ones targeted by the spacers". Meaning a different group, or just different SPs in general?	A different StCI in general that is not targeted by the spacer. Text was clarified.	161 - 163
Line 196-197: Add *"... and that these SPs provide no protection against infection by the tested virulent phages*" Otherwise, the previous paragraph seems out of place. What is the rationale for testing phage-resistance here? Why no conclusion? I think this is supposed to provide a complementary point to the experiment on phage resistance later on, but here it seems a bit lost.	It was to verify if the StCI from the phages' hosts could provide phage resistance. We added a conclusion to clarify this point.	204 - 214
Line 207: Figure 4, instead of Figure 2?	Modified accordingly.	
- Lines 311-312: "*The orf33 is a non essential gene and it matched a putative..."* -> Has homology with?	Modified accordingly.	358 - 360
Lines 429-430: "*We found that swapping SPs enhance the overall phage resistance of a S.t. strain.* " -> I would tamper this a bit and just say that "swapping SPs CAN enhance the overall phage resistance". Given that the phage-SP interaction might be specific, some pairs swapped might not give any resistance at all (and only one proper swap was tested in the manuscript).	Modified accordingly.	493 - 494
Figure S18 is out of order in the supplementary data file (I thought it was missing at first!)	Modified accordingly.	

Reviewer #1 comments	Our answers
The revised manuscript represents a significant improvement. The authors provide compelling new evidence that virulent phages can induce *Streptococcus thermophilus* chromosomal islands (StCIs) via an interaction between the phage-encoded Orf33 and the island-encoded repressor. I am convinced that this interaction underlies island derepression, and the data in Figure 6, cassette swaps and EMSA, strongly support a conserved mechanism similar to that seen in other PICIs.	Thank you.
While I have no major concerns with the central conclusion, several issues remain with the presentation and clarity of the manuscript. Some previous reviewer comments are not addressed in the text or rebuttal, and aspects of the experimental design and interpretation remain unclear. I detail these below.	We are glad that the reviewer has no major concerns with the central conclusion. We hope to have improved the presentation and clarity of the manuscript.
The manuscript repeatedly refers to StCIs as "satellite phages." This term implies they are defective prophages, which may not accurately reflect their evolutionary origin. I recommend replacing this with "phage satellites," which avoids presuming origin while still describing their dependency on helper phages.	Modified accordingly. We are now calling them "phage satellites".
The authors show convincingly that virulent phages induce StCIs, but the manuscript does not address whether StCIs are packaged into phage capsids. While negative data may not be available, this omission should be acknowledged. The authors could include a short discussion of plausible reasons for lack of packaging. For example, absence of a recognised *pac* site or TerS functon (no terS gene is annotated), and whether the StCIs might be *cos*-type PICIs instead. Could other virulent phages with compatible packaging machinery enable StCI mobilisation? How likely is transfer via released StCI DNA post-lysis compared to classical transduction mechanisms?	We do agree and we added a discussion on this topic. See lines 513-528.<br><br>However, we cannot speculate on the StCI transfer likelihood in nature via released StCI DNA post-lysis compared to classical transduction, since our natural transformation data required the addition of the ComS peptide (which is naturally expressed in some *S. thermophilus* strains in some conditions) to trigger the natural transformation pathway in laboratory settings.<br><br>In our settings, in the last experiment using phage 2972 lysates, the StCI transfer was only possible with released StCI DNA post-lysis by natural competence and not by transduction.
Issues requiring clarification or revision<br>• Lines 204–210: The rebuttal notes that strains were used before plasmid loss due to the emergence of mutations during plasmid curing. This should be stated clearly in the	We have now stated in the manuscript (lines 212-215): *"Of note, we used the propagation host containing the pNZ123-RSR plasmid, before the plasmid loss, to avoid any potential effect caused by*

manuscript, along with whether the mutations affected phage resistance and, if so, their locations.	*the emergence of potential mutations during plasmid curing.*<br><br>We also showed that the phage resistance profile of DGCC7710::StCI-301-CAT is not caused by mutations from the bacterial background. Indeed, we restored phage sensitivity by removing the StCI-301-CAT (Figure S11). However, we did not sequence the UY03, DGCC7891, and SMQ-301 strains following the plasmid curing. This is the reason why we used the strains before plasmid curing.<br><br>The potential mutations are not affecting phage resistance in DGCC7710, since the wild-type strain, DGCC7710-delta-StCI + pNZ123-RSR and DGCC7710-delta-StCI-delta-pNZ123-RSR have the same phage titer (Figure 5).
Orf33 mutant titer: The rebuttal claims the orf33 mutant has higher titre than wild-type, but the manuscript states the opposite: wild-type phage yields 9.95 × 10^9 PFU/ml, compared to 2.27 × 10^8 PFU/ml for the mutant. Please clarify which is correct and ensure consistency between text and data.	In the rebuttal, we were referring to the last experiment on the StCI transfer. In this experiment, when amplified phages on strain DGCC7710::StCI-301-CAT and phage 2972 Orf-33-mut had a higher titer than the wild-type phage 2972, because of the StCI-CAT phage resistance mechanism interfering with the wild-type phage. On the wild-type DGCC7710 strain, the wild-type phage had a higher titer than the phage mutant.
Lines 268 ff. ("StCI excision and replication"): This section is difficult to follow due to insufficient description of the qPCR setup. It is unclear where primers bind, what exactly is being amplified, or what is meant by "excised StCI" in this context. Why does primase copy number not correlate with excised StCI copy number? A schematic showing amplicon positions (as in other figures) would help, along with expanded legends and clearer explanation of the biological meaning of these measurements.	We added the amplicon positions on the qPCR Figures (Figure 8, Supp. Figures 8, 9, and 17). We added more details in Figure 8 and Supp. Figure 17 to improve clarity.<br><br>In Figure S17, StCI-301 is induced by a virulent phage, and the qPCR targets are first normalized against reference genes and then to the non-infected control (ΔΔcq method). Upon induction by the wild-type phage 2972 (30 min post-infection), there is approximately a 23-fold increase of StCI primase levels compared to the reference genes and the non-infected control. This suggests that there is approx 23 StCI copies per cell following induction, compared to the single integrated copy per cell observed in the non-infected control. In the non-infected condition, the excised form of StCI is rare (substantially less than one copy per cell, as shown in Figure S9). Therefore, when excised, the StCI levels from the phage-infected samples (in which presumably all StCIs were excised) were normalized to those from the non-infected control, the resulting fold change appeared even higher than that observed for the primase. This reflects both the increase in copy number due to replication and the near absence of excised StCIs in the baseline condition. We do understand that it could be confusing, thus we added more explanations (lines 399-410) and a new Table S3 that contain the cq values of the qPCR

	experiments. The cq values of the excised StCI were similar to the primase values in the infected samples (with phage 2972 wt). We also included in this rebuttal two new figures containing the cq values only normalized to the reference genes (Δcq method) (Figures 1 and 2 at the end of this rebuttal). However, we would prefer to keep the ΔΔcq method (using a calibrator sample) in the manuscript, since it is a statistically more robust method and was recommended by Bio-Rad.
Figures S8 and S9: The differences between these two datasets are not explained clearly in either the figure legends or the text. Clarify what each figure represents and what distinguishes them. Please clarify whether fold changes are plotted on a linear or log scale, and if statistics were performed on log-transformed values, state so in the legend.	We added more details in the legends of Figure 8, Supp. Figures 8, 9 and 17. Fold changes are plotted on a linear scale and the statistics were performed on log_2-transformed values. In the main text, we clarified the difference between the two datasets in lines 291-297.
Lines 294–296: These two sentences contradict each other. Please revise for clarity.	We clarified this point. We replaced "induction" by "excision". There is spontaneous excision, since we can detect excised StCI and empty attC sites in overnight cultures without phages. However, this is a rare event as the StCI *pri* levels are not significantly higher than the bacterial genes. Thus, StCI replication might be low or absent. We added this statement: *"Thus, the StCI replication was not detected in overnight cultures for all the three strains, indicating that there was no StCI replication or the replication levels were too low to be detected. Thus, StCIs can be spontaneously excised, even in a new host, at low excision levels, and independent of the growth phase."* See lines 304-307.
Lines 403–421: As above, a schematic showing the positions of primers and expected amplicons would greatly improve this section's clarity.	Modified accordingly. See Figure 8.
Figure-specific feedback<br>• Figure 1: The "phage resistance protein" annotation appears speculative and is not supported elsewhere. Based on genomic context, a primase seems more plausible. Please revise or remove accordingly.	We specified that all the annotations are from the original GenBank files. We have now removed the "phage resistance protein" annotation from the figure and added more details in the figure legend.
Figure 3: While colour coding has improved, the colours may still be difficult to distinguish when reduced to journal page size. Consider using distinct patterns or line types to enhance clarity.	We have now tried to use a more distinct color pattern, and we increased the size of the primers in Figures 3 and 4.
Figure 5: There appears to be mislabelling of some strains in the figure or legend. Please check that all labels match and are correctly described.	Modified accordingly. We made sure the strain names were consistent with the ones in the legend.
Figure 6: Excellent and compelling.	Thanks

Figure 8: As noted above, explain why primase copy number does not correlate with excised StCI despite being located on the island.	In Figure 8, strain DGCC7710-StCI containing the regulatory *orfs* of StCI-301 was used. Indeed, the results show that this chimeric StCI can only be excised by phage 2972, but there is no replication, since the primase levels are the same as the reference genes. The cq values were normalized to the reference genes and then to the non-infected control, using the ΔΔcq method. There may be an incompatibility with the new regulatory *orfs* that impedes the replication process, we added this hypothesis in lines 465-468.
Figures S18 and S19: The depiction in S18A is confusing; it appears to show the result rather than the experimental setup, which is misleading as panel A. Consider switching panels, so the experimental design comes first and clearly label outcomes. If S19 is an extended view of S18, please state this explicitly or combine them into one figure.	We have now switched panels in Figure S18. Indeed, S19 is an extended view of S18. We have now stated this explicitly in S19 legend.
Minor revisions Line 59: "gram-positive" → "Gram-positive"	Modified accordingly.
Line 98: "was not" → "were not"	Modified accordingly.
Line 342: "an increased" → "an increase"	Modified accordingly.
SP vs StCI: Consistently use "StCI" throughout, including figures and legends (e.g. lines 161, 166, 168, 169, 172–185).	Modified accordingly. However, for lines 169-171 and 179-186, we kept "phage satellites" since they are not in *S. thermophilus*, thus they are not StCIs.
Line 212–214: The statement about phages evolving to bypass restriction should be supported with data or omitted.	We initially added this hypothesis to bring an evolutionary component suggested by another reviewer. We do know that the StCI of SMQ-301 provides phage resistance to phages infecting DGCC7710 (Figure 5). However, phage DT1 and 73 titers are not affected by the presence or absence of the SMQ-301 StCI. We have now clarified this section and stated explicitly that it is a hypothesis. See lines 320-325.
Lines 239–242: Consider moving this into the earlier experiment description (lines 223–224) for better narrative flow.	Modified accordingly.
Lines 252–255: The inability to circularise the linear StCI might explain poor replication and low integrase dosage, preventing integration. This possibility should be discussed.	We added a statement to discuss this topic in Lines 252-254: *"The linear StCI might fail to circularize, which could lead to poor replication and low integrase dosage, thus preventing integration."*
Lines 385 ff.: The choice of a non-inducible expression system should be justified. While such systems may not yet exist for *S. thermophilus*, inducible tools (e.g. anhydrotetracycline-regulated) have been developed in this species (see PMID: 37191512).	We added a justification. *"To our knowledge, there was no tightly inducible-expression system available in S. thermophilus when we conducted the experiment, thus we used pNZ123-ERY which has constitutive expression."* See lines 416-418.
Suggestions for future work (not required for acceptance) Lines 357–369 note that orf33 is a MuGam-like end-protection protein. If Orf33 affects	Thanks for your relevant comment. It is likely that Orf33 can affect phage DNA replication, and its role is not restricted to only induce the StCI-301. We are currently working on this perspective.

phage DNA replication rather than just StCI induction, this could impact plaque formation. Future work could test whether orf33 complementation in both donor and recipient restores phage replication and StCI induction. This would help clarify whether Orf33 is required primarily for island derepression, phage replication, or both. This is a suggestion only and not essential for the current manuscript.	
Conclusion<br>This revised manuscript contains valuable insights into StCI biology and the role of phage-encoded inducers. While the main conclusions are well supported, the manuscript would benefit from improvements in clarity, consistency, and figure annotation to ensure accessibility to a broader readership. I look forward to seeing a revised version addressing these concerns.	Thanks for your comments, they were definitely helpful to improve the manuscript.

**Reviewer #2 blue comments in attachment**	**Our answers**
Figure 5 has very darked background and I can see some plaques formed, but it is not comparable as contrast is not the same throughout all the plates.	We adjusted the contrast values, see new Figure 5. We previously used different contrast values to see the plaques better. Plaques on DGCC7710::StCI-301-CAT strain are likely phage mutants, since all phages isolated on this strain (through full plate assays) had a mutation in their *orf33*.
I would also like to encourage the authors to call these elements phage satellites and not satellite phages to keep consistency with terminology and not provide confusion that these elements are cryptic or non-functional, as this satellite phage is typically used in these terms.	Modified accordingly. They are now called phage satellites.
But then it says “Thus, StCIs can be spontaneously induced, even in a new host, at low induction levels and independent of the growth phase.” So it is spontaneous or not?<br><br>Why is the replicase following a different pattern that the excision of the element?	StCIs are spontaneously induced as there is excision in several non-phage infected strains. See Figures 2 and S9 as well as lines 291-300.<br><br>Since there is one integrated StCI copy in the chromosome, like the reference bacterial genes (*gyrA* & *gyrB*), there is a similar quantity of StCI replicase/primase and the reference bacterial genes in non-infected strains (Supp. Figure 8). StCI can occur spontaneously without any phage infection (as shown in Supp. Figure 9) but it is a rare event. Therefore, not all the *St* cells will have an excised StCI, but all *St* cells have at least one copy of the StCI (and the primase) and the reference genes. See also Table S3.

In the ΔΔcq method (recommended by Bio-Rad), the samples were normalized against the reference bacterial genes and then to a calibrator sample (the non-infected control). In Figure S8, the targets are normalized against the reference genes and then to the sample collected at an O.D. of 0.2 (calibrator sample). Since there is no significant difference in the spontaneous induction across different growth phases, all the targets follow the same pattern regardless of the growth phase.

In Figure S9, we used the Δcq method, where the targets are only normalized against the reference genes: we did not have a calibrator sample in this experiment because all target comparisons were in the same strain. Our results show that for each strain tested, almost all the StCIs were integrated in the chromosome (1 copy/cell), reflected by the same levels of StCI primase and reference bacterial genes. The excised StCI levels are very low compared to the reference genes, indicating that StCI (spontaneous) excision is a rare event. In accordance, empty attC sites were also rare.

However, in Figure S17, the StCI-301 is induced by a virulent phage, and the targets are first normalized against the reference genes and then to the non-infected control (ΔΔcq method). Upon induction by the wild-type phage 2972 (30 min post-infection), there is approximately a 23-fold increase of StCI primase levels compared to the reference bacterial genes and to the non-infected control. This suggests that there is approx 23 StCI copies per cell following induction, compared to the single integrated copy per cell observed in the non-infected control. In the non-infected condition, the excised form of StCI is rare (substantially less than one copy per cell, as shown Figure S9). Therefore, when excised, StCI levels from the phage-infected samples (in which all StCIs were excised and replicating) were normalized to those from the non-infected control, the resulting fold change appeared even higher than that observed for the primase. This reflects both the increase in copy number due to replication and the near absence of excised StCIs in the baseline condition. We do understand that it could be confusing, thus we added more explanations (lines 399-412) and a new Table S3 that contain the cq values of the qPCR experiments. The cq values of the excised StCI were similar to the primase values in the infected samples (with phage 2972 wt). We also included in this rebuttal two new figures containing the cq values only normalized to the reference genes (Δcq method) (See Figures 1 and 2 below in this rebuttal).

	However, we would prefer to keep the ΔΔcq method (using a calibrator sample) in the manuscript, since it is a statistically more robust method and was recommended by Bio-Rad.
Thanks for addressing that the lytic phages used in this study can indeed induce the PICI. Perhaps it would be useful to generate a figure where you describe how the spontaneous induction cycle works and how the induction by a lytic phage happens. I think readers will benefit greatly.	We included a graphical abstract to illustrate these concepts.
Thanks for addressing this. Why is the replicase not following the pattern of excision? In supp fig 20 we could speculate that that ORF33 is helping excise the PICI, but it seems that its replication, verify with the qPCR of replicase gene, has very little expression..... has this been measured also with the original PICI that is spontenously induced? as technically it excises and replicates basally, seen by the constant bands on the gel in SuppFig 20A.	We have not done RT-qPCR. We have done qPCR on extracted genomic DNA. Thus, we estimated target quantity, not their expression. See lines 385-412.<br><br>In Figure S20, the strain SMQ-1528, which is strain DGCC7710 containing the StCI-301 regulatory *orfs*, was used. Supp. Figure 20B shows that the constitutive expression of ORF33, on a plasmid, increase the relative quantity of empty attC sites in SMQ-1528, which suggests that ORF33 triggers StCI excision. In Figure 8, indeed, the results show that this chimeric StCI can only be excised by phage 2972. We showed in Figure 8, that there was no StCI replication, since the replicase/primase levels were the same as the reference genes. The cq values were normalized to the reference genes and then to the non-infected control, using the ΔΔcq method. There may be an incompatibility with the new regulatory *orfs* that impedes the replication process, we added this hypothesis in lines 465-468.<br><br>The excised StCI and primase levels of the original StCIs (in DGCC7710 and in SMQ-301) have been measured in non-infected strains in Figure S9. It reveals that spontaneous induction is a rare event. In this figure, the targets have been normalized only against the reference genes (Δcq method).<br><br>Since we did the qPCR on DNA samples, the quantity of the primase is similar to the reference genes in the non-infection conditions. Indeed, there is one integrated StCI per chromosome (unless there is a rare spontaneous excision event). There is also one copy of each reference gene per chromosome.<br><br>The Orf33 induces StCI-301 in Figure 6 and Figure S17, Panels B and C (excision + replication). When there is a mutation in Orf33, there is no difference in StCI excision and replication levels compared to the non-infected sample (Figure 6 and Panel A from Figure S17).<br><br>We clarified the difference observed in excised StCI vs primase levels (lines 399-410).

	As mentioned above, we have added Supp. Table S3 containing the cq values. We are also providing Figures 1 and 2 in this rebuttal. We have also modified all the qPCR Figures (Figure 8, Supp. Figure 8, 9 and 17) to improve clarity.
Thanks for the experiment. However, this doesn't overrule that the PICI could be transferred via phage capsids. Perhaps examined the phage capsids by sequencing is a more realistic way. I am not suggesting you need to do this; however, I believe the manuscript would benefit greatly if you could include a better discussion on this finding. Importantly, because these elements are phage-induced, one would quickly assume that it will travel by parasitizing the phage, but in contrast it increases its DNA concentration to then be transformed into the host. This is an extremely odd strategy that will create an exciting discussion amongst the experts on the field. Another aspect, PICIs have shown to be induced by phages but not been packed by the same prophage. Perhaps there is a incompatibility with the phage used and that's why the WT DGCC7710 is not induced.	We tried to purify phage 2972 amplified on strain DGCC7710::StCI-301-CAT to send for DNA sequencing, but because there is a phage resistance mechanism, the titer was too low and we did not see any band on a CsCl gradient. Even when conducting a regular phage DNA extraction on this phage lysate, the DNA yield was too low for sequencing.<br><br>We showed that Orf33 from phage 2972 is binding to the StCI-301 Stl protein and the DGCC7710 StCI has a different Stl protein. It could explain the incompatibility that you are mentioning. We added this hypothesis in the discussion (lines 465-468).<br><br>We agree that a better discussion about the mobilization through phage capsids is needed. Therefore, we added a discussion on this (lines 513-528).
Thank you for addressing this. Indeed, the authors show the protein-protein interaction. However, Fig8 suggests that the replication cycle is not efficient or not comparable to the excision of the element.	In Figure 8, strain DGCC7710-StCI containing the regulatory *orfs* of StCI-301 was used in this experiment. Indeed, the results show that this chimeric StCI can only be excised by phage 2972, but there is no replication, since the primase levels are the same as the reference host genes. The cq values were normalized to the reference genes and then to the non-infected control, using the ΔΔcq method. There may be an incompatibility with the new regulatory *orfs* that impedes the replication process, we added this hypothesis in lines 450-455.<br><br>The Orf33 from phage 2972 induces the StCI-301 in Figure 6 and Figure S17 Panel B and C (excision + replication). When there is a mutation in Orf33, there is no difference in the StCI excision and replication levels compared to the non-infected sample (Figure 6 and Panel A Figure S17).
The PICI DNA is cloned into the plasmid, and this application shows that PICIs can be transformed to then activate their integrases to excise and integrate in the chromosome. In that manuscript, WT SaPIs and EcCI are transformed to then excise and integrate into the host. Then the same proof of concept was used for engineered PICIs. I would leave this matter to the editors.	We showed, without electroporating plasmids, that the entire StCI DNA (from a ligation product or an inducing-phage lysate) can integrate into its specific integration site (attC) in another strain using natural transformation. In addition, transferring new DNA into industrial strains requires careful consideration as the use of genetically engineered organisms (GMOs) has generally a negative perception to consumers. The European regulatory authorities for

	example do not classify strains as GMOs when genes are mobilized via transduction, conjugation, or transformation, provided the genetic material originates from the same species as that of the recipient strain. Thus, transferring a StCI from one strain to another through natural competence to enhance phage resistance would not be classified as a GMO. We also showed this concept by using a phage lysate as a template in natural transformation, mimicking natural settings.
What region on the PICI is this spacer targeting? Is it an accessory gene or a functional gene?	Mostly functional genes (integrase and regulatory region). There is one spacer targeting a gene coding for a hypothetical protein. We have now specified the functional module for the relevant spacers. See lines 176-182.

**Reviewer #2 comments**	**Our answers**
I appreciate the authors' efforts in addressing the previous comments and providing additional experimental data. The change in nomenclature to StCI (*Streptococcus thermophilus* Chromosomal Island) is a welcome improvement for consistency within the field.	Thank you for your comment.
However, despite the new data, I remain largely unconvinced regarding the complete lifecycle of the StCI, particularly its replication. While the Illumina experiment (Figure 6 and Supp. Figure 17) and qPCR data (Figure 8) indicate increased excision after phage infection, the evidence for robust replication of the entire element is still not as clear as would be expected for a successful transfer mechanism.	The Illumina sequencing results (Figure 6), using strain SMQ-1512, show the StCI-301-CAT replication over time following the infection by phage 2972. The read coverage from the *attL* to *attR* sites, which is the whole StCI, increased over time and the read coverage on the flanking regions (outside the StCI region) stays at the same level during the same period, which indicates that there are more StCIs than bacterial DNA following the phage 2972 infection. These results are strongly consistent with the qPCR results obtained from the same DNA samples, which showed that the StCI primase/replicase levels also increased over time during infection by phage 2972 (Supp. Figure 17). Our results also showed that the phage mutant 2972-Orf33 cannot induce the StCI-301-CAT.<br><br>We believe that they may have been a confusion with the qPCR results from Figure 8, which were conducted on a different strain (SMQ-1528). The DGCC7710-StCI containing the regulatory *orfs* of StCI from strain SMQ-301 was used in this experiment. Our results showed that this chimeric StCI can only be excised by phage 2972, but there is no replication, since the primase levels are the same as the reference genes.<br><br>We added the strain numbers in Figures 6, 8, and Supp. Figure 17 to improve clarity. We have also

	modified all the qPCR figures (Figure 8, Supp. Figure 8, 9 and 17) to improve clarity.
Specifically, I note that the replicase expression in Figure 8 (assuming this refers to the qPCR of the replicase gene) does not appear to follow the same pattern as the excision of the element. This discrepancy raises questions about the efficiency of the replication cycle, and perhaps this is why the authors cannot transduce the element (maybe). For an element to be successfully transferred, extensive replication is crucial to generate sufficient copies for packaging and transfer. The current data, while showing some induction and excision, does not fully demonstrate the prolific replication necessary to support a complete satellite lifecycle.	As indicated above, in Figure 8, the strain DGCC7710-StCI contains the regulatory *orfs* of StCI from the strain SMQ-301 (strain SMQ-1528). Indeed, our results showed that this chimeric StCI can only be excised by phage 2972, but there is no replication, since the primase levels are the same as the reference bacterial genes. There may be an incompatibility with the new regulatory *orfs* that impedes the replication process, we added this hypothesis in lines 465-468.<br>The transduction and the natural competence experiment using phage lysates were conducted with the strain DGCC7710::StCI-301-CAT (SMQ-1512), which is a different strain from SMQ-1528, and we showed that it is induced (excision + replication) by phage 2972 (Figure 6 and Supp. Figure 17).
The evidence for efficient replication of the entire StCI, comparable to what is seen in PICIs, PICMIs, P4s and PLEs, is still lacking. I would like to encourage the authors to further discuss this apparent disconnect between excision and replication, and perhaps explore why the replicase gene's expression pattern differs from the excision profile. This would significantly strengthen the manuscript's conclusions regarding the StCI's lifecycle and its classification.	As indicated above, the Illumina sequencing results (Figure 6), using SMQ-1512, clearly show the StCI-301-CAT replication over time following the infection by phage 2972. The read coverage from the *attL* to *attR* sites, which is the whole StCI, increases over time, and the read coverage on the flanking regions (outside the StCI region) stays at the same level during the same period, which indicates that there are more StCIs than bacterial DNA following the phage 2972 infection. A confusion may have arisen Figure 8, which used a different strain (SMQ-1528). The DGCC7710-StCI containing the regulatory *orfs* of StCI-301 was used in this experiment. This chimeric StCI can be excised by phage 2972, but there is no replication, as the primase levels are the same as the host genes. We believed to have clarify this by adding the strain numbers in Figures 6, 8, and Supp. Figure 17. We have also modified all the qPCR figures (Figure 8, Supp. Figure 8, 9, and 17) to improve clarity.<br><br>In Figure S17, the StCI-301 is induced by a virulent phage, and the targets are first normalized against the reference host genes and then to the non-infected control (ΔΔcq method). Upon induction by the wild-type phage 2972 (30 min post-infection), there is approximately a 23-fold increase of StCI primase levels compared to the reference genes and to the non-phage infected control. This suggests that there is approx 23 StCI copies per cell following induction, compared to the single integrated copy per cell observed in the non-infected control. In the non-infected condition, the excised form of StCI is rare (less than one copy per cell, as shown in Figure S9). Therefore, when excised, StCI levels from the phage-infected samples (in which all StCIs were excised and replicating) were normalized to those

	from the non-infected control, the resulting fold change appeared even higher than that observed for the primase. This reflects both the increase in copy number due to replication during phage infection and the near absence of excised StCIs in the baseline condition. We do understand that it could be confusing, thus we added more explanations (lines 399-410) and a new Table S3 that contain the cq values of the qPCR experiments. The cq values of the excised StCI were similar to the primase values in the phage 2972 infected samples. We also included in this rebuttal two new figures containing the cq values only normalized to the reference genes (Δcq method) (Figures 1 and 2 below). However, we would prefer to keep the ΔΔcq method (using a calibrator sample) in the manuscript, since it is a statistically more robust method and was recommended by Bio-Rad.
This aspect is central to classifying these elements as novel phage satellites or understanding their complete mechanism of transfer. And while the transformation experiments seem to prove their transfer by this mechanism, the authors do not discuss the great implications that this mean of transfer would have on the field. Having a phage-induced element without transferring via viral particles seems like an extremely odd strategy after demonstrating that the StCI can interfere with the phage.	We added a discussion on this topic in lines 513-528. We don't rule out the possibility that other phages could package the StCI-301-CAT, but phage 2972 seems incompatible to package the StCI-301-CAT. We showed that the concentration of StCI-301-CAT increased after its induction by phage 2972, which, upon cell lysis, releases enough StCI copies to be taken up by natural competence and transferred to another host.

**Reviewer's #3 comment**	**Our answer**
The authors have now satisfactorily addressed all the points I had raised in the two previous rounds of revisions. I commend the authors for the additional analyses that make the study and its results much more robust.	Thank you.

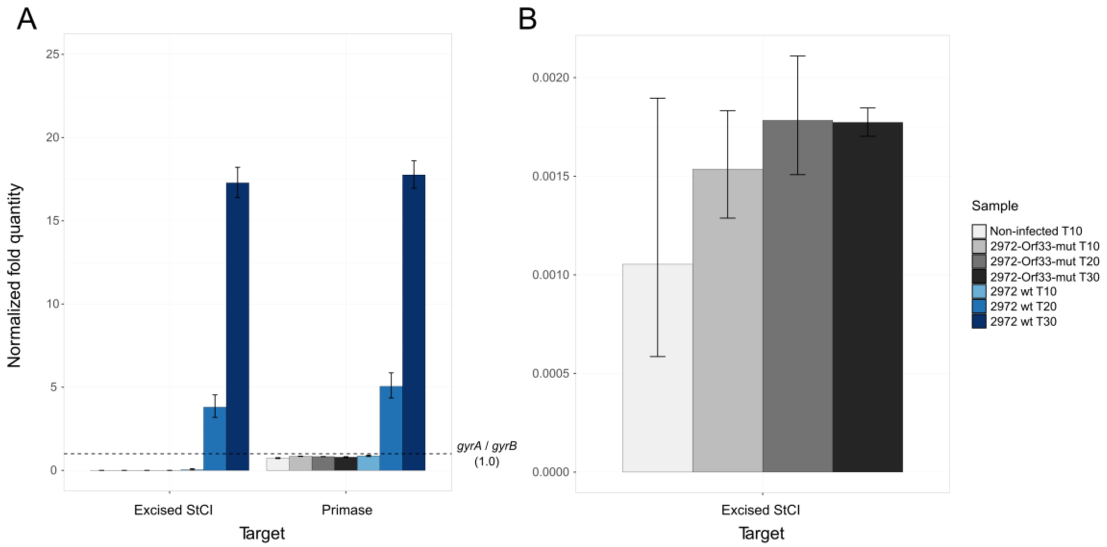

Figure 1: Relative qPCR results to quantify the StCI excision and replication in strain *S.t.* DGCC7710::StCI-301-CAT (SMQ-1512) when infected with wild-type phage 2972 and phage mutant 2972-Orf33-mut over time (T10, T20, and T30). The relative qPCR experiment was conducted on extracted genomic DNA, using primers targeting the StCI *pri* gene (primase), the excised StCI, and the housekeeping genes *gyrA* and *gyrB*. The relative quantity of each target was normalized to the housekeeping genes *gyrA* and *gyrB* using the ΔCq method[76]. Error bars represent the mean ± standard error of the mean and were calculated on log_2-transformed data as previously described[72]. Panel A, qPCR data for all the tested conditions. Panel B, zoom on the qPCR data from Panel A for the excised StCI target in the non-infected sample and in the samples infected with phage 2972-Orf33-mut.

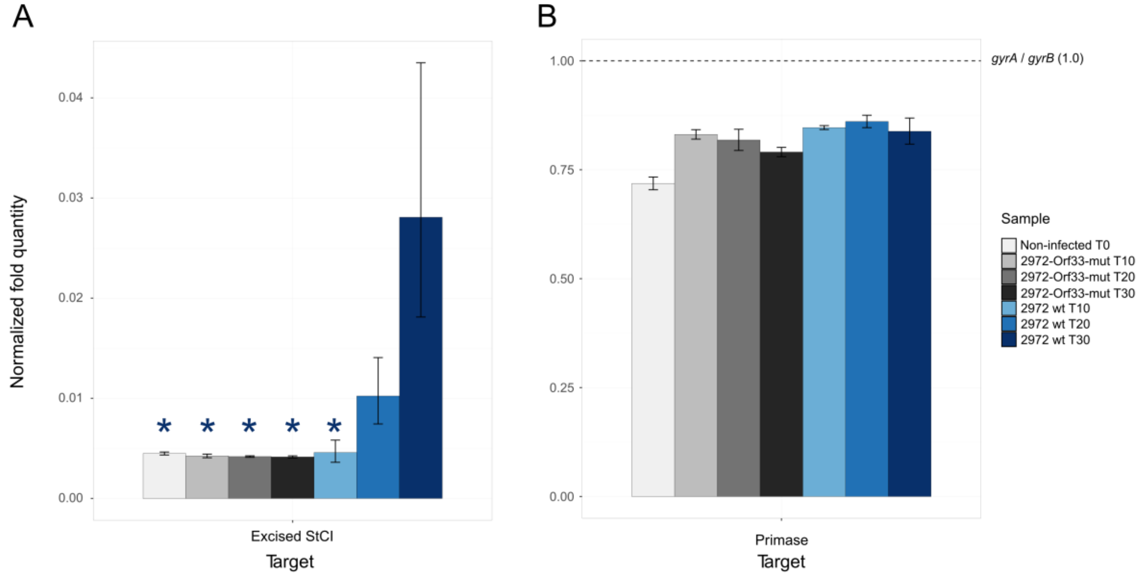

Figure 2: Relative qPCR results to quantify the StCI excision and replication in strain *S.t.* DGCC7710 containing the StCI-301 regulatory orfs (SMQ-1528) when infected with wild-type phage 2972 and phage mutant 2972-Orf33-mut over time (T10, T20, and T30). The relative qPCR experiment was conducted on extracted genomic DNA, using primers targeting the StCI *pri* gene (primase), the excised StCI, and the housekeeping genes *gyrA* and *gyrB*. The relative quantity of each target was normalized to the housekeeping genes *gyrA* and *gyrB* using the Δcq method[76]. Error bars represent the mean ± standard error of the mean and were calculated on $\log_2$-transformed data as previously described[72]. An asterisk above a condition indicates that there is a significant difference (p-value ≤ 0.05) between this condition and the one sharing the asterisk shade/color (from the same target). Each condition with an asterisk has a lower normalized fold quantity value than the color/shade matching condition. Panel A, qPCR data for the excised StCI target. Panel B, qPCR data for the StCI *pri* target.

Reviewer #2 remarks	Our answer
The manuscript presents a substantial revision with further experimental analysis of *Streptococcus thermophilus* chromosomal islands (StCIs), addressing their excision behaviour, inducibility by lytic phages, transfer via natural competence, and associated phage resistance phenotypes.	Thank you!
At the same time, I believe several conclusions still require further clarification or experimental support to ensure they are fully aligned with the data presented.	See below
While spontaneous excision of the StCIs is clearly demonstrated, the data suggests that such events occur at low frequency and without detectable replication under non-phage conditions. In non-infected cells, the StCI primase copy number remains comparable to single-copy chromosomal genes, suggesting that StCI activity is limited to excision rather than amplification. Clarifying this distinction more explicitly throughout the manuscript would help avoid overinterpretation of spontaneous StCI activity like other phage satellites and PICIs have.	We understand the reviewer concern. We added a few words to make it clearer to the reader in the text. First, we added "with no detectable replication" in line 304 and we added new sentences.<br><br>Lines 296-304: "*Our results show that spontaneous excision is a rare event, since the relative quantity of the excised StCI and empty attC sites were significantly lower than the integrated StCI and the StCI primase in all tested strains (Supp. Figure 8 and Table S4). The empty attC sites were also significantly lower than the excised StCI in all the strains. However, the empty attC site target had low cq values in the DGCC7710 strain (< 30 cq), which makes the corresponding relative quantities less reliable. The StCI pri levels were not significantly different than the housekeeping genes gyrA and gyrB and the integrated StCI targets. Thus, StCIs can be spontaneously excised, even in a new host, at low excision levels with no detectable replication, and independent of the growth phase.*"<br><br>Lines 392-398: *"The relative qPCR results showed that only the wild-type phage 2972 triggered StCI-301-CAT excision and replication. Indeed, there was a 17503-fold increase of excised StCIs and 23-fold more StCIs (StCI primase gene was used as a proxy of the total StCIs to quantify its replication) in the sample infected by phage 2972 at 30 min post-infection compared to the non-infected sample (T10) (Supp. Figure 15D). In the non-infected condition, the excised form of StCI was rare as most of the StCIs were integrated in the bacterial chromosome at levels comparable to the reference host genes (Supp. Figure 8).*
StCI replication is inferred primarily from increased primase copy number during phage infection. Although this is	The StCI-301 replication state has been validated twice with different assays. You can find below, the explanation of the two different methods:

a reasonable proxy for copy number, replication itself is not directly measured. In addition, the large fold changes observed—particularly for excised StCI forms are strongly influenced by normalisation to very low baseline levels, which complicates biological interpretation. Presenting these results with additional emphasis on absolute copy number estimates or raw qPCR metrics could help readers better contextualise the extent of replication (if any). In line with this, the graphical abstract should be modify as the replication state has not been validated and this figure could be representing a typical mechanism from phage satellites which the StCIs might not use.	**Relative qPCR methods**: Because the StCI is initially integrated in one copy in the chromosome, normalizing the StCI primase against bacterial housekeeping genes is a suitable method for quantifying StCI replication levels. The quantification of PICI replication using relative qPCR was also conducted in relevant PICI/PLE studies (Ubeda et al., 2008; Maiques et al., 2007; O'Hara et al., 2017). We also included in this rebuttal a figure containing the cq values only normalized to the reference genes (Δcq method) (Figure 1 from this rebuttal). We have also added a new panels containing the cq values only normalized to the reference genes (Δcq method) (Supp. Figure 15 panels B and C). However, we would prefer to also keep the ΔΔcq method (using a calibrator sample) in the manuscript, since it is a statistically more robust method and recommended by Bio-Rad. *The raw qPCR metrics were already available in the now Table S3.* The extend of the StCI replication, when induced by the virulent wild-type phage 2972, is approximately 23-fold, as specified in lines 392-396: "*The relative qPCR results showed that only the wild-type phage 2972 triggered StCI-301-CAT excision and replication. Indeed, there was a 17503-fold increase of excised StCIs and 23-fold more StCIs (StCI primase gene was used as a proxy of the total StCIs to quantify its replication) in the sample infected by phage 2972 at 30 min post-infection compared to the non-infected sample (T10) (Supp. Figure 15D).*"<br><br>**Illumina sequencing:** We extracted the whole DNA from non-infected cells as well as from cells infected with either the wild-type virulent phage 2972 or the phage-2972-orf33-mutant across multiple timepoints (Figure 6). The sequencing results clearly showed that the whole StCI region had an increasing read coverage over time only in the samples infected by the wild-type phage 2972. When comparing the read coverage of the StCI region with the flanking regions outside of the StCI, the replication levels are consistent with the qPCR data. Sequencing datasets are available under the BioProject ID PRJNA1260446.
The mobilisation of StCIs via natural competence is convincingly demonstrated under controlled laboratory conditions using circularised DNA and competence induction. However, the authors do not address how frequently or efficiently such transfer might occur outside of these experimental settings. Framing	Indeed, we showed under laboratory conditions that free StCIs from an inducing-phage lysate can be mobilized via natural competence in another strain. This was achieved using conditions that mimic natural environments, including growth in milk (the ecological niche of *S. thermophilus),* the use of untreated phage lysate as the StCI DNA source, and the addition of a peptide naturally produced by *S. thermophilus*. Our results strongly

this explicitly as proof-of-principle mobilisation under defined conditions would help align the conclusions with the scope of the experiments.	suggest that process could occur under natural conditions, as the experimental parameters closely resemble those found in the environment. We have now explicitly stated that it is a proof-of-principle in lines 508-510: "*Taken altogether, this experiment is a proof-of-principle that a StCI-inducing virulent phage enhances StCI mobility through natural competence and facilitates its specific integration in a new host.*"
Finally, although acquisition of StCIs correlates with increased resistance to several virulent phages, the underlying mechanism of resistance remains unresolved. The data do not distinguish whether resistance arises from StCI replication burden, direct interference with phage development, or indirect effects associated with island induction. Importantly, excision alone is unlikely to be the cause for phage resistance, as excision without dedicated interference functions would not be expected to substantially impair phage propagation. In well-characterised phage satellites with dedicated interference genes reduces phage progeny typically range from 100 to 1000-fold, reflecting active inhibition rather than excision per se. The resistance reported here may be involve an additional, as yet uncharacterised mechanism linked to StCI induction or to StCI interactions beyond excision alone (perhaps metabolic). Further clarification of this point, either experimentally or within a discussion of alternative mechanisms, would strengthen the interpretation of the resistance phenotype.	The molecular basis of the observed phage resistance is beyond the scope of this study. Accordingly, we do not speculate on the underlying mechanism, as it cannot be inferred solely from the lower phage resistance phenotypes reported for other PICI interference genes. However, we added this statement:<br><br>Lines 469-472: "*We also showed that the phage 2972-Orf33-mut does not induce the StCI-301 (Figure 6 and Supp. Figure 15), and it can escape the StCI-301 phage-resistance mechanism (Figure 6). Therefore, the phage-resistant mechanism activation seems to depend on the StCI induction*".
Overall, the findings support a model in which StCIs are conditionally inducible genetic elements whose excision are strongly dependent on phage encoded proteins. Further refinement of the distinction between excision and replication, clearer framing of mobilisation under experimental versus natural conditions, and additional insight into the basis of phage resistance would enhance the alignment between the data and the manuscript's conclusions.	Thanks for your comments, it helped to strengthen the manuscript.

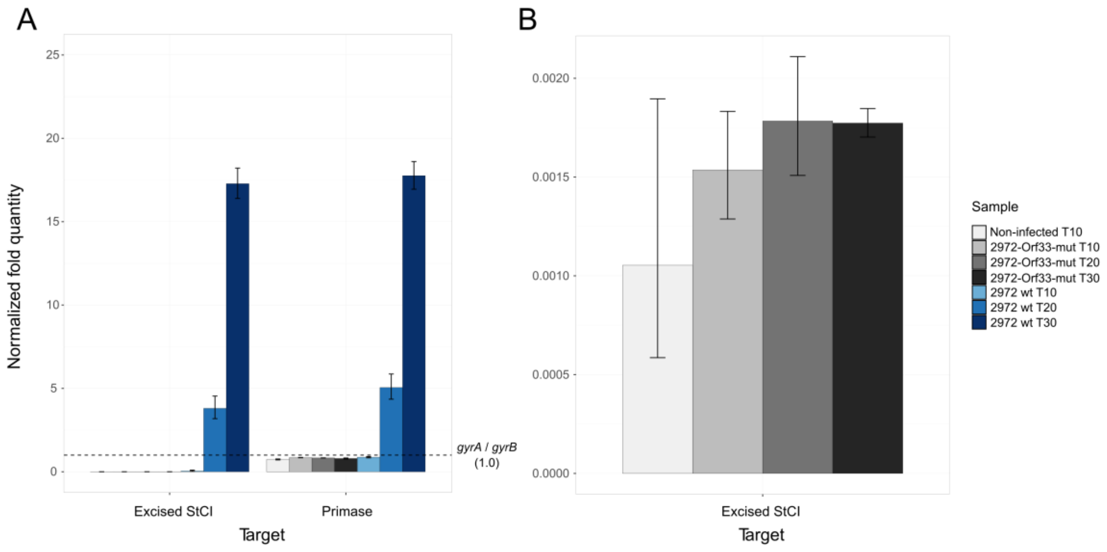

Figure 1: Relative qPCR results to quantify the StCI excision and replication in strain *S.t.* DGCC7710::StCI-301-CAT (SMQ-1512) when infected with the virulent wild-type phage 2972 and the phage mutant 2972-Orf33-mut over time (T10, T20, and T30). The relative qPCR experiment was conducted on extracted genomic DNA, using primers targeting the StCI *pri* gene (primase), the excised StCI, and the housekeeping genes *gyrA* and *gyrB*. The relative quantity of each target was normalized to the housekeeping genes *gyrA* and *gyrB* using the ΔCq method[76]. Error bars represent the mean ± standard error of the mean and were calculated on log_2-transformed data as previously described[72]. **Panel A**, qPCR data for all the tested conditions. **Panel B**, zoom on the qPCR data from **Panel A** for the excised StCI target in the non-infected sample and in the samples infected with phage 2972-Orf33-mut.

Bibliography

1. Úbeda, C. et al. SaPI mutations affecting replication and transfer and enabling autonomous replication in the absence of helper phage. *Mol Microbiol.* **67**, 493-503 (2008).
2. Maiques, E. et al. Role of staphylococcal phage and SaPI integrase in intra- and interspecies SaPI Transfer. *J Bacteriol.* **189**, 5608-5616 (2007).
3. O'Hara, B.J., Barth, Z.K., McKitterick, A.C. & Seed, K.D. A highly specific phage defense system is a conserved feature of the *Vibrio cholerae* mobilome. *PLoS Genet.* **13**, e1006838 (2017).

Comments from Reviewer #2 Our answers

The phage satellites described and used in their analyses were previously annotated as a phage satellite by Javan et al., and identified as PICIs by De Sousa et al., importantly these were classified as Type C, which depend on packaging proteins from their helper phage to mobilise. I would like to encourage the authors to follow the nomenclature already establish in the field, e.g. for E. coli is EcCIn, where first letters are referencing the specie, "CI" for chromosomal island, and "n" would be the specific strain containing the PICI.	We added the term StCI in the introduction. We conducted an extra analysis using SatelliteFinder by De Sousa et al. 2023 to have a better classification of these elements (Table S1).<br><br>Thank you for performing this analysis. As confirmed by Sousa et al. these elements can be classified as PICIs. I would like to encourage again to follow the nomenclature and use it throughout all the manuscript and not just as a brief mention.
The authors focus on the excision of the satellite. While excision is indeed important and part of the induction and packaging mechanisms of these elements, their findings lack evidence for the rest of the typical life cycle of an induced phage satellite. It is possible that they do not capture the full breadth of the interaction and excision is happening due to SOS-response after phage infection (as seen with P4 satellites and some PICIs). Typically, induction will trigger the element to excise and undergoes extensive replication, to then use its packaging genes to hijack and interfere with the helper and other resident prophages.	A friendly reminder that the excision of the SP described in this study can occur spontaneously induced without helper phages and there is no complete prophage in the strain (SMQ-301). We have clarified the text to avoid confusion.<br><br>Indeed, SP can be spontaneously induced as observed with the PICIs found in *S. pyogenes* and strains with PICIs can lack helper prophages as seen with some *S. aureus* and *S. pyogenes* isolates. I apologise if my response was suggesting that these elements can only be induced by the resident prophages. My point here is that to demonstrate that these elements are phage-inducible, or not, extensive replication is necessary to be proven. This mechanism grants the element with many copies of their DNA for a succesfull packaging/transfer and as seen with PICIs and other Satellite. Please avoid using responses as "friendly reminder". We all do our best on granting a fair review.
Evaluating replication is crucial and a standard for the field of satellites. Please see publications by Seed and Penadés where they've used either southern blots and/or qPCR to quantify and show	We have now shown the replication of the satellite phage in the semi- quantitative PCR, we have added a new figure (Figure 8).<br><br>Thank you. Based on this analysis we can see that the lytic phages can induce the PICI to

replication patterns in cell and encapsidation in phage particles.	some extend. **And this shows increased excision.** However, these PCR products were performed on the att sites and replication is still require for a succesful transfer. Therefore, either southern blots or qPCR probing a gene in the PICI will be necessary. For qPCR a chromosome gene can be use as reference for normalising data.
Furthermore, if these elements can be induced by virulent phages, then these would probably transfer in their capsids into new host cells. Which would likely interfere with the phage production and result in less viable phage particles. Was there any inspection of the phage lysate? Was the satellite DNA packaged into the capsids?<br><br>Demonstrating the transfer rates of the phage satellite is important to validate these aspects, and to showcase that these can transfer into closely related species and potentially exploit the HGT driven by their new host phages.	When we amplified the virulent phage 2972 on *S. thermophilus* DGCC7710::SP-301-CAT (DGCC7710 derivative containing the SP of strain SMQ-301), all the isolated phages had a mutation in orf33 and they cannot induce the SP- 301-CAT. We also did not observe a clear lysis during phage amplification. These results suggest that the virulent phages that can lyse the cells have quickly evolved or/and were strongly selected to prevent SP induction.<br><br>We conducted a transduction assay using phage 2972 lysate amplified on *S. thermophilus* DGCC7710::SP- 301-CAT. We added 1.8 x 106 pfu to 5.1 x 107 cfu of the strain DGCC7710-ΔSP (containing a marker to differ from cells in the lysate) and we got only one transductant. This very low frequency (0.00006%) may be generalized transduction.<br><br>We had a control with the same condition using a phage 2972 lysate amplified on *S. thermophilus* DGCC7710:: SP-DGCC7891 (with an erythromycin resistance gene). **There was no phage resistance**, so it is unlikely that this phage packaged specifically the SP of DGCC7891 and we had a higher phage titer. We used 1.4 x 108 pfu and we got 86 colonies, which represents a transduction frequency of 0.00006%, which is exactly the same as in the 2972 lysate amplified on DGCC7710::SP-301-CAT with more than 4 logs of phage resistance. Therefore, we do not think that the phage resistance phenotype is related to the encapsidation.

	Thank you for performing these. Indeed it seems that the PICIs are not able to be transfer by these phages. My main point here was to show that these PICIs are transfer by the phage as seen with other satellites, like cfPICIs and PICMIs, who don't necessarily interfere with their helper, but they are able to encapsidate and transfer to other host using the viral capsids. It would be interesting to see if phage capsids can transfer these elements.<br><br>Regarding the transformation and induction by the 2972-orf33-mut, it seems that the phage titer is 100-fold reduced. This is likely affecting the induction and transformation assays as this is likely to impact the amount of cells being destroyed and then PICI DNA released for transformation. I find this part a bit problematic, and as also mentioned by the other reviewers, it makes the transfer less reliable by this mechanism.
Additionally, they mentioned that these elements were able to excise after an overnight incubation (Line 21-22), which highlight the unstable integration or independent excision via QS signals.<br><br>I would test these aspects using the marked StCISMQ- 301 with the chloramphenicol resistance prove if this is still the case under growth selection of chloramphenicol on cultures with infection and without infection.	In fact, these SP are rather stable as they are found in most wild-type *S. thermophilus* strains for which the genome has been sequenced to date, presumably because they offer a fitness advantage (for example phage resistance). And here we show that they may be transferred from one strain to another by natural competence.<br><br>We have done an extra experiment that shows that the excision of the SP of strain *S. thermophilus* DGCC7710 is dependant on its regulatory proteins. Indeed, the SP of DGCC7710 is not induced by the virulent phage 2972. But it is induced by phage 2972 when we swapped its regulatory proteins with the ones from SP found in strain *S. thermophilus* SMQ-301. There was no chloramphenicol in this experiment (Figure 8).

	We also added an extra figure showing that in a culture of a wild- type *S. thermophilus,* the level of integrated SP is much higher that the spontaneous excised SP (Figure S15).<br><br>I have no further comments and I appreciate that the authors have performed these experiments.
The authors were able to identify mutations on ORF33 in their phages that enabled them to escape from the interference properties of the satellite, and they propose that this is the inducer of the satellite and therefore the defense system. I would argue that ORF33 is a gene that triggers or is targeted by the defence system in the satellite, as typically defence systems on PICIs and P4- satellites will carry their interference role against non- inducing phages without the induction of the satellites. (See Rousset et al., Cell Host Microbe 2022 and Fillol-Salom et al., Cell 2022). If ORF33 is indeed the inducer of the StCI, I would require seeing the protein-protein interaction with the satellite repressor (stl) to confirm the mechanism.	The focus of this paper is not on the induction mechanism, but we conducted an extra experiment showing that the phage-encoded Orf33 alone is inducing SPs by interacting with their regulatory orfs. (Figure 8) These results were added to the main text.<br><br>I find this argument a bit weak, since a big part of the manuscript focuses on the mechanism of inudction-excision-replication-transfer, which is ultimately the proof to showcase a new member phage-inducible chromosomal island or to classify these elements as a novel phage satellite. As I mentioned above, it seems phages can indeed induce excision, but we are still lacking the confirmation of the whole satellite lifecycle.
Transfer of PICIs and other satellites elements via transformation has been proven by using the concept of rebooting. I would argue that many PICIs, including those in *Streptococcus* can be acquired by transformation.	And yet, nobody as shown it by natural transformation until this manuscript.<br><br>This was shown in an application approach for engineering satellites by Ibarra-Chavez et al. BioDesgin Research (2020). As mentioned before, there is little prove of phage-mediated transfer or transformation as the differences on phage titer could have a great implications on the amount of DNA available for transforming. An MOI of 1:1 typically results in 63% of cells being infected, compared to MOI 1:10 resulting in 10% infected cells.
Since most PICIs hijack temperate phages. Where there any prophages tested in the interference and/or induction? If resident prophages are in the same strains, we would suspect those to be able to induce the satellite when infecting or being induce with mitomycin C.	There is no prophage in the *S. thermophilus* strains used in this study. This is now mentioned clearly in the manuscript.<br><br>Thank you.

Things are difficult to follow on Fig 1. are the elements cluster by phage defence system? Please provide a detail list with genes and putative roles in the figures where the genome organization is showcase. Also provide what the colour codes indicate and please take a careful look at the colour schemes used in different figures. Please consider using colour-blind friendly pallets in figure preparation (https://www.nature.com/articles/d41586-021-02696-z).	We do agree. We modified Figure 1 accordingly and we made a simpler version. The homology details between each satellite phage are also available in Supp. Figure 1.<br><br>Colors have accordingly.<br><br>been changed<br><br>Thank you. Please consider revising the manuscript once more as there are still figures that are labelled incorrectly.

**Reviewer #1 comments**	**Our answer**	**Lines**
A major problem of the study is that the authors only assess induction semi-quantitatively based on OD/CFU estimates and do not relate quantity of SP relative induction levels to a chromosomal gene. While this data suggests that more attS and attC sites are formed, it is difficult to gauge how complete this is. All evidence is PCR-based and at no point do we see evidence of induction of the whole SP. I would have expected to see evidence of the spontaneous induction of SP in Figure 7, for example through increased read coverage across the SP-301 region compared to the surrounding chromosome. This is not evident from the current representation.	We have now conducted an Illumina sequencing experiment with different timepoints during phage infection to monitor the induction of the whole StCI over time (Figure 6). Of note, the spontaneous excision is rare as we observed in our new qPCR experiment (Supp. Figure 9). We also conducted qPCR experiments to quantify the StCI induction (Supp. Figure 17).	268 – 296<br><br>370 – 384
There is also no attempt to confirm that the full length of the SP is excised and replicating. This would have been valuable data to support the ability of virulent phages to induce SPs completely and is applicable to many of the experiments that were performed using PCR.	In the above Illumina experiment (Figure 6), we have now observed a significant increase of read coverage in the whole StCI area over time when induced by the wild-type phage, indicating excision and replication.	370 – 378
Nomenclature of SP: The authors are trying to establish that virulent phages can induce these chromosomal islands, which they call satellite phages. It would be better to use a nomenclature that has been established in the field of phage-inducible chromosomal islands as indicated by reviewer 2. “The phage satellites described and used in their analyses were previously annotated as a phage satellite by Javan et al., and idenFfied as PICIs by De Sousa et al., importantly these were classified as Type C, which depend on packaging proteins from their helper phage to mobilise. I would like to encourage the authors to follow the nomenclature already establish in the field, e.g. for E. coli is EcCIn, where first lemers are referencing the specie, “CI” for chromosomal island, and “n” would be the specific strain containing the PICI.” Introducing a new independent nomenclature would complicate future cross referencing and complicate further analysis.<br>“Satellite phages can be mobilized in other strains via natural competence” and “SP mobility during phage infection”	We are now using the suggested nomenclature. We used StCI (*Streptococcus thermophilus* Chromosomal Island).	From line 101
The authors use PCR amplified and ligated SP in the experiments to assess mobilisation of the SP through natural competence (Figures 4, S5, S6). While they succeed in showing this, its relevance is not clear at this stage. In the end, this just reflects the uptake of any circular DNA into a naturally competent host. It becomes clearer once they perform their phage infection and lysate transformation experiments.	The purpose of the experiment was to show that the entire StCI DNA integrated in its specific integration site (*attC*) in another strain. This experiment was also necessary to quantify the phage resistance phenotype in Figure 5 (and Supp. Fig. 10). In addition, transferring new DNA into industrial strains requires careful consideration as the use of genetically engineered organisms (GMOs) has generally a negative perception to consumers. The European regulatory authorities for example do not classify strains	

	as GMOs when genes are mobilized via transduction, conjugation, or *transformation*, provided the genetic material originates from the same species as that of the recipient strain. Thus, transferring a StCI from one strain to another through natural competence to enhance phage resistance would not be classified as a GMO. Its relevance is of significance.<br><br>Figure 5 has very darked background and I can see some plaques formed, but it is not comparable as contrast is not the same throughout all the plates.	
How are these SPs released? Is phage infection required? How much is released in a normal supernatant by spontaneous induction? Is this enough to transform via natural competence? I would suggest combining this section (or have consecutive sections) with "SP mobility during phage infection". It might make be more straightforward to show that lysates contain transformable DNA and then provide the in vitro ligated PCR product data to confirm the type of DNA (i.e. circular DNA is required). It would fit at the end of the results to give biological relevance.	We have redesigned and redone this experiment to address these questions. The number of StCIs released by spontaneous induction in the supernatant of the non-infected control (no phage) is not enough for the StCI transfer in our experimental settings. Also, when using the phage 2972-orf33-mutant lysate, we obtained no colony that had a StCI-specific integration. We only obtained colonies that had a StCI-specific integration when using a wild-type phage lysate. Therefore, a StCI-inducing phage infection is required in our conditions to have transformable DNA that can be mobilized in another strain through natural competence (not through transduction). The DNA in the ligation product from the *in vitro* experiment could be circular and/or concatemeric, we clarified this section.	258 - 261<br><br>442 - 476
However, one important aspect that needs to be addressed here is the impact of the orf33 mutation on phage fitness. How can the authors be sure that the reduced number of transformants with this mutant is not due to fitness impacts? What were the phage titres after amplification for both? The materials and methods section states that 50 ul of lysate were used in the experiment. Given the lower phage titre and fitness impact of the orf33 mutant, could it be assumed that less DNA is released in the cells infected with mutant lysates compared to those infected with the wt strain? There is no control to monitor release of bacterial DNA as a reference for the general efficiency of DNA release. How can the authors be sure that they are providing the same quantity of bacterial lysate for both phages?	We redesigned and performed another experiment to address these issues. We infected strain DGCC7710::StCI-301-CAT with the wild-type phage 2972 or the phage mutant 2972-Orf33-mut in the same conditions (same bacterial culture, same OD_{600}, and same MOI) and we measured the final phage titers. The phage 2972-Orf33-mut had a higher titer than the wild-type phage. Thus, we transformed 30 µl of each lysate (and the non-infected supernatant as a control) and we added a condition where we transformed with the same number of phage 2972-Orf33-mut and wild-type phage 2972 in the 30 µl of lysate. We also added a control to monitor the release of bacterial DNA and no colony was obtained. When using the phage 2972-Orf33-mutant lysates, we obtained no colony that had a StCI-specific integration. We only obtained colonies that had a StCI-specific integration when using the wild-type phage 2972 lysate.	442 – 476<br><br>891 - 931

"SP-301-CAT is induced by a phage-285 encoded protein"<br>The authors show that infection with virulent phages can result in an increase in attS sites, indicative of excised and circularised SP. This is promising but requires further verification and supporting data (see below).<br>Another important consideration is how the ofr33 frameshif impacts the inducing phage. If the phage is severely impacted in terms of replication, phage titres and/or plaque sizes, loss of induction might no be due to the loss of orf33 but caused by lower overall phage reproduction and consequently lower levels of other phage proteins. From the data provided, the orf33 phage mutant is severely impaired in its replication when infecting a strain containing a non-induced SP (DGCC7710) with an almost 2-log reduction in phage titres compared to the wt phage. This alone could explain an inability of the phage to induce the SP and consequently for the uninduced SP not to provide protection against phage infection. As clarified in their response, the authors use a constitutive expression vector to drive expression of orf33. This results in the complete loss of SP sequence coverage (Fig. 7), which the authors attribute to rapid loss of the SP after induction. However, my previous contention remains unaddressed. While it is possible that orf33 induces the island, this experiment provides no conclusive evidence for this but rather only allows to conclude that expression of orf33 wt is detrimental to the presence of the SP. Is this why, whenever the authors are using this plasmid, they never show reconstitution of the attS site and focus on attC only? A better and necessary experiment to address this might have been to either infect the SP containing strain with the wt or orf33 mutant phage and monitor excision and replication over a time course following infection (including a sample before adding the phage) via Illumina sequencing. This would have shown that orf33 was important for induction of the SP.	While conducting the original semi-quantitative experiments, the phage titer of phage 2972 mutant was too low.<br><br>We have now been able to purify and concentrate the phage Orf33-mutant (as well as the wild-type phage) and conducted the requested Illumina sequencing (Figure 6). By doing so, we have showed that Orf33 is indeed essential for the StCI induction.	370 – 378
To further corroborate this or as an alternative experiment, a tightly controlled, inducible-expression plasmid would have better allowed to monitor induction following orf33 expression. Again, a time course of induction would have been appropriate and could either be monitored by Illumina sequencing or Southern blotting.	To our knowledge, there is no tightly inducible-expression plasmid available in *S. thermophilus*. However, our results from the Illumina experiment clearly show that Orf33 is essential to the StCI induction (Figure 6). We have also conducted a new experiment that show the interaction between Orf33 protein and the Stl protein of StCI-301 (Figure 9).	370 – 378<br><br>432 - 440
The experiment showing that the regulatory region of SP-301 participated in the induction process is elegant. It clearly showed that a phage protein was targeting this region. However, infection with the orf33 phage mutant was not performed and thereby	We have now shown that the Orf33 is binding to the StCI-301 Stl regulatory protein and our qPCR and Illumina experiments show that Orf33 is essential in the StCI induction (Figure 6 and Supp. Figure 17). These results	432 – 440<br><br>370 – 395

a crucial piece of evidence is missing. Again, only restoration of the attC site is monitored when expressing orf33 from a constitutive promoter not allowing any conclusion of whether it is responsible or only detrimental to the SP. Could the authors use a reporter plasmid to monitor the induction of replication genes to confirm the role of ORF33? This would also require the inducible expression or the protein to monitor induction.	corroborate with the loss of the StCI-301 when Orf33 is constitutively expressed and inducing this StCI (Supp. Figure 18).	
"SP mobility during phage infection"<br>Satellite prophages or PICI are normally packaged into phage particles when induced and can be transferred via transduction. Have the authors evaluated this? Their methodology relies on filtering lysates, which would not remove any phage particles. To exclude that any form of transduction plays a role in their observations, this should be confirmed.	To create a generalized transduction control, we first added a chloramphenicol resistance gene (CAT) into the StCI of *S. thermophilus* strain DGCC7710. We amplified phage 2972 on this strain and on the strain DGCC7710::StCI-301-CAT (a strain engineered to contain the StCI from strain SMQ-301). Then, we conducted a transduction experiment with these lysates to infect DGCC7710-ΔStCI-ΔRSR and UY03-ΔStCI-ΔRSR, two strains engineered to lack a a StCI. We obtained no colony in the UY03-ΔStCI-ΔRSR conditions. We only obtained one transductant in DGCC7710-ΔStCI-ΔRSR using the lysate of phage 2972 amplified on DGCC7710::StCI-CAT (our "positive generalized transduction control"). Thus, we do not have an efficient positive control to conduct a transduction experiment, but still no colony was observed in the recipient UY03-ΔStCI-ΔRSR strain. However, in the transformation experiment using phage lysates, we have done controls containing phages and bacteria without adding the ComS peptide (which is essential for the DNA entry in the cell by natural competence). We obtained no colony in the no-ComS conditions; thus transduction is not involved in the StCI transfer in our experiment. We also showed that phage 2972 does not adsorb to the strain UY03-ΔStCI-ΔRSR (Supp. Figure 22).	442 – 476<br><br>891 - 931
The authors state that "The virulent phage 2972 does not adsorb to the receiving strain UY03 thereby eliminating the transduction possibility." However, no evidence for this statement is provided.	We have added the Supp. Figure 22.	
I would like to see confirmation of whether SP-301 DNA is packaged into phage capsids or not.	We conducted a transduction experiment using a virulent phage 2972 lysate amplified on *S. thermophilus* DGCC7710::StCI-301-CAT. We added 1.8 x 10^6 pfu to 5.1 x 10^7 cfu of the strain DGCC7710-ΔStCI-ΔRSR (containing a marker to differ from cells in the lysate) and we got only one transductant. This very low frequency (0.00006%) may be generalized transduction.	

	We had a control using a phage 2972 lysate amplified on strain DGCC7710::DGCC7891-StCI (with an erythromycin resistance gene). There was no phage resistance, so it is unlikely that this phage packaged specifically the StCI of DGCC7891 and we had a higher phage titer. We used 1.4 x 10^8 pfu and we got 86 colonies, which represents a transduction frequency of 0.00006%, which is the same as in the 2972 lysate amplified on DGCC7710::StCI-301-CAT with more than 4 logs of phage resistance. Therefore, we do not think that the phage resistance phenotype is related to the encapsidation and the very low transduction frequency is probably generalized transduction, since phage 2972 is a *pac*-type phage.	
Tables:<br>Please ensure consistent nomenclature of all strains used in the study and verify that all strains that are indicated in the manuscript are indeed present in the relevant table. Currently, several key strains are either missing or labelled inconsistently between the text and the table.	Modified accordingly. We used StCI (*Streptococcus thermophilus* Chromosomal Island).	From line 101
Figures:<br>General comments on figures and supplementary figures:<br>The use of colours and line thickness as well as figure sizes is not very accessible. Colour coding for the different PCR products throughout the manuscript is extremely hard to see and the chosen colours are not colour-blind friendly. Only using large zoom on the pdf could colours be guessed at. Please rework this in future submissions according to the guidelines specified by reviewer 2 (https://www.nature.com/articles/d41586-021-02696-z).	Modified accordingly.	
-Figures should have a descriptive title before launching into experimental details.<br>- Please consider the use of panel labels (a,b,c, etc.) this would greatly help with identifying the relevant sections between figures and text.<br>- Please check labelling of all figures. There are multiple instances where there are labelling errors in both main and supplementary figures.	Modified accordingly.	
Figure 1 is much improved, and I am happy with the changes together with Figure S1.<br><br>Figure 2 is still missing the relevant controls for integrated SP compared to the spontaneously induced SP.<br><br>Figure S16 does not include any of the required information and relates to a different strain set.	The purpose of Figure 2 was to show that StCIs can excised from the chromosome and the sequencing of the excised PCR product allowed us to identify the *attS*, *attL,* and *attR* sites. We cannot compare the excised vs integrated StCI only based on the intensity of PCR products, since the binding efficiency may differ between primer pairs. However, we added the requested conditions in Supp	

I appreciate that the authors have evaluated spontaneous induction of one of their SPs in other growth phases. This data should be included with the main manuscript and not only as a response to the reviewers. Why was this done for only one of their SPs? How was data normalised? Is the data they are showing due to bacterial growth rather than real changes in excision?	Figure 4 and we conducted qPCR experiments to compare the integrated vs excised StCIs, between different growth phases, in the three main strains mentioned in this paper: SMQ-301, DGCC7710, and SMQ-1512. (Supp. Figures 8 and 9).	
Figure 4: SP-301 appears to be far less stable in this strain background. There is also a considerable proportion of attC still present suggesting that the island is not fully integrated. Does the cat gene cause this?<br><br>Figure S6 suggests that there are differences in stability between different strain backgrounds receiving the SP-301.	As mentioned in previous answer, we can't compare the excised vs integrated StCI only based on the intensity of PCR products. It seems that StCI-301 is less stable in the SMQ-301 strain than the StCI-301-CAT in the DGCC7710 strain background (Supp. Figure 9). Interestingly, the DGCC7710-StCI seems more stable than the StCI-301 in their respective host. However, the StCIs and the bacterial background are quite different and cannot be directly compared. In DGCC7710, there is not a clear difference in the stability between the DGCC7710-StCI and the StCI-301-CAT. These StCIs are different and cannot be directly compared. The StCI stability could be more related to the strain context rather than the CAT gene (Supp. Figure 9).	
Figure 8: Please introduce panels to better label the different elements of the figure.	Modified accordingly.	
While there is evidence that phage infection induces the chimeric SP when using phage 2972, a complementary experiment using the orf33 phage mutant would support the role of the orff33 product in inducing SP-301 regulatory element containing SPs. Expression from a constitutive promoter is not a suitable system to show that a gene product is responsible for the induction of an SP. Here, only restoration of the attC site is monitored but no evidence for the presence/absence or quantity of SP is provided.<br>Top panels: What are the green circles meant to represent? Bottom left panel: should read "SP-DGCC7710 wt" not "DGCC7710 wt no-DNA control"	We have now conducted qPCR experiments with the chimeric StCI in infected conditions (wild-type phage 2972 and phage mutant 2972-Orf33-mut), and non-infected conditions. Our results clearly show that Orf33 is essential in the StCI excision (Figure 8). We have also clarified the panels.	403 - 413<br><br>874 - 889
Figure 9: What are the green circles meant to represent?	It is the Orf33 protein. The Figure 7 and Supp. Figure 20 were modified to improve clarity.	
Figure S5: attC controls only performed for delta SP recipient but not for the strains after transformation. No control for stability of the integrated island (attS). This was done for Figure 4 and should have been also performed for S5.	We have now done the requested controls (Supp. Figure 6).	
Figures S10: There is some error in the labelling of this figure. From the experimental description in the results section, both samples were treated with phage and the difference is the presence or absence	Modified accordingly.	

of SP-301 DNA. Currently, the figure states that this was added to all samples.		
Minor comments:<br>Line 146-148: "*The sequencing of the resulting PCR products also confirmed the attC sequence for the three most common S.t. SP integration sites: ATATTTCCCTCTAAATC (IS_a), ATTTCATGAAAAAATAC (IS_b), and AATTCTACAACAAAAT (IS_c) (Table S1).*" This statement is missing context, i.e. "*As attS sites mirror attC sites, the sequencing of the resulting PCR products...*"	Modified accordingly.	142 - 148
Line 151: "*Because we could induce S.t. SPs and pinpointed the attC site,...*" This sentence is redundant as already covered in the previous sentence.	Modified accordingly.	146 - 154
Lines 169-170: "*Therefore, it is tempting to speculate that CRISPR-Cas may also serve as a defense system against SPs*." Yes, but also this could be considered evidence that these SP can move between streptococcal species pointing towards their mobility.	We have conducted an additional analysis requested by reviewer 3, and we showed that some spacers are specific to StCIs (Table S2). Thus, there is evidence that StCIs have moved between streptococcal species. We have modified our conclusions accordingly.	172 - 185
Lines 190-192: "*By conducting spot test assays, we verified if the loss of the SP in DGCC7710-ΔSP::pNZ123-RSR, UY03- ΔSP::pNZ123-RSR, SMQ-301-ΔSP::pNZ123-RSR and DGCC7891-ΔSP::pNZ123-RSR strains could increase their phage sensitivity when compared to the wild-type strains containing pNZ123*." There is something wrong with the nomenclature of the strain here. Do they really contain an integrated plasmid? Where are these strains in the strain table? Why did the authors assess the phage titres in strains with the plasmid? This is in contradiction to the previous paragraph where the plasmid was eliminated. Please correct/clarify both in the text as well as in the table.	We have modified the strain nomenclature to avoid confusion. They do not contain an integrated plasmid, they contain the RSR-plasmid. However, in this experiment, we used the strains before their plasmid loss, because the several passages without antibiotic pressure caused the accumulation of mutations in the whole genome. This section was clarified.	204 - 210
Lines 207-208: the authors could highlight that by amplifying from attL to just before attR, this would result in the complete circularised SP after ligation.	Indeed, we modified accordingly, but we also specified that the ligation reaction could also produce concatemeric StCIs.	223 – 226<br><br>258 - 261
Lines 219-221: "*In contrast, when the SP-301-CAT-rev linear PCR product was transformed into the ΔSP strains with an equivalent amount of ligated DNA from SP-301-221 CAT-rev, no colonies containing integrated SP were detected (data not shown).*" There is a reference missing: an equivalent amount of DNA relative to what?	The amount of linear PCR product was the same as the DNA used in the ligation reaction. We clarified the sentence.	236 - 239
Lines 273-283: The authors use an elegant approach to select for SPs providing phage resistance. By challenging a transformation assay either with or without the addition of circular SP-301, the effectively exploited the anti-phage properties of this island to select for successful transformation of the markerless SP. There are	We have clarified the annotation. We have tested, in a spot test assay, three purified transformants selected using phage 2972 and they all have a phage-resistance phenotype of approx. 5 logs compared to a colony that was not selected with phages. However, based only on this experiment, we cannot be certain	

some issues with the annotations in the cited figure S10 (see figure section).<br>I would also have liked to see how these newly transformed colonies performed on a spot assay and whether they would show the same levels of phage protections as the marked chloramphenicol-resistant SP.	that the StCI is solely responsible for the observed phage-resistant phenotype, since the phage interacts with other cell components. Thus, we cannot really compare this phage-resistance phenotype with the chloramphenicol-resistant StCI strain that was not in contact with any phages. This experiment only shows that phage 2972 selects StCI+ cells and we addressed the StCI phage-resistance effect in other experiments (Figure 5 and Supp. Figure 11).	
Line 308: "*Surprisingly*" I am not sure why this is surprising, please remove. Additionally, while overall colony numbers obtained are stated in the figure legend and text, there is no reference as to how many colonies/PCR products were Sanger sequenced to confirm attR and its flanking regions.<br><br>Lines 404, 410 &416: How many colonies/PCR products were sequenced for each?	All the PCR products in the Figure were Sanger sequenced. Modified accordingly (Supp. Figure 23).	

Reviewer #2

Reviewer's comment	Our answer	Lines
Thank you for performing this analysis. As confirmed by Sousa et al. these elements can be classified as PICIs. I would like to encourage again to follow the nomenclature and use it throughout all the manuscript and not just as a brief mention.	We have changed the nomenclature in the manuscript accordingly.<br><br>I would also like to encourage the authors to call these elements phage satellites and not satellite phages to keep consistency with terminology and not provide confusion that these elements are cryptic or non-functional, as this satellite phage is typically used in these terms.	From line 101
However, these PCR products were performed on the att sites and replication is still require for a succesful transfer. Therefore, either southern blots or qPCR probing a gene in the PICI will be necessary. For qPCR a chromosome gene can be use as reference for normalising data.	We have now conducted an Illumina experiment to follow the entire StCI replication over time when induced by phages (Figure 6 and Supp. Figure 17). As suggested, we have also conducted a qPCR experiment to monitor the replication of the DGCC7710-StCI containing the StCI-301 regulatory *orfs* when induced by phages (Figure 8).<br><br>But then it says "Thus, StCIs can be spontaneously induced, even in a new host, at low induction levels and independent of the growth phase." So it is spontaneous or not? Why is the replicase following a different pattern that the excision of the element?	370 – 384<br><br>403 - 413<br><br>874 - 889
Indeed, SP can be spontaneously induced as observed with the PICIs found in S. pyogenes and strains with PICIs can lack helper prophages as seen with some S. aureus and S. pyogenes isolates. I apologise if my response was suggesting that these elements can only be induced by the resident prophages. My point here is that to demonstrate that these elements are phage-inducible, or not, extensive replication is necessary to be proven. This mechanism grants the element with many copies of their DNA for a succesfull packaging/transfer and as seen with PICIs and other Satellite. Please avoid using responses as "friendly reminder". We all do our best on granting a fair review.	We have now proven the induction and replication of the StCI-301 by phage 2972 (Figure 6 and Supp. Figure 17).<br><br>Thanks for addressing that the lytic phages used in this study can indeed induce the PICI. Perhaps it would be useful to generate a figure where you describe how the spontaneous induction cycle works and how the induction by a lytic phage happens. I think readers will benefit greatly.	370 – 384
Thank you. Based on this analysis we can see that the lytic phages can induce the PICI to some extend. And this shows increased excision. However, these PCR products were performed on the att sites and replication is still require for a succesful transfer. Therefore, either southern blots or qPCR probing a gene in the PICI will be necessary. For qPCR a chromosome gene can be use as reference for normalising data.	Thank you for your comment, we have now conducted the requested qPCR experiment and the StCI-301 is induced (excision + replication) by phage 2972 (Supp. Figure 17).<br><br>Thanks for addressing this. Why is the replicase not following the pattern of excision? In suppfig 20 we could speculate that that ORF33 is helping excise the PICI, but it seems that its replication, verify with the qPCR of replicase gene, has very little expression.....	370 – 384

	has this been measured also with the original PICI that is spontenously induced? as technically it excises and replicates basally, seen by the constant bands on the gel in SuppFig 20A.	
Thank you for performing these. Indeed it seems that the PICIs are not able to be transfer by these phages. My main point here was to show that these PICIs are transfer by the phage as seen with other satellites, like cfPICIs and PICMIs, who don't necessarily interfere with their helper, but they are able to encapsidate and transfer to other host using the viral capsids. It would be interesting to see if phage capsids can transfer these elements.	We did not observe StCI-CAT mobilization through transduction in our transformation experiment using phage lysates. To create a generalized transduction control, we first added a chloramphenicol resistance gene (CAT) into the StCI of strain DGCC7710. We amplified phage 2972 on this strain and on strain DGCC7710::StCI-301-CAT. Then, we conducted a transduction experiment with these lysates to infect DGCC7710-ΔStCI-ΔRSR and UY03-ΔStCI-ΔRSR. We obtained no colony in the UY03-ΔStCI-ΔRSR conditions. We only obtained one transductant in DGCC7710-ΔStCI-ΔRSR using the lysate of phage 2972 amplified on DGCC7710::StCI-CAT (our "positive generalized transduction control"). Thus, we do not have a very efficient positive control to conduct a transduction experiment, but no colony was observed in the recipient UY03-ΔStCI-ΔRSR strain. However, in the transformation experiment using phage lysates, we have done controls containing the phages and bacteria without adding the ComS peptide (which is essential for the DNA entry in the cell by natural competence). We obtained no colony in the no-ComS conditions; thus transduction is not involved in the StCI transfer in our experiment. We also showed that phage 2972 does not adsorb to the strain UY03-ΔStCI-ΔRSR (Supp. Figure 22).<br><br>Thanks for the experiment. However, this doesn't overrule that the PICI could be transferred via phage capsids. Perhaps examined the phage capsids by sequencing is a more realistic way. I am not suggesting you need to do this; however, I believe the manuscript would benefit greatly if you could include a better discussion on this finding. Importantly, because these elements are phage-induced, one would quickly assume that it will travel by parasitizing the phage, but in contrast it increases its DNA concentration to then be transformed into the host. This is an extremely odd strategy that will create an exciting discussion amongst the experts on the field.<br>Another aspect, PICIs have shown to be induced by phages but not been packed by the same prophage. Perhaps there is a	442 – 476<br><br>891 - 931

	incompatibility with the phage used and that's why the WT DGCC7710 is not induced.	
Regarding the transformation and induction by the 2972-orf33-mut, it seems that the phage titer is 100-fold reduced. This is likely affecting the induction and transformation assays as this is likely to impact the amount of cells being destroyed and then PICI DNA released for transformation. I find this part a bit problematic, and as also mentioned by the other reviewers, it makes the transfer less reliable by this mechanism.	We understand the issue that the lysate contents might differ. Thus, we have redesigned the experiment to consider the difference in the phage titers and we amplified the wild-type phage 2972 and phage mutant 2972-Orf33-mut. Titers were normalized.<br>Because there is a phage-resistance mechanism in the DGCC7710::StCI-301-CAT, the wild-type phage 2972 had a lower titer than phage 2972-Orf33-mut.<br><br>Thank you for addressing this.	442 – 476<br><br>891 - 931
I find this argument a bit weak, since a big part of the manuscript focuses on the mechanism of induction-excision-replication-transfer, which is ultimately the proof to showcase a new member phage-inducible chromosomal island or to classify these elements as a novel phage satellite. As I mentioned above, it seems phages can indeed induce excision, but we are still lacking the confirmation of the whole satellite lifecycle.	We have now shown that the Orf33 is binding to the StCI-301 regulatory protein, through a protein-protein interaction (Figure 9).<br><br>Thank you for addressing this. Indeed, the authors show the protein-protein interaction. However, Fig8 suggests that the replication cycle is not efficient or not comparable to the excision of the element.	432 - 440
This was shown in an application approach for engineering satellites by Ibarra-Chavez et al. BioDesgin Research (2020).	Ibarra-Chavez et al. have electroporated plasmids and did not use only the PICI as the DNA template or natural transformation. Of note, their work was done in *E. coli* and *S. aureus*, but not in *S. thermophilus.*<br><br>The PICI DNA is cloned into the plasmid, and this application shows that PICIs can be transformed to then activate their integrases to excise and integrate in the chromosome. In that manuscript, WT SaPIs and EcCI are transformed to then excise and integrate into the host. Then the same proof of concept was used for engineered PICIs. I would leave this matter to the editors	
As mentioned before, there is little prove of phage-mediated transfer or transformation as the differences on phage titer could have a great implications on the amount of DNA available for transforming. An MOI of 1:1 typically results in 63% of cells being infected, compared to MOI 1:10 resulting in 10% infected cells.	We have now redesigned the experiment to address these issues. Both phages were amplified on the DGCC7710::StCI-301-CAT in the same conditions using a MOI of 5.<br><br>Thank you for addressing this, I am happy with this experiment.	891 - 931
Thank you. Please consider revising the manuscript once more as there are still figures that are labelled incorrectly.	Modified accordingly.	

Reviewer #3

Reviewer's comment	Our answer	Lines
The targeting of CRISPR-Cas to SPs. I will not argue against the existence of spacers that target the SPs, but I'm not sure I agree that knowing the DNA targeted by specific spacer is irrelevant. Some proteins of SPs have commonalities with those found in virulent and/or temperate phages (Integrases, TerS, etc). Hence, a CRISPR spacer that was acquired from a phage might target a SP as a collateral effect. On the other hand, if they target SP specific genes (like stl or the equivalent regulatory protein in the StSPs), then this would be supporting evidence that bacteria are acquired spacers directly from the SP. The reason I insist on this is also because it would be nice to have some discussion on why would bacteria actively try to prevent infection by SPs that can provide an advantage (by protecting against some phage). Since the authors already have identified the spacers, the targeted DNA (which genes, from which SPs and from which strains) should be easy to pinpoint and present, and I think it would add a nice eco-evolutionary component to the paper.	Thank you for the clarification.<br><br>We have now specified the region in the StCI targeted by the spacer and conducted BLAST analysis to verify if the spacers are found in other genomic contexts (see Table S2).<br><br>By aligning each spacer with the NCBI BLASTn database, we identified three spacers that target specifically a SP region, as they are not found in other genomic contexts.<br><br>What region on the PICI is this spacer targeting? Is it an accessory gene or a functional gene?<br><br>"Taken altogether, our results suggest that some spacers are specifically acquired from satellite phage DNA that can be mobilized between different *Streptoccocal* species."	172 – 185<br><br>538 - 541
- On the point of mobilization by transformation, I am now more convinced that is the case. I apologise that I missed the data on mobilization under more natural conditions on Figures S17 and S18 in the previous version, but it was indeed not very clear what was being tested. Nevertheless, I am still a bit puzzled on the particular experiment that uses the artificially ligated SP. I understand that this is meant to provide a laboratory-specific assay to test the concept of the mobilization by transformation. As I was reading the new version, I initially assumed that this would be because the efficiency of transfer by natural transformation using just cell lysates would be too low to detect. But the SPs do circularize by themselves, and if I understand correctly the data in the last section of results (Figure S18), transfer under natural conditions (without phage induction) occurs on average in 12.5% of the cases, which is very reasonable. Thus these conditions should be enough to generate enough SP DNA and detect the transfer, especially if there is the CAT gene to select on. So the need to add a level of artificiality using the ligated form is still not clear to me. Perhaps the authors can just add a brief rationale at the beginning of this part (lines 207-208) that could help the readers follow the reasoning for their methodological approach?	We wanted to have an experiment, in which only the StCI DNA is mobilized by natural competence to show that the entire StCI DNA integrates in its specific integration site (*attC*) in another strain. This experiment was also necessary to quantify the phage resistance phenotype in Figure 5 and Supp Figure 10.<br><br>We have now transformed the supernatant from a DGCC7710::StCI-301-CAT culture into UY03-ΔStCI-ΔRSR and we obtained no colony, thus it might not be easy to transfer StCI this way in laboratory conditions.<br><br>Indeed, based on experiment from our previous version of this manuscript, the DNA from an overnight culture of the strain containing the StCI-CAT could have been a successful DNA template to mobilize the StCI-CAT to other strains. However, during natural competence, all the DNA with homologous regions can recombine with the recipient strain DNA. Thus, the genomic DNA from the overnight culture could recombine with the DNA of the recipient strain, making it highly variable and unsuitable to properly verify the phage resistance phenotype.	219 - 242<br><br>442 – 476<br><br>891 - 931
Even if it has a technical reason. I would also like to read some justification on why two orientations for	We started working with the CAT-rev to mobilize the StCI in four strains, but we	

the CAT gene were used, as well as some speculation on why it gives different results, given that only (?) the CAT-rev version worked in all four strains. Maybe some of these points could be more strongly addressed by including the quantifications of the efficiency of transfer here (and not just in the last experiment). If colonies were selected, there must have been colonies to count. Are the number of CAT resistant (and thus SP containing) colonies obtained with the ligated version that much higher? And are there differences between the CAT and CAT-rev efficiencies?	switched to the CAT, since the gene is in the same orientation as the other StCI accessory genes. Thus, we transformed the StCI-CAT in strain DGCC7710 for the other experiments, we did not try in other strains, since we already showed the specific SP mobilization with the StCI-CAT-rev. Since our ligation experiments were not done within the same conditions for the StCI-CAT and StCI-CAT-rev, we can't compare their colony numbers and there could be a lot of variations in the amount of the different DNA conformations between the ligation reactions. The use of the two orientations of the CAT genes did not affect the phage resistance phenotype (Figure 5 and Supp. Figure 10).<br><br>We clarified this section in the manuscript and added the colony numbers related to the mobilization of the excised StCI from the (new) last experiment, which is mimicking natural settings and comparing the StCI transfer from inducing, non-inducing, and spontaneous induction conditions.	
Considering the phage resistance provided by the SP, I think it is quite convincing now. What is also interesting to me is that SP-301 provides no protection against the phages that infect its own host SMQ-301 strain (73 and DT1, lines 194-195), but it does provide resistance against phages that infect other strains. This to me suggests that some of these SPs might not only travel frequently between hosts, but might also undergo selection across multiple hosts as well (and the phages they encounter). If the authors agree, maybe some discussion could be added on this point?	We agree. Discussion was added.<br><br>"These findings demonstrate that CRISPR-Cas9 can effectively facilitate the recovery of *S.t.* cells without any StCI, and it also suggests that the phages might have evolved to bypass their host StCI phage resistance mechanism, since the presence/absence of these specific StCIs did not change the phage sensitivity profile of their host."	211 - 214
Finally, regarding the spontaneous excision of SPs. The authors suggest, in the conclusion, that SP can be lost naturally within a population ("*While the spontaneous SP excision suggests a scarcity of these MGE within the bacterial population, especially if the SP fails to reintegrate the chromosome before cell division*"). However, it would be beneficial to provide a point of speculation on whether the re-acquisition by transformation could counter-act this loss. Note that it would be extra nice to have some numbers too for this process of loss (how frequent it is) and this would add an important information for this process. But I understand it might fall out of the scope of the paper.	It is a good point, and we added the re-acquisition hypothesis in our conclusion. We added qPCR experiment that quantify the relative amount of empty *attC* site vs excised StCI vs integrated StCI in three different strains (Supp. Figure 9), but we cannot know if the element is lost in one cell, since the excised form can still be present even if there is an empty *attC* site. However, our qPCR results showed that empty *attC* sites (= no integrated StCI) are extremely rare in the three strains tested (Supp. Figure 9).	284 – 296<br><br>491 - 492
- Line 98: "... seemingly functional SP..." -> Not wanting to be annoying, but I still think there is no obvious evidence (at this point in the text) that it is "functional". I would just say "a putative SP".	Agree. Modified accordingly.	89

The SMQ-310 and DGCC7710 strains have no prophages, as stated by the authors. Maybe I missed it, but what about the other strains from Figure 2? Can you state from early on (e.g., lines 138-139) whether this is the case or not?	We have now specified if prophage are detected in the genomes available on GenBank.	136 - 137
Line 161: "... or they have a different SP than the ones targeted by the spacers". Meaning a different group, or just different SPs in general?	A different StCI in general that is not targeted by the spacer. Text was clarified.	161 - 163
Line 196-197: Add *"... and that these SPs provide no protection against infection by the tested virulent phages*" Otherwise, the previous paragraph seems out of place. What is the rationale for testing phage-resistance here? Why no conclusion? I think this is supposed to provide a complementary point to the experiment on phage resistance later on, but here it seems a bit lost.	It was to verify if the StCI from the phages' hosts could provide phage resistance. We added a conclusion to clarify this point.	204 - 214
Line 207: Figure 4, instead of Figure 2?	Modified accordingly.	
- Lines 311-312: "*The orf33 is a non essential gene and it matched a putative..."* -> Has homology with?	Modified accordingly.	358 - 360
Lines 429-430: "*We found that swapping SPs enhance the overall phage resistance of a S.t. strain.* " -> I would tamper this a bit and just say that "swapping SPs CAN enhance the overall phage resistance". Given that the phage-SP interaction might be specific, some pairs swapped might not give any resistance at all (and only one proper swap was tested in the manuscript).	Modified accordingly.	493 - 494
Figure S18 is out of order in the supplementary data file (I thought it was missing at first!)	Modified accordingly.	
